# Establishing a Regional Nitrogen Management Approach to Mitigate Greenhouse Gas Emission Intensity from Intensive Smallholder Maize Production

**DOI:** 10.1371/journal.pone.0098481

**Published:** 2014-05-29

**Authors:** Liang Wu, Xinping Chen, Zhenling Cui, Weifeng Zhang, Fusuo Zhang

**Affiliations:** Center for Resources, Environment and Food Security, China Agricultural University, Beijing, People's Republic of China; North Carolina State University, United States of America

## Abstract

The overuse of Nitrogen (N) fertilizers on smallholder farms in rapidly developing countries has increased greenhouse gas (GHG) emissions and accelerated global N consumption over the past 20 years. In this study, a regional N management approach was developed based on the cost of the agricultural response to N application rates from 1,726 on-farm experiments to optimize N management across 12 agroecological subregions in the intensive Chinese smallholder maize belt. The grain yield and GHG emission intensity of this regional N management approach was investigated and compared to field-specific N management and farmers' practices. The regional N rate ranged from 150 to 219 kg N ha^−1^ for the 12 agroecological subregions. Grain yields and GHG emission intensities were consistent with this regional N management approach compared to field-specific N management, which indicated that this regional N rate was close to the economically optimal N application. This regional N management approach, if widely adopted in China, could reduce N fertilizer use by more than 1.4 MT per year, increase maize production by 31.9 MT annually, and reduce annual GHG emissions by 18.6 MT. This regional N management approach can minimize net N losses and reduce GHG emission intensity from over- and underapplications, and therefore can also be used as a reference point for regional agricultural extension employees where soil and/or plant N monitoring is lacking.

## Introduction

The need to increase global food production while also increasing nitrogen (N) use efficiency and limiting environmental costs [e.g., greenhouse gas (GHG) emissions] have received increasing public and scientific attention [1]–[6]. Coordinated global efforts are particularly critical when dealing with N-related GHG emissions because such emissions and their impacts recognize no borders. The most rapidly developing countries, such as China and India, are becoming central to the issue, not only because these countries consume the most chemical N fertilizer [7], [8], but they have also become dominating forces in the production of new N fertilizers in recent decades [7], [8]. From 2001 to 2010, global N fertilizer consumption increased from 83 to 105 MT, with 83% of this global increase originating from five rapidly developing countries, specifically China (9.9 MT), India (5.2 MT), Pakistan (0.8 MT), Indonesia (1.1 MT), and Brazil (1.1 MT). In comparison, chemical N fertilizer consumption decreased by 6.5% (0.7 MT) in Western Europe and Central Europe, and increased by only 7.1% (0.8 MT) in the United States over this period [8]. Optimizing N management in these rapidly developing countries clearly has important implications worldwide.

In the past 30 years, the N application rate in many developed economies has been optimized based on recommended systems, and have included soil nitrate (NO_3_) and plant testing [9], [10], and more recently, remote sensing [11]. However, in rapidly developing countries, small-scale farming with high variability between fields and poor infrastructure in the extension service makes the use of many advanced N management technologies difficult. Fox example, the average area per farm in China is only 0.6 ha, and individually managed fields are generally 0.1–0.3 ha [12]. Therefore, the challenge is to develop agronomically effective and environmentally friendly practices that are applicable to hundreds of millions of smallholder farmers, while producing high yields and reducing N losses.

Decisions regarding the optimal N fertilizer application rate require knowledge of existing soil N supplies, crop N uptake, and the expected crop yield in response to N application [13]. Optimal N rates often vary depending on soil-specific criteria and/or crop management variables such as soil productivity, producer management level, and geographic location [14]. However, the optimal N rate will become more uniform under geographically similar soil and climatic conditions, and when the main factors causing the variation in optimal N rates are either addressed or removed [14].

Our hypothesis is that a regional N management approach could be adopted to accommodate hundreds of millions of small farmers and reduce variation among farms, increase crop yield, and lower the GHG emission intensity of maize production. In China, maize (*Zea mays* L.) is the largest food crop produced, accounting for 37% of Chinese cereal production and 22% of the global maize output in 2011 [15]. Chinese maize production results in some of the most intensive N applications globally, and the resulting enrichment of N in soil, water, and air has created serious environmental problems.

In the present study, we developed a regional N management approach across major maize agroecological regions in China. We also compared grain yield and GHG emissions between the regional N management approach and site-specific N management, and evaluated the potential for increasing grain yields and mitigating GHG emission intensity using this regional N management approach when compared to farmers' practices across each region.

## Materials and Methods

### Description of China's agroecological maize regions

**Figure 1:**
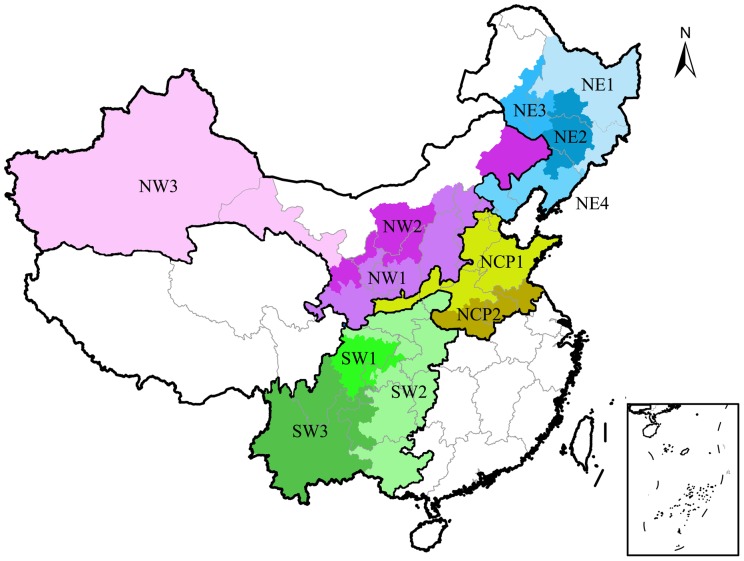
Map showing the four major maize-planting agroecological regions (thick lines, NE, NCP, NW, SW) and their subregions in China (different colors). Northeast China (NE1, NE2, NE3, NE4), North China Plain (NCP1, NCP2), Northwest China (NW1, NW2, NW3), and Southwest China (SW1, SW2, SW3). Here, we show the distribution of maize production in China; the total maize sowing area in the 12 subregions is approximately 32 million hectares, which represents 96% of the total maize production in China.

In China, maize is grown primarily in 4 main agroecological regions and 12 agroecological subregions, including Northeast China (NE1, NE2, NE3, NE4), North China Plain (NCP1, NCP2), Northwest China (NW1, NW2, NW3), and Southwest China (SW1, SW2, SW3) (Fig. 1) [16]. These agroecological subregions were divided based on climatic conditions, terrains, agricultural management practices (e.g., irrigation), and soil types. Detailed information on each of these subregions is provided in Table S1 and Text S1.

### Farmers' survey

A multistage sampling technique was used to select representative farmers for a face-to-face, questionnaire-based household survey conducted once a year between 2007 and 2009 [17]. In this study, 5,406 farmers from 66 counties in 22 provinces were surveyed (Table 1). In each province, three counties were randomly selected, three townships were randomly selected in each county, two to five villages were randomly selected in each township, and 20 farmers from the villages were randomly surveyed to collect information on N fertilizer use and grain yield in each farmer's household. This study was approved by a research ethics review committee at the College of Resources and Environmental Science (CRES), China Agricultural University, Beijing, China. Data was collected through an in-house survey, which was conducted by research staff at the College of Resources and Environmental Science. Before beginning the survey, an informed consent information sheet was given to the farmer to read (or in some cases was read to the farmer), and verbal informed consent was requested. Because this study was considered anonymous and each participating household could not be identified directly or indirectly, the research ethics review committee of CRES waived the need for written informed consent from the participants.

**Table 1:** N fertilizer application rate, maize grain yield, N balance, and GHG emission intensity of N fertilizer use, N fertilizer production and other sources in different agro-ecological subregions.

Region & Subregion	n a	N rate (kg ha^−1^)	Grain yield (Mg ha^−1^)	N balance (kg N ha^−1^)	GHG emission intensity (kg CO_2_ eq Mg^−1^ grain)			
					N fertilizer use	N fertilizer production	Other sources	Total
NE	1263	195±61	8.91±1.19	32±30	115±38	182±60	50±17	347±131
NE1	361	156±43	8.59±0.79	3±13	96±27	151±40	51±13	298±85
NE2	411	201±59	9.03±1.16	40±28	116±34	183±54	49±15	348±124
NE3	311	226±76	8.80±1.52	68±41	137±46	213±71	51±17	402±164
NE4	180	205±77	8.68±1.50	49±39	124±51	196±74	52±21	373±183
NCP	1983	208±72	7.42±1.24	61±43	148±46	233±76	55±19	436±178
NCP1	1460	206±71	7.68±1.27	54±38	141±49	223±77	54±19	418±180
NCP2	523	217±66	7.14±1.15	76±46	161±45	252±75	57±17	471±174
NW	882	238±107	7.58±1.91	95±68	170±77	261±119	56±27	487±240
NW1	394	234±103	6.93±1.70	98±73	182±80	280±123	58±26	520±282
NW2	289	246±128	8.22±2.50	91±64	163±86	248±129	54±29	466±171
NW3	199	234±83	7.15±1.48	106±47	176±62	272±96	60±20	508±257
SW	1278	250±91	5.45±1.17	144±86	251±93	381±140	78±30	710±319
SW1	427	257±83	5.41±1.13	151±71	263±85	394±127	75±24	732±225
SW2	447	232±90	5.36±1.08	129±93	232±90	358±134	84±34	675±359
SW3	404	272±101	5.59±1.32	160±103	274±100	403±152	75±28	752±375

a n: number of observations.

### On-farm field experiments

In total, 1,726 on-farm maize N fertilizer experiments in 181 counties of 22 provinces were conducted from 2005 to 2010 in the NE (*n* = 397) and NW (*n* = 416) spring maize areas, and in the NCP (*n* = 407) and SW (*n* = 506) summer maize areas. All 66 counties where farm surveys were conducted were included in these 181 counties.

All experimental fields received four treatments without replication: without N fertilizer (N0), medium N rate (MN), 50% and 150% of MN. The amount of N fertilizer for the MN treatment was recommended by local agricultural extension employees based on experience and target yield (1.1 times the average yield of the past 5 years). The median N application rates for the 1,726 sites are shown in Table 2. Approximately one-third of the granular urea was applied by broadcasting at sowing, while the remainder was applied as a side-dressing at the six-leaf stage. All experimental fields received 30–150 kg P_2_O_5_ (P) ha^−1^ as triple superphosphate and 30–135 kg K_2_O (K) ha^−1^ as potassium chloride, based on experience and target yield. All P and K fertilizers were applied by broadcasting before sowing. No manure was used, which is common for maize production in China. Detailed information regarding the N application rate and selected soil chemical properties before maize planting at 1,726 on-farm experimental sites is provided in Table S2.

**Table 2:** The number of on-farm experiments, maize yield without N, medium N rate, grain yield at the medium N rate and N rate, grain yield, GHG emission intensity of N fertilizer use, N fertilizer production and other sources for regional N management approach and field-specific N management.

Subregion	n^a^	Yield without N (Mg ha^−1^)	Medium N rate (kg ha^−1^)	Yield for medium N rate (Mg ha^−1^)	Regional N management approach						Field-specific N management					
					N rate (kg ha^−1^)	Grain yield (Mg ha^−1^)	GHG emission intensity (kg CO_2_ eq Mg^−1^ grain)				N rate (kg ha^−1^)	Grain yield (Mg ha^−1^)	GHG emission intensity (kg CO_2_ eq Mg^−1^ grain)			
							N fertilizer use	N fertilizer production	Other sources	Total			N fertilizer use	N fertilizer production	Other sources	Total
NE1	132	6.40±1.01^b^	153±6	8.98±1.09	150	8.85	91	141	49	280	158±26	8.87±1.11	91±19	149±32	49±7	289±55
NE2	62	6.82±1.23	147±21	9.05±1.51	150	9.18	87	136	49	272	155±25	9.13±1.48	87±14	143±23	51±8	281±42
NE3	126	6.50±1.26	162±15	9.48±1.38	164	9.01	96	151	50	298	165±20	9.10±1.25	92±18	153±29	50±8	295±53
NE4	77	6.92±1.66	204±28	8.93±1.59	188	8.76	113	178	55	346	191±49	8.84±1.48	117±37	183±53	57±9	356±94
NCP1	348	6.58±1.13	194±22	8.23±1.13	178	8.13	115	182	58	355	179±27	8.14±1.19	113±23	185±36	59±9	357±64
NCP2	59	6.91±1.13	213±30	8.67±0.95	177	8.37	111	176	55	342	185±33	7.59±1.12	129±30	208±45	62±9	399±80
NW1	100	6.30±1.06	190±34	8.35±1.03	181	8.13	117	185	59	360	180±44	8.13±1.12	115±29	184±45	59±9	357±77
NW2	309	8.12±1.83	190±20	10.53±1.71	176	10.38	89	141	47	277	182±34	10.48±1.82	91±25	148±37	48±9	288±68
NW3	7	7.23±1.74	221±9	10.33±1.59	219	9.83	118	185	46	349	215±16	9.85±1.56	116±20	185±30	46±7	347±57
SW1	78	5.70±1.30	217±22	7.63±1.20	174	7.46	123	194	63	379	191±39	7.56±1.19	134±34	214±53	63±11	412±93
SW2	368	5.59±1.13	195±22	7.72±1.18	183	7.71	125	197	66	387	184±38	7.77±1.26	125±33	202±52	67±12	394±92
SW3	60	6.00±1.09	207±25	8.29±1.33	186	8.10	121	191	65	376	191±37	8.38±1.33	120±28	192±44	65±11	376±78
National c	-	6.60	187	8.69	174	8.56	108	171	56	334	178	8.55	109	185	57	343

a n: number of observations.

b Mean ± SD.

c National values are computed from the regional values weighted by area. The regional weights are as follows:

NE1, 4.5%; NE2, 14.9%; NE3, 4.7%; NE4, 6.4%; NCP1, 25.6%; NCP2, 6.0%; NW1, 10.4%; NW2, 7.3%; NW3, 2.6%; SW1, 3.5%; SW2, 7.9%; SW3, 6.2%.

Individual plots were approximately 40 m^2^ (5 m wide and 8 m long). All experiments were managed (including maize variety, density, planting, harvesting, herbicide and insecticide for pests, diseases, and weeds) by local farmers based on a field manual provided by local agricultural extension employees, whereas for the treatments, local agricultural extension employees conducted fertilizer applications. The time of planting and harvest were determined by farmers and differed among sites. Generally, in NE and NW, maize was planted in early May and harvested in late September. Maize was planted from June to October in NCP and from April to August in SW. Plant densities were 50,000–65,000 plants ha^−1^ in NE, 70,000–75,000 plants ha^−1^ in NCP, 65,000–75,000 plants ha^−1^ in NW, and 45,000–50,000 plants ha^−1^ in SW. The locations of the 1,726 experiments were not privately-owned or protected in any way. No specific permits were required for the field studies. The farming operations employed during the experiment were similar to the operations routinely employed on rural farms and did not involve endangered or protected species. All operations were approved by the CRES, China Agricultural University.

### Sampling and laboratory procedures

Prior to the experiments, five chemical soil properties were examined. Values were determined based on soil samples from a combined soil sample of the 10–20 cores from depths of 0–20 cm. Soil samples collected before planting were air-dried and sieved through a 0.2-mm mesh. Soil samples were used to measure organic matter content (OM) [18], alkaline hydrolyzable N (AN) [19], Olsen-P [20], NH_4_OAc-K [21], and pH [22]. Upon harvest, approximately 2.5×8-m^2^ sections of each plot were assessed, and ears were harvested from all plants by hand. The grain yield was adjusted to a moisture content of 15.5%.

### A regional N management approach

A guideline for regional N rate was calculated for each subregion through several steps. First, yield data were collected from a large number of N response trials (*n* = 1,726). Grain yield responses to N fertilizer curves were fit using a quadratic model with PROC NLIN (SAS Institute Inc., Cary, NC, USA) to generate yield function equations (the yield significantly (P<0.05) responded to N) [23], [24]. Next, from the response curve equation at each experimental site, the yield increase (above the yield in the N0 treatment), gross Chinese yuan return at that yield increase (maize grain price times yield), N fertilizer cost (N fertilizer price times N fertilizer rate), and net return to N ratio (gross yuan return minus N fertilizer cost) were calculated for each 1 kg N fertilizer rate increment from 0 to 270 kg N ha^−1^. Finally, for each incremental N rate, the net return was averaged across all trials in the subregional data set to generate an estimated ratio of the maximum return to N rate, and the corresponding yield across all trials at an N fertilizer:maize grain price ratio [14], [25]. In recent years, the fertilizer:maize grain price ratio has remained relatively stable, and a value of 2.05 was used in this study.

### Field-specific N management

In total, grain yield responses to N fertilizer curves were fit for 1,726 on-farm sites, using a quadratic model with PROC NLIN (SAS Institute Inc.) to generate yield function equations (the yield significantly (P<0.05) responded to N) [23], [24]. The minimum N rate for the maximum net return was calculated from the selected model based on an N:maize price ratio of 2.05.

### Nitrogen use efficiency and N balance

Nitrogen use efficiency for each treatment using the partial factor productivity (PFP_N_) indices. 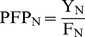
(1)


Where Y_N_  =  Crop yield with N applied;

F_N_  =  Amount of N applied.

Soil surface N balance was calculated as described in the Organization for Economic Co-operation and Development (OECD) [26].
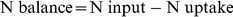
(2)where N input is N applied as chemical fertilizer, and N uptake is N in the harvested yield.



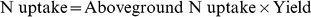
(3)The maize aboveground N uptake requirement per million grams (Mg) grain yield in China was determined previously; spring maize grain yield was <7.5 Mg ha^−1^, 7.5–9.0 Mg ha^−1^, 9.0–10.5 Mg ha^−1^, and 10.5–12.0 Mg ha^−1^, and N uptake requirements per Mg grain yield were 19.8, 18.1, 17.4 and 17.1 kg, respectively [27]. Summer maize N uptake requirements per Mg grain yield were 20 kg [28].

### Estimation of GHG emissions and emission intensity

Total GHG emissions during the entire life cycle of maize production, including CO_2_, CH_4_, and N_2_O, consisted of three components: (1) emissions during N fertilizer application, production and transportation, (2) emissions during P and K fertilizer production and transportation, and (3) emissions from pesticide and herbicide production (delivered to the gate) and diesel fuel consumption during sowing, harvesting, and tillaging operations [29].
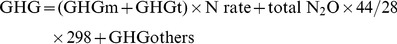
(4)where GHG (kg CO_2_ eq ha^−1^) is the total GHG emission, and GHGm is the GHG emission originating from fossil fuel mining as the industry's energy source to N product manufacturing, and was 8.21 kg CO_2_ eq kg N^−1^ (Table S3) [30]. GHGt is the N fertilizer transportation emission factor, and was 0.09 kg CO_2_ eq kg N^−1^ (Table S3) [30]. N rate is the N fertilizer application rate (kg N ha^−1^). GHG_others_ represents GHG emission of P and K fertilizer production and transportation, pesticide and herbicide production and transportation, and diesel fuel consumption (Table S3).

Total N_2_O emission included direct and indirect N_2_O emissions. Indirect N_2_O emissions were estimated with a method used by the International Panel on Climate Change [31], where 1% and 0.75% of ammonia (NH_3_) volatilization and nitrate (NO_3_^−^) leaching, respectively, is lost as N_2_O. N_2_O emission is calculated based on empirical models. Based on previous reports, the final data set consisted of 10 (30 observations) and 22 (117 observations) studies on direct N_2_O emissions for spring maize and summer maize, respectively. Detailed information is provided in Table S4 and Figure S1. 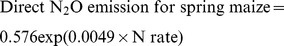
(5)

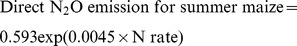
(6)


**Figure 2:**
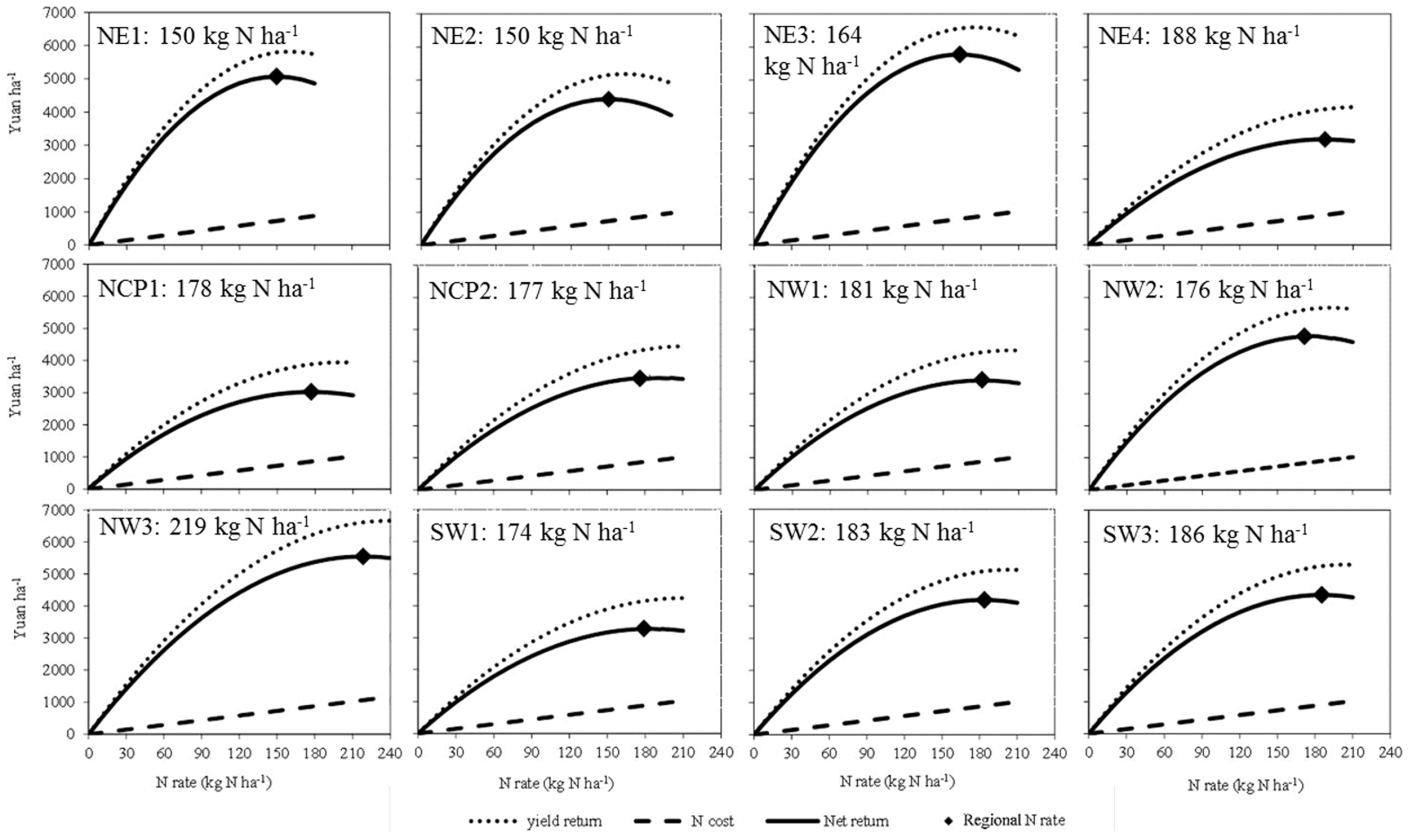
Maize grain yield and fertilizer economic components of calculated net return across N rates using the regional N management approach indicated at the 2.05 price ratio (N price 4.87 yuan kg^−1^ and maize price 2.37 yuan ha^−1^) in the 12 agroecological subregions. In total, 1,726 N responses trials were used to estimate the regional N rate. The net return is the increase in yield times the grain price at a particular N rate, minus the cost of that amount of N fertilizer. The maximum return is the N rate at which the net return is greatest.



NH_3_ volatilization and N leaching employs the following equation (Cui *et al* 2013, Global Change Biology, main text, Fig. 2) [6].

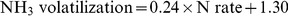
(7)

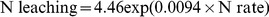
(8)


The system boundaries were set as the periods of the life cycle from the production inputs (such as fertilizers, pesticides, and herbicides), delivery of the inputs to the farm gates, and farming operations. We calculated total GHG emissions expressed as kg CO_2_ eq ha^−1^ and the GHG emission intensity expressed as kg CO_2_ eq Mg^−1^ grain. The change in soil organic carbon content was also not included in our analysis, because it was difficult to detect the small magnitude of the changes that occurred over a short time [32]. The soil CO_2_ flux as a contributor to global warming potential (GWP) was also not included in this study because the net flux was estimated to contribute less than 1% to the GWP of agriculture on a global scale [33].

To calculate total GHG emissions and emission intensity, the N rate and corresponding yield of each farm were used for farmers' N practices. The regional N rate and corresponding yield of each subregion were used for the regional N management approach, and the optimal N rate and corresponding yield of each field were used for field-specific N management.

## Results

### Farmers' Practice

Across all 5,406 farms, maize grain yield averaged 7.56 Mg ha^−1^, the corresponding N application rate averaged 220 kg ha^−1^, and the N balance averaged was 69 kg N ha^−1^ (Table S5). Calculated GHG emission intensity averaged 482 kg CO_2_ eq Mg^−1^ grain (Table S5), including the contributions of 155, 242, and 85 kg CO_2_ eq Mg^−1^ grain from N fertilizer use, N fertilizer production, and other sources, respectively (data not shown).

Large variations were observed in grain yield and N fertilizer application rates across the four main agroecological regions. The N application rates followed the order SW (250 kg N ha^−1^) ≈ NW (238 kg N ha^−1^) > NCP (208 kg N ha^−1^) ≈ NE (195 kg N ha^−1^). In contrast, the maize grain yields were highest in NE (8.91 Mg ha^−1^) followed by NW (7.58 Mg ha^−1^), NCP (7.42 Mg ha^−1^) and SW (5.45 Mg ha^−1^). The GHG emission intensity averaged 347, 436, 487, and 710 kg CO_2_ eq Mg^−1^ grain for NE, NCP, NW, and SW, respectively (Table 1).

### Regional N management approach

Across all 1,726 on-farm experiments, the average grain yield under the N0 treatment, weighted by maize area in each subregion, was 6.60 Mg ha^−1^ and ranged from 5.59 Mg ha^−1^ (SW2) to 8.12 Mg ha^−1^ (NW2) (Table 2). The average medium N rate (MN) recommended by local extension employees, weighted by maize area in each subregion, was 187 kg N ha^−1^ and ranged from 147 kg N ha^−1^ (NE2) to 221 kg N ha^−1^ (NW3). The corresponding grain yield under MN treatment averaged 8.69 Mg ha^−1^ and ranged from 7.63 Mg ha^−1^ (SW1) to 10.53 Mg ha^−1^ (NW2) (Table 2).

Considering all on-farm experiments, the calculated regional N rate based on the cost response to N application rate for the subregions, weighted by maize area in each subregion, averaged 174 kg N ha^−1^ and ranged from 150 kg N ha^−1^ (NE1 & NE2) to 219 kg N ha^−1^ (NW3) (Table 2, Fig 2). The corresponding grain yield averaged 8.56 Mg ha^−1^ and ranged from 7.46 Mg ha^−1^ (SW1) to 10.38 Mg ha^−1^ (NW2) (Table 2). Calculated GHG emission intensity, weighted by maize area in each subregion, averaged 334 kg CO_2_ eq Mg^−1^ grain and ranged from 272 kg CO_2_ eq Mg^−1^ grain (NE2) to 387 kg CO_2_ eq Mg^−1^ grain (SW2).

Based on the maize grain yield response to N application rates in all 1,726 on-farm experiments, the calculated field-specific N rate, weighted by maize area in each subregion, averaged 178 kg N ha^−1^ (Table 2) and ranged from 53 kg N ha^−1^ to 271 kg N ha^−1^ (Table S2), with a coefficient of variation (CV) of 18% (data not shown). The corresponding grain yield averaged 8.63 Mg ha^−1^ (Table 2) and ranged from 4.29 Mg ha^−1^ to 14.91 Mg ha^−1^ (Table S2), with a CV of 19% (data not shown). The calculated GHG emission intensity averaged 343 kg CO_2_ eq Mg^−1^ grain (Table 2). The similar N rate, grain yield and GHG emission intensity between the regional N management approach and field-specific N management supported the notion that the regional N rate was close to an economic and environmentally optimal N application (Table 2).

### Opportunities to reduce the GHG emission intensity

Compared to farmer's practices, the regional N management approach proposed reducing N fertilizer by 20.9% (220 vs. 174 kg N ha^−1^). The grain yield would increase by 13.2% (7.56 vs. 8.56 Mg ha^−1^). The GHG emission intensity would decrease by 30.7%, from 482 to 334 kg CO_2_ eq Mg^−1^ grain. The overuse and high variability of N use by farmers has resulted in a high variability in GHG emission intensity, ranging from 364 to 1,399 kg CO_2_ eq Mg^−1^ grain (Table S5) with a CV of 43% (data not shown).

**Figure 3:**
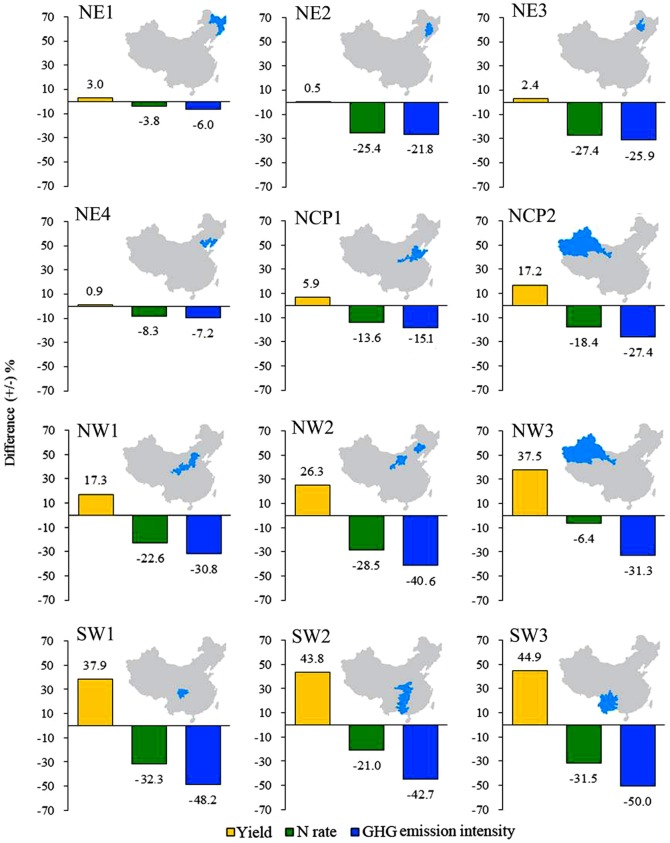
Regional differences (±%) in N application rates, grain yield, and GHG emission intensity between the regional N management approach and farmers' practice in the 12 agroecological subregions. Regional difference (±%)  =  (regional approach minus farmers' practice)/farmers' practice ×100.

Of the 12 agroecological subregions, NE2, NE3, NW1, NW2, SW1, SW2, and SW3 showed the highest potential for N-reduction (>20%), ranging from 21.0% to 31.5% and accounting for 55% of the total maize-sown area. Reduced N rates in other subregions ranged from 3.8% to 18.4% and accounted for 45% of the total maize-sown area. The subregions with a high yield increase potential (>15%; Fig. 3) were NCP2, NW1, NW2, NW3, SW1, SW2, and SW3, with increases ranging from 17.2% to 44.9% and accounting for 44% of the total maize-sown area. Grain yield in other regions ranged from 0.5% to 5.9%, accounting for 56% of the total maize-sown area. Subregions with a high potential to decrease GHG emission intensity (>20%) included NE2, NE3, NCP2, NW1, NW2, NW3, SW1, SW2, and SW3, ranging from 21.8% to 50.0% and accounting for 64% of the total maize-sown area. Reduced GHG emission intensity in other regions ranged from 6.0% to 15.1%, accounting for 36% of the total maize-sown area.

This regional N management approach, if widely adopted in China, regional N fertilizer consumption would be reduced by 1.4 MT (−20.3%), and 91% of this reduction would occur in the NE2, NE3, NCP1, NW1, NW2, SW1, SW2, and SW3 subregions (Table 3). At the same time, Chinese maize production could be increased by 31.9 MT (13.1%), from 244.1 MT to 276.0 MT, when undertaking this regional N management approach (Table 3). Total GHG emissions would be reduced by 18.6 MT eq CO_2_ year^−1^ (−16.9%) (from 110.2 to 91.5 MT eq CO_2_ year^−1^) (Table 3), with 91% of this reduction occurring in the NE2, NE3, NCP1, NW1, NW2, SW1, SW2, and SW3 subregions.

**Table 3:** Maize production, N fertilizer consumption and total GHG emission between the regional N rate and farmers' practice in 12 agro-ecological subregions.

Subregion	Area (million ha)	N fertilizer consumption (MT)			Maize production (MT)			Total GHG emission (MT eq CO_2_ yr^−1^)		
		Farmers' practice	Regional N rate	Difference a	Farmers' practice	Regional N rate	Difference a	Farmers' practice	Regional N rate	Difference a
NE1	1.45	0.23	0.22	−0.01	12.5	12.8	0.4	3.7	3.6	−0.1
NE2	4.80	0.96	0.72	−0.24	43.8	44.1	0.2	15.2	12.0	−3.3
NE3	1.50	0.34	0.25	−0.09	13.2	13.5	0.3	5.3	4.0	−1.3
NE4	2.06	0.42	0.39	−0.04	17.9	18.0	0.2	6.7	6.2	−0.4
NCP1	8.24	1.70	1.47	−0.23	63.3	67.0	3.7	26.4	23.8	−2.7
NCP2	1.94	0.42	0.34	−0.08	13.9	16.2	2.4	6.5	5.6	−1.0
NW1	3.35	0.78	0.61	−0.18	23.2	27.2	4.0	12.1	9.8	−2.3
NW2	2.36	0.58	0.42	−0.17	19.4	24.5	5.1	9.0	6.8	−2.2
NW3	0.85	0.20	0.19	−0.01	6.1	8.4	2.3	3.1	2.9	−0.2
SW1	1.14	0.29	0.20	−0.09	6.2	8.5	2.3	4.5	3.2	−1.3
SW2	2.54	0.59	0.46	−0.12	13.6	19.6	6.0	9.2	7.6	−1.6
SW3	1.99	0.54	0.37	−0.17	11.1	16.1	5.0	8.4	6.1	−2.3
National b	32.23	7.06	5.62	−1.43	244.1	276.0	31.9	110.2	91.5	−18.6

a Different mean the different of maize production, N fertilizer consumption, and total GHG emission between regional N rate and farmer's practice.

b National values are computed from the regional values weighted by area. The regional weights are as follows:

NE1, 4.5%; NE2, 14.9%; NE3, 4.7%; NE4, 6.4%; NCP1, 25.6%; NCP2, 6.0%; NW1, 10.4%; NW2, 7.3%; NW3, 2.6%; SW1, 3.5%; SW2, 7.9%; SW3, 6.2.

## Discussion

The current intensive maize system used in farmers' practices in China results in a median yield, high N application, and GHG emission intensity of 7.56 Mg ha^−1^, 220 kg N ha^−1^, and 482 kg CO_2_ eq Mg^−1^ grain, respectively. These yields and N application rates are higher than the reported global averages (4.81 Mg ha^−1^ and 104.9 kg N ha^−1^, the N rate calculated based on maize N fertilizer consumption and maize area harvested) for these crops in 2006 [8], [15], [34] and are similar to the previously reported Chinese averages for maize [35], [36]. In comparison, grain yield in central Nebraska, USA, averaged 13.2 Mg ha^−1^ with only 183 kg N ha^−1^. GHG emission intensity in this region was only 231 kg CO_2_ eq Mg^−1^ grain, which was 48% lower than the average for China [37] and 109% lower than the 482 kg CO_2_ eq Mg^−1^ grain for individual farmer's practices in China. The median yield and large GHG emission intensity for Chinese maize systems were attributable to the large variation in N application rates among fields. Considering 5,406 farms, N application rates ranged from 46 (only 56% of crop N uptake) to 615 kg N ha^−1^ (414% of crop N uptake). Similar results were reported by Wang *et al* (2007), showing that one-third of farmers apply too little N, while another one-third of farmers apply too much (*n* = 10,000) [38].

In small-scale farming, a lack of basic knowledge and information on crop responses to N fertilizer often results in the over- and underapplication of N fertilizer [39], [40]. We developed and assessed regional N management approach using large pools of response trial data that have been grouped according to criteria that indicate differing N responses for regions with similar management, climates, and soil. Our guide provides a N application rate that can be used to reduce the potential for N-deficiency or N-surplus, lowers the likelihood of reduced yields and profits, and lessens GHG emissions intensity (particularly N_2_O emissions associated with N fertilization). Using a regional N management approach, potential for crop productivity increases and the mitigation of GHG emission intensity are likely to be achieved through a combination of increased N application in regions with a low N input and improved PFP_N_ in regions where N fertilizer application is already high. Meanwhile, crop N uptake and N use efficiency can improve the ratio split application, with one-third for base dressing and two-thirds for top dressing [36]. Currently, typical farmers' practices apply 50% of the total N fertilizer before planting or at the early growth stage [36], [41]. Some recent practices have indicated that the amount of basal application should be added to the ratio of the top dressing to improve N use efficiency and increase grain yield [36].

The gains in yield and reduced GHG emissions achieved using regional N management approach are significant. Moreover, we believe these benefits can be further improved by applying other best-management strategies to fertilizer (e.g., slow-release N fertilizer, N transformation inhibitors, and fertigation) [42] and related practices that enhance the crop recovery of applied N (e.g., rotation with N fixing crops, precision agriculture management techniques) [42]. While this approach for N fertilizer management should be extended to farmers throughout the entire Chinese cereal production area, it is also relevant to other high-yield cropping systems outside of China. The economic approach to N rate recommendations based on multiple N rate trials has been applied for two to three decades in the U.S. Midwest, and has been more recently “formalized” with the Iowa State MRTN approach for seven Midwestern states [43].

This regional N management approach, if widely adopted in China, could reduce fertilizer N consumption by 20.3%, increase Chinese maize production by 13.1%, and reduce total GHG emissions by 16.9%. Moreover, the recommendations provide reasonable N rates and high net return, and can be easily adopted in rural areas of China where no available soil and/or plant N monitoring facilities exist [44]. The regional N rate can also be used as a reference point for agricultural extension employees without any soil and/or plant N monitoring. In practice, some factors also affect these suggested regional N rates, such as timing of crop rotation, tillage system, and soil productivity [14]. For example, the recommended N rate for soybean following maize rotations is lower than maize following maize rotations [14]. No-till management can delay or reduce residue breakdown, or mineralization, thereby reducing the N supplied from crop residue [14]. Soils where productivity is limited frequently require higher rates of fertilizer N to reach optimum yield. Conversely, lower rates of fertilizer N may be needed to reach optimum yield on highly productive soils [14].

Although this regional N management approach can easily be adopted in rural areas, delivering this technology to millions of farmers is challenging due to the lack of effective advisory systems and knowledgeable farmers. For example, educated young male farmers tend to leave the farming sector for more profitable jobs, leaving farmwork to the older and less educated individuals, especially in low income or remote areas [45]. In addition, adding more N fertilizer based on the regional N rate is difficult for farmers with low incomes or in remote areas. The Chinese central government has been aware of this problem and has attempted to provide agricultural technologies to these areas. For example, China has launched national programs for soil testing and fertilizer recommendations since 2005. In 2009, 2,500 counties in China were involved in the programs, receiving a total of 1.5 billion yuan from the Chinese central government [40].

Although the on-farm trials were conducted by local farmers in the same counties as the farmers' surveys (including experimental counties), the management and environment is not always the same for on-farm trials and farmers' surveys. While gains in grain yield and GHG were achieved by farmers using the trials, we believe that the majority of these gains can be realized in practice in many counties if improved agronomic and N management techniques are adopted. The management and environment differed among four maize regions; thus, N losses may also differ. For example, the annual direct N_2_O emission accounted for 0.92% of the applied N with an uncertainty of 29%. The highest N_2_O fluxes occurred in East China as compared with the lowest fluxes in West China [46]. In this study, we use the different exponential relationships of the N application rate and N_2_O fluxes for spring maize and summer maize, respectively. However, developing N loss models at the regional or subregional scale is difficult due to insufficient field measurement data in China. Long-term field observations covering all subregions are required to accurately assess farming potential and mitigate GHG emissions.

## Supporting Information

Figure S1 **Relationships between the N application rate and direct N_2_O emissions for spring maize (A) and summer maize (B) production in China based on a meta-analysis.** The direct N_2_O emission data was taken from Table S4.
(TIF)

Table S1 **The criteria and values for the sub-regional divisions.**
(DOCX)

Table S2 **The site, year, soil type, irrigation, crop rotations, soil organic matter (SOM) content, alkaline hydrolyzable N (AN), Olsen-P (AP), NH_4_OAc-K (AK), pH, medium N rate (MN), recommended P_2_O_5_ rate (RP), recommended K_2_O rate (RK), grain yield without N fertilizer, yield at 50% MN, yield at 100% MN, yield at 150% MN, economic optimal N rate (EONR), yield at EONR, and GHG emissions intensity at EONR for all 1,726 on-farm experiments.**
(DOCX)

Table S3 **GHG emission factors of agricultural inputs.**
(DOCX)

Table S4 **The site, year, annual mean precipitation, temperature, soil organic matter (SOM), total N content, pH, N rate, grain yield, and direct N_2_O emissions at different experimental sites.**
(DOCX)

Table S5 Descriptive statistics of the surveyed farms N fertilizer application rate, maize grain yield, PFP_N_, N balance and GHG emission intensity for 5,406 farmed fields between 2007 and 2009 in China.
(DOCX)

Text S1 **Detailed information for each of these regions.**
(DOCX)
