# Supplementary figures and images for: Establishing a Regional Nitrogen Management Approach to Mitigate Greenhouse Gas Emission Intensity from Intensive Smallholder Maize Production
Source: PLoS One. 2014 May 29;9(5):e98481. doi: 10.1371/journal.pone.0098481 (PMC4038602; doi:10.1371/journal.pone.0098481)

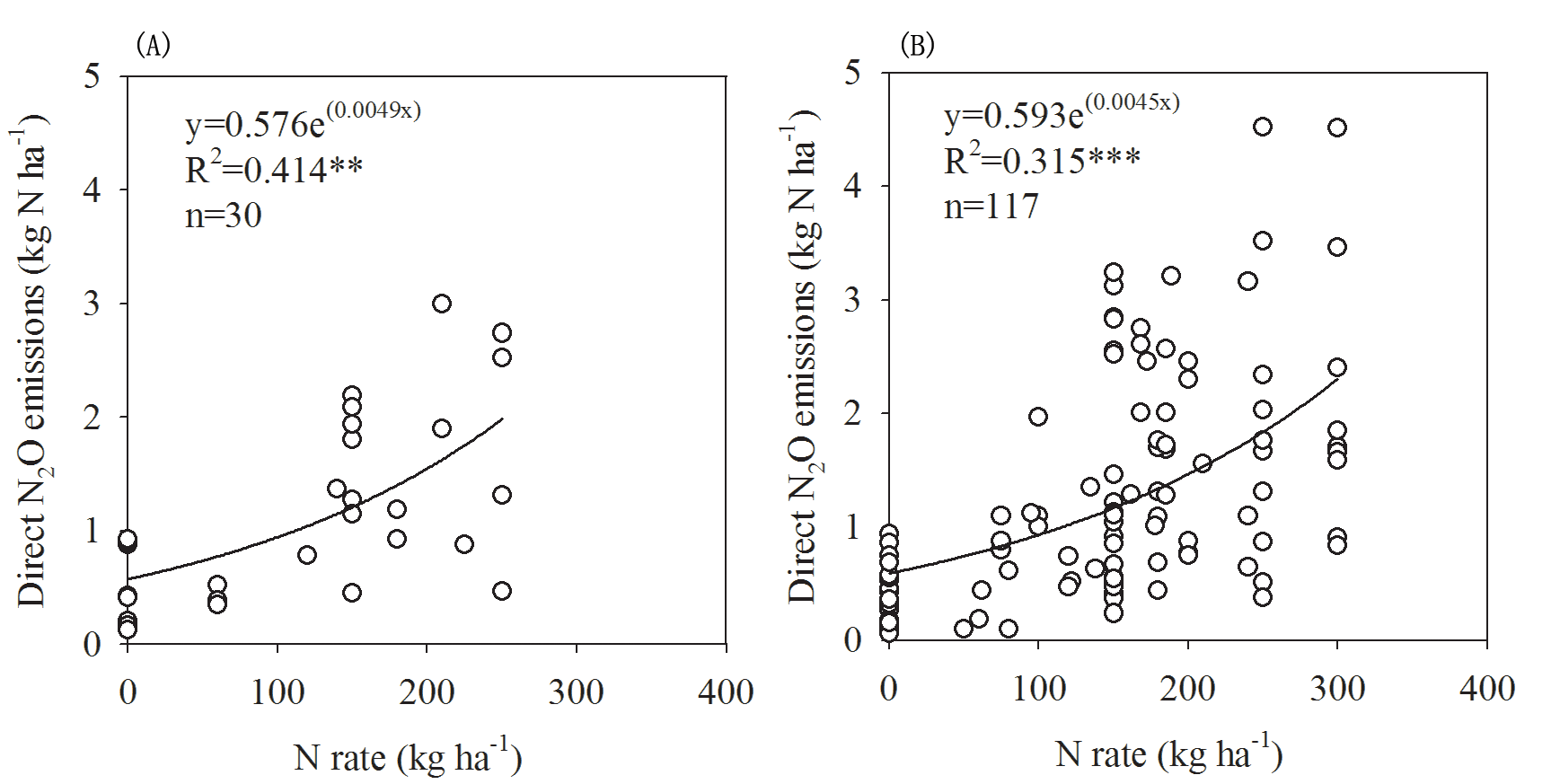

Supplement: Figure S1 — Relationships between the N application rate and direct N2O emissions for spring maize (A) and summer maize (B) production in China based on a meta-analysis. The direct N2O emission data was taken from Table S4. (TIF) [file pone.0098481.s001.tif]
