# Supplementary material for: Establishing a Regional Nitrogen Management Approach to Mitigate Greenhouse Gas Emission Intensity from Intensive Smallholder Maize Production
Source: PLoS One. 2014 May 29;9(5):e98481. doi: 10.1371/journal.pone.0098481 (PMC4038602; doi:10.1371/journal.pone.0098481)
Supplement: Table S1 — The criteria and values for the sub-regional divisions. (DOCX) [file pone.0098481.s002.docx]

**Table S1** The criteria and values for the sub-regional divisions.

| Region | Subregion | Maize cultivated  area  (million ha) ^a^ | Cropping system ^a^ | Cumulative temperature above 10 degree C in maize grown season | Rainfall  (mm) | Irrigation ^b^ | Terrain ^c^ | Soil type ^d^ | Main limiting factor ^e^ |
| --- | --- | --- | --- | --- | --- | --- | --- | --- | --- |
| NE | NE1 | 1.45 | MM | <2900 | >450 | No | M, P | DBE, BS, MS | Tem., Lig., frost |
|  | NE2 | 4.8 | MM | >2900 | >450 | No | P | BS, Che., MS | PDIP |
|  | NE3 | 1.5 | MM | >2900 | <450 | Yes | P | ASS, DC, Che. | Drou., PDIP |
|  | NE4 | 2.06 | MM | >2900 | >450 | No | P | BE, CS, MS | PDIP |
| NCP | NCP1 | 8.24 | MW | <1900 | 426-750 | Partly | P | CS, FAS, CCS, BE | IWS, HT, PDIP, RIM |
|  | NCP2 | 1.94 | MW | >1900 | 600-900 | No | P | FAS, LCFA | PDIP, HT |
| NW | NW1 | 3.35 | MM | 2140-4000 | 400-550 | No | P, B, H, M | CS, BE, CLS | Drou., HT |
|  | NW2 | 2.36 | MM | 1584-3300 | 125-500 | Yes | P, H, RV | IWS, CS | Drou., HT |
|  | NW3 | 0.85 | MM | 2000-5300 | <200 | Yes | D, G | BS, AMS | Drou., HT |
| SW | SW1 | 1.14 | MW | 4500-6000 | 900-1200 | No | B | PS | PDIP |
|  | SW2 | 2.55 | MW, MM, WMI | 3863-7938 | 800-1700 | No | H, M | YE, YBE, RE, LRE | PDIP, SF |
|  | SW3 | 1.99 | MM, WMI | 3000-8200 | 600-1700 | No | P, B, RV | YE, RE, LRE | SF, SD, RIM |

^a^ China Statistics Yearbook, 2012

^b^ Cropping system: MM = continuous maize; MW = maize-wheat rotation; WMI = winter wheat-maize interplant.

^c^ Irrigation: Yes = Irrigation; No = Rainfed; Partly = Irrigation & rainfed.

^d^ Terrain: M = Mountain, P = Plain, H = Hill, B = Basin, P = Plateau, RV = River valley, D = Desert, G = Gobi

^e^ Soil type: BS = Black soils, Che. = Chernozems, ASS = Aeolian sandy soils, MS = Meadow soils, DBE = Dark brown earths, DC = Dark castanozems, CS = Clinnamon soils, BE = Brown earths, FAS = Fluvo-aquic soils, LCFA = Lime concretion fluvo-aquic soils, YBE = Yellow-brown earths, CCS = Cumulic clinnamon soils, CLS = Cultivated loessial soils, BS = Bog soils, AMS = Alpine meadow soils, IWS = Irrigating warped soils, RE = Red earths, LRE = Lateritic red earths, YE = Yellow earths, PS = Purplish soils.

^f^ Main limiting factor: Tem. = Temperature, Lig. = Light, Drou. = Drought, PDIP = Plant diseases and insect pests, IWS = Irrigation water shortage, HT = High temperature, RIM = Rainfall imbalance, SF = Soil fertility, SD = Spring drought.
