# Supplementary material for: Establishing a Regional Nitrogen Management Approach to Mitigate Greenhouse Gas Emission Intensity from Intensive Smallholder Maize Production
Source: PLoS One. 2014 May 29;9(5):e98481. doi: 10.1371/journal.pone.0098481 (PMC4038602; doi:10.1371/journal.pone.0098481)
Supplement: Table S2 — The site, year, soil type, irrigation, crop rotations, soil organic matter (SOM) content, alkaline hydrolyzable N (AN), Olsen-P (AP), NH4OAc-K (AK), pH, medium N rate (MN), recommended P2O5 rate (RP), recommended K2O rate (RK), grain yield without N fertilizer, yield at 50% MN, yield at 100% MN, yield at 150% MN, economic optimal N rate (EONR), yield at EONR, and GHG emissions intensity at EONR for all 1,726 on-farm experiments. (DOCX) [file pone.0098481.s003.docx]

**Table S2** The site, year, soil type, irrigation, crop rotations, soil organic matter (SOM) content, alkaline hydrolyzable N (AN), Olsen-P (AP), NH_4_OAc-K (AK), pH, medium N rate (MN), recommended P_2_O_5_ rate (RP), recommended K_2_O rate (RK), grain yield without N fertilizer, yield at 50% MN, yield at 100% MN, yield at 150% MN, economic optimal N rate (EONR), yield at EONR, and GHG emissions intensity at EONR for all 1,726 on-farm experiments.

| Site | Coordinate | year | Soil type ^a^ | Irrigation  ^b^ | | | Crop rotations ^c^ | SOM  (g kg^-1^) | AN  (mg kg^-1^) | AP  (mg kg^-1^) | AK  (mg kg^-1^) | pH | RN  (kg N ha^-1^) | RP (kg P_2_O_5_ ha^-1^) | RK  (kg K_2_O ha^-1^) | Yield without N (Mg ha^-1^) | Yield at 50% RNR (Mg ha^-1^) | yield at 100% RNR (Mg ha^-1^) | yield at 150% RNR (Mg ha^-1^) | EONR  (kg N ha^-1^) | EONR yield  (Mg ha^-1^) | GHG intensity  (kg CO_2_ eq Mg^-1^ grain) |
| --- | --- | --- | --- | --- | --- | --- | --- | --- | --- | --- | --- | --- | --- | --- | --- | --- | --- | --- | --- | --- | --- | --- |
| **NE1 subregions** |  |  |  |  | | |  |  |  |  |  |  |  |  |  |  |  |  |  |  |  |  |
| 853 Farm,  Heilongjiang | 46.3°N,  133.0°E | 2008 | MS | | R | | MM | 19.9 | 115.0 | 17.5 | 287.0 | 6.3 | 169 | 90 | 36 | 6.44 | 7.25 | 8.59 | 8.08 | 160 | 8.42 | 304 |
|  |  |  | DBE | | R | | MM | 46.3 | 244.0 | 17.4 | 94.0 | 6.2 | 169 | 90 | 36 | 7.43 | 7.84 | 8.52 | 8.00 | 169 | 8.62 | 310 |
|  |  |  | BS | | R | | MS | 34.6 | 127.0 | 21.8 | 190.0 | 6.4 | 169 | 90 | 36 | 6.48 | 7.72 | 8.61 | 7.99 | 147 | 9.08 | 263 |
|  |  |  | MS | | R | | MM | 38.6 | 74.0 | 18.8 | 107.0 | 5.9 | 169 | 90 | 36 | 6.33 | 7.58 | 8.98 | 8.16 | 154 | 9.37 | 264 |
|  |  |  | DBE | | R | | MM | 37.5 | 189.0 | 23.9 | 204.0 | 6.3 | 169 | 90 | 36 | 7.85 | 8.49 | 9.39 | 8.67 | 178 | 9.45 | 296 |
|  |  |  | BS | | R | | MM | 28.4 | 93.0 | 21.5 | 74.0 | 6.5 | 169 | 90 | 36 | 6.88 | 8.64 | 9.52 | 8.60 | 173 | 9.17 | 298 |
|  |  | 2009 | MS | | R | | MM | 19.4 | 80.0 | 16.3 | 143.0 | 6.4 | 169 | 90 | 36 | 6.65 | 8.63 | 9.38 | 9.18 | 156 | 7.87 | 317 |
|  |  |  | DBE | | R | | MM | 34.5 | 197.0 | 15.8 | 83.0 | 6.2 | 169 | 90 | 36 | 7.76 | 8.58 | 9.28 | 9.06 | 155 | 8.06 | 308 |
|  | 46.3°N,  133.1°E | 2009 | BS | | R | | MM | 32.3 | 84.0 | 15.9 | 112.0 | 6.3 | 169 | 90 | 36 | 5.75 | 7.21 | 8.00 | 7.30 | 144 | 8.17 | 288 |
|  |  |  | DBE | | R | | MM | 24.9 | 153.0 | 15.3 | 215.0 | 5.8 | 169 | 90 | 36 | 6.47 | 7.59 | 8.13 | 7.72 | 174 | 7.76 | 354 |
|  |  |  | DBE | | R | | MM | 38.9 | 216.0 | 19.7 | 243.0 | 6.1 | 169 | 90 | 36 | 7.05 | 7.82 | 8.27 | 7.88 | 200 | 9.27 | 336 |
|  |  |  | MS | | R | | MM | 37.5 | 119.0 | 22.1 | 170.0 | 5.8 | 169 | 90 | 36 | 6.14 | 6.70 | 8.24 | 7.46 | 140 | 9.01 | 255 |
|  |  | 2010 | MS | | R | | MS | 33.4 | 173.0 | 22.1 | 85.0 | 5.7 | 169 | 90 | 36 | 7.69 | 8.36 | 9.43 | 9.20 | 174 | 8.77 | 313 |
|  |  |  | BS | | R | | MM | 26.6 | 142.0 | 20.1 | 65.0 | 5.9 | 169 | 90 | 36 | 7.38 | 8.32 | 9.36 | 8.17 | 183 | 5.93 | 484 |
|  | 46.2°N,  133.0°E | 2008 | BS | | R | | MM | 25.7 | 140.0 | 20.8 | 135.0 | 6.2 | 169 | 90 | 36 | 6.70 | 8.08 | 8.80 | 8.55 | 189 | 10.71 | 276 |
|  |  |  | BS | | R | | MM | 17.2 | 155.0 | 19.6 | 106.4 | 5.8 | 152 | 83 | 40 | 4.80 | 5.07 | 6.18 | 5.85 | 163 | 9.39 | 277 |
|  |  | 2009 | MS | | R | | MM | 25.0 | 112.0 | 23.0 | 102.0 | 5.8 | 152 | 83 | 40 | 8.11 | 9.32 | 10.87 | 10.58 | 178 | 9.25 | 303 |
|  |  |  | MS | | R | | MM | 25.6 | 137.0 | 20.0 | 80.0 | 6.3 | 152 | 83 | 40 | 7.66 | 8.79 | 9.38 | 9.29 | 147 | 5.92 | 403 |
|  |  | 2010 | MS | | R | | MM | 22.7 | 117.7 | 21.6 | 161.1 | 5.8 | 161 | 92 | 50 | 6.47 | 9.18 | 9.65 | 9.20 | 156 | 9.84 | 254 |
|  |  |  | MS | | R | | MM | 24.5 | 165.2 | 15.0 | 128.7 | 5.2 | 161 | 92 | 50 | 5.36 | 7.20 | 9.54 | 6.82 | 138 | 8.75 | 259 |
| 855 Farm,  Heilongjiang | 45.4°N,  131.4°E | 2007 | AS | | R | | MM | 31.8 | 174.3 | 18.4 | 127.3 | 5.9 | 126 | 81 | 41 | 6.96 | 10.13 | 9.63 | 10.73 | 136 | 10.34 | 311 |
|  |  |  | AS | | R | | MS | 31.7 | 156.9 | 24.3 | 141.0 | 5.8 | 126 | 81 | 41 | 7.07 | 8.09 | 9.14 | 7.75 | 186 | 9.90 | 355 |
|  |  | 2008 | AS | | R | | MM | 23.5 | 129.0 | 23.7 | 110.0 | 5.5 | 138 | 69 | 41 | 7.21 | 8.46 | 8.91 | 8.00 | 146 | 9.91 | 274 |
|  |  |  | AS | | R | | MM | 45.2 | 221.0 | 19.5 | 139.0 | 5.5 | 138 | 69 | 41 | 7.95 | 9.36 | 9.43 | 9.79 | 183 | 10.88 | 304 |
|  |  | 2009 | AS | | R | | MM | 33.4 | 169.0 | 19.4 | 125.0 | 5.5 | 138 | 69 | 41 | 8.14 | 9.42 | 10.74 | 9.52 | 160 | 10.91 | 315 |
|  |  | 2010 | PS | | R | | MM | 29.2 | 145.0 | 22.6 | 136.0 | 5.8 | 138 | 76 | 41 | 6.00 | 9.47 | 8.77 | 10.16 | 210 | 10.81 | 276 |
|  | 45.4°N,  131.3°E | 2007 | AS | | R | | MM | 47.2 | 337.4 | 22.6 | 184.1 | 5.6 | 131 | 69 | 27 | 6.96 | 9.42 | 9.65 | 9.41 | 176 | 9.25 | 265 |
|  |  |  | Chern | | R | | MM | 36.1 | 224.4 | 21.5 | 158.2 | 5.5 | 131 | 69 | 27 | 6.12 | 9.50 | 10.34 | 10.85 | 140 | 8.73 | 281 |
|  |  | 2008 | BS | | R | | MM | 42.9 | 204.8 | 23.8 | 127.9 | 5.6 | 131 | 69 | 27 | 6.51 | 10.16 | 10.34 | 10.59 | 158 | 8.81 | 403 |
|  |  |  | MS | | R | | MM | 48.3 | 250.7 | 16.9 | 161.0 | 5.5 | 131 | 69 | 27 | 7.03 | 9.82 | 9.85 | 11.02 | 182 | 10.42 | 217 |
|  |  | 2009 | MS | | R | | MM | 53.6 | 265.6 | 20.1 | 129.4 | 5.8 | 131 | 69 | 27 | 7.92 | 8.54 | 9.34 | 9.22 | 189 | 9.69 | 294 |
|  |  |  | DBE | | R | | MM | 49.9 | 258.2 | 22.5 | 160.5 | 5.4 | 131 | 69 | 27 | 6.39 | 8.04 | 8.85 | 8.11 | 168 | 9.60 | 239 |
|  |  | 2010 | DBE | | R | | MM | 62.7 | 343.0 | 22.1 | 152.0 | 5.1 | 131 | 69 | 27 | 6.85 | 7.80 | 9.14 | 8.52 | 148 | 8.91 | 263 |
|  |  |  | DBE | | R | | MM | 45.8 | 229.0 | 23.6 | 125.0 | 5.6 | 131 | 69 | 27 | 7.10 | 9.21 | 10.24 | 10.35 | 123 | 9.68 | 235 |
| Chahayang Farm,  Heilongjiang | 48.1°N,  124.2°E | 2007 | Chern | | R | | MM | 30.9 | 171.8 | 24.8 | 349.8 | 6.9 | 119 | 69 | 12 | 6.06 | 7.56 | 8.26 | 8.44 | 160 | 7.56 | 301 |
|  |  |  | BS | | R | | MM | 20.3 | 137.8 | 21.9 | 223.6 | 6.8 | 119 | 69 | 12 | 5.14 | 6.53 | 6.88 | 7.33 | 184 | 7.11 | 300 |
|  |  |  | AS | | R | | MM | 35.4 | 189.5 | 23.5 | 249.8 | 6.9 | 119 | 69 | 12 | 6.06 | 7.15 | 8.68 | 8.44 | 160 | 7.71 | 263 |
|  |  | 2008 | Chern | | R | | MM | 46.0 | 167.9 | 29.4 | 164.7 | 6.8 | 119 | 69 | 12 | 4.79 | 6.85 | 7.80 | 3.14 | 185 | 7.46 | 288 |
|  |  | 2009 | Chern | | R | | MM | 31.0 | 293.0 | 20.6 | 224.0 | 7.8 | 119 | 69 | 12 | 4.81 | 6.84 | 7.41 | 7.34 | 166 | 7.71 | 274 |
|  |  |  | Chern | | R | | MM | 40.8 | 151.6 | 26.4 | 214.3 | 6.2 | 119 | 69 | 12 | 4.98 | 6.06 | 7.17 | 7.04 | 177 | 9.07 | 304 |
|  | 48.1°N,  124.1°E | 2009 | DBE | | R | | MM | 30.6 | 155.8 | 21.4 | 174.7 | 7.1 | 119 | 69 | 12 | 4.96 | 6.99 | 7.56 | 7.49 | 153 | 6.41 | 278 |
|  |  |  | BS | | R | | MM | 38.0 | 132.5 | 15.3 | 174.7 | 6.4 | 119 | 69 | 12 | 5.04 | 6.25 | 7.54 | 7.36 | 129 | 10.39 | 269 |
|  |  |  | MS | | R | | MM | 31.0 | 151.6 | 18.4 | 224.2 | 5.9 | 119 | 69 | 12 | 4.64 | 6.70 | 7.65 | 7.49 | 182 | 9.34 | 215 |
|  |  | 2010 | DBE | | R | | MS | 40.8 | 207.3 | 21.4 | 286.8 | 6.7 | 105 | 69 | 15 | 7.18 | 8.19 | 9.13 | 9.01 | 122 | 8.04 | 265 |
|  |  |  | BS | | R | | MM | 42.9 | 204.8 | 23.8 | 98.0 | 5.6 | 152 | 83 | 40 | 7.92 | 8.54 | 9.34 | 9.22 | 142 | 7.66 | 256 |
|  |  |  | DBE | | R | | MM | 48.9 | 214.6 | 23.4 | 115.1 | 5.8 | 161 | 92 | 50 | 5.21 | 5.58 | 6.05 | 5.90 | 142 | 7.75 | 262 |
| Daqing,  Heilongjiang | 46.3°N,  125.1°E | 2008 | DBE | | R | | MM | 37.0 | 128.0 | 16.8 | 90.0 | 7.9 | 105 | 75 | 90 | 5.39 | 7.90 | 8.40 | 7.96 | 147 | 8.55 | 263 |
| Fenghuangshan Farm,  Heilongjiang | 48.2°N,  126.2°E | 2007 | DBE | | R | | MM | 44.7 | 233.0 | 19.3 | 248.0 | 6.3 | 161 | 107 | 24 | 5.31 | 7.22 | 10.13 | 9.37 | 102 | 7.61 | 288 |
|  |  |  | BS | | R | | MM | 32.0 | 121.9 | 24.9 | 218.4 | 6.1 | 152 | 83 | 40 | 7.51 | 8.38 | 10.00 | 9.33 | 152 | 9.25 | 274 |
|  |  |  | BS | | R | | MM | 33.3 | 139.6 | 15.4 | 144.5 | 6.3 | 152 | 83 | 40 | 7.65 | 8.40 | 9.05 | 8.75 | 161 | 8.08 | 301 |
|  |  | 2008 | BS | | R | | MM | 39.0 | 154.0 | 24.5 | 130.0 | 6.0 | 152 | 83 | 40 | 7.46 | 9.11 | 9.81 | 8.36 | 159 | 10.70 | 338 |
|  |  |  | MS | | R | | MM | 47.8 | 160.0 | 20.1 | 146.0 | 5.9 | 137 | 69 | 30 | 6.95 | 7.51 | 7.63 | 7.18 | 187 | 9.53 | 405 |
|  |  |  | MS | | R | | MM | 63.5 | 80.0 | 21.2 | 279.0 | 6.0 | 137 | 69 | 30 | 6.02 | 8.38 | 9.21 | 8.74 | 182 | 9.52 | 331 |
|  |  | 2009 | MS | | R | | MM | 52.6 | 198.0 | 15.3 | 216.0 | 5.6 | 137 | 69 | 30 | 6.60 | 7.17 | 8.42 | 7.89 | 199 | 10.30 | 388 |
|  |  |  | BS | | R | | MM | 63.9 | 152.0 | 16.7 | 148.0 | 5.6 | 137 | 69 | 30 | 6.24 | 9.38 | 10.67 | 10.13 | 97 | 8.70 | 342 |
|  |  |  | BS | | R | | MM | 26.7 | 114.0 | 19.9 | 174.0 | 5.2 | 137 | 69 | 30 | 7.13 | 8.07 | 9.83 | 9.37 | 120 | 9.94 | 306 |
|  |  | 2010 | BS | | R | | MM | 32.1 | 158.0 | 19.7 | 267.0 | 5.2 | 137 | 69 | 30 | 7.56 | 8.14 | 9.94 | 9.33 | 167 | 10.84 | 385 |
|  |  |  | BS | | R | | MS | 39.7 | 213.0 | 16.1 | 124.0 | 6.7 | 137 | 69 | 30 | 6.43 | 8.04 | 10.56 | 10.09 | 140 | 9.99 | 208 |
|  | 48.2°N,  126.1°E | 2008 | MS | | R | | MM | 44.6 | 174.0 | 16.6 | 165.2 | 5.9 | 137 | 69 | 30 | 6.48 | 8.67 | 8.10 | 5.60 | 128 | 9.59 | 305 |
|  |  |  | GS | | R | | MM | 39.6 | 154.0 | 19.4 | 159.2 | 6.1 | 137 | 69 | 30 | 4.90 | 9.90 | 9.01 | 6.85 | 133 | 9.42 | 258 |
|  |  |  | MS | | R | | MM | 52.7 | 210.7 | 22.1 | 128.6 | 5.9 | 129 | 65 | 32 | 7.90 | 10.23 | 10.44 | 10.78 | 130 | 10.47 | 303 |
|  |  |  | MS | | R | | MM | 43.6 | 197.3 | 17.2 | 114.0 | 5.9 | 129 | 65 | 32 | 7.89 | 9.22 | 10.23 | 9.39 | 184 | 8.44 | 299 |
|  |  | 2009 | DBE | | R | | MM | 69.7 | 264.4 | 19.2 | 180.4 | 6.0 | 129 | 65 | 32 | 8.19 | 9.24 | 9.64 | 9.12 | 199 | 7.25 | 329 |
|  |  |  | DBE | | R | | MM | 40.0 | 270.9 | 15.4 | 108.9 | 5.9 | 129 | 65 | 32 | 6.89 | 8.88 | 9.42 | 8.53 | 193 | 8.55 | 405 |
|  |  |  | DBE | | R | | MM | 33.2 | 170.2 | 20.8 | 117.0 | 5.6 | 129 | 65 | 32 | 7.82 | 9.60 | 10.76 | 9.24 | 100 | 7.77 | 331 |
| Jidong,  Heilongjiang | 45.2°N,  131.1°E | 2005 | PS | | R | | MM | 13.0 | 120.0 | 22.0 | 130.0 | 8.1 | 150 | 90 | 75 | 7.80 | 8.84 | 9.77 | 8.84 | 175 | 10.44 | 304 |
|  |  | 2006 | PS | | R | | MM | 14.0 | 118.0 | 22.0 | 140.0 | 7.9 | 105 | 75 | 90 | 7.80 | 8.84 | 8.81 | 8.87 | 122 | 9.02 | 338 |
|  |  | 2007 | PS | | R | | MM | 12.0 | 123.0 | 24.0 | 90.0 | 7.8 | 105 | 75 | 90 | 7.05 | 7.76 | 8.74 | 8.64 | 175 | 7.37 | 385 |
|  |  | 2008 | MS | | R | | MM | 23.8 | 177.9 | 17.0 | 63.0 | 7.6 | 105 | 75 | 90 | 4.82 | 6.49 | 7.73 | 7.69 | 146 | 9.12 | 208 |
|  |  | 2009 | MS | | R | | MM | 13.7 | 110.0 | 15.1 | 150.0 | 7.5 | 105 | 75 | 90 | 4.82 | 6.50 | 7.69 | 7.77 | 122 | 9.02 | 305 |
|  |  | 2010 | MS | | R | | MM | 10.4 | 120.0 | 20.7 | 103.0 | 7.9 | 105 | 75 | 90 | 4.93 | 6.49 | 7.72 | 7.82 | 200 | 10.95 | 278 |
|  |  |  | MS | | I | | MM | 13.5 | 121.0 | 21.8 | 161.0 | 7.7 | 105 | 75 | 90 | 4.90 | 6.51 | 7.68 | 7.78 | 146 | 10.43 | 240 |
| Junchuan Farm,  Heilongjiang | 47.3°N,  131.2°E | 2006 | Chern | | R | | MM | 26.0 | 145.0 | 15.4 | 182.0 | 6.7 | 150 | 92 | 30 | 6.08 | 7.61 | 7.98 | 8.43 | 164 | 7.81 | 265 |
|  |  |  | DBE | | R | | MM | 22.0 | 92.0 | 20.0 | 166.0 | 6.8 | 150 | 92 | 30 | 5.16 | 6.54 | 7.08 | 7.25 | 60 | 8.07 | 318 |
|  |  |  | Chern | | R | | MS | 22.6 | 96.0 | 27.0 | 120.0 | 6.8 | 150 | 92 | 30 | 6.84 | 8.43 | 10.29 | 9.90 | 114 | 9.43 | 238 |
|  |  | 2007 | BS | | I | | MM | 33.0 | 106.0 | 24.0 | 120.0 | 6.6 | 150 | 92 | 30 | 7.28 | 8.60 | 8.11 | 7.17 | 180 | 10.56 | 307 |
|  |  |  | BS | | R | | MM | 25.0 | 101.0 | 31.0 | 126.0 | 6.4 | 150 | 92 | 30 | 5.57 | 6.45 | 8.33 | 7.46 | 122 | 8.79 | 301 |
|  |  | 2008 | GS | | R | | MM | 27.1 | 113.0 | 20.0 | 153.0 | 6.3 | 150 | 92 | 30 | 7.88 | 8.06 | 8.10 | 7.73 | 121 | 8.85 | 301 |
|  |  |  | Castan | | R | | MM | 29.4 | 147.0 | 26.3 | 178.0 | 6.7 | 156 | 69 | 20 | 4.65 | 7.64 | 10.07 | 3.73 | 171 | 9.73 | 204 |
| Nongxingminshan Farm,  Heilongjiang | 47.4°N,  131.1°E | 2008 | Casta. | | R | | MM | 26.9 | 154.0 | 17.9 | 125.0 | 5.5 | 105 | 69 | 15 | 5.19 | 6.20 | 6.23 | 6.43 | 186 | 7.33 | 206 |
|  |  |  | Casta. | | I | | MM | 25.3 | 167.0 | 15.6 | 127.0 | 5.1 | 105 | 69 | 15 | 6.45 | 8.96 | 10.97 | 8.21 | 142 | 8.04 | 245 |
|  |  |  | Castan | | R | | MM | 26.7 | 142.0 | 27.0 | 125.0 | 4.9 | 105 | 69 | 15 | 6.38 | 8.79 | 8.69 | 9.46 | 141 | 7.35 | 230 |
|  |  |  | DBE | | R | | MM | 20.5 | 74.5 | 16.8 | 83.0 | 6.4 | 138 | 69 | 25 | 6.49 | 7.80 | 7.99 | 7.36 | 136 | 8.88 | 224 |
|  |  | 2009 | BS | | R | | MM | 26.3 | 76.3 | 17.3 | 105.0 | 6.4 | 138 | 69 | 25 | 5.72 | 6.85 | 7.96 | 7.13 | 119 | 8.01 | 234 |
|  |  |  | BS | | R | | MM | 19.8 | 150.7 | 16.9 | 145.0 | 5.8 | 138 | 69 | 25 | 5.84 | 6.96 | 8.03 | 7.24 | 122 | 7.84 | 207 |
|  |  |  | BS | | R | | MM | 17.2 | 105.6 | 15.1 | 112.0 | 6.1 | 138 | 69 | 25 | 5.40 | 6.54 | 7.20 | 7.34 | 137 | 9.44 | 341 |
|  |  |  | MS | | R | | MM | 17.1 | 88.4 | 25.6 | 91.0 | 6.6 | 138 | 69 | 25 | 6.10 | 7.23 | 8.34 | 7.52 | 138 | 9.77 | 427 |
|  |  | 2010 | MS | | R | | MM | 19.3 | 91.2 | 16.6 | 116.0 | 5.5 | 138 | 69 | 25 | 5.49 | 6.58 | 7.64 | 6.85 | 151 | 9.51 | 352 |
|  | 47.3°N,  131.1°E | 2008 | MS | | R | | MM | 39.6 | 311.0 | 21.9 | 153.0 | 7.4 | 133 | 46 | 26 | 7.11 | 9.54 | 9.39 | 9.19 | 195 | 8.12 | 233 |
|  |  |  | BS | | R | | MM | 55.1 | 253.0 | 17.0 | 187.0 | 8.0 | 133 | 46 | 26 | 6.71 | 8.86 | 9.39 | 9.11 | 174 | 8.93 | 279 |
|  |  |  | BS | | R | | MM | 42.9 | 323.0 | 19.3 | 210.0 | 7.7 | 133 | 46 | 26 | 4.61 | 6.83 | 7.74 | 8.15 | 195 | 8.61 | 250 |
|  |  | 2009 | BS | | R | | MM | 38.5 | 256.0 | 15.8 | 256.0 | 8.6 | 133 | 46 | 26 | 4.85 | 6.96 | 9.30 | 8.54 | 106 | 8.40 | 263 |
|  |  |  | BS | | R | | MS | 38.5 | 226.0 | 23.0 | 254.0 | 8.5 | 133 | 46 | 26 | 4.53 | 6.69 | 8.56 | 8.50 | 152 | 9.57 | 301 |
|  |  | 2010 | MS | | R | | MM | 48.9 | 254.2 | 16.3 | 93.5 | 5.6 | 118 | 55 | 45 | 6.33 | 8.03 | 8.31 | 6.23 | 130 | 8.24 | 218 |
|  |  |  | GS | | R | | MM | 50.1 | 213.2 | 26.2 | 168.6 | 5.8 | 118 | 55 | 45 | 6.69 | 8.75 | 9.58 | 9.12 | 124 | 10.08 | 295 |
|  |  |  | MS | | R | | MM | 36.7 | 216.8 | 19.7 | 77.3 | 5.5 | 118 | 55 | 45 | 6.60 | 7.53 | 8.59 | 7.53 | 144 | 9.26 | 347 |
|  |  |  | MS | | R | | MM | 41.9 | 224.3 | 28.0 | 65.9 | 5.8 | 118 | 55 | 45 | 7.08 | 9.45 | 10.10 | 8.42 | 141 | 8.52 | 233 |
|  | 47.4°N,  131.2°E | 2008 | DBE | | R | | MM | 58.9 | 337.0 | 19.3 | 150.0 | 5.4 | 157 | 87 | 26 | 5.32 | 8.42 | 9.21 | 8.02 | 170 | 9.27 | 264 |
|  |  | 2009 | AS | | R | | MM | 43.7 | 253.0 | 20.8 | 147.0 | 5.5 | 157 | 87 | 26 | 6.24 | 8.46 | 8.10 | 8.03 | 144 | 8.92 | 229 |
|  |  |  | AS | | R | | MM | 45.1 | 245.0 | 27.0 | 213.0 | 5.7 | 157 | 87 | 26 | 5.32 | 7.90 | 9.35 | 8.83 | 164 | 7.71 | 374 |
|  |  |  | PS | | R | | MM | 49.0 | 297.0 | 23.8 | 181.7 | 5.5 | 157 | 87 | 26 | 5.57 | 8.19 | 8.89 | 7.90 | 191 | 10.78 | 260 |
|  |  |  | AS | | R | | MM | 53.1 | 279.0 | 18.7 | 148.8 | 5.6 | 157 | 87 | 26 | 5.16 | 7.10 | 7.55 | 7.50 | 184 | 8.27 | 229 |
|  |  | 2010 | Chern | | R | | MM | 49.0 | 297.0 | 23.8 | 181.7 | 5.5 | 157 | 87 | 26 | 4.50 | 8.93 | 10.16 | 10.68 | 139 | 8.26 | 284 |
| Mingshui, Heilongjiang | 47.1°N,  125.5°E | 2008 | BS | | R | | MM | 32.3 | 94.0 | 22.0 | 200.0 | 8.1 | 105 | 75 | 90 | 7.00 | 7.84 | 9.32 | 8.88 | 178 | 9.04 | 227 |
|  |  |  | MS | | R | | MM | 7.6 | 63.7 | 21.5 | 130.0 | 8.3 | 105 | 75 | 90 | 6.95 | 7.95 | 9.85 | 9.45 | 197 | 9.60 | 334 |
|  |  |  | MS | | R | | MM | 11.2 | 103.0 | 23.5 | 160.0 | 8.3 | 105 | 75 | 90 | 7.50 | 9.00 | 10.50 | 9.49 | 148 | 10.12 | 168 |
| Puyang Farm, Heilongjiang | 47.2°N,  131.2°E | 2007 | DBE | | R | | MM | 37.8 | 145.3 | 20.1 | 102.1 | 6.4 | 137 | 69 | 35 | 7.53 | 9.44 | 10.40 | 10.08 | 160 | 10.38 | 209 |
|  |  |  | DBE | | R | | MM | 20.9 | 90.0 | 22.5 | 91.0 | 5.8 | 143 | 69 | 30 | 5.57 | 6.45 | 7.04 | 7.16 | 188 | 7.14 | 267 |
|  |  | 2008 | DBE | | R | | MM | 20.9 | 90.0 | 22.5 | 91.0 | 5.8 | 143 | 69 | 30 | 5.75 | 6.23 | 7.24 | 6.77 | 153 | 6.94 | 236 |
|  |  |  | Chern | | R | | MM | 20.9 | 90.0 | 22.5 | 91.0 | 5.8 | 143 | 69 | 30 | 7.52 | 8.35 | 9.03 | 9.04 | 180 | 9.05 | 233 |
|  |  | 2009 | BS | | R | | MM | 21.7 | 68.0 | 20.9 | 90.0 | 5.4 | 143 | 69 | 30 | 8.08 | 9.77 | 9.54 | 10.31 | 192 | 10.14 | 277 |
|  |  |  | AS | | R | | MM | 48.4 | 152.5 | 15.6 | 84.7 | 4.9 | 143 | 69 | 30 | 6.19 | 8.35 | 10.50 | 8.00 | 132 | 9.83 | 295 |
|  | 47.2°N,  131.1°E | 2008 | Chern | | R | | MM | 28.1 | 130.0 | 24.7 | 173.0 | 5.6 | 143 | 69 | 30 | 6.99 | 10.26 | 8.97 | 7.51 | 109 | 9.90 | 202 |
|  |  |  | Chern | | R | | MM | 39.8 | 223.9 | 25.5 | 142.9 | 5.3 | 143 | 69 | 30 | 7.32 | 9.10 | 10.79 | 8.93 | 133 | 10.28 | 209 |
|  |  | 2009 | Chern | | R | | MM | 30.2 | 170.6 | 23.1 | 138.4 | 5.8 | 143 | 69 | 30 | 8.16 | 8.78 | 10.18 | 9.85 | 190 | 9.96 | 233 |
|  |  |  | DBE | | R | | MM | 32.0 | 124.0 | 22.0 | 135.0 | 6.7 | 128 | 92 | 25 | 6.80 | 7.86 | 9.39 | 8.04 | 194 | 8.35 | 277 |
|  |  |  | BS | | R | | MM | 38.0 | 159.0 | 19.0 | 145.0 | 6.2 | 128 | 92 | 25 | 5.69 | 7.52 | 8.06 | 6.53 | 177 | 7.25 | 397 |
|  |  | 2010 | DBE | | R | | MM | 22.0 | 129.0 | 21.0 | 102.0 | 5.9 | 128 | 92 | 25 | 6.42 | 7.14 | 8.24 | 6.98 | 184 | 10.09 | 288 |
|  |  |  | BS | | R | | MM | 53.3 | 302.0 | 16.6 | 167.0 | 5.8 | 128 | 92 | 25 | 8.09 | 8.83 | 9.70 | 9.07 | 97 | 8.49 | 314 |
| Antu, Jilin | 43.1°N,  128.5°E | 2008 | BS | | R | | MM | 15.0 | 110.0 | 20.0 | 98.0 | 6.0 | 150 | 60 | 65 | 6.55 | 7.10 | 8.45 | 7.70 | 150 | 8.02 | 252 |
| Dunhua, Jilin | 43.2°N,  128.1°E | 2006 | BS | | R | | MM | 10.3 | 91.0 | 25.7 | 100.0 | 7.0 | 120 | 65 | 50 | 8.00 | 8.51 | 9.00 | 9.00 | 170 | 8.98 | 254 |
| Huichun, Jilin | 42.5°N,  130.2°E | 2008 | MS | | R | | MM | 40.9 | 106.4 | 21.4 | 154.0 | 7.2 | 130 | 70 | 90 | 4.70 | 5.79 | 6.78 | 6.71 | 183 | 6.76 | 264 |
| Ji'an, Jilin | 41.1°N,  126.1°E | 2007 | MS | | R | | MM | 11.0 | 81.0 | 16.9 | 210.0 | 8.1 | 188 | 60 | 90 | 4.70 | 7.65 | 11.00 | 8.81 | 132 | 9.49 | 239 |
| Jingyu, Jilin | 42.2°N,  127.2°E | 2008 | MS | | R | | MM | 27.8 | 157.0 | 21.1 | 142.0 | 7.8 | 150 | 60 | 65 | 6.08 | 8.64 | 10.89 | 10.64 | 137 | 8.96 | 233 |
|  |  |  | BS | | R | | MM | 22.3 | 125.6 | 25.5 | 87.0 | 7.7 | 150 | 60 | 65 | 7.29 | 9.86 | 11.18 | 9.68 | 190 | 8.68 | 256 |
| Longjin, Jilin | 42.5°N,  129.3°E | 2007 | BS | | R | | MM | 15.5 | 114.0 | 34.5 | 101.0 | 8.0 | 150 | 60 | 65 | 7.35 | 9.76 | 9.95 | 10.46 | 185 | 7.77 | 374 |
|  |  |  | BS | | R | | MM | 14.7 | 108.0 | 21.6 | 93.0 | 8.1 | 150 | 60 | 75 | 6.97 | 8.47 | 9.16 | 7.84 | 193 | 7.81 | 308 |
|  |  | 2008 | BS | | R | | MS | 7.8 | 121.0 | 27.7 | 95.0 | 8.1 | 150 | 60 | 75 | 5.20 | 6.70 | 7.17 | 7.37 | 198 | 7.85 | 353 |
|  |  |  | MS | | R | | MM | 9.2 | 115.0 | 27.0 | 115.0 | 8.2 | 150 | 60 | 65 | 4.87 | 8.07 | 9.10 | 8.13 | 195 | 7.81 | 224 |
|  |  |  | GS | | R | | MM | 10.4 | 132.0 | 27.0 | 85.0 | 8.1 | 150 | 60 | 65 | 6.97 | 8.47 | 9.16 | 7.84 | 154 | 10.12 | 256 |
| Tumen, Jilin | 42.6°N,  129.5°E | 2008 | MS | | R | | MM | 15.3 | 68.0 | 21.8 | 297.0 | 8.0 | 150 | 60 | 66 | 6.99 | 9.12 | 10.79 | 10.89 | 189 | 10.83 | 263 |
|  |  |  | MS | | R | | MM | 26.7 | 110.0 | 23.2 | 132.0 | 7.9 | 150 | 60 | 65 | 7.62 | 9.45 | 10.67 | 9.77 | 138 | 10.94 | 208 |
| **NE2 subregion** |  |  |  | |  | |  |  |  |  |  |  |  |  |  |  |  |  |  |  |  |  |
| Anda,  Heilongjiang | 46.3°N,  125.3°E | 2006 | BS | | R | | MM | 14.1 | 110.0 | 17.1 | 124.0 | 8.4 | 130 | 75 | 90 | 5.48 | 6.90 | 7.31 | 7.27 | 137 | 7.41 | 307 |
|  |  |  | BS | | R | | MS | 15.2 | 121.0 | 21.5 | 131.0 | 7.9 | 130 | 75 | 90 | 5.16 | 6.85 | 7.64 | 7.39 | 139 | 7.65 | 300 |
|  |  |  | BS | | R | | MM | 23.1 | 154.0 | 16.5 | 155.0 | 7.5 | 130 | 75 | 90 | 5.19 | 6.36 | 6.88 | 6.59 | 128 | 6.85 | 316 |
|  |  | 2007  2007 | Chern. | | R | | MM | 20.5 | 116.0 | 21.4 | 146.0 | 8.1 | 130 | 75 | 90 | 5.40 | 6.80 | 7.26 | 7.15 | 135 | 7.33 | 308 |
|  | 46.2°N,  125.2°E |  | Chern. | | R | | MM | 21.0 | 124.0 | 23.5 | 136.0 | 8.3 | 130 | 75 | 90 | 5.30 | 6.57 | 7.03 | 6.72 | 125 | 7.03 | 303 |
|  |  |  | Chern. | | R | | MM | 22.4 | 124.0 | 18.6 | 116.0 | 8.4 | 130 | 75 | 90 | 6.49 | 7.72 | 8.22 | 7.94 | 128 | 8.21 | 264 |
|  |  | 2008 | MS | | I | | MM | 21.3 | 157.0 | 17.8 | 125.0 | 7.9 | 130 | 75 | 90 | 5.78 | 7.22 | 7.76 | 7.40 | 127 | 7.76 | 277 |
|  |  |  | MS | | R | | MM | 19.5 | 136.0 | 24.0 | 174.0 | 7.8 | 130 | 75 | 90 | 5.77 | 6.85 | 7.48 | 7.60 | 162 | 7.60 | 343 |
|  | 46.1°N,  125.3°E | 2008 | MS | | R | | MM | 18.9 | 123.0 | 23.1 | 165.0 | 7.6 | 130 | 75 | 90 | 6.23 | 7.47 | 8.09 | 8.05 | 145 | 8.13 | 293 |
|  |  |  | BS | | R | | MM | 21.4 | 134.0 | 22.4 | 158.0 | 8.4 | 130 | 75 | 90 | 6.26 | 7.41 | 7.84 | 7.54 | 124 | 7.83 | 270 |
|  |  |  | BS | | R | | MM | 17.6 | 95.0 | 21.5 | 176.0 | 8.1 | 130 | 75 | 90 | 5.47 | 6.42 | 6.77 | 6.59 | 125 | 6.78 | 314 |
|  |  |  | BS | | R | | MM | 18.5 | 134.0 | 26.1 | 125.0 | 8.0 | 130 | 75 | 90 | 6.77 | 7.87 | 8.41 | 8.37 | 144 | 8.45 | 279 |
|  | 46.2°N,  125.5°E | 2008 | Chern | | R | | MM | 20.6 | 152.0 | 23.4 | 104.0 | 7.9 | 130 | 75 | 90 | 5.28 | 6.64 | 7.11 | 6.71 | 123 | 7.10 | 296 |
|  |  |  | MS | | R | | MM | 19.4 | 114.0 | 19.9 | 113.0 | 8.2 | 130 | 75 | 90 | 5.13 | 6.49 | 7.11 | 7.16 | 151 | 7.21 | 341 |
|  |  | 2009 | BS | | R | | MM | 21.2 | 105.0 | 24.1 | 114.0 | 7.8 | 130 | 75 | 90 | 5.47 | 6.42 | 6.77 | 6.74 | 135 | 6.82 | 330 |
|  |  |  | Chern | | R | | MM | 22.1 | 132.0 | 21.5 | 123.0 | 7.8 | 130 | 75 | 90 | 6.53 | 7.47 | 8.09 | 8.20 | 164 | 8.19 | 321 |
|  |  |  | MS | | R | | MM | 23.4 | 117.0 | 22.4 | 132.0 | 7.7 | 130 | 75 | 90 | 4.91 | 6.57 | 7.43 | 7.59 | 163 | 7.61 | 344 |
|  | 46.3°N,  125.6°E | 2009 | BS | | R | | MM | 25.6 | 151.0 | 23.7 | 124.0 | 8.0 | 130 | 75 | 90 | 5.22 | 6.44 | 6.94 | 6.56 | 123 | 6.90 | 305 |
|  |  | 2009 | MS | | R | | MM | 24.1 | 124.0 | 19.9 | 141.0 | 8.2 | 130 | 75 | 90 | 5.21 | 6.42 | 7.16 | 7.05 | 146 | 7.15 | 334 |
|  |  | 2010 | BS | | R | | MM | 30.1 | 136.0 | 20.6 | 132.0 | 8.1 | 130 | 75 | 90 | 4.14 | 7.05 | 7.94 | 6.72 | 121 | 7.93 | 262 |
|  |  | 2010 | Solon. | | R | | MS | 22.1 | 145.0 | 11.4 | 121.0 | 8.3 | 130 | 75 | 90 | 6.21 | 8.20 | 8.98 | 8.55 | 132 | 8.98 | 247 |
|  |  | 2010 | ASS | | R | | MM | 16.5 | 139.3 | 12.5 | 102.0 | 8.3 | 130 | 75 | 90 | 4.98 | 6.49 | 7.11 | 6.71 | 128 | 7.08 | 306 |
|  |  | 2010 | ASS | | I | | MM | 14.9 | 41.2 | 13.6 | 100.0 | 8.5 | 130 | 75 | 90 | 5.46 | 6.70 | 7.33 | 7.35 | 150 | 7.40 | 331 |
| Shuangcheng,  Heilongjiang | 45.2°N,  126.2°E | 2005 | FAS | | R | | MM | 14.1 | 63.0 | 15.4 | 164.0 | 8.3 | 120 | 33 | 45 | 7.19 | 7.93 | 9.15 | 8.85 | 147 | 8.96 | 269 |
| Zhaodong,  Heilongjiang | 46.1°N,  125.6°E | 2007 | MS | | R | | MM | 14.9 | 67.4 | 33.8 | 72.0 | 8.4 | 160 | 75 | 90 | 9.68 | 10.38 | 11.21 | 10.53 | 140 | 10.94 | 212 |
|  |  |  | MS | | R | | MS | 18.6 | 77.4 | 12.1 | 114.0 | 8.4 | 160 | 75 | 90 | 9.77 | 11.12 | 11.62 | 11.60 | 171 | 11.70 | 232 |
|  |  |  | MS | | R | | MM | 14.7 | 84.9 | 19.6 | 176.0 | 8.6 | 160 | 75 | 90 | 8.28 | 8.97 | 9.51 | 9.44 | 170 | 9.46 | 287 |
|  | 46.3°N,  125.6°E | 2007 | FAS | | R | | MM | 19.8 | 85.0 | 12.3 | 130.0 | 8.6 | 160 | 75 | 90 | 8.56 | 9.45 | 9.96 | 9.79 | 158 | 9.91 | 258 |
|  |  |  | Castan | | R | | MM | 17.8 | 105.0 | 13.4 | 100.0 | 8.5 | 160 | 75 | 90 | 7.92 | 8.55 | 9.15 | 8.79 | 146 | 8.99 | 265 |
|  |  | 2008 | ASS | | R | | MM | 18.7 | 156.0 | 28.5 | 250.0 | 8.4 | 160 | 75 | 90 | 7.93 | 8.45 | 9.17 | 8.76 | 147 | 8.95 | 269 |
|  |  |  | MS | | R | | MM | 13.9 | 125.7 | 13.9 | 116.0 | 8.1 | 160 | 75 | 90 | 5.87 | 7.24 | 7.61 | 7.99 | 202 | 7.92 | 399 |
| Lishu,  Jilin | 43.2°N,  124.2°E | 2008 | ASS | | R | | MM | 39.8 | 78.0 | 17.2 | 132.0 | 8.1 | 180 | 60 | 75 | 8.63 | 9.57 | 10.44 | 10.47 | 225 | 10.47 | 334 |
| Shuangliao,  Jilin | 43.3°N,  123.3°E | 2006 | MS | | R | | MM | 19.1 | 60.0 | 19.9 | 150.0 | 8.5 | 180 | 70 | 70 | 8.76 | 9.72 | 10.63 | 10.21 | 180 | 10.43 | 272 |
|  |  |  | MS | | R | | MM | 13.2 | 98.0 | 15.2 | 170.0 | 9.6 | 180 | 70 | 70 | 8.32 | 9.86 | 10.47 | 10.37 | 189 | 10.52 | 282 |
|  |  | 2007 | CLS | | R | | MM | 20.2 | 39.5 | 44.1 | 123.0 | 9.2 | 180 | 70 | 70 | 8.59 | 9.26 | 11.13 | 10.17 | 188 | 10.55 | 280 |
|  |  |  | CLS | | R | | MM | 26.6 | 162.0 | 17.0 | 140.0 | 8.7 | 180 | 70 | 70 | 8.50 | 9.10 | 10.40 | 9.92 | 197 | 10.07 | 306 |
|  |  | 2008 | CLS | | R | | MM | 17.5 | 115.0 | 41.6 | 127.0 | 8.9 | 180 | 71 | 71 | 8.76 | 9.72 | 10.63 | 10.21 | 180 | 10.43 | 272 |
|  |  |  | MS | | R | | MM | 14.2 | 82.0 | 13.5 | 170.0 | 8.9 | 180 | 71 | 71 | 8.15 | 9.81 | 10.60 | 10.18 | 179 | 10.55 | 268 |
|  |  |  | CLS | | R | | MM | 24.6 | 143.0 | 19.7 | 203.0 | 9.1 | 180 | 71 | 71 | 6.48 | 9.16 | 10.91 | 9.61 | 177 | 10.59 | 265 |
| Yitong,  Jilin | 43.2°N,  125.2°E | 2005 | GS | | I | | MM | 16.0 | 85.0 | 16.1 | 126.0 | 8.5 | 180 | 75 | 75 | 5.70 | 10.15 | 11.10 | 9.72 | 169 | 11.27 | 240 |
|  |  |  | GS | | R | | MM | 16.7 | 66.0 | 16.5 | 126.0 | 8.3 | 180 | 75 | 75 | 5.49 | 9.54 | 11.13 | 10.27 | 186 | 11.15 | 263 |
|  |  |  | Castan | | R | | MM | 11.5 | 134.0 | 13.6 | 130.0 | 8.3 | 160 | 75 | 75 | 8.53 | 9.83 | 10.66 | 10.43 | 171 | 10.60 | 257 |
|  |  |  | Castan | | R | | MM | 17.6 | 142.0 | 12.7 | 90.0 | 8.3 | 160 | 75 | 75 | 9.97 | 11.89 | 12.38 | 11.99 | 154 | 12.46 | 200 |
|  |  | 2006 | DC | | R | | MM | 15.4 | 89.0 | 16.8 | 150.0 | 8.2 | 180 | 75 | 75 | 6.47 | 8.63 | 10.27 | 7.78 | 149 | 9.80 | 249 |
|  |  |  | FAS | | R | | MM | 11.8 | 121.0 | 14.6 | 100.0 | 8.3 | 160 | 75 | 75 | 7.31 | 8.65 | 9.73 | 9.43 | 176 | 9.61 | 291 |
|  |  |  | MS | | R | | MM | 20.4 | 92.0 | 17.7 | 180.0 | 8.1 | 160 | 75 | 75 | 6.87 | 9.60 | 9.55 | 10.44 | 199 | 10.30 | 302 |
|  | 43.4°N,  126.3°E | 2007 | GS | | R | | MM | 19.2 | 69.0 | 17.0 | 115.0 | 8.2 | 160 | 75 | 75 | 7.37 | 10.23 | 11.39 | 10.73 | 164 | 11.38 | 230 |
|  |  |  | GS | | R | | MM | 20.4 | 136.0 | 17.0 | 85.0 | 8.1 | 180 | 75 | 75 | 8.11 | 9.28 | 10.40 | 9.43 | 162 | 10.08 | 258 |
|  |  |  | DC | | R | | MM | 16.3 | 130.0 | 15.0 | 48.0 | 8.2 | 180 | 75 | 75 | 7.41 | 8.15 | 9.96 | 9.12 | 193 | 9.44 | 321 |
|  |  | 2008 | ASS | | R | | MM | 20.8 | 58.0 | 14.1 | 93.0 | 8.7 | 180 | 60 | 75 | 8.05 | 8.67 | 10.03 | 9.18 | 171 | 9.57 | 284 |
|  |  |  | MS | | R | | MM | 15.1 | 98.0 | 17.8 | 210.0 | 8.0 | 160 | 60 | 75 | 6.95 | 8.02 | 8.88 | 8.72 | 179 | 8.82 | 321 |
|  | 43.3°N,  126.5°E | 2007 | MS | | R | | MM | 25.7 | 92.0 | 32.5 | 180.0 | 8.1 | 160 | 75 | 75 | 7.09 | 8.10 | 8.94 | 8.46 | 155 | 8.76 | 287 |
|  |  | 2008 | CLS | | R | | MM | 14.7 | 105.0 | 19.8 | 139.0 | 7.9 | 160 | 60 | 75 | 7.04 | 8.20 | 8.71 | 8.74 | 176 | 8.78 | 318 |
|  |  |  | CLS | | R | | MM | 18.9 | 84.0 | 23.9 | 72.0 | 8.2 | 160 | 60 | 75 | 6.78 | 8.13 | 9.12 | 8.70 | 166 | 8.98 | 296 |
|  |  |  | CLS | | R | | MM | 11.5 | 78.0 | 18.2 | 95.0 | 8.6 | 130 | 60 | 75 | 8.55 | 9.82 | 10.50 | 9.60 | 112 | 10.35 | 191 |
| Yushu,  Jilin | 44.5°N,  126.3°E | 2006 | MS | | R | | MM | 15.3 | 132.0 | 24.9 | 165.0 | 8.2 | 130 | 75 | 75 | 7.25 | 9.13 | 10.79 | 10.52 | 159 | 10.70 | 239 |
|  |  | 2007 | CLS | | R | | MM | 14.7 | 154.0 | 18.3 | 139.0 | 8.2 | 130 | 90 | 90 | 7.56 | 8.26 | 9.79 | 8.93 | 133 | 9.32 | 240 |
|  |  | 2008 | GS | | I | | MM | 20.5 | 154.0 | 24.6 | 70.0 | 8.1 | 130 | 75 | 75 | 7.25 | 9.13 | 10.79 | 10.52 | 159 | 10.70 | 239 |
|  | 44.3°N,  126.4°E | 2008 | MS | | R | | MM | 18.5 | 73.0 | 17.2 | 65.0 | 8.2 | 130 | 60 | 75 | 8.99 | 10.85 | 11.97 | 10.62 | 116 | 11.72 | 173 |
|  |  |  | MS | | R | | MS | 19.5 | 154.0 | 10.8 | 126.0 | 8.2 | 130 | 60 | 75 | 7.63 | 9.00 | 9.22 | 8.33 | 103 | 9.27 | 201 |
|  |  |  | MS | | R | | MM | 26.5 | 101.0 | 25.7 | 215.0 | 8.0 | 130 | 60 | 75 | 7.64 | 8.84 | 10.40 | 9.75 | 144 | 10.06 | 235 |
|  |  |  | FAS | | R | | MM | 20.9 | 132.0 | 15.4 | 220.0 | 8.2 | 180 | 60 | 75 | 8.54 | 10.17 | 10.65 | 10.04 | 184 | 8.90 | 327 |
| **NE3 subregion** |  |  |  | |  | |  |  |  |  |  |  |  |  |  |  |  |  |  |  |  |  |
| Lindian,  Heilongjiang | 48.5°N,  125.2°E | 2006 | Castan | | I | | MM | 57.7 | 80.0 | 12.5 | 294.0 | 7.3 | 150 | 75 | 90 | 6.77 | 8.15 | 8.63 | 8.57 | 157 | 8.70 | 292 |
|  |  | 2008 | Castan | | I | | MS | 32.8 | 70.0 | 7.6 | 124.0 | 6.6 | 159 | 75 | 90 | 6.80 | 8.72 | 9.36 | 7.17 | 120 | 9.30 | 222 |
|  |  | 2009 | Castan | | I | | MM | 26.1 | 98.0 | 12.4 | 108.0 | 7.4 | 159 | 75 | 90 | 8.49 | 10.86 | 10.53 | 10.68 | 152 | 11.00 | 225 |
|  | 48.4°N,  125.3°E | 2009 | Castan | | I | | MM | 31.0 | 56.0 | 11.1 | 224.0 | 6.0 | 159 | 75 | 90 | 9.66 | 10.17 | 11.67 | 11.13 | 184 | 11.29 | 257 |
|  |  | 2010 | MS | | I | | MM | 28.8 | 79.0 | 15.4 | 179.0 | 6.4 | 160 | 75 | 90 | 5.39 | 6.95 | 7.97 | 8.15 | 209 | 8.15 | 401 |
|  |  |  | CLS | | I | | MM | 29.2 | 96.0 | 7.9 | 170.0 | 6.1 | 161 | 75 | 90 | 5.10 | 6.87 | 7.94 | 7.54 | 170 | 7.85 | 346 |
| Longjiang,  Heilongjiang | 49.0°N,  122.5°E | 2006 | GS | | I | | MM | 17.2 | 85.0 | 6.9 | 147.0 | 8.5 | 161 | 75 | 90 | 5.34 | 6.94 | 8.04 | 7.44 | 162 | 7.86 | 332 |
|  |  | 2007 | MS | | I | | MM | 22.8 | 75.0 | 12.1 | 281.0 | 8.6 | 161 | 75 | 90 | 5.24 | 6.97 | 8.04 | 7.55 | 166 | 7.92 | 336 |
|  |  | 2008 | ISS | | I | | MM | 7.7 | 74.0 | 3.2 | 77.0 | 8.6 | 161 | 75 | 90 | 6.75 | 9.10 | 10.25 | 9.60 | 164 | 10.17 | 258 |
|  |  | 2009 | Castan | | I | | MM | 11.8 | 47.0 | 4.6 | 100.0 | 8.3 | 161 | 97 | 45 | 6.20 | 8.54 | 10.17 | 9.34 | 168 | 9.93 | 270 |
| Zhaozhou,  Heilongjiang | 44.5°N,  127.2°E | 2005 | Solon. | | I | | MM | 9.8 | 95.0 | 30.7 | 73.0 | 8.8 | 161 | 75 | 90 | 6.07 | 8.07 | 9.30 | 8.74 | 168 | 9.17 | 292 |
|  |  | 2006 | FAS | | I | | MM | 17.6 | 94.0 | 16.0 | 248.0 | 8.4 | 161 | 75 | 90 | 6.20 | 8.54 | 10.17 | 9.34 | 168 | 9.93 | 270 |
|  |  | 2007 | MS | | I | | MS | 15.6 | 130.0 | 21.5 | 282.0 | 8.7 | 161 | 75 | 90 | 6.07 | 8.07 | 9.30 | 8.74 | 168 | 9.17 | 292 |
|  |  | 2008 | ISS | | I | | MM | 16.4 | 132.0 | 15.6 | 234.0 | 8.6 | 161 | 75 | 90 | 4.77 | 6.57 | 7.84 | 7.17 | 165 | 7.64 | 346 |
|  |  | 2009 | Castan | | I | | MM | 14.5 | 140.0 | 6.0 | 88.0 | 8.6 | 161 | 75 | 90 | 4.67 | 6.44 | 7.50 | 7.10 | 170 | 7.42 | 366 |
|  | 45.6°N,  125.2°E | 2008 | Solon. | | I | | MM | 11.1 | 151.0 | 17.4 | 106.0 | 8.2 | 161 | 75 | 90 | 5.47 | 7.07 | 8.37 | 7.70 | 165 | 8.13 | 326 |
|  |  |  | FAS | | I | | MM | 11.8 | 110.0 | 6.2 | 108.0 | 8.8 | 161 | 75 | 90 | 6.37 | 7.97 | 9.27 | 8.57 | 164 | 9.02 | 291 |
|  |  | 2009 | FAS | | I | | MM | 9.8 | 98.0 | 15.3 | 95.0 | 8.7 | 161 | 75 | 90 | 6.97 | 9.32 | 10.47 | 9.83 | 164 | 10.39 | 253 |
|  |  |  | DBE | | I | | MM | 15.4 | 89.0 | 5.2 | 180.0 | 8.7 | 161 | 75 | 90 | 6.37 | 7.97 | 9.27 | 8.57 | 164 | 9.02 | 291 |
|  |  |  | DBE | | I | | MM | 14.5 | 78.0 | 5.5 | 230.0 | 8.5 | 161 | 75 | 90 | 5.10 | 7.17 | 8.70 | 7.87 | 166 | 8.44 | 314 |
|  | 45.5°N,  126.3°E | 2009 | DBE | | I | | MM | 14.5 | 95.0 | 12.6 | 250.0 | 8.3 | 161 | 75 | 90 | 4.63 | 6.38 | 7.60 | 6.98 | 165 | 7.41 | 357 |
|  |  |  | MS | | I | | MM | 15.6 | 92.0 | 21.6 | 240.0 | 8.6 | 161 | 75 | 90 | 5.44 | 7.44 | 8.84 | 8.10 | 166 | 8.62 | 308 |
|  |  | 2010 | MS | | I | | MM | 17.8 | 110.0 | 21.0 | 250.0 | 8.9 | 161 | 75 | 90 | 7.28 | 9.68 | 11.17 | 10.49 | 170 | 11.01 | 246 |
|  |  |  | BS | | I | | MM | 14.5 | 162.0 | 4.3 | 230.0 | 8.8 | 161 | 75 | 90 | 7.34 | 9.47 | 11.21 | 10.31 | 168 | 10.89 | 246 |
|  | 45.4°N,  126.4°E | 2006 | Chern. | | I | | MM | 16.3 | 113.0 | 11.3 | 140.0 | 8.8 | 161 | 75 | 90 | 7.50 | 9.71 | 11.47 | 10.54 | 168 | 11.15 | 240 |
|  |  |  | Chern. | | I | | MM | 15.9 | 150.0 | 7.0 | 170.0 | 8.5 | 161 | 75 | 90 | 7.10 | 9.20 | 10.87 | 10.01 | 168 | 10.58 | 253 |
|  |  | 2007 | Chern. | | I | | MS | 14.8 | 124.0 | 5.3 | 110.0 | 8.2 | 161 | 75 | 90 | 5.80 | 7.94 | 9.44 | 8.67 | 167 | 9.21 | 290 |
|  |  | 2008 | Chern. | | I | | MM | 12.8 | 114.0 | 10.6 | 120.0 | 8.0 | 161 | 75 | 90 | 6.34 | 9.20 | 10.84 | 10.11 | 171 | 10.70 | 254 |
|  |  | 2009 | Chern. | | I | | MM | 9.8 | 116.0 | 36.6 | 280.0 | 8.5 | 161 | 75 | 90 | 6.94 | 9.34 | 10.81 | 10.24 | 173 | 10.68 | 258 |
|  |  | 2010 | Chern. | | I | | MM | 14.2 | 113.0 | 7.5 | 172.0 | 8.4 | 161 | 75 | 90 | 6.94 | 9.34 | 10.81 | 10.24 | 173 | 10.68 | 258 |
| Baicheng,  Jilin | 45.3°N,  122.3°E | 2007 | DC | | I | | MM | 30.1 | 161.7 | 10.4 | 71.0 | 8.1 | 161 | 80 | 75 | 7.19 | 9.59 | 11.07 | 10.39 | 170 | 10.91 | 248 |
|  |  | 2008 | Chern. | | I | | MM | 24.5 | 107.4 | 67.0 | 190.0 | 8.1 | 161 | 80 | 75 | 6.54 | 8.17 | 9.41 | 8.70 | 162 | 9.18 | 284 |
|  |  | 2009 | Chern. | | I | | MM | 23.6 | 116.3 | 33.0 | 206.0 | 7.9 | 161 | 80 | 75 | 6.90 | 8.87 | 10.64 | 9.60 | 164 | 10.26 | 257 |
|  |  | 2010 | Chern | | I | | MM | 24.7 | 124.0 | 25.0 | 82.0 | 7.8 | 161 | 80 | 75 | 5.60 | 7.70 | 8.90 | 8.37 | 168 | 8.80 | 305 |
|  | 45.4°N,  122.5°E | 2005 | AS | | I | | MM | 19.8 | 91.0 | 17.0 | 69.0 | 7.5 | 161 | 80 | 75 | 5.54 | 7.10 | 8.27 | 8.10 | 187 | 8.23 | 358 |
|  |  | 2006 | AS | | I | | MM | 20.1 | 108.0 | 19.0 | 76.0 | 8.4 | 161 | 80 | 75 | 5.60 | 7.70 | 8.90 | 8.37 | 168 | 8.80 | 305 |
|  |  | 2007 | Chern. | | I | | MM | 20.3 | 95.8 | 17.6 | 63.0 | 8.2 | 161 | 80 | 75 | 6.86 | 9.21 | 10.36 | 9.71 | 164 | 10.28 | 255 |
|  |  | 2008 | BE | | I | | MM | 25.8 | 138.0 | 31.0 | 92.0 | 8.1 | 161 | 80 | 75 | 4.70 | 6.44 | 7.64 | 7.04 | 165 | 7.46 | 355 |
|  |  | 2009 | PS | | I | | MM | 20.0 | 119.0 | 8.0 | 155.0 | 7.6 | 161 | 80 | 75 | 4.28 | 5.49 | 6.09 | 5.48 | 143 | 5.99 | 393 |
|  |  | 2010 | AS | | I | | MP | 10.0 | 112.0 | 10.0 | 81.0 | 7.8 | 161 | 80 | 75 | 5.60 | 7.36 | 8.60 | 7.84 | 161 | 8.38 | 309 |
|  | 45.4°N,  123.0°E | 2005 | AS | | I | | MM | 10.0 | 73.0 | 10.0 | 119.0 | 8.1 | 161 | 80 | 75 | 7.27 | 9.41 | 10.94 | 10.27 | 171 | 10.73 | 254 |
|  |  |  | PS | | I | | MM | 20.0 | 93.0 | 14.0 | 108.0 | 7.9 | 161 | 80 | 75 | 5.64 | 7.74 | 9.17 | 8.54 | 170 | 8.99 | 302 |
|  |  | 2007 | PS | | I | | MM | 20.0 | 109.0 | 8.0 | 131.0 | 7.5 | 161 | 80 | 75 | 4.79 | 6.54 | 7.76 | 7.13 | 165 | 7.57 | 349 |
|  |  |  | PS | | I | | MM | 20.0 | 75.0 | 17.0 | 85.0 | 8.4 | 161 | 80 | 75 | 6.67 | 8.87 | 10.24 | 9.60 | 169 | 10.09 | 267 |
|  |  | 2008 | ASS | | I | | MM | 20.0 | 87.0 | 15.0 | 153.0 | 8.2 | 161 | 80 | 75 | 5.27 | 7.20 | 8.57 | 7.84 | 165 | 8.35 | 317 |
|  |  |  | PS | | I | | MM | 10.0 | 104.0 | 10.0 | 170.0 | 7.4 | 161 | 80 | 75 | 5.02 | 6.95 | 8.30 | 7.61 | 166 | 8.09 | 329 |
| Changlin,  Jilin | 44.2°N,  123.6°E | 2005 | ASS | | I | | MM | 17.1 | 45.0 | 10.2 | 89.0 | 7.8 | 161 | 60 | 65 | 5.14 | 7.77 | 9.11 | 8.47 | 168 | 9.03 | 297 |
|  |  |  | DBE | | I | | MM | 15.6 | 65.0 | 5.4 | 96.0 | 8.1 | 161 | 80 | 75 | 5.65 | 7.79 | 9.08 | 8.60 | 172 | 8.98 | 305 |
|  | 44.3°N,  123.4°E | 2006 | DBE | | I | | MP | 18.1 | 78.0 | 4.6 | 110.0 | 8.2 | 161 | 80 | 75 | 5.87 | 7.94 | 9.07 | 8.51 | 165 | 8.97 | 295 |
|  |  | 2007 | DBE | | I | | MM | 11.5 | 65.0 | 9.8 | 150.0 | 8.5 | 161 | 80 | 75 | 4.50 | 5.94 | 6.94 | 6.30 | 157 | 6.75 | 376 |
|  |  | 2008 | CS | | I | | MM | 19.8 | 64.0 | 8.0 | 112.0 | 8.4 | 161 | 71 | 71 | 4.30 | 5.78 | 6.69 | 6.34 | 167 | 6.61 | 405 |
| Da'an,  Jilin | 45.2°N,  123.3°E | 2005 | CS | | I | | MM | 31.0 | 89.0 | 16.0 | 75.0 | 7.8 | 161 | 80 | 75 | 3.90 | 5.37 | 6.40 | 5.87 | 163 | 6.24 | 419 |
|  |  |  | FAS | | I | | MM | 32.0 | 110.0 | 8.7 | 139.0 | 7.9 | 161 | 80 | 75 | 7.22 | 9.62 | 11.10 | 10.42 | 170 | 10.94 | 247 |
|  |  |  | CS | | I | | MM | 12.4 | 93.0 | 17.6 | 118.0 | 7.6 | 161 | 80 | 75 | 5.12 | 7.05 | 8.40 | 7.71 | 166 | 8.20 | 324 |
|  |  |  | CS | | I | | MM | 15.7 | 101.0 | 22.0 | 99.0 | 8.1 | 161 | 80 | 75 | 6.56 | 8.47 | 10.02 | 9.22 | 167 | 9.74 | 274 |
|  |  |  | BS | | I | | MM | 30.4 | 100.1 | 15.0 | 46.0 | 8.0 | 161 | 80 | 75 | 6.40 | 8.28 | 9.79 | 8.99 | 166 | 9.51 | 280 |
|  | 45.1°N,  123.2°E | 2006 | Castan | | I | | MM | 26.0 | 141.0 | 27.8 | 85.0 | 7.4 | 150 | 80 | 75 | 6.06 | 8.04 | 8.12 | 7.28 | 124 | 8.30 | 256 |
|  |  |  | MS | | I | | MM | 41.7 | 178.0 | 7.2 | 127.0 | 7.3 | 150 | 80 | 75 | 5.42 | 6.40 | 8.00 | 6.98 | 148 | 7.51 | 322 |
|  |  |  | Castan | | I | | MM | 14.4 | 206.0 | 23.5 | 123.0 | 7.6 | 150 | 80 | 75 | 5.48 | 7.08 | 7.08 | 6.36 | 120 | 7.25 | 285 |
|  |  |  | MS | | I | | MM | 26.3 | 206.0 | 31.7 | 88.0 | 8.1 | 150 | 80 | 75 | 6.24 | 7.08 | 8.40 | 7.13 | 130 | 7.94 | 276 |
|  | 45.3°N,  123.1°E | 2005 | MS | | I | | MM | 28.5 | 159.0 | 20.4 | 52.0 | 8.2 | 150 | 80 | 75 | 6.23 | 8.37 | 8.60 | 8.93 | 169 | 8.97 | 300 |
|  |  |  | BS | | I | | MM | 15.3 | 84.0 | 13.6 | 130.0 | 7.5 | 150 | 80 | 75 | 6.46 | 7.67 | 8.84 | 8.84 | 196 | 8.88 | 347 |
|  |  | 2006 | BS | | I | | MM | 32.0 | 228.0 | 20.6 | 92.0 | 7.9 | 150 | 80 | 75 | 9.75 | 10.13 | 10.47 | 10.29 | 124 | 10.36 | 204 |
|  |  |  | BS | | I | | MM | 27.1 | 129.0 | 11.0 | 115.0 | 7.8 | 150 | 80 | 75 | 7.03 | 7.46 | 8.68 | 8.37 | 187 | 8.45 | 349 |
|  |  | 2007 | Chern. | | I | | MM | 36.7 | 138.0 | 16.2 | 89.0 | 8.5 | 150 | 80 | 75 | 7.53 | 8.18 | 9.03 | 8.89 | 177 | 8.93 | 315 |
|  | 45.2°N,  123.2°E | 2006 | Chern. | | I | | MM | 35.2 | 130.0 | 8.6 | 87.0 | 8.1 | 150 | 80 | 75 | 6.90 | 7.88 | 8.58 | 8.71 | 194 | 8.70 | 350 |
|  |  |  | Chern. | | I | | MM | 23.8 | 90.4 | 58.4 | 90.0 | 8.4 | 180 | 80 | 75 | 8.54 | 8.83 | 9.03 | 9.03 | 125 | 8.93 | 239 |
|  |  | 2007 | Chern. | | I | | MM | 28.4 | 151.0 | 7.4 | 78.0 | 7.9 | 180 | 80 | 75 | 6.38 | 7.89 | 8.97 | 8.72 | 199 | 8.89 | 350 |
|  |  | 2008 | Chern. | | I | | MM | 22.3 | 213.0 | 41.4 | 67.0 | 8.3 | 180 | 80 | 75 | 6.42 | 7.94 | 9.06 | 8.61 | 189 | 8.90 | 333 |
|  |  | 2009 | Chern. | | I | | MM | 30.5 | 199.5 | 57.9 | 220.0 | 8.0 | 180 | 80 | 75 | 4.73 | 5.94 | 6.83 | 6.50 | 186 | 6.71 | 437 |
| Qian'an,  Jilin | 48.3°N,  125.6°E | 2006 | Chern. | | I | | MM | 9.4 | 127.0 | 9.8 | 118.0 | 8.1 | 180 | 57 | 90 | 6.32 | 7.94 | 9.03 | 8.61 | 189 | 8.91 | 334 |
|  |  |  | Chern. | | I | | MM | 14.9 | 149.0 | 17.0 | 139.0 | 7.8 | 180 | 57 | 90 | 5.58 | 6.35 | 7.65 | 6.58 | 159 | 7.20 | 356 |
|  |  |  | Chern. | | I | | MM | 10.1 | 141.0 | 8.3 | 108.0 | 8.5 | 180 | 57 | 90 | 5.67 | 6.54 | 7.05 | 7.13 | 204 | 7.10 | 449 |
|  | 48.2°N,  125.7°E | 2006 | Chern. | | I | | MM | 6.4 | 110.0 | 5.4 | 50.0 | 8.2 | 180 | 57 | 90 | 7.01 | 7.81 | 8.05 | 8.27 | 202 | 8.18 | 386 |
|  |  |  | Chern. | | I | | MM | 6.6 | 85.0 | 5.7 | 50.0 | 8.2 | 180 | 57 | 90 | 6.96 | 8.70 | 9.59 | 9.26 | 187 | 9.55 | 308 |
|  |  | 2007 | Chern. | | I | | MP | 6.4 | 65.0 | 9.7 | 60.0 | 8.2 | 180 | 57 | 90 | 7.19 | 8.93 | 9.81 | 9.49 | 187 | 9.78 | 301 |
|  |  |  | Chern. | | I | | MM | 10.0 | 98.0 | 4.4 | 65.0 | 8.1 | 180 | 57 | 90 | 7.10 | 8.84 | 9.72 | 9.40 | 187 | 9.69 | 304 |
|  |  | 2008 | Chern. | | I | | MM | 6.5 | 115.0 | 8.3 | 94.0 | 7.8 | 180 | 57 | 90 | 8.63 | 9.40 | 10.70 | 9.63 | 159 | 10.24 | 250 |
| Songyuan,  Jilin | 48.3°N,  125.6°E | 2006 | Chern. | | I | | MM | 7.8 | 78.0 | 11.0 | 88.0 | 7.8 | 180 | 75 | 75 | 7.20 | 8.94 | 9.83 | 9.51 | 187 | 9.79 | 301 |
|  |  |  | BP | | I | | MM | 9.1 | 98.0 | 10.0 | 66.0 | 7.9 | 180 | 75 | 75 | 8.63 | 9.40 | 10.70 | 9.93 | 174 | 10.30 | 269 |
|  |  | 2007 | BP | | I | | MM | 11.2 | 180.0 | 9.8 | 56.0 | 8.1 | 180 | 75 | 75 | 8.61 | 9.38 | 10.68 | 9.61 | 159 | 10.22 | 251 |
|  |  |  | BP | | I | | MM | 12.5 | 170.0 | 5.9 | 96.0 | 6.0 | 161 | 75 | 75 | 9.06 | 9.83 | 11.13 | 10.06 | 143 | 10.68 | 221 |
|  | 48.4°N,  125.7°E | 2005 | MS | | I | | MM | 13.4 | 54.0 | 6.9 | 68.0 | 5.6 | 161 | 75 | 75 | 9.10 | 9.87 | 11.17 | 10.10 | 143 | 10.72 | 220 |
|  |  | 2006 | MS | | I | | MM | 9.9 | 69.0 | 4.9 | 63.0 | 7.3 | 161 | 75 | 75 | 9.09 | 9.86 | 11.16 | 10.08 | 143 | 10.71 | 221 |
|  |  | 2007 | MS | | I | | MM | 18.1 | 96.0 | 6.6 | 126.0 | 5.3 | 161 | 75 | 75 | 9.08 | 9.85 | 11.15 | 10.08 | 143 | 10.70 | 221 |
|  |  | 2008 | MS | | I | | MM | 17.8 | 87.0 | 19.1 | 105.0 | 6.1 | 161 | 60 | 75 | 8.96 | 9.72 | 11.02 | 9.95 | 143 | 10.57 | 223 |
|  | 48.3°N,  125.8°E | 2008 | MS | | I | | MM | 16.5 | 85.0 | 9.8 | 125.0 | 5.9 | 161 | 60 | 75 | 5.87 | 8.33 | 9.17 | 7.87 | 142 | 9.10 | 258 |
|  |  |  | PS | | I | | MM | 14.0 | 89.0 | 6.6 | 146.0 | 7.9 | 161 | 60 | 75 | 7.97 | 8.57 | 8.47 | 8.46 | 113 | 8.54 | 233 |
|  |  |  | PS | | I | | MM | 14.5 | 92.0 | 4.3 | 129.0 | 7.1 | 161 | 60 | 75 | 7.97 | 8.57 | 8.47 | 8.47 | 113 | 8.55 | 233 |
|  |  |  | PS | | I | | MM | 13.9 | 93.0 | 6.1 | 163.0 | 6.8 | 161 | 60 | 75 | 6.70 | 7.57 | 8.30 | 8.20 | 180 | 8.25 | 345 |
| Taobei,  Jilin | 44.5°N,  127.2°E | 2007 | ASS | | I | | MM | 11.1 | 110.0 | 9.3 | 283.0 | 7.5 | 161 | 80 | 75 | 7.90 | 8.40 | 9.20 | 8.87 | 160 | 8.98 | 287 |
|  |  |  | PS | | I | | MM | 10.9 | 112.0 | 4.8 | 108.0 | 7.8 | 161 | 80 | 75 | 5.18 | 9.30 | 9.56 | 9.26 | 160 | 10.05 | 256 |
|  |  |  | ASS | | I | | MM | 9.9 | 131.0 | 11.9 | 54.0 | 8.5 | 161 | 80 | 75 | 6.11 | 7.89 | 9.33 | 9.12 | 191 | 9.27 | 325 |
|  |  | 2008 | DBE | | I | | MM | 14.5 | 141.0 | 13.1 | 93.0 | 8.2 | 161 | 71 | 71 | 4.65 | 7.34 | 8.51 | 8.97 | 211 | 8.95 | 368 |
|  |  |  | DBE | | I | | MP | 15.6 | 150.0 | 5.2 | 120.0 | 8.2 | 161 | 71 | 71 | 7.97 | 9.96 | 12.20 | 7.97 | 122 | 11.47 | 183 |
| Taonan,  Jilin | 45.1°N,  122.3°E | 2006 | DBE | | I | | MM | 10.0 | 98.0 | 18.0 | 137.0 | 7.2 | 161 | 80 | 75 | 5.40 | 7.27 | 10.31 | 9.44 | 200 | 9.79 | 320 |
|  |  | 2007 | CS | | I | | MM | 10.0 | 109.0 | 15.0 | 110.0 | 7.1 | 161 | 80 | 75 | 5.57 | 8.97 | 11.07 | 9.24 | 156 | 10.67 | 236 |
|  |  | 2008 | CS | | I | | MM | 20.0 | 121.0 | 11.0 | 120.0 | 7.5 | 161 | 80 | 75 | 6.84 | 9.06 | 9.63 | 9.06 | 152 | 9.70 | 256 |
|  |  | 2009 | FAS | | I | | MM | 20.0 | 127.0 | 11.0 | 145.0 | 7.6 | 161 | 80 | 75 | 7.97 | 9.96 | 12.20 | 7.97 | 122 | 11.47 | 183 |
|  | 45.2°N,  122.5°E | 2008 | CS | | I | | MM | 10.0 | 132.0 | 10.0 | 110.0 | 7.4 | 161 | 80 | 75 | 6.11 | 8.30 | 10.40 | 7.04 | 130 | 9.73 | 225 |
|  |  |  | CS | | I | | MM | 10.0 | 106.0 | 9.0 | 77.0 | 7.8 | 161 | 80 | 75 | 6.57 | 8.74 | 11.41 | 10.01 | 171 | 10.76 | 254 |
|  |  | 2009 | BS | | I | | MM | 10.0 | 117.0 | 16.0 | 116.0 | 8.1 | 161 | 80 | 75 | 6.94 | 9.04 | 9.97 | 9.54 | 165 | 9.95 | 266 |
|  |  |  | Castan | | I | | MM | 10.0 | 155.0 | 11.0 | 147.0 | 8.0 | 161 | 80 | 75 | 6.94 | 9.57 | 9.71 | 8.27 | 130 | 9.93 | 221 |
|  |  | 2010 | MS | | I | | MM | 10.0 | 81.0 | 9.0 | 119.0 | 8.0 | 161 | 80 | 75 | 4.14 | 5.04 | 5.84 | 4.77 | 129 | 5.58 | 391 |
|  |  |  | Castan | | I | | MM | 10.0 | 89.0 | 10.0 | 155.0 | 7.9 | 161 | 80 | 75 | 5.64 | 8.27 | 8.84 | 8.90 | 175 | 9.12 | 304 |
|  | 45.1°N,  123.0°E | 2006 | MS | | I | | MM | 10.0 | 110.0 | 12.0 | 96.0 | 7.5 | 161 | 80 | 75 | 7.50 | 10.97 | 11.44 | 11.91 | 185 | 12.01 | 243 |
|  |  |  | MS | | I | | MM | 10.0 | 100.0 | 16.0 | 185.0 | 7.6 | 150 | 80 | 75 | 6.78 | 7.74 | 8.94 | 8.72 | 182 | 8.81 | 326 |
|  |  | 2007 | BS | | I | | MM | 20.0 | 110.0 | 18.0 | 143.0 | 7.6 | 150 | 80 | 75 | 5.95 | 7.68 | 8.88 | 8.60 | 169 | 8.81 | 307 |
|  |  |  | BS | | I | | MM | 20.0 | 135.0 | 8.0 | 157.0 | 8.1 | 150 | 80 | 75 | 5.24 | 7.72 | 8.92 | 8.73 | 169 | 8.99 | 300 |
|  |  | 2008 | BS | | I | | MM | 20.0 | 94.0 | 19.0 | 175.0 | 7.8 | 150 | 80 | 75 | 4.54 | 7.57 | 8.77 | 8.77 | 174 | 8.99 | 307 |
|  |  |  | Chern. | | I | | MM | 20.0 | 122.0 | 16.0 | 187.0 | 8.2 | 150 | 80 | 75 | 5.38 | 7.61 | 8.81 | 8.18 | 155 | 8.70 | 289 |
| Zhenlai,  Jilin | 44.5°N,  127.3°E | 2005 | Chern. | | I | | MP | 11.9 | 178.0 | 10.0 | 260.0 | 8.5 | 150 | 80 | 75 | 6.04 | 7.71 | 8.91 | 8.50 | 163 | 8.79 | 298 |
|  |  |  | Chern. | | I | | MM | 15.6 | 54.0 | 5.0 | 128.0 | 8.9 | 150 | 80 | 75 | 5.80 | 7.34 | 8.54 | 8.55 | 191 | 8.61 | 349 |
|  |  |  | Chern. | | I | | MM | 8.9 | 96.0 | 4.8 | 105.0 | 8.6 | 150 | 80 | 75 | 4.97 | 7.39 | 8.59 | 8.48 | 173 | 8.69 | 317 |
|  |  |  | Chern. | | I | | MM | 14.2 | 89.0 | 3.3 | 230.0 | 8.8 | 150 | 80 | 75 | 5.68 | 7.32 | 8.52 | 8.61 | 194 | 8.65 | 353 |
|  |  | 2006 | BP | | I | | MM | 14.3 | 98.0 | 4.5 | 163.0 | 8.5 | 150 | 80 | 75 | 7.14 | 7.67 | 8.87 | 8.40 | 164 | 8.56 | 307 |
|  |  |  | BP | | I | | MM | 9.9 | 55.0 | 10.1 | 150.0 | 8.2 | 150 | 80 | 75 | 5.69 | 7.24 | 8.44 | 8.42 | 187 | 8.48 | 348 |
|  |  |  | BP | | I | | MM | 8.9 | 130.0 | 7.1 | 98.0 | 8.3 | 150 | 80 | 75 | 5.21 | 7.25 | 8.45 | 8.66 | 194 | 8.68 | 351 |
|  | 44.4°N,  127.3°E | 2006 | MS | | I | | MM | 15.6 | 152.0 | 28.4 | 132.0 | 8.6 | 150 | 80 | 75 | 5.64 | 7.79 | 8.99 | 8.87 | 174 | 9.04 | 306 |
|  |  |  | MS | | I | | MM | 14.9 | 142.0 | 11.6 | 116.0 | 8.6 | 150 | 80 | 75 | 6.42 | 7.71 | 8.91 | 8.88 | 193 | 8.93 | 340 |
|  |  | 2008 | MS | | I | | MM | 18.4 | 162.0 | 17.7 | 110.0 | 8.4 | 150 | 75 | 75 | 6.55 | 7.72 | 8.92 | 8.88 | 195 | 8.93 | 343 |
|  |  |  | MS | | I | | MM | 9.8 | 140.0 | 15.1 | 185.0 | 8.6 | 150 | 75 | 75 | 6.36 | 7.65 | 8.85 | 8.82 | 193 | 8.87 | 342 |
|  |  | 2009 | MS | | I | | MM | 11.4 | 115.0 | 8.0 | 260.0 | 8.5 | 150 | 75 | 75 | 5.30 | 7.34 | 8.54 | 8.75 | 194 | 8.77 | 348 |
|  |  |  | MS | | I | | MM | 12.5 | 98.0 | 7.3 | 140.0 | 8.6 | 150 | 75 | 75 | 8.55 | 9.59 | 11.25 | 10.61 | 169 | 10.87 | 248 |
|  |  | 2010 | MS | | I | | MM | 14.3 | 89.0 | 3.1 | 120.0 | 8.5 | 150 | 75 | 75 | 8.33 | 9.36 | 11.03 | 10.38 | 169 | 10.65 | 253 |
|  |  |  | MS | | I | | MM | 12.3 | 69.0 | 24.3 | 280.0 | 8.4 | 150 | 75 | 75 | 8.27 | 9.30 | 10.97 | 10.32 | 169 | 10.59 | 254 |
| **NE4 subregion** |  |  |  | |  | |  |  |  |  |  |  |  |  |  |  |  |  |  |  |  |  |
| Anshan,  Liaoning | 40.2°N,  123.0°E | 2006 | MS | | R | | MM | 14.5 | 81.6 | 18.7 | 47.2 | 5.9 | 180 | 120 | 120 | 6.47 | 8.52 | 9.12 | 8.78 | 178 | 9.19 | 310 |
| Benxi,  Liaoning | 41.2°N,  123.5°E | 2006 | BE | | R | | MM | 30.0 | 85.8 | 21.3 | 111.2 | 5.6 | 210 | 150 | 150 | 3.15 | 7.29 | 10.80 | 8.55 | 215 | 10.05 | 337 |
| Chaoyang,  Liaoning | 41.5°N,  120.5°E | 2006 | CS | | R | | MM | 11.5 | 88.5 | 14.7 | 187.1 | 5.8 | 180 | 120 | 120 | 7.52 | 8.00 | 9.18 | 8.87 | 212 | 8.94 | 374 |
| Faku,  Liaoning | 42.3°N,  123.2°E | 2006 | Chern. | | R | | MM | 19.6 | 87.6 | 22.0 | 208.6 | 5.8 | 210 | 150 | 150 | 5.31 | 6.93 | 7.38 | 7.02 | 194 | 7.41 | 415 |
| Fengcheng,  Liaoning | 40.3°N,  124.0°E | 2006 | BE | | R | | MM | 19.3 | 98.5 | 14.6 | 93.3 | 5.5 | 180 | 120 | 120 | 4.22 | 6.76 | 9.86 | 7.06 | 168 | 8.89 | 306 |
|  |  | 2007 | CS | | R | | MM | 23.8 | 164.8 | 31.3 | 97.3 | 5.5 | 210 | 150 | 150 | 5.75 | 8.79 | 7.92 | 5.68 | 145 | 8.68 | 278 |
|  | 40.3°N,  124.1°E | 2008 | DBE | | I | | MS | 19.3 | 110.8 | 14.6 | 93.3 | 5.5 | 240 | 150 | 150 | 5.89 | 10.41 | 9.84 | 8.65 | 202 | 10.58 | 302 |
| Fushun,  Liaoning | 41.4°N,  125.0°E | 2006 | AS | | R | | MM | 34.5 | 119.7 | 10.5 | 61.0 | 5.8 | 210 | 150 | 150 | 11.23 | 11.91 | 12.62 | 11.40 | 147 | 12.36 | 197 |
| Haicheng,  Liaoning | 40.5°N,  122.4°E | 2006 | BE | | R | | MM | 18.0 | 125.0 | 22.3 | 137.0 | 7.9 | 240 | 150 | 150 | 9.47 | 10.84 | 11.07 | 10.51 | 191 | 11.10 | 273 |
|  |  |  | BE | | R | | MM | 18.0 | 125.0 | 22.3 | 137.0 | 7.9 | 240 | 150 | 150 | 8.47 | 10.35 | 12.06 | 11.58 | 267 | 11.84 | 359 |
| Linyuan,  Liaoning | 41.2°N,  119.4°E | 2006 | CS | | R | | MM | 15.5 | 64.3 | 13.5 | 200.5 | 6.9 | 180 | 120 | 120 | 6.66 | 8.21 | 10.85 | 10.23 | 231 | 10.47 | 348 |
| Yingkou,  Liaoning | 40.4°N,  122.3°E | 2006 | CS | | R | | MM | 18.0 | 125.0 | 22.3 | 137.0 | 7.9 | 210 | 150 | 150 | 6.36 | 9.00 | 11.55 | 9.84 | 210 | 10.93 | 303 |
| Chengde,  Hebei | 41.2°N,  117.4°E | 2008 | BE | | R | | MM | 24.6 | 143.3 | 18.3 | 80.0 | 7.1 | 240 | 120 | 75 | 6.30 | 7.05 | 7.80 | 7.37 | 210 | 7.58 | 437 |
|  | 40.5°N,  118.1°E | 2007 | BE | | R | | MM | 13.6 | 135.4 | 27.1 | 99.0 | 7.2 | 225 | 138 | 90 | 5.31 | 6.83 | 7.02 | 6.95 | 209 | 7.15 | 461 |
| Fengning,  Hebei | 41.2°N,  116.2°E | 2006 | BE | | R | | MM | 15.1 | 114.0 | 37.6 | 130.0 | 7.5 | 225 | 60 | 150 | 7.04 | 8.06 | 10.16 | 8.91 | 223 | 9.49 | 370 |
|  |  | 2007 | BE | | R | | MM | 11.5 | 121.0 | 24.3 | 127.0 | 7.4 | 225 | 60 | 150 | 5.18 | 5.76 | 7.44 | 6.93 | 264 | 7.06 | 594 |
|  |  | 2008 | CS | | R | | MM | 14.1 | 141.0 | 19.5 | 114.0 | 7.2 | 180 | 60 | 120 | 5.58 | 5.97 | 7.46 | 7.04 | 224 | 7.14 | 494 |
|  |  | 2009 | CS | | R | | MM | 14.3 | 132.0 | 14.1 | 132.0 | 7.6 | 225 | 60 | 150 | 7.02 | 7.91 | 8.66 | 8.75 | 271 | 8.70 | 496 |
|  |  | 2010 | CS | | R | | MM | 12.0 | 98.0 | 15.9 | 100.0 | 7.5 | 180 | 60 | 120 | 5.58 | 5.97 | 7.46 | 6.93 | 208 | 7.07 | 464 |
|  | 41.3°N,  116.4°E | 2006 | CS | | R | | MM | 9.6 | 78.0 | 19.6 | 120.0 | 7.3 | 180 | 60 | 120 | 7.02 | 7.97 | 10.08 | 8.94 | 187 | 9.43 | 316 |
|  |  | 2007 | CS | | R | | MM | 15.1 | 154.0 | 27.9 | 190.0 | 7.6 | 225 | 60 | 150 | 7.04 | 8.06 | 10.16 | 8.91 | 223 | 9.49 | 370 |
|  |  | 2008 | BE | | R | | MM | 17.8 | 108.0 | 13.5 | 154.0 | 7.2 | 180 | 60 | 120 | 5.58 | 5.97 | 7.46 | 7.04 | 224 | 7.14 | 494 |
|  |  | 2009 | BE | | R | | MM | 17.6 | 81.6 | 19.8 | 132.0 | 7.1 | 225 | 60 | 150 | 7.02 | 7.91 | 8.66 | 8.75 | 271 | 8.70 | 496 |
|  |  | 2010 | CS | | R | | MM | 6.5 | 69.4 | 18.2 | 83.0 | 6.9 | 180 | 60 | 120 | 7.02 | 7.97 | 10.08 | 8.94 | 187 | 9.43 | 316 |
|  |  |  | CS | | R | | MM | 17.1 | 131.0 | 17.8 | 246.0 | 6.8 | 180 | 60 | 120 | 5.58 | 5.97 | 7.46 | 6.93 | 208 | 7.07 | 464 |
|  | 41.1°N,  116.4°E | 2005 | CS | | R | | MM | 16.0 | 121.0 | 17.9 | 78.0 | 7.3 | 225 | 60 | 150 | 6.03 | 7.01 | 8.51 | 7.80 | 229 | 8.10 | 445 |
|  |  |  | CS | | R | | MM | 14.7 | 125.0 | 12.4 | 84.0 | 7.3 | 150 | 60 | 120 | 6.68 | 7.04 | 7.20 | 7.10 | 109 | 7.14 | 276 |
|  |  | 2006 | CS | | R | | MM | 16.5 | 113.0 | 12.3 | 83.0 | 7.2 | 225 | 60 | 150 | 5.96 | 7.17 | 7.88 | 7.80 | 237 | 7.86 | 475 |
|  | 41.1°N,  116.5°E | 2006 | CS | | I | | MM | 17.2 | 108.0 | 10.5 | 76.0 | 7.2 | 150 | 60 | 120 | 5.88 | 6.15 | 6.51 | 6.41 | 128 | 6.39 | 344 |
|  |  | 2007 | BE | | R | | MS | 11.6 | 110.0 | 10.1 | 79.0 | 7.2 | 225 | 60 | 120 | 7.46 | 7.65 | 7.76 | 7.01 | 84 | 7.72 | 218 |
|  |  | 2008 | BE | | R | | MS | 9.6 | 88.2 | 19.6 | 83.0 | 7.2 | 150 | 60 | 120 | 4.74 | 6.09 | 6.21 | 6.18 | 144 | 6.36 | 378 |
|  |  | 2009 | BE | | R | | MM | 18.6 | 76.6 | 21.2 | 343.0 | 7.3 | 150 | 60 | 120 | 5.88 | 6.15 | 6.51 | 6.41 | 128 | 6.39 | 344 |
|  |  | 2010 | FAS | | R | | MM | 19.3 | 111.8 | 15.9 | 104.0 | 6.9 | 225 | 60 | 120 | 7.46 | 7.65 | 7.76 | 7.01 | 84 | 7.72 | 218 |
|  | 41.0°N,  116.3°E | 2006 | CS | | R | | MM | 15.2 | 75.8 | 17.8 | 129.0 | 7.4 | 150 | 60 | 120 | 4.74 | 6.09 | 6.21 | 6.18 | 144 | 6.36 | 378 |
|  |  |  | FAS | | R | | MS | 27.3 | 88.4 | 12.4 | 139.0 | 7.2 | 225 | 60 | 150 | 6.03 | 7.01 | 8.51 | 7.80 | 229 | 8.10 | 445 |
|  |  | 2009 | FAS | | R | | MS | 30.4 | 99.4 | 18.9 | 129.0 | 7.4 | 180 | 60 | 120 | 5.58 | 5.97 | 7.46 | 6.93 | 208 | 7.07 | 464 |
|  |  |  | FAS | | I | | MM | 33.4 | 102.2 | 17.7 | 120.0 | 7.5 | 180 | 60 | 120 | 7.04 | 8.06 | 10.16 | 8.91 | 182 | 9.49 | 306 |
|  |  |  | FAS | | R | | MM | 13.9 | 68.4 | 11.6 | 110.0 | 7.3 | 225 | 60 | 150 | 7.02 | 7.97 | 10.08 | 8.94 | 229 | 9.42 | 383 |
|  |  |  | BE | | R | | MM | 19.0 | 138.0 | 15.2 | 104.0 | 6.9 | 180 | 60 | 120 | 7.02 | 7.91 | 8.66 | 8.75 | 227 | 8.73 | 410 |
| Tangshan,  Hebei | 39.3°N,  118.1°E | 2006 | BE | | R | | MM | 15.3 | 89.7 | 12.3 | 38.0 | 7.2 | 225 | 60 | 150 | 7.48 | 7.66 | 7.75 | 7.00 | 81 | 7.73 | 213 |
|  |  |  | BE | | R | | MM | 25.0 | 89.7 | 16.2 | 68.0 | 7.3 | 225 | 60 | 150 | 7.42 | 8.56 | 9.04 | 9.02 | 226 | 9.07 | 392 |
|  |  |  | CS | | R | | MM | 25.0 | 88.6 | 13.5 | 115.0 | 7.2 | 225 | 60 | 150 | 5.81 | 7.62 | 7.63 | 7.86 | 223 | 7.93 | 443 |
|  |  | 2007 | FAS | | R | | MM | 16.3 | 94.5 | 11.7 | 38.0 | 7.2 | 225 | 60 | 150 | 7.44 | 8.28 | 8.58 | 7.57 | 149 | 8.52 | 289 |
|  |  |  | FAS | | R | | MM | 18.1 | 89.4 | 11.4 | 36.0 | 7.3 | 225 | 60 | 150 | 7.90 | 9.00 | 9.15 | 9.39 | 229 | 9.32 | 387 |
|  |  |  | BE | | R | | MM | 21.1 | 104.5 | 12.7 | 65.0 | 7.0 | 225 | 60 | 150 | 6.12 | 7.09 | 7.50 | 6.94 | 177 | 7.41 | 383 |
|  |  | 2008 | CS | | R | | MM | 28.6 | 112.5 | 17.9 | 50.0 | 7.5 | 225 | 60 | 150 | 6.04 | 7.02 | 8.22 | 7.80 | 236 | 7.97 | 467 |
|  |  |  | BE | | R | | MM | 21.6 | 98.7 | 12.3 | 62.0 | 6.8 | 225 | 60 | 150 | 4.75 | 6.10 | 6.15 | 6.32 | 215 | 6.35 | 534 |
|  |  |  | CS | | R | | MM | 24.9 | 88.4 | 14.6 | 85.0 | 7.5 | 225 | 60 | 150 | 7.28 | 8.40 | 9.36 | 7.39 | 161 | 9.06 | 289 |
|  | 39.3°N,  118.0°E | 2007 | FAS | | R | | MM | 21.3 | 115.4 | 12.8 | 105.0 | 7.7 | 225 | 60 | 150 | 7.44 | 8.01 | 8.86 | 8.19 | 183 | 8.55 | 342 |
|  |  |  | BE | | R | | MS | 29.0 | 114.5 | 18.3 | 117.0 | 7.4 | 225 | 60 | 150 | 8.15 | 8.63 | 9.12 | 8.95 | 188 | 8.97 | 334 |
|  |  |  | BE | | R | | MM | 18.2 | 108.9 | 14.6 | 96.0 | 6.6 | 225 | 60 | 150 | 7.66 | 8.28 | 8.30 | 8.36 | 161 | 8.31 | 316 |
|  |  | 2008 | FAS | | R | | MM | 10.1 | 89.8 | 21.1 | 118.0 | 7.7 | 225 | 60 | 150 | 6.50 | 7.38 | 8.20 | 7.33 | 181 | 7.93 | 365 |
|  |  |  | CS | | R | | MM | 16.2 | 98.6 | 12.7 | 89.0 | 7.6 | 225 | 60 | 150 | 7.36 | 7.77 | 7.92 | 7.50 | 122 | 7.84 | 271 |
|  |  | 2009 | FAS | | R | | MS | 24.0 | 79.6 | 14.6 | 114.0 | 7.2 | 225 | 60 | 150 | 7.17 | 8.97 | 9.46 | 9.67 | 249 | 9.68 | 406 |
|  | 39.3°N,  118.1°E | 2006 | FAS | | R | | MS | 25.0 | 78.5 | 14.9 | 116.0 | 7.6 | 225 | 60 | 150 | 6.65 | 8.76 | 9.36 | 9.48 | 246 | 9.57 | 406 |
|  |  | 2007 | FAS | | I | | MM | 28.6 | 104.1 | 18.5 | 118.0 | 6.6 | 225 | 60 | 150 | 7.21 | 8.78 | 9.65 | 9.98 | 294 | 9.93 | 479 |
|  |  |  | FAS | | R | | MM | 16.2 | 105.2 | 12.3 | 93.0 | 7.5 | 225 | 60 | 150 | 6.70 | 8.61 | 8.83 | 8.45 | 200 | 8.97 | 353 |
|  |  | 2008 | BE | | R | | MS | 10.1 | 119.7 | 12.2 | 97.0 | 7.1 | 225 | 60 | 150 | 6.56 | 8.09 | 8.51 | 8.65 | 239 | 8.67 | 435 |
|  |  |  | BE | | R | | MM | 18.6 | 98.6 | 16.5 | 106.0 | 7.1 | 225 | 60 | 150 | 5.62 | 7.73 | 8.56 | 8.92 | 277 | 8.88 | 499 |
|  |  | 2009 | FAS | | R | | MM | 18.6 | 99.6 | 13.8 | 20.0 | 7.2 | 225 | 60 | 150 | 5.81 | 7.88 | 7.67 | 7.77 | 207 | 8.03 | 407 |
|  | 39.5°N,  118.2°E | 2008 | CS | | R | | MM | 19.7 | 79.5 | 32.2 | 55.0 | 7.6 | 225 | 60 | 150 | 10.93 | 11.54 | 11.71 | 11.55 | 160 | 11.66 | 224 |
|  |  |  | BE | | R | | MM | 18.9 | 65.9 | 45.6 | 78.0 | 7.4 | 225 | 60 | 150 | 7.33 | 7.77 | 7.98 | 6.59 | 116 | 7.98 | 257 |
|  |  | 2009 | BE | | R | | MM | 28.1 | 89.4 | 80.3 | 24.0 | 7.5 | 135 | 60 | 90 | 5.24 | 6.72 | 6.87 | 7.05 | 145 | 7.10 | 340 |
|  |  | 2010 | BE | | R | | MM | 18.0 | 78.9 | 30.7 | 42.0 | 7.4 | 135 | 60 | 90 | 8.47 | 10.54 | 10.28 | 9.63 | 110 | 10.61 | 187 |
|  | 39.3°N,  118.4°E | 2008 | CS | | R | | MM | 13.1 | 89.5 | 35.4 | 72.0 | 7.8 | 225 | 60 | 150 | 6.03 | 7.40 | 7.82 | 7.65 | 212 | 7.85 | 426 |
|  |  | 2009 | FAS | | R | | MM | 14.2 | 101.0 | 39.3 | 91.0 | 6.9 | 225 | 60 | 150 | 7.10 | 8.53 | 8.55 | 7.88 | 169 | 8.67 | 315 |
|  |  |  | FAS | | R | | MM | 15.1 | 115.0 | 31.5 | 156.0 | 7.0 | 225 | 60 | 150 | 9.21 | 9.46 | 9.81 | 9.36 | 120 | 9.61 | 219 |
|  |  | 2010 | BE | | R | | MM | 14.7 | 109.4 | 27.0 | 88.0 | 7.0 | 225 | 60 | 150 | 8.82 | 9.81 | 10.04 | 9.69 | 178 | 10.03 | 284 |
|  |  |  | CS | | R | | MM | 21.2 | 125.4 | 39.3 | 117.0 | 6.9 | 225 | 60 | 150 | 6.10 | 7.65 | 7.83 | 8.37 | 273 | 8.23 | 529 |
| Zunhua,  Hebei | 40.1°N,  117.5°E | 2007 | BE | | R | | MM | 17.8 | 121.0 | 17.8 | 787.0 | 7.1 | 225 | 60 | 120 | 10.83 | 11.31 | 11.57 | 11.45 | 161 | 11.46 | 229 |
|  |  | 2010 | CS | | R | | MM | 21.2 | 131.0 | 22.4 | 98.0 | 7.2 | 225 | 60 | 150 | 10.31 | 11.22 | 11.03 | 11.49 | 221 | 11.28 | 309 |
|  | 40.0°N,  117.6°E | 2008 | FAS | | R | | MM | 18.9 | 124.0 | 25.9 | 95.0 | 7.1 | 225 | 60 | 120 | 10.23 | 10.70 | 10.85 | 10.98 | 175 | 10.80 | 260 |
|  |  |  | BE | | R | | MS | 21.0 | 105.4 | 28.9 | 89.0 | 7.2 | 225 | 60 | 150 | 9.75 | 10.34 | 10.19 | 9.81 | 115 | 10.26 | 199 |
|  |  | 2009 | BE | | R | | MM | 21.8 | 110.2 | 63.2 | 81.0 | 7.4 | 225 | 60 | 120 | 10.23 | 10.70 | 10.85 | 10.98 | 175 | 10.80 | 260 |
|  |  |  | BE | | R | | MM | 17.3 | 89.6 | 65.5 | 51.0 | 7.4 | 225 | 60 | 150 | 9.75 | 10.34 | 10.19 | 9.81 | 115 | 10.26 | 199 |
|  | 40.1°N,  118.0°E | 2007 | BE | | R | | MM | 15.9 | 87.9 | 29.5 | 66.0 | 7.5 | 225 | 60 | 120 | 10.83 | 11.31 | 11.57 | 11.45 | 161 | 11.46 | 229 |
|  |  | 2008 | BE | | R | | MM | 14.7 | 85.6 | 27.1 | 51.0 | 7.3 | 225 | 60 | 150 | 10.31 | 11.22 | 11.03 | 11.49 | 221 | 11.28 | 309 |
| **NCP1 subregion** |  |  |  | |  | |  |  |  |  |  |  |  |  |  |  |  |  |  |  |  |  |
| Anguo,  Hebei | 38.2°N,  115.1°E | 2009 | CS | | R | | MW | 30.2 | 205.0 | 9.3 | 101.0 | 6.5 | 160 | 90 | 120 | 6.72 | 8.02 | 8.88 | 8.34 | 156 | 8.73 | 291 |
|  |  |  | FAS | | I | | MW | 12.6 | 42.8 | 20.1 | 120.5 | 7.4 | 180 | 120 | 120 | 7.87 | 8.78 | 9.98 | 9.28 | 175 | 9.64 | 290 |
|  |  | 2010 | CS | | R | | MW | 30.4 | 178.0 | 16.7 | 130.0 | 7.8 | 180 | 120 | 120 | 6.56 | 7.56 | 9.06 | 8.66 | 212 | 8.81 | 378 |
|  |  |  | FAS | | R | | MW | 31.2 | 52.8 | 19.1 | 134.7 | 6.8 | 160 | 90 | 120 | 6.13 | 9.13 | 9.23 | 8.43 | 143 | 9.54 | 249 |
|  | 38.3°N,  115.1°E | 2007 | BE | | R | | MW | 12.0 | 129.0 | 16.5 | 143.0 | 8.0 | 180 | 120 | 120 | 5.48 | 8.58 | 9.69 | 9.18 | 187 | 9.76 | 304 |
|  |  | 2008 | BE | | R | | MW | 18.5 | 100.0 | 17.7 | 145.0 | 7.1 | 180 | 60 | 120 | 7.31 | 8.40 | 8.20 | 8.33 | 153 | 8.41 | 297 |
|  |  | 2009 | BE | | R | | MW | 10.0 | 57.1 | 18.4 | 149.4 | 7.5 | 220 | 60 | 150 | 7.03 | 8.41 | 9.53 | 9.23 | 237 | 9.40 | 396 |
|  | 38.2°N,  115.2°E | 2008 | BE | | R | | MW | 20.0 | 54.2 | 19.6 | 158.9 | 6.8 | 180 | 60 | 120 | 7.82 | 9.45 | 9.17 | 9.36 | 166 | 9.51 | 281 |
|  |  |  | FAS | | R | | MW | 10.0 | 58.0 | 21.1 | 172.2 | 6.9 | 180 | 60 | 120 | 6.41 | 7.52 | 9.04 | 8.41 | 193 | 8.69 | 351 |
|  | 38.3°N,  115.2°E | 2008 | FAS | | R | | MW | 15.4 | 49.9 | 27.1 | 173.8 | 7.1 | 220 | 60 | 150 | 7.66 | 8.29 | 8.63 | 8.41 | 174 | 8.53 | 326 |
|  |  | 2010 | CS | | R | | MW | 11.9 | 59.5 | 21.3 | 186.5 | 8.0 | 160 | 60 | 90 | 6.68 | 7.21 | 8.21 | 7.92 | 181 | 8.00 | 360 |
|  |  |  | FAS | | R | | MW | 12.8 | 42.8 | 21.6 | 186.6 | 7.6 | 180 | 60 | 120 | 6.67 | 7.15 | 8.17 | 7.87 | 199 | 7.94 | 395 |
| Anping,  Hebei | 38.1°N,  115.3°E | 2010 | FAS | | R | | MW | 15.4 | 48.0 | 17.3 | 85.0 | 7.6 | 160 | 60 | 90 | 4.63 | 7.12 | 7.98 | 7.76 | 171 | 8.09 | 339 |
|  |  |  | FAS | | R | | MW | 13.5 | 107.4 | 24.1 | 112.0 | 8.1 | 220 | 60 | 150 | 6.70 | 7.70 | 8.13 | 8.12 | 217 | 8.15 | 418 |
|  | 38.1°N,  115.4°E | 2006 | CS | | R | | MW | 28.4 | 23.5 | 14.4 | 117.0 | 7.9 | 160 | 60 | 90 | 6.63 | 7.51 | 8.47 | 7.57 | 139 | 8.17 | 284 |
|  |  | 2007 | CS | | I | | MW | 12.1 | 43.3 | 10.5 | 125.0 | 8.5 | 160 | 60 | 90 | 6.95 | 7.28 | 8.64 | 8.15 | 185 | 8.28 | 354 |
|  |  |  | CS | | R | | MW | 11.6 | 84.9 | 20.3 | 130.5 | 7.6 | 160 | 60 | 90 | 7.04 | 8.34 | 8.93 | 9.00 | 183 | 9.03 | 322 |
|  | 38.2°N,  115.4°E | 2008 | CS | | R | | MW | 15.5 | 98.0 | 14.7 | 150.0 | 8.2 | 220 | 60 | 150 | 7.03 | 7.90 | 9.30 | 8.60 | 219 | 8.91 | 385 |
|  |  |  | CS | | R | | MW | 18.9 | 77.0 | 13.4 | 174.0 | 8.3 | 160 | 60 | 90 | 6.03 | 8.07 | 8.40 | 8.03 | 151 | 8.54 | 290 |
|  |  |  | CS | | R | | MW | 22.3 | 128.6 | 24.0 | 177.0 | 7.5 | 160 | 60 | 90 | 8.03 | 9.53 | 9.53 | 10.10 | 198 | 9.98 | 313 |
| Baixiang,  Hebei | 37.2°N,  114.4°E | 2007 | CS | | R | | MW | 11.7 | 142.0 | 17.8 | 143.0 | 6.9 | 180 | 60 | 120 | 3.95 | 7.13 | 6.75 | 6.11 | 155 | 7.27 | 348 |
|  | 37.2°N,  114.4°E | 2006 | CS | | R | | MW | 10.3 | 129.0 | 9.6 | 135.0 | 6.2 | 220 | 150 | 150 | 6.01 | 6.97 | 8.57 | 7.28 | 196 | 8.02 | 385 |
|  |  | 2007 | FAS | | R | | MW | 12.2 | 90.0 | 11.8 | 145.0 | 8.2 | 220 | 60 | 150 | 4.53 | 8.00 | 8.91 | 8.07 | 212 | 9.02 | 369 |
|  |  | 2008 | FAS | | I | | MW | 11.2 | 80.0 | 9.8 | 114.0 | 7.5 | 180 | 60 | 120 | 6.89 | 8.64 | 8.82 | 7.73 | 140 | 8.92 | 262 |
|  |  | 2009 | FAS | | R | | MW | 9.8 | 113.0 | 9.8 | 131.0 | 7.4 | 180 | 60 | 120 | 7.16 | 8.48 | 8.90 | 7.91 | 141 | 8.85 | 265 |
|  | 37.3°N,  114.4°E | 2008 | FAS | | R | | MW | 11.0 | 100.0 | 9.3 | 114.0 | 8.2 | 180 | 60 | 120 | 6.98 | 8.81 | 8.96 | 8.09 | 146 | 9.08 | 266 |
|  |  | 2009 | FAS | | R | | MW | 16.0 | 188.0 | 15.5 | 95.0 | 8.1 | 180 | 60 | 120 | 7.01 | 8.18 | 9.38 | 8.06 | 153 | 8.99 | 278 |
|  |  | 2010 | BE | | R | | MW | 12.0 | 239.0 | 21.4 | 126.0 | 7.5 | 220 | 60 | 150 | 7.13 | 8.51 | 9.12 | 8.24 | 181 | 9.00 | 320 |
|  | 37.3°N,  114.5°E | 2008 | BE | | R | | MW | 11.6 | 106.0 | 21.5 | 152.0 | 6.8 | 220 | 60 | 150 | 6.23 | 7.82 | 8.97 | 8.07 | 204 | 8.71 | 368 |
|  |  |  | FAS | | R | | MW | 11.8 | 83.0 | 36.2 | 132.0 | 7.4 | 180 | 60 | 120 | 6.41 | 8.33 | 9.05 | 7.76 | 151 | 8.95 | 277 |
|  |  | 2009 | CS | | R | | MW | 15.6 | 117.0 | 35.2 | 115.0 | 7.9 | 180 | 60 | 120 | 5.51 | 8.18 | 9.18 | 8.13 | 169 | 9.11 | 298 |
|  |  |  | FAS | | R | | MW | 16.5 | 79.0 | 20.8 | 171.0 | 6.8 | 160 | 60 | 90 | 5.81 | 6.41 | 7.53 | 7.13 | 175 | 7.26 | 385 |
|  | 37.2°N,  114.5°E | 2005 | BE | | R | | MW | 14.5 | 94.0 | 12.5 | 112.0 | 6.9 | 180 | 60 | 120 | 5.49 | 6.62 | 7.52 | 6.42 | 150 | 7.25 | 340 |
|  |  | 2006 | CS | | R | | MW | 12.3 | 90.0 | 14.6 | 201.0 | 7.6 | 160 | 60 | 90 | 5.81 | 6.41 | 7.35 | 6.98 | 166 | 7.11 | 376 |
|  |  |  | FAS | | R | | MW | 12.4 | 117.0 | 17.8 | 123.0 | 7.6 | 180 | 60 | 120 | 6.87 | 8.87 | 9.02 | 7.92 | 143 | 9.16 | 259 |
|  |  |  | FAS | | R | | MW | 13.5 | 94.0 | 15.6 | 141.0 | 7.9 | 160 | 60 | 90 | 3.99 | 6.32 | 7.49 | 6.27 | 149 | 7.29 | 336 |
|  |  | 2007 | BE | | R | | MW | 15.0 | 91.0 | 16.2 | 159.0 | 8.1 | 160 | 60 | 90 | 4.31 | 6.26 | 7.43 | 6.98 | 170 | 7.33 | 372 |
|  |  | 2008 | BE | | R | | MW | 9.8 | 94.0 | 16.8 | 153.0 | 8.4 | 160 | 60 | 90 | 6.83 | 7.71 | 8.08 | 7.92 | 150 | 8.06 | 306 |
|  |  | 2009 | CS | | R | | MW | 8.9 | 123.0 | 18.9 | 145.0 | 7.8 | 180 | 60 | 120 | 3.91 | 7.31 | 7.72 | 6.26 | 156 | 7.92 | 321 |
|  |  | 2010 | FAS | | R | | MW | 13.1 | 55.0 | 20.5 | 165.0 | 7.6 | 180 | 60 | 120 | 4.20 | 7.42 | 8.22 | 6.01 | 150 | 8.22 | 300 |
|  |  |  | CS | | R | | MW | 9.6 | 90.0 | 20.4 | 113.0 | 7.5 | 180 | 60 | 120 | 3.84 | 7.44 | 7.87 | 6.52 | 159 | 8.10 | 319 |
| Bazhou,  Hebei | 39.1°N,  116.2°E | 2007 | BE | | R | | MW | 41.2 | 72.5 | 12.8 | 114.0 | 7.6 | 180 | 60 | 120 | 5.91 | 8.83 | 9.17 | 9.00 | 180 | 9.48 | 302 |
|  |  | 2008 | BE | | I | | MW | 16.5 | 50.0 | 10.5 | 116.0 | 8.1 | 180 | 60 | 120 | 6.00 | 7.64 | 9.19 | 9.09 | 228 | 9.17 | 390 |
|  | 38.7°N,  116.3°E | 2008 | BE | | R | | MW | 24.0 | 62.5 | 20.4 | 117.0 | 8.0 | 220 | 60 | 150 | 5.94 | 8.33 | 8.57 | 7.82 | 191 | 8.74 | 345 |
|  |  |  | CS | | R | | MW | 44.6 | 74.5 | 13.3 | 118.0 | 7.4 | 220 | 60 | 150 | 8.36 | 10.32 | 10.79 | 8.66 | 160 | 10.80 | 240 |
|  |  | 2009 | FAS | | R | | MW | 12.6 | 119.5 | 20.5 | 119.0 | 8.1 | 220 | 60 | 150 | 6.01 | 8.14 | 8.74 | 7.97 | 197 | 8.75 | 355 |
|  |  |  | FAS | | I | | MW | 20.0 | 11.4 | 13.7 | 119.0 | 8.0 | 220 | 60 | 150 | 8.44 | 10.08 | 10.62 | 8.72 | 159 | 10.56 | 244 |
| Botou,  Hebei | 38.2°N,  116.2°E | 2005 | FAS | | R | | MW | 7.5 | 100.0 | 10.5 | 181.0 | 6.7 | 180 | 60 | 120 | 6.02 | 7.35 | 7.71 | 7.68 | 181 | 7.80 | 369 |
|  |  | 2006 | FAS | | R | | MW | 12.3 | 120.0 | 15.4 | 126.0 | 6.5 | 220 | 60 | 150 | 6.99 | 8.18 | 8.55 | 8.54 | 216 | 8.61 | 394 |
|  |  | 2007 | CS | | R | | MW | 11.7 | 100.0 | 17.8 | 168.0 | 8.2 | 220 | 60 | 150 | 6.97 | 8.15 | 8.52 | 8.46 | 211 | 8.56 | 387 |
|  |  | 2008 | CS | | R | | MW | 9.8 | 110.0 | 14.8 | 143.0 | 8.2 | 180 | 60 | 120 | 5.82 | 7.15 | 7.52 | 7.48 | 181 | 7.60 | 379 |
|  | 38.3°N,  116.3°E | 2006 | CS | | R | | MW | 7.3 | 130.0 | 15.9 | 111.0 | 7.6 | 180 | 60 | 120 | 5.94 | 7.27 | 7.64 | 7.60 | 181 | 7.72 | 373 |
|  |  |  | CS | | R | | MW | 7.5 | 80.0 | 18.9 | 123.0 | 7.5 | 180 | 60 | 120 | 5.87 | 7.19 | 7.56 | 7.53 | 182 | 7.64 | 379 |
|  |  | 2007 | CS | | R | | MW | 19.8 | 120.0 | 16.5 | 129.0 | 6.5 | 180 | 60 | 120 | 7.23 | 8.53 | 8.81 | 8.89 | 185 | 8.94 | 328 |
|  |  | 2008 | BE | | R | | MW | 9.8 | 120.0 | 26.5 | 142.0 | 7.9 | 220 | 60 | 150 | 7.01 | 8.22 | 8.54 | 8.50 | 210 | 8.60 | 383 |
|  | 38.4°N,  116.3°E | 2009 | BE | | R | | MW | 9.6 | 110.0 | 15.9 | 147.0 | 8.1 | 180 | 60 | 120 | 6.18 | 7.49 | 7.86 | 7.83 | 181 | 7.95 | 362 |
|  |  |  | BE | | R | | MW | 8.8 | 140.0 | 18.9 | 156.0 | 7.5 | 220 | 60 | 150 | 7.49 | 8.78 | 9.11 | 9.00 | 206 | 9.16 | 353 |
|  |  | 2010 | FAS | | R | | MW | 8.9 | 90.0 | 31.4 | 152.0 | 8.4 | 180 | 60 | 120 | 5.83 | 7.17 | 7.54 | 7.48 | 180 | 7.62 | 376 |
|  |  |  | FAS | | R | | MW | 15.2 | 150.0 | 20.8 | 132.0 | 8.1 | 220 | 60 | 150 | 6.86 | 8.07 | 8.38 | 8.36 | 211 | 8.45 | 392 |
|  | 38.5°N,  116.3°E | 2007 | BE | | R | | MW | 8.6 | 130.0 | 17.8 | 127.0 | 7.8 | 220 | 60 | 150 | 7.02 | 8.36 | 8.69 | 8.55 | 204 | 8.74 | 367 |
|  |  | 2008 | BE | | R | | MW | 8.9 | 140.0 | 30.5 | 97.0 | 7.4 | 220 | 60 | 150 | 8.43 | 9.76 | 10.09 | 9.94 | 204 | 10.14 | 316 |
|  |  |  | CS | | R | | MW | 17.4 | 110.0 | 15.9 | 189.0 | 6.9 | 180 | 60 | 120 | 6.62 | 7.95 | 8.31 | 8.28 | 181 | 8.40 | 343 |
|  |  |  | CS | | R | | MW | 11.5 | 120.0 | 18.6 | 194.0 | 6.8 | 180 | 60 | 120 | 7.18 | 8.48 | 8.76 | 8.83 | 185 | 8.89 | 330 |
|  |  |  | CS | | I | | MW | 12.5 | 130.0 | 26.5 | 210.0 | 8.1 | 220 | 60 | 150 | 8.46 | 9.79 | 10.12 | 9.97 | 204 | 10.17 | 315 |
|  | 38.5°N,  116.4°E | 2009 | CS | | R | | MW | 12.4 | 130.0 | 15.6 | 114.0 | 7.5 | 220 | 60 | 150 | 7.47 | 8.70 | 9.06 | 8.99 | 210 | 9.11 | 362 |
|  |  |  | FAS | | R | | MW | 9.8 | 120.0 | 15.8 | 145.0 | 8.4 | 180 | 60 | 120 | 6.81 | 8.12 | 8.43 | 8.47 | 184 | 8.54 | 342 |
|  |  |  | FAS | | R | | MW | 8.9 | 130.0 | 16.4 | 187.0 | 7.8 | 180 | 60 | 120 | 6.58 | 7.89 | 8.20 | 8.23 | 183 | 8.32 | 349 |
|  | 38.5°N,  116.3°E | 2009 | FAS | | R | | MW | 16.1 | 170.0 | 13.5 | 145.0 | 6.8 | 220 | 60 | 150 | 7.62 | 8.67 | 8.99 | 8.93 | 204 | 9.02 | 356 |
|  |  |  | FAS | | R | | MW | 14.0 | 140.0 | 12.8 | 125.0 | 6.8 | 180 | 60 | 120 | 6.45 | 7.80 | 8.15 | 8.10 | 179 | 8.24 | 346 |
|  |  |  | FAS | | R | | MW | 16.8 | 130.0 | 15.7 | 178.0 | 7.6 | 180 | 60 | 120 | 7.31 | 8.67 | 9.01 | 8.96 | 179 | 9.10 | 313 |
|  | 38.5°N,  116.2°E | 2006 | FAS | | R | | MW | 16.0 | 170.0 | 34.5 | 69.0 | 7.5 | 220 | 60 | 150 | 8.38 | 9.73 | 10.06 | 9.91 | 204 | 10.11 | 317 |
|  |  | 2007 | FAS | | R | | MW | 7.7 | 100.0 | 15.9 | 177.0 | 7.9 | 180 | 60 | 120 | 6.73 | 8.03 | 8.30 | 8.38 | 185 | 8.44 | 348 |
|  |  |  | FAS | | R | | MW | 15.7 | 160.0 | 16.8 | 124.0 | 8.1 | 220 | 60 | 150 | 7.01 | 8.22 | 8.53 | 8.51 | 211 | 8.60 | 385 |
|  |  |  | FAS | | R | | MW | 8.9 | 140.0 | 15.5 | 125.0 | 8.3 | 180 | 60 | 120 | 6.10 | 7.46 | 7.80 | 7.75 | 179 | 7.89 | 361 |
| Boye,  Hebei | 38.3°N,  115.2°E | 2009 | FAS | | I | | MW | 13.0 | 89.0 | 16.5 | 74.0 | 6.8 | 220 | 60 | 150 | 7.49 | 8.71 | 8.45 | 8.46 | 172 | 8.67 | 318 |
|  |  |  | BE | | R | | MW | 5.9 | 127.0 | 36.5 | 96.0 | 7.8 | 220 | 60 | 150 | 6.62 | 8.82 | 8.45 | 7.42 | 163 | 8.84 | 298 |
|  |  |  | BE | | I | | MW | 15.6 | 96.0 | 15.6 | 135.0 | 8.1 | 220 | 60 | 150 | 4.72 | 8.20 | 8.88 | 8.41 | 218 | 9.12 | 375 |
|  |  |  | BE | | R | | MW | 8.9 | 73.0 | 20.4 | 142.0 | 7.5 | 220 | 60 | 150 | 7.75 | 8.91 | 9.30 | 9.39 | 228 | 9.39 | 381 |
| Dongguang,  Hebei | 37.5°N,  116.3°E | 2007 | BE | | R | | MW | 9.6 | 150.0 | 8.9 | 89.0 | 6.7 | 160 | 60 | 90 | 5.26 | 6.09 | 6.76 | 6.85 | 201 | 6.83 | 463 |
|  |  | 2008 | BE | | R | | MW | 8.9 | 110.0 | 15.8 | 151.0 | 7.8 | 160 | 60 | 90 | 5.39 | 6.88 | 7.19 | 7.13 | 160 | 7.31 | 355 |
|  |  | 2009 | CS | | R | | MW | 6.3 | 104.0 | 15.1 | 210.0 | 7.5 | 160 | 60 | 90 | 5.36 | 6.42 | 6.93 | 6.75 | 159 | 6.91 | 373 |
|  | 37.6°N,  116.3°E | 2009 | CS | | R | | MW | 8.9 | 165.0 | 16.0 | 125.0 | 7.4 | 220 | 60 | 150 | 7.33 | 7.62 | 8.51 | 8.10 | 195 | 8.18 | 376 |
|  |  |  | CS | | R | | MW | 12.6 | 223.0 | 21.4 | 124.0 | 7.6 | 220 | 60 | 150 | 6.32 | 8.54 | 8.77 | 7.76 | 181 | 8.91 | 323 |
|  |  | 2010 | BE | | R | | MW | 10.3 | 200.0 | 16.5 | 89.0 | 8.5 | 220 | 60 | 150 | 6.31 | 7.16 | 7.55 | 7.72 | 240 | 7.63 | 494 |
|  | 37.6°N,  116.4°E | 2007 | BE | | R | | MW | 8.9 | 128.0 | 20.3 | 84.0 | 7.5 | 220 | 60 | 150 | 6.40 | 7.45 | 7.82 | 7.59 | 194 | 7.80 | 393 |
|  |  | 2008 | BE | | R | | MW | 10.1 | 187.0 | 31.5 | 124.0 | 7.4 | 180 | 60 | 120 | 5.46 | 6.13 | 6.81 | 6.12 | 146 | 6.58 | 366 |
|  |  |  | BE | | R | | MW | 9.9 | 118.0 | 15.9 | 119.0 | 6.8 | 180 | 60 | 120 | 5.04 | 6.81 | 7.77 | 7.81 | 212 | 7.88 | 422 |
|  |  | 2010 | CS | | R | | MW | 6.5 | 114.0 | 33.2 | 115.0 | 6.5 | 180 | 60 | 120 | 6.31 | 6.71 | 7.55 | 7.40 | 209 | 7.41 | 443 |
|  |  |  | CS | | R | | MW | 9.7 | 112.0 | 15.6 | 116.0 | 8.4 | 160 | 60 | 90 | 4.73 | 5.71 | 6.39 | 6.43 | 191 | 6.43 | 470 |
| Gaocheng,  Hebei | 38.0°N,  114.5°E | 2006 | CS | | R | | MW | 28.8 | 136.0 | 14.0 | 157.0 | 7.4 | 220 | 60 | 150 | 7.00 | 7.26 | 7.95 | 7.60 | 173 | 7.66 | 361 |
|  |  | 2007 | Solon. | | R | | MW | 11.0 | 82.0 | 9.8 | 113.0 | 6.8 | 180 | 150 | 120 | 7.97 | 9.01 | 9.45 | 9.51 | 194 | 9.52 | 322 |
|  |  |  | Solon. | | R | | MW | 15.2 | 110.0 | 9.6 | 115.0 | 6.7 | 220 | 60 | 150 | 6.02 | 7.35 | 7.83 | 7.68 | 213 | 7.85 | 426 |
|  | 38.0°N,  114.6°E | 2006 | Solon. | | R | | MW | 21.3 | 114.0 | 20.1 | 107.0 | 7.3 | 180 | 150 | 120 | 6.52 | 6.89 | 7.92 | 7.48 | 181 | 7.60 | 379 |
|  |  |  | BE | | R | | MW | 58.3 | 70.0 | 37.8 | 128.0 | 7.6 | 220 | 60 | 150 | 7.05 | 7.35 | 8.24 | 7.78 | 188 | 7.90 | 377 |
|  |  | 2007 | CS | | R | | MW | 15.5 | 122.0 | 8.8 | 70.0 | 8.1 | 220 | 60 | 150 | 6.57 | 7.32 | 7.89 | 7.43 | 181 | 7.72 | 373 |
|  |  |  | FAS | | R | | MW | 14.6 | 114.0 | 14.0 | 71.0 | 8.2 | 220 | 60 | 150 | 6.25 | 7.08 | 8.19 | 7.65 | 214 | 7.88 | 426 |
|  | 37.6°N,  114.6°E | 2006 | FAS | | R | | MW | 21.8 | 152.0 | 38.7 | 110.0 | 7.5 | 180 | 150 | 120 | 6.55 | 7.20 | 8.31 | 7.17 | 146 | 7.90 | 305 |
|  |  |  | FAS | | R | | MW | 22.6 | 84.0 | 17.8 | 150.0 | 7.9 | 180 | 150 | 120 | 6.46 | 6.80 | 7.86 | 7.57 | 209 | 7.62 | 431 |
|  |  |  | FAS | | R | | MW | 16.0 | 106.0 | 14.5 | 145.0 | 7.8 | 180 | 150 | 120 | 6.09 | 6.55 | 7.91 | 7.56 | 222 | 7.65 | 455 |
|  | 37.6°N,  114.5°E | 2007 | FAS | | R | | MW | 11.2 | 110.0 | 19.8 | 108.0 | 7.5 | 180 | 150 | 120 | 7.97 | 9.01 | 9.45 | 9.51 | 194 | 9.52 | 322 |
|  |  |  | FAS | | R | | MW | 13.1 | 154.0 | 29.5 | 157.0 | 8.2 | 220 | 60 | 150 | 6.02 | 7.35 | 7.83 | 7.68 | 213 | 7.85 | 426 |
|  |  | 2009 | FAS | | R | | MW | 20.5 | 118.0 | 49.0 | 160.0 | 7.6 | 220 | 60 | 150 | 9.67 | 10.28 | 10.73 | 10.46 | 179 | 10.59 | 269 |
|  |  |  | FAS | | R | | MW | 17.8 | 57.0 | 23.0 | 77.0 | 8.1 | 180 | 150 | 120 | 7.72 | 8.16 | 8.64 | 8.51 | 166 | 8.52 | 314 |
|  | 37.7°N,  114.5°E | 2008 | FAS | | R | | MW | 18.6 | 115.0 | 30.8 | 156.0 | 8.0 | 180 | 150 | 120 | 8.40 | 9.02 | 9.21 | 8.87 | 131 | 9.17 | 242 |
|  |  |  | FAS | | R | | MW | 8.3 | 127.0 | 33.5 | 69.0 | 7.5 | 220 | 60 | 150 | 7.53 | 8.42 | 9.09 | 8.77 | 204 | 8.95 | 358 |
|  |  | 2009 | BE | | R | | MW | 13.4 | 140.0 | 9.6 | 154.0 | 8.0 | 220 | 60 | 150 | 7.00 | 7.26 | 7.95 | 7.60 | 173 | 7.66 | 361 |
|  |  |  | BE | | R | | MW | 14.5 | 120.0 | 15.4 | 113.0 | 7.4 | 180 | 150 | 120 | 6.52 | 6.89 | 7.92 | 7.48 | 181 | 7.60 | 379 |
|  | 37.7°N,  114.6°E | 2010 | PS | | R | | MW | 13.4 | 170.0 | 14.5 | 123.0 | 6.7 | 220 | 60 | 150 | 7.05 | 7.35 | 8.24 | 7.78 | 188 | 7.90 | 377 |
|  |  |  | PS | | R | | MW | 14.2 | 120.0 | 9.8 | 180.0 | 6.8 | 180 | 150 | 120 | 8.40 | 9.02 | 9.21 | 8.87 | 131 | 9.17 | 242 |
|  |  |  | PS | | R | | MW | 18.5 | 140.0 | 8.9 | 195.0 | 6.5 | 220 | 60 | 150 | 7.53 | 8.42 | 9.09 | 8.77 | 204 | 8.95 | 358 |
|  | 37.8°N,  114.6°E | 2006 | PS | | R | | MW | 12.2 | 123.0 | 17.8 | 124.0 | 5.2 | 220 | 60 | 150 | 6.57 | 7.32 | 7.89 | 7.43 | 181 | 7.72 | 373 |
|  |  | 2007 | PS | | I | | MW | 11.6 | 110.0 | 8.9 | 125.0 | 6.5 | 220 | 60 | 150 | 6.25 | 7.08 | 8.19 | 7.65 | 214 | 7.88 | 426 |
|  |  | 2008 | PS | | R | | MW | 13.0 | 60.0 | 12.6 | 113.0 | 8.1 | 180 | 150 | 120 | 6.55 | 7.20 | 8.31 | 7.17 | 146 | 7.90 | 305 |
|  | 37.9°N,  114.6°E | 2006 | PS | | R | | MW | 11.0 | 170.0 | 15.6 | 125.0 | 6.9 | 180 | 150 | 120 | 6.46 | 6.80 | 7.86 | 7.57 | 209 | 7.62 | 431 |
|  |  | 2007 | PS | | R | | MW | 13.7 | 90.0 | 13.9 | 145.0 | 7.8 | 180 | 150 | 120 | 6.09 | 6.55 | 7.91 | 7.56 | 222 | 7.65 | 455 |
|  |  | 2008 | YE | | R | | MW | 11.0 | 100.0 | 17.8 | 141.0 | 7.5 | 220 | 60 | 150 | 9.67 | 10.28 | 10.73 | 10.46 | 179 | 10.59 | 269 |
|  |  | 2009 | YB | | R | | MW | 10.0 | 70.0 | 18.7 | 89.0 | 7.8 | 180 | 150 | 120 | 7.72 | 8.16 | 8.64 | 8.51 | 166 | 8.52 | 314 |
| Jizhou,  Hebei | 37.3°N,  115.3°E | 2005 | CS | | R | | MW | 17.3 | 95.0 | 23.0 | 82.0 | 8.2 | 220 | 60 | 150 | 8.39 | 9.43 | 10.02 | 8.36 | 151 | 9.88 | 251 |
|  |  |  | CS | | R | | MW | 16.8 | 90.0 | 21.0 | 79.0 | 7.6 | 220 | 60 | 150 | 9.25 | 9.85 | 10.79 | 10.28 | 200 | 10.49 | 300 |
|  | 37.3°N,  115.4°E | 2006 | CS | | R | | MW | 25.9 | 146.0 | 12.0 | 56.0 | 8.1 | 220 | 60 | 150 | 6.46 | 9.00 | 9.19 | 9.09 | 214 | 9.49 | 354 |
|  |  |  | CS | | R | | MW | 14.8 | 76.0 | 20.0 | 80.0 | 8.0 | 220 | 60 | 150 | 8.25 | 9.47 | 9.62 | 8.72 | 158 | 9.67 | 265 |
|  |  | 2007 | CS | | R | | MW | 12.2 | 61.0 | 36.0 | 130.0 | 7.4 | 220 | 60 | 150 | 8.41 | 9.39 | 9.75 | 9.00 | 163 | 9.67 | 272 |
|  | 37.4°N,  115.4°E | 2007 | CS | | R | | MW | 21.1 | 97.0 | 32.0 | 70.0 | 6.7 | 220 | 60 | 150 | 7.61 | 10.14 | 10.30 | 9.61 | 192 | 10.52 | 288 |
|  |  | 2009 | CS | | R | | MW | 16.9 | 89.0 | 27.6 | 108.0 | 5.2 | 220 | 60 | 150 | 9.05 | 9.99 | 10.30 | 9.90 | 175 | 10.25 | 273 |
|  |  |  | CS | | R | | MW | 16.9 | 88.0 | 25.6 | 104.0 | 6.5 | 220 | 60 | 150 | 7.70 | 9.45 | 10.47 | 7.93 | 162 | 10.23 | 256 |
|  | 37.4°N,  115.3°E | 2009 | CS | | R | | MW | 18.6 | 100.0 | 15.8 | 87.0 | 6.2 | 220 | 60 | 150 | 7.31 | 8.68 | 9.90 | 8.57 | 187 | 9.53 | 311 |
|  |  |  | CS | | R | | MW | 12.0 | 93.2 | 17.8 | 200.0 | 8.2 | 220 | 60 | 150 | 7.17 | 8.85 | 9.30 | 8.42 | 182 | 9.29 | 311 |
|  |  |  | CS | | R | | MW | 22.0 | 113.0 | 14.2 | 154.0 | 7.6 | 220 | 60 | 150 | 7.31 | 8.68 | 9.90 | 8.57 | 187 | 9.53 | 311 |
| Lincheng,  Hebei | 37.2°N,  114.3°E | 2007 | CS | | R | | MW | 9.0 | 104.0 | 15.6 | 201.0 | 7.7 | 180 | 60 | 120 | 7.92 | 8.81 | 9.03 | 8.96 | 163 | 9.06 | 291 |
|  |  | 2008 | CS | | R | | MW | 10.5 | 106.0 | 18.4 | 158.0 | 6.9 | 180 | 60 | 120 | 6.00 | 8.21 | 8.50 | 9.16 | 228 | 9.05 | 395 |
|  |  | 2009 | FAS | | R | | MW | 9.6 | 106.0 | 20.1 | 98.0 | 8.0 | 150 | 60 | 90 | 6.27 | 7.27 | 7.84 | 7.98 | 185 | 7.96 | 369 |
|  |  | 2010 | FAS | | I | | MW | 14.5 | 98.0 | 22.0 | 115.0 | 7.5 | 150 | 60 | 90 | 5.84 | 7.13 | 7.41 | 7.55 | 163 | 7.58 | 347 |
|  |  |  | FAS | | R | | MW | 13.6 | 154.0 | 16.6 | 165.0 | 7.4 | 180 | 60 | 120 | 6.70 | 7.34 | 7.54 | 7.77 | 205 | 7.67 | 420 |
|  |  |  | FAS | | R | | MW | 14.5 | 124.0 | 15.6 | 174.0 | 6.9 | 150 | 60 | 90 | 5.40 | 5.82 | 7.07 | 6.50 | 158 | 6.69 | 384 |
| Luancheng,  Hebei | 37.5°N,  114.4°E | 2009 | YE | | R | | MW | 16.2 | 216.0 | 12.0 | 140.0 | 8.1 | 180 | 60 | 120 | 8.49 | 9.81 | 10.02 | 9.81 | 162 | 10.10 | 259 |
|  |  |  | YE | | R | | MW | 9.8 | 158.0 | 15.6 | 135.0 | 6.9 | 220 | 60 | 150 | 8.21 | 8.60 | 8.94 | 8.72 | 151 | 8.78 | 282 |
|  |  |  | YE | | R | | MW | 12.6 | 110.0 | 15.4 | 152.0 | 6.7 | 180 | 60 | 120 | 4.40 | 5.51 | 6.06 | 5.43 | 154 | 5.95 | 423 |
|  | 37.6°N,  114.5°E | 2009 | LS | | R | | MW | 13.9 | 143.0 | 21.1 | 115.0 | 7.9 | 180 | 60 | 120 | 6.30 | 7.64 | 8.16 | 8.22 | 198 | 8.26 | 378 |
|  |  |  | YE | | R | | MW | 9.8 | 109.0 | 19.8 | 102.0 | 8.1 | 180 | 60 | 120 | 5.78 | 7.29 | 8.22 | 8.04 | 197 | 8.19 | 379 |
|  |  | 2010 | CS | | R | | MW | 16.2 | 216.0 | 16.4 | 125.0 | 6.9 | 180 | 60 | 120 | 5.37 | 7.08 | 8.51 | 8.60 | 238 | 8.63 | 433 |
|  |  |  | CS | | I | | MW | 17.1 | 133.0 | 15.5 | 98.0 | 7.6 | 180 | 60 | 120 | 5.48 | 6.77 | 6.63 | 7.11 | 200 | 6.99 | 451 |
|  |  |  | FAS | | R | | MW | 9.7 | 117.0 | 9.5 | 66.0 | 6.8 | 220 | 60 | 150 | 7.62 | 10.10 | 10.92 | 10.55 | 222 | 10.99 | 317 |
| Luquan,  Hebei | 38.0°N,  114.2°E | 2008 | CS | | R | | MW | 18.0 | 121.0 | 15.4 | 156.0 | 8.1 | 180 | 60 | 120 | 4.95 | 5.64 | 5.70 | 5.60 | 138 | 5.73 | 403 |
|  |  |  | CS | | R | | MW | 16.8 | 145.0 | 18.9 | 160.0 | 8.1 | 220 | 60 | 150 | 6.80 | 7.17 | 7.14 | 6.99 | 94 | 7.10 | 251 |
|  | 38.1°N,  114.2°E | 2010 | FAS | | R | | MW | 17.4 | 163.0 | 20.5 | 154.0 | 8.0 | 180 | 30 | 60 | 4.87 | 5.64 | 5.97 | 5.02 | 124 | 5.90 | 362 |
|  |  |  | PS | | R | | MW | 18.9 | 147.0 | 21.4 | 153.0 | 7.9 | 195 | 15 | 60 | 5.46 | 5.85 | 6.01 | 5.54 | 110 | 5.94 | 331 |
|  |  |  | YE | | R | | MW | 20.1 | 112.0 | 22.6 | 135.0 | 8.2 | 180 | 30 | 60 | 5.37 | 5.91 | 6.21 | 5.96 | 140 | 6.12 | 381 |
| Pingshan,  Hebei | 38.2°N,  113.6°E | 2008 | YB | | R | | MW | 12.2 | 70.0 | 14.5 | 56.0 | 7.6 | 220 | 120 | 150 | 7.58 | 7.78 | 8.64 | 8.39 | 216 | 8.37 | 405 |
|  |  |  | FAS | | R | | MW | 11.4 | 71.0 | 17.8 | 123.0 | 6.8 | 210 | 135 | 120 | 7.58 | 7.78 | 8.64 | 8.39 | 211 | 8.38 | 395 |
| Qingyuan,  Hebei | 39.4°N,  115.2°E | 2006 | FAS | | R | | MW | 15.2 | 59.0 | 24.7 | 124.0 | 7.6 | 180 | 60 | 120 | 5.47 | 7.86 | 8.15 | 8.15 | 184 | 8.43 | 346 |
|  |  |  | YE | | I | | MW | 14.0 | 148.0 | 24.5 | 135.0 | 7.9 | 220 | 60 | 150 | 3.46 | 8.49 | 7.33 | 8.20 | 222 | 8.57 | 406 |
|  |  | 2007 | FAS | | R | | MW | 16.0 | 84.0 | 15.9 | 114.0 | 7.5 | 220 | 60 | 150 | 6.76 | 7.84 | 8.71 | 7.49 | 173 | 8.43 | 328 |
|  |  |  | PS | | R | | MW | 11.4 | 117.0 | 18.9 | 124.0 | 7.1 | 180 | 60 | 120 | 5.47 | 7.86 | 7.55 | 8.15 | 195 | 8.13 | 378 |
|  | 39.4°N,  115.3°E | 2005 | YE | | R | | MW | 21.0 | 90.0 | 9.9 | 126.0 | 7.0 | 180 | 60 | 120 | 6.24 | 8.24 | 8.26 | 8.51 | 186 | 8.60 | 343 |
|  |  |  | YB | | R | | MW | 9.9 | 75.0 | 26.9 | 128.0 | 6.8 | 180 | 60 | 120 | 5.47 | 7.86 | 8.15 | 8.15 | 184 | 8.43 | 346 |
|  |  | 2006 | PS | | R | | MW | 10.5 | 68.0 | 15.4 | 127.0 | 6.9 | 220 | 60 | 150 | 3.46 | 8.49 | 7.33 | 8.20 | 222 | 8.57 | 406 |
|  |  |  | PS | | R | | MW | 11.5 | 89.0 | 19.8 | 115.0 | 8.0 | 220 | 60 | 150 | 6.76 | 7.84 | 8.71 | 7.49 | 173 | 8.43 | 328 |
|  | 39.4°N,  115.4°E | 2007 | YE | | R | | MW | 12.6 | 107.0 | 24.7 | 113.0 | 6.7 | 160 | 60 | 90 | 4.58 | 7.45 | 8.25 | 8.45 | 185 | 8.58 | 342 |
|  |  | 2009 | YE | | R | | MW | 15.6 | 80.0 | 36.5 | 126.0 | 8.0 | 180 | 60 | 120 | 5.47 | 7.86 | 7.55 | 8.15 | 195 | 8.13 | 378 |
|  |  |  | YE | | R | | MW | 22.2 | 91.0 | 14.6 | 145.0 | 7.5 | 180 | 60 | 120 | 5.48 | 7.94 | 7.55 | 8.16 | 192 | 8.16 | 372 |
|  |  | 2010 | PS | | R | | MW | 18.2 | 102.0 | 16.6 | 171.0 | 7.6 | 180 | 60 | 120 | 6.27 | 8.40 | 8.27 | 8.52 | 180 | 8.67 | 330 |
|  |  |  | PS | | R | | MW | 19.7 | 116.0 | 15.6 | 111.0 | 7.7 | 180 | 60 | 120 | 5.49 | 7.87 | 8.16 | 8.16 | 184 | 8.44 | 346 |
|  | 39.5°N,  115.4°E | 2009 | YE | | R | | MW | 14.9 | 57.0 | 16.9 | 115.0 | 6.6 | 220 | 60 | 150 | 3.49 | 8.53 | 7.34 | 8.21 | 221 | 8.58 | 404 |
|  |  |  | PS | | R | | MP | 17.3 | 132.0 | 15.9 | 119.0 | 6.8 | 220 | 60 | 150 | 6.94 | 7.90 | 8.72 | 7.49 | 167 | 8.45 | 318 |
|  |  | 2010 | RE | | R | | MW | 14.5 | 272.0 | 17.8 | 159.0 | 7.4 | 180 | 60 | 120 | 5.47 | 7.86 | 7.55 | 8.16 | 195 | 8.14 | 378 |
|  |  |  | DLS | | R | | MW | 16.2 | 119.0 | 19.5 | 142.0 | 7.5 | 180 | 60 | 120 | 6.26 | 8.25 | 8.27 | 8.52 | 186 | 8.61 | 342 |
|  | 39.5°N,  115.3°E | 2008 | CS | | I | | MW | 18.4 | 135.0 | 15.4 | 156.0 | 7.9 | 180 | 60 | 120 | 5.47 | 7.86 | 8.16 | 8.16 | 184 | 8.44 | 346 |
|  |  |  | FAS | | R | | MW | 12.5 | 153.0 | 34.2 | 135.0 | 7.6 | 220 | 60 | 150 | 3.47 | 8.50 | 7.34 | 8.21 | 222 | 8.57 | 406 |
|  |  |  | LCFS | | R | | MW | 13.6 | 137.0 | 20.5 | 96.0 | 8.1 | 220 | 60 | 150 | 6.77 | 7.85 | 8.72 | 7.49 | 172 | 8.44 | 326 |
|  |  | 2009 | FAS | | R | | MW | 14.2 | 101.0 | 20.4 | 126.0 | 7.4 | 160 | 60 | 90 | 3.89 | 4.79 | 6.01 | 5.29 | 157 | 5.67 | 450 |
|  |  |  | FAS | | R | | MW | 11.3 | 72.0 | 9.6 | 157.0 | 8.1 | 180 | 60 | 120 | 5.39 | 6.21 | 7.06 | 6.63 | 174 | 6.85 | 406 |
|  | 39.5°N,  115.2°E | 2005 | FAS | | R | | MW | 9.8 | 88.0 | 21.4 | 114.0 | 6.9 | 220 | 60 | 150 | 5.36 | 6.27 | 6.91 | 6.48 | 193 | 6.75 | 452 |
|  |  |  | FAS | | R | | MW | 10.6 | 98.0 | 21.3 | 161.0 | 8.1 | 220 | 60 | 150 | 5.37 | 6.28 | 7.11 | 6.74 | 211 | 6.93 | 478 |
|  |  | 2006 | CS | | R | | MW | 11.5 | 92.0 | 18.9 | 159.0 | 7.5 | 180 | 60 | 120 | 5.19 | 5.93 | 6.79 | 6.29 | 168 | 6.55 | 412 |
|  |  |  | CS | | R | | MW | 12.1 | 148.0 | 16.9 | 115.0 | 7.4 | 220 | 60 | 150 | 6.06 | 6.85 | 7.96 | 6.96 | 184 | 7.57 | 386 |
|  |  | 2007 | CS | | R | | MW | 12.9 | 123.0 | 18.4 | 115.0 | 7.6 | 220 | 60 | 150 | 5.98 | 7.01 | 8.27 | 7.40 | 203 | 7.89 | 405 |
|  |  |  | DLS | | R | | MW | 11.5 | 79.0 | 20.5 | 132.0 | 8.1 | 160 | 60 | 90 | 3.77 | 4.82 | 5.85 | 5.13 | 151 | 5.58 | 444 |
|  |  |  | CS | | R | | MW | 10.5 | 83.0 | 21.4 | 153.0 | 6.9 | 180 | 60 | 120 | 6.24 | 8.24 | 8.26 | 8.51 | 186 | 8.60 | 343 |
| Rongcheng,  Hebei | 39.0°N,  115.5°E | 2009 | FAS | | I | | MW | 9.9 | 140.0 | 14.9 | 210.0 | 7.6 | 220 | 60 | 150 | 7.43 | 8.64 | 8.61 | 8.31 | 170 | 8.73 | 312 |
|  |  |  | LCFS | | R | | MW | 8.9 | 70.0 | 20.8 | 85.0 | 6.9 | 180 | 60 | 120 | 5.32 | 6.53 | 6.78 | 7.00 | 198 | 6.97 | 448 |
| Sanhe,  Hebei | 39.6°N,  116.6°E | 2006 | LCFS | | R | | MW | 20.1 | 125.0 | 20.1 | 125.0 | 8.1 | 180 | 60 | 120 | 6.92 | 7.56 | 7.42 | 7.30 | 115 | 7.51 | 270 |
|  |  | 2007 | LCFS | | R | | MW | 8.9 | 114.0 | 9.8 | 126.0 | 6.5 | 180 | 60 | 120 | 6.86 | 8.26 | 8.50 | 8.73 | 197 | 8.71 | 357 |
|  |  | 2008 | LCFS | | R | | MW | 15.4 | 104.0 | 15.5 | 196.0 | 7.5 | 180 | 60 | 120 | 4.35 | 5.41 | 6.42 | 4.86 | 140 | 6.09 | 383 |
|  | 39.6°N,  117.0°E | 2007 | MS | | R | | MW | 16.5 | 114.0 | 18.9 | 134.0 | 8.2 | 180 | 60 | 120 | 6.86 | 8.26 | 8.50 | 8.73 | 197 | 8.71 | 357 |
|  |  | 2008 | MS | | R | | MW | 18.4 | 104.0 | 14.5 | 125.0 | 7.6 | 180 | 60 | 120 | 4.35 | 5.41 | 6.42 | 4.86 | 140 | 6.09 | 383 |
|  |  | 2009 | MS | | R | | MW | 19.5 | 91.0 | 9.8 | 134.0 | 8.1 | 180 | 60 | 120 | 7.15 | 8.17 | 8.54 | 8.76 | 210 | 8.70 | 379 |
| Shunping,  Hebei | 38.5°N,  115.0°E | 2005 | CS | | R | | MW | 40.9 | 215.0 | 19.1 | 145.0 | 7.8 | 220 | 105 | 105 | 6.40 | 7.40 | 7.80 | 7.51 | 190 | 7.75 | 388 |
|  |  |  |  | | R | | MW | 22.2 | 198.0 | 14.5 | 198.0 | 7.8 | 220 | 105 | 105 | 7.16 | 8.49 | 9.18 | 8.08 | 176 | 9.02 | 311 |
|  |  |  | BE | | R | | MW | 44.0 | 214.0 | 48.4 | 175.0 | 7.7 | 220 | 105 | 105 | 6.71 | 8.79 | 8.94 | 7.81 | 173 | 9.09 | 305 |
|  |  | 2006 | CS | | R | | MW | 33.6 | 255.0 | 17.9 | 194.0 | 8.0 | 220 | 105 | 105 | 6.63 | 8.09 | 9.15 | 6.51 | 157 | 8.87 | 288 |
|  |  |  | FAS | | R | | MW | 35.6 | 193.0 | 21.1 | 171.0 | 8.2 | 220 | 105 | 105 | 7.67 | 8.90 | 9.02 | 9.00 | 197 | 9.13 | 340 |
|  |  |  | FAS | | R | | MW | 28.8 | 126.0 | 9.7 | 184.0 | 8.1 | 220 | 105 | 105 | 6.50 | 8.58 | 8.64 | 9.07 | 238 | 9.05 | 413 |
|  |  | 2007 | BE | | R | | MP | 28.5 | 84.0 | 18.0 | 179.0 | 8.3 | 220 | 105 | 105 | 7.09 | 9.26 | 9.17 | 9.14 | 203 | 9.50 | 336 |
|  |  |  | CS | | R | | MP | 27.9 | 94.5 | 16.6 | 121.0 | 6.2 | 220 | 105 | 105 | 7.52 | 8.92 | 9.64 | 8.49 | 177 | 9.47 | 298 |
|  |  | 2008 | FAS | | R | | MW | 25.3 | 126.0 | 17.8 | 156.0 | 6.5 | 220 | 105 | 105 | 6.97 | 8.50 | 9.60 | 6.84 | 157 | 9.31 | 274 |
|  |  |  | CS | | R | | MW | 14.6 | 85.0 | 9.6 | 201.0 | 7.2 | 220 | 105 | 105 | 7.52 | 8.92 | 9.64 | 8.49 | 177 | 9.47 | 298 |
|  | 38.5°N,  115.1°E | 2006 | FAS | | R | | MW | 12.9 | 156.0 | 9.8 | 141.0 | 8.0 | 220 | 105 | 105 | 6.97 | 8.50 | 9.60 | 6.84 | 157 | 9.31 | 274 |
|  |  |  | FAS | | R | | MW | 16.5 | 280.0 | 9.5 | 161.0 | 6.9 | 220 | 105 | 105 | 8.05 | 9.35 | 9.47 | 9.45 | 199 | 9.59 | 327 |
|  |  | 2007 | BE | | R | | MW | 11.5 | 203.0 | 20.0 | 124.0 | 6.8 | 220 | 105 | 105 | 7.44 | 9.73 | 9.62 | 9.60 | 204 | 9.97 | 322 |
|  |  |  | CS | | R | | MW | 25.9 | 87.0 | 13.6 | 162.0 | 6.4 | 220 | 105 | 105 | 8.05 | 9.35 | 9.47 | 9.45 | 199 | 9.59 | 327 |
|  |  |  | FAS | | R | | MW | 19.0 | 176.0 | 30.6 | 122.0 | 5.6 | 220 | 105 | 105 | 7.44 | 9.73 | 9.62 | 9.60 | 204 | 9.97 | 322 |
|  |  | 2008 | FAS | | R | | MW | 8.8 | 99.0 | 9.6 | 151.0 | 6.5 | 220 | 105 | 105 | 6.40 | 7.40 | 7.80 | 7.51 | 190 | 7.75 | 388 |
|  |  |  | CS | | R | | MW | 7.2 | 79.0 | 20.5 | 141.0 | 7.5 | 220 | 105 | 105 | 7.16 | 8.49 | 9.18 | 8.08 | 176 | 9.02 | 311 |
|  |  |  | LS | | R | | MW | 8.4 | 68.0 | 19.8 | 123.0 | 7.2 | 220 | 105 | 105 | 6.71 | 8.79 | 8.94 | 7.81 | 173 | 9.09 | 305 |
|  |  | 2009 | YB | | R | | MW | 9.2 | 94.0 | 26.5 | 126.0 | 7.6 | 220 | 105 | 105 | 6.63 | 8.09 | 9.15 | 6.51 | 157 | 8.87 | 288 |
|  |  |  | LS | | R | | MW | 12.0 | 89.0 | 19.8 | 125.0 | 7.4 | 220 | 105 | 105 | 7.67 | 8.90 | 9.02 | 9.00 | 197 | 9.13 | 340 |
|  |  | 2010 | YE | | R | | MW | 11.6 | 78.0 | 15.8 | 125.0 | 7.8 | 220 | 105 | 105 | 6.50 | 8.58 | 8.64 | 9.07 | 238 | 9.05 | 413 |
|  |  |  | YE | | R | | MW | 14.0 | 79.0 | 29.6 | 122.0 | 8.1 | 220 | 105 | 105 | 7.09 | 9.26 | 9.17 | 9.14 | 203 | 9.50 | 336 |
| Tangxian,  Hebei | 38.4°N,  114.5°E | 2007 | YE | | R | | MW | 17.8 | 81.0 | 33.6 | 98.0 | 6.9 | 220 | 60 | 150 | 5.19 | 5.77 | 6.48 | 5.81 | 170 | 6.21 | 439 |
|  |  |  | YE | | R | | MW | 16.8 | 66.0 | 31.5 | 89.0 | 8.4 | 220 | 60 | 150 | 4.94 | 6.68 | 7.05 | 6.05 | 177 | 7.07 | 399 |
|  |  |  | YE | | R | | MW | 18.4 | 60.0 | 15.4 | 125.0 | 8.5 | 220 | 60 | 150 | 4.72 | 7.22 | 6.99 | 5.96 | 173 | 7.35 | 377 |
|  | 38.4°N,  114.6°E | 2008 | YE | | R | | MW | 9.8 | 80.0 | 12.5 | 121.0 | 7.6 | 180 | 60 | 120 | 5.60 | 6.84 | 6.68 | 6.88 | 167 | 6.92 | 388 |
|  |  |  | LS | | R | | MW | 11.5 | 89.0 | 9.9 | 157.0 | 7.8 | 180 | 60 | 120 | 5.20 | 5.79 | 6.49 | 5.82 | 145 | 6.24 | 384 |
|  |  |  | YE | | I | | MW | 16.8 | 78.0 | 27.8 | 98.0 | 7.5 | 180 | 60 | 120 | 5.12 | 5.70 | 6.41 | 5.74 | 145 | 6.16 | 389 |
| Xinji,  Hebei | 37.5°N,  115.2°E | 2007 | YE | | R | | MW | 14.0 | 150.0 | 15.4 | 115.0 | 6.5 | 220 | 60 | 150 | 4.70 | 6.00 | 5.90 | 5.50 | 166 | 6.06 | 441 |
|  |  | 2008 | YE | | R | | MW | 12.9 | 220.0 | 23.4 | 98.0 | 7.9 | 220 | 60 | 150 | 8.85 | 9.30 | 9.40 | 9.13 | 125 | 9.34 | 230 |
|  |  | 2009 | RE | | R | | MP | 8.9 | 150.0 | 22.6 | 115.0 | 6.9 | 180 | 60 | 120 | 8.26 | 10.06 | 10.89 | 11.04 | 215 | 11.07 | 305 |
|  | 37.5°N,  115.1°E | 2005 | RE | | I | | MW | 11.0 | 130.0 | 20.5 | 125.0 | 7.1 | 180 | 60 | 120 | 8.32 | 9.60 | 10.34 | 10.20 | 192 | 10.32 | 294 |
|  |  | 2006 | PS | | R | | MW | 18.9 | 120.0 | 21.4 | 120.0 | 6.5 | 180 | 60 | 120 | 7.17 | 8.85 | 9.63 | 9.15 | 176 | 9.57 | 294 |
|  |  | 2007 | PS | | R | | MW | 11.9 | 110.0 | 18.9 | 124.0 | 6.4 | 160 | 60 | 90 | 7.32 | 8.39 | 9.51 | 9.19 | 179 | 9.34 | 305 |
|  |  | 2008 | PS | | R | | MW | 19.8 | 150.0 | 22.5 | 145.0 | 7.5 | 220 | 60 | 150 | 4.70 | 6.00 | 5.90 | 5.50 | 166 | 6.06 | 441 |
|  | 38.2°N,  114.4°E | 2009 | YB | | R | | MW | 10.6 | 38.5 | 24.4 | 77.0 | 7.1 | 220 | 60 | 150 | 8.28 | 8.87 | 9.74 | 9.05 | 181 | 9.43 | 305 |
|  |  |  | FAS | | R | | MW | 39.2 | 172.0 | 18.7 | 90.0 | 6.3 | 180 | 60 | 120 | 8.00 | 8.36 | 8.91 | 8.83 | 184 | 8.80 | 332 |
|  |  |  | BE | | R | | MW | 10.0 | 64.0 | 17.3 | 91.0 | 8.3 | 180 | 60 | 120 | 8.21 | 8.60 | 9.12 | 8.75 | 142 | 8.92 | 265 |
|  | 38.1°N,  114.4°E | 2009 | CS | | R | | MW | 13.2 | 72.0 | 15.2 | 76.0 | 8.3 | 180 | 60 | 120 | 7.53 | 8.21 | 8.87 | 8.36 | 154 | 8.67 | 290 |
|  |  | 2010 | CCS | | R | | MW | 11.5 | 98.0 | 16.9 | 129.0 | 8.2 | 220 | 60 | 150 | 8.06 | 8.93 | 9.88 | 9.26 | 199 | 9.60 | 327 |
|  | 38.2°N,  114.5°E | 2010 | CCS | | R | | MW | 28.6 | 105.0 | 10.0 | 116.0 | 7.4 | 180 | 60 | 120 | 6.86 | 7.21 | 7.32 | 7.09 | 102 | 7.26 | 258 |
|  |  |  | CCS | | R | | MW | 19.2 | 154.0 | 8.3 | 81.0 | 7.8 | 220 | 150 | 150 | 7.37 | 7.86 | 8.06 | 8.09 | 170 | 8.00 | 341 |
|  |  |  | YE | | R | | MW | 16.8 | 157.5 | 28.2 | 116.0 | 5.7 | 220 | 60 | 150 | 7.85 | 8.21 | 8.42 | 8.45 | 149 | 8.30 | 295 |
| Zhuozhou,  Hebei | 39.2°N,  115.6°E | 2006 | PS | | R | | MW | 40.8 | 291.0 | 14.8 | 92.0 | 5.6 | 165 | 83 | 60 | 9.50 | 9.88 | 10.09 | 10.01 | 126 | 10.02 | 216 |
|  |  | 2007 | YE | | R | | MW | 63.5 | 189.0 | 8.9 | 83.0 | 8.3 | 160 | 138 | 108 | 6.76 | 8.21 | 8.54 | 7.84 | 134 | 8.56 | 264 |
|  |  |  | YE | | R | | MW | 23.1 | 103.1 | 13.9 | 139.0 | 6.2 | 220 | 138 | 180 | 6.29 | 9.20 | 9.52 | 8.05 | 182 | 9.69 | 298 |
|  | 39.3°N,  115.6°E | 2008 | PS | | R | | MW | 49.6 | 184.2 | 13.7 | 114.0 | 7.9 | 220 | 138 | 144 | 6.30 | 6.65 | 7.13 | 6.57 | 142 | 6.91 | 342 |
|  |  |  | PS | | I | | MW | 18.9 | 74.0 | 29.0 | 134.0 | 7.4 | 160 | 150 | 108 | 5.60 | 6.88 | 7.13 | 6.57 | 133 | 7.16 | 314 |
|  |  |  | PS | | R | | MW | 23.1 | 125.0 | 14.0 | 104.0 | 5.9 | 220 | 138 | 180 | 7.27 | 7.89 | 8.09 | 7.74 | 151 | 8.03 | 308 |
|  | 39.3°N,  116.1°E | 2009 | PS | | R | | MW | 18.6 | 86.0 | 15.3 | 136.8 | 6.0 | 220 | 138 | 144 | 4.08 | 7.94 | 7.67 | 8.38 | 238 | 8.49 | 440 |
|  |  |  | PS | | R | | MW | 19.6 | 100.0 | 15.2 | 94.0 | 6.4 | 180 | 138 | 144 | 7.97 | 8.66 | 9.62 | 9.01 | 166 | 9.32 | 287 |
|  | 39.3°N,  116.0°E | 2010 | RE | | R | | MW | 17.8 | 88.0 | 14.9 | 95.0 | 7.5 | 180 | 138 | 144 | 4.34 | 5.89 | 5.49 | 5.57 | 153 | 5.83 | 429 |
|  |  |  | YE | | R | | MW | 18.3 | 95.0 | 15.4 | 110.0 | 8.2 | 180 | 138 | 144 | 5.19 | 6.59 | 8.02 | 5.99 | 145 | 7.56 | 317 |
| Binzhou,  Shandong | 37.1°N,  117.5°E | 2008 | YE | | R | | MW | 11.8 | 102.0 | 28.4 | 125.0 | 6.8 | 195 | 60 | 90 | 9.05 | 9.49 | 10.30 | 10.05 | 197 | 10.09 | 308 |
|  |  |  | YE | | R | | MW | 19.2 | 114.0 | 11.3 | 142.0 | 7.5 | 195 | 60 | 90 | 5.60 | 6.28 | 6.83 | 6.85 | 214 | 6.81 | 493 |
|  |  |  | YE | | R | | MW | 34.0 | 154.0 | 18.8 | 135.0 | 6.7 | 225 | 80 | 120 | 8.79 | 9.48 | 10.01 | 9.45 | 172 | 9.84 | 280 |
|  | 37.2°N,  117.6°E | 2006 | LS | | R | | MW | 13.2 | 69.0 | 17.2 | 165.0 | 7.6 | 195 | 60 | 60 | 5.19 | 5.70 | 5.78 | 5.83 | 145 | 5.77 | 416 |
|  |  |  | YE | | I | | MW | 13.9 | 89.0 | 14.0 | 187.0 | 7.4 | 195 | 60 | 90 | 4.65 | 5.35 | 5.50 | 5.45 | 160 | 5.50 | 472 |
|  |  |  | YE | | R | | MW | 18.3 | 112.0 | 13.5 | 165.0 | 6.8 | 195 | 60 | 60 | 5.97 | 6.35 | 6.77 | 6.73 | 178 | 6.66 | 426 |
|  | 37.3°N,  117.8°E | 2007 | YE | | R | | MW | 8.1 | 154.0 | 36.9 | 165.0 | 6.9 | 195 | 60 | 60 | 5.78 | 6.30 | 6.57 | 6.46 | 156 | 6.50 | 391 |
|  |  |  | RE | | R | | MP | 15.9 | 142.0 | 45.7 | 111.0 | 6.5 | 195 | 60 | 60 | 6.15 | 6.63 | 6.69 | 6.87 | 160 | 6.71 | 387 |
|  |  | 2008 | RE | | I | | MW | 16.5 | 125.0 | 8.9 | 89.0 | 7.1 | 195 | 60 | 60 | 5.67 | 6.38 | 6.23 | 6.30 | 139 | 6.33 | 367 |
|  |  |  | PS | | R | | MW | 12.1 | 123.0 | 11.1 | 98.0 | 8.0 | 195 | 60 | 60 | 4.60 | 5.36 | 5.52 | 5.72 | 200 | 5.63 | 559 |
| Dezhou,  Shandong | 37.2°N,  116.1°E | 2007 | PS | | R | | MW | 14.1 | 98.0 | 15.0 | 114.0 | 7.4 | 210 | 60 | 150 | 8.53 | 9.31 | 9.74 | 9.47 | 179 | 9.65 | 295 |
|  |  | 2008 | PS | | R | | MW | 12.0 | 78.0 | 15.9 | 100.0 | 8.0 | 195 | 60 | 90 | 5.91 | 6.62 | 6.75 | 6.92 | 189 | 6.83 | 438 |
|  |  | 2009 | YB | | R | | MW | 22.0 | 134.0 | 15.4 | 170.0 | 6.8 | 195 | 60 | 90 | 5.23 | 5.81 | 6.82 | 6.11 | 171 | 6.47 | 424 |
|  | 37.3°N,  116.1°E | 2006 | FAS | | R | | MW | 20.4 | 122.0 | 38.4 | 123.0 | 6.9 | 195 | 60 | 90 | 4.82 | 5.57 | 6.39 | 5.67 | 164 | 6.12 | 432 |
|  |  | 2008 | BE | | R | | MW | 10.1 | 65.3 | 29.8 | 101.0 | 7.5 | 195 | 60 | 90 | 5.80 | 6.31 | 6.48 | 6.71 | 207 | 6.57 | 495 |
|  |  |  | CS | | R | | MW | 10.6 | 65.3 | 17.4 | 91.0 | 7.6 | 195 | 60 | 90 | 6.04 | 6.52 | 6.62 | 6.79 | 168 | 6.64 | 407 |
|  | 37.3°N,  116.2°E | 2006 | CCS | | R | | MW | 18.7 | 75.3 | 25.4 | 117.0 | 7.4 | 195 | 60 | 90 | 6.89 | 7.53 | 7.98 | 7.94 | 191 | 7.93 | 381 |
|  |  | 2007 | CCS | | R | | MW | 13.5 | 49.0 | 28.3 | 110.0 | 8.0 | 156 | 36 | 48 | 6.63 | 6.93 | 7.20 | 7.22 | 140 | 7.14 | 327 |
|  |  | 2009 | CCS | | R | | MW | 19.8 | 110.0 | 16.6 | 170.0 | 6.8 | 195 | 60 | 23 | 4.82 | 5.51 | 5.54 | 5.77 | 183 | 5.65 | 514 |
|  |  | 2010 | YE | | R | | MW | 20.8 | 137.0 | 21.0 | 90.0 | 6.9 | 195 | 60 | 90 | 7.20 | 7.87 | 8.65 | 8.48 | 207 | 8.52 | 382 |
| Dongying,  Shandong | 37.3°N,  118.4°E | 2005 | PS | | R | | MW | 13.6 | 89.0 | 17.5 | 77.0 | 8.6 | 210 | 120 | 60 | 4.30 | 5.05 | 5.89 | 5.56 | 205 | 5.69 | 566 |
|  |  | 2006 | YE | | R | | MW | 16.3 | 98.0 | 11.7 | 154.0 | 6.8 | 195 | 60 | 60 | 4.35 | 5.45 | 5.70 | 5.93 | 213 | 5.87 | 569 |
|  |  | 2007 | YE | | R | | MW | 18.1 | 88.0 | 11.4 | 124.0 | 6.5 | 210 | 75 | 65 | 3.89 | 5.01 | 5.43 | 5.55 | 226 | 5.53 | 641 |
|  |  | 2008 | PS | | R | | MW | 15.1 | 62.9 | 27.9 | 114.0 | 7.8 | 195 | 75 | 65 | 6.13 | 7.05 | 7.61 | 7.71 | 225 | 7.68 | 459 |
| Heze,  Shandong | 35.1°N,  115.3°E | 2007 | PS | | I | | MW | 11.2 | 58.0 | 8.4 | 153.0 | 7.9 | 195 | 60 | 90 | 6.27 | 6.98 | 7.67 | 7.68 | 229 | 7.66 | 469 |
|  |  | 2008 | PS | | R | | MW | 13.5 | 69.0 | 14.0 | 126.0 | 8.4 | 225 | 120 | 120 | 5.70 | 6.58 | 6.58 | 6.75 | 195 | 6.69 | 460 |
|  |  | 2009 | PS | | R | | MW | 16.2 | 59.0 | 8.9 | 145.0 | 7.4 | 195 | 60 | 90 | 5.78 | 6.62 | 7.10 | 7.20 | 219 | 7.15 | 480 |
| Ji'nan,  Shandong | 36.6°N,  116.5°E | 2006 | PS | | R | | MW | 8.3 | 127.0 | 33.5 | 100.0 | 7.4 | 165 | 60 | 72 | 5.48 | 5.93 | 6.16 | 6.26 | 166 | 6.18 | 433 |
|  |  | 2007 | RE | | R | | MW | 18.1 | 103.4 | 18.9 | 161.0 | 8.0 | 165 | 60 | 72 | 5.89 | 6.56 | 6.90 | 6.73 | 147 | 6.84 | 354 |
|  |  |  | YE | | R | | MW | 44.2 | 126.0 | 22.8 | 152.0 | 7.5 | 165 | 60 | 72 | 6.18 | 6.85 | 7.21 | 7.04 | 148 | 7.15 | 341 |
|  |  | 2008 | YE | | R | | MW | 23.8 | 69.8 | 10.9 | 145.0 | 7.6 | 165 | 45 | 60 | 5.65 | 6.47 | 6.81 | 6.77 | 162 | 6.82 | 384 |
|  | 36.5°N,  116.4°E | 2008 | YE | | R | | MW | 23.4 | 93.7 | 22.5 | 132.0 | 6.9 | 165 | 45 | 60 | 6.49 | 6.80 | 7.39 | 7.37 | 199 | 7.34 | 427 |
|  |  | 2009 | YE | | R | | MW | 14.6 | 90.0 | 25.5 | 142.0 | 8.0 | 165 | 48 | 60 | 6.62 | 7.24 | 7.96 | 7.48 | 148 | 7.74 | 315 |
|  |  |  | LS | | R | | MW | 18.9 | 86.0 | 25.1 | 116.0 | 7.6 | 165 | 48 | 60 | 6.57 | 7.38 | 7.85 | 7.50 | 146 | 7.76 | 311 |
|  |  | 2010 | YE | | I | | MW | 15.7 | 85.0 | 24.7 | 89.0 | 6.5 | 188 | 45 | 60 | 4.25 | 5.44 | 5.96 | 5.94 | 198 | 6.00 | 520 |
| Jining,  Shandong | 35.2°N,  116.3°E | 2007 | YE | | R | | MW | 14.5 | 127.0 | 26.5 | 100.0 | 6.5 | 195 | 30 | 150 | 8.81 | 9.15 | 9.33 | 9.35 | 132 | 9.23 | 242 |
|  |  | 2008 | YE | | R | | MW | 15.1 | 89.0 | 11.9 | 69.0 | 6.7 | 195 | 60 | 90 | 8.38 | 8.98 | 9.30 | 9.43 | 207 | 9.34 | 348 |
|  |  |  | RE | | R | | MP | 16.5 | 79.8 | 26.0 | 98.0 | 7.5 | 195 | 60 | 90 | 8.71 | 9.41 | 9.64 | 9.75 | 192 | 9.68 | 313 |
|  |  | 2009 | RE | | I | | MW | 15.4 | 69.0 | 25.0 | 78.0 | 7.4 | 195 | 60 | 90 | 6.73 | 7.24 | 7.14 | 7.44 | 148 | 7.21 | 338 |
| Liaocheng,  Shandong | 36.3°N,  116.0°E | 2008  2008  2008 | PS | | R | | MW | 10.0 | 74.0 | 74.0 | 68.0 | 6.7 | 195 | 45 | 75 | 7.40 | 7.91 | 8.28 | 8.05 | 154 | 8.16 | 308 |
|  |  |  | PS | | R | | MW | 13.8 | 87.0 | 22.0 | 71.0 | 6.8 | 195 | 45 | 75 | 6.21 | 6.71 | 7.00 | 6.74 | 143 | 6.89 | 344 |
|  |  |  | PS | | R | | MW | 15.6 | 99.0 | 35.2 | 72.0 | 6.9 | 195 | 45 | 75 | 7.79 | 8.36 | 8.62 | 8.35 | 145 | 8.54 | 281 |
|  | 36.2°N,  116.0°E | 2009 | YB | | R | | MW | 17.2 | 105.0 | 13.4 | 72.0 | 8.4 | 195 | 45 | 75 | 8.04 | 8.46 | 8.68 | 8.60 | 142 | 8.59 | 275 |
|  |  |  | FAS | | R | | MW | 10.5 | 105.0 | 23.4 | 98.0 | 7.8 | 195 | 45 | 75 | 6.60 | 7.25 | 7.64 | 7.37 | 161 | 7.53 | 346 |
|  |  | 2010 | BE | | R | | MW | 9.0 | 89.0 | 12.7 | 200.0 | 7.6 | 195 | 120 | 120 | 7.86 | 8.41 | 8.52 | 8.73 | 187 | 8.59 | 345 |
|  |  |  | CS | | R | | MW | 13.3 | 98.0 | 40.2 | 80.0 | 7.5 | 210 | 75 | 120 | 8.39 | 8.82 | 9.12 | 9.15 | 180 | 9.05 | 317 |
|  | 36.2°N,  115.5°E | 2007 | CCS | | R | | MW | 9.3 | 114.0 | 16.5 | 80.0 | 7.9 | 195 | 60 | 90 | 6.68 | 7.28 | 7.79 | 7.84 | 216 | 7.79 | 435 |
|  |  | 2009 | CCS | | R | | MW | 7.4 | 125.0 | 42.5 | 89.0 | 6.7 | 210 | 75 | 120 | 5.61 | 6.26 | 6.30 | 6.53 | 188 | 6.38 | 467 |
|  |  |  | CCS | | R | | MW | 7.6 | 145.0 | 15.0 | 114.0 | 6.9 | 195 | 120 | 120 | 6.75 | 7.31 | 7.29 | 7.47 | 153 | 7.34 | 341 |
|  | 36.2°N,  115.6°E | 2006 | YE | | R | | MW | 8.7 | 112.0 | 18.0 | 85.0 | 6.5 | 195 | 120 | 120 | 6.23 | 6.86 | 6.71 | 6.90 | 140 | 6.80 | 343 |
|  |  | 2007 | PS | | R | | MW | 13.6 | 165.0 | 30.6 | 86.0 | 7.4 | 210 | 75 | 120 | 9.00 | 9.75 | 9.92 | 10.04 | 195 | 9.97 | 309 |
|  |  | 2008 | YE | | R | | MW | 8.7 | 89.0 | 14.0 | 159.0 | 7.6 | 210 | 75 | 120 | 4.79 | 5.46 | 5.67 | 5.83 | 207 | 5.73 | 568 |
|  |  | 2009 | YE | | R | | MW | 11.8 | 97.0 | 13.5 | 89.0 | 8.4 | 210 | 75 | 120 | 8.28 | 8.59 | 8.93 | 9.03 | 198 | 8.87 | 352 |
| Qingdao,  Shandong | 36.1°N,  120.0°E | 2009 | PS | | R | | MW | 16.6 | 124.0 | 15.6 | 154.0 | 7.2 | 195 | 75 | 90 | 7.52 | 7.97 | 8.38 | 8.13 | 153 | 8.23 | 304 |
|  |  |  | PS | | I | | MW | 13.5 | 50.0 | 10.9 | 145.0 | 8.6 | 195 | 75 | 90 | 6.48 | 7.14 | 8.03 | 7.58 | 184 | 7.78 | 375 |
|  |  | 2010 | PS | | R | | MW | 11.4 | 99.0 | 16.8 | 165.0 | 6.8 | 195 | 75 | 90 | 6.24 | 6.90 | 6.84 | 6.89 | 142 | 6.90 | 342 |
|  |  |  | PS | | R | | MW | 16.2 | 90.0 | 30.2 | 187.0 | 6.7 | 195 | 75 | 90 | 9.41 | 9.86 | 10.83 | 10.60 | 219 | 10.63 | 323 |
| Rizhao,  Shandong | 35.2°N,  119.2°E | 2007 | PS | | R | | MW | 31.4 | 163.0 | 9.0 | 191.0 | 7.6 | 195 | 120 | 90 | 6.49 | 7.31 | 8.27 | 7.92 | 201 | 8.06 | 393 |
| Tai'an,  Shandong | 36.1°N,  117.1°E | 2007 | RE | | R | | MW | 21.5 | 100.0 | 16.8 | 80.0 | 6.4 | 195 | 60 | 105 | 5.26 | 5.72 | 6.54 | 5.85 | 156 | 6.23 | 408 |
| Weifang,  Shandong | 36.4°N,  119.0°E | 2006 | RE | | I | | MW | 15.9 | 112.0 | 17.6 | 98.0 | 6.5 | 195 | 120 | 90 | 5.57 | 6.28 | 6.42 | 6.14 | 144 | 6.41 | 372 |
|  |  |  | PS | | R | | MW | 19.3 | 114.0 | 49.0 | 68.0 | 6.5 | 195 | 120 | 90 | 6.63 | 6.94 | 7.62 | 7.52 | 209 | 7.48 | 439 |
|  |  | 2008 | PS | | R | | MW | 17.5 | 78.0 | 25.0 | 88.0 | 8.0 | 195 | 120 | 90 | 6.80 | 7.31 | 7.62 | 7.64 | 179 | 7.57 | 377 |
|  |  |  | PS | | R | | MW | 27.3 | 126.0 | 9.8 | 63.0 | 8.0 | 195 | 120 | 90 | 7.20 | 7.83 | 8.22 | 8.21 | 189 | 8.18 | 366 |
|  |  | 2009 | YB | | R | | MW | 34.6 | 167.0 | 32.5 | 134.0 | 6.8 | 195 | 120 | 90 | 6.35 | 6.84 | 7.18 | 7.19 | 180 | 7.13 | 402 |
|  | 36.5°N,  119.0°E | 2006 | FAS | | R | | MW | 37.9 | 204.0 | 23.5 | 187.0 | 7.8 | 195 | 120 | 90 | 5.57 | 6.43 | 6.72 | 6.44 | 162 | 6.69 | 392 |
|  |  | 2007 | BE | | R | | MW | 21.4 | 103.0 | 14.8 | 196.0 | 7.8 | 195 | 120 | 90 | 5.74 | 6.58 | 6.89 | 6.86 | 184 | 6.90 | 423 |
|  |  | 2008 | CS | | R | | MW | 21.3 | 104.0 | 18.6 | 174.0 | 5.5 | 195 | 120 | 90 | 6.93 | 7.39 | 7.92 | 7.82 | 187 | 7.80 | 380 |
|  |  | 2009 | CCS | | R | | MW | 34.5 | 189.0 | 23.5 | 189.0 | 7.8 | 195 | 120 | 90 | 7.05 | 7.65 | 8.08 | 8.05 | 191 | 8.03 | 376 |
|  | 36.5°N,  119.1°E | 2007 | CCS | | R | | MW | 19.9 | 97.0 | 17.4 | 96.0 | 6.4 | 180 | 60 | 90 | 5.09 | 5.46 | 6.17 | 5.63 | 144 | 5.89 | 405 |
|  |  | 2008 | CCS | | R | | MW | 30.0 | 167.0 | 18.2 | 109.0 | 6.4 | 195 | 120 | 90 | 6.15 | 6.72 | 7.58 | 7.18 | 186 | 7.33 | 402 |
|  |  | 2009 | YE | | R | | MW | 27.1 | 114.0 | 37.2 | 163.0 | 7.5 | 180 | 60 | 90 | 6.38 | 6.88 | 7.15 | 7.11 | 156 | 7.10 | 358 |
|  |  | 2010 | PS | | R | | MW | 21.3 | 165.0 | 22.7 | 147.0 | 4.9 | 195 | 60 | 90 | 6.35 | 6.80 | 7.07 | 7.23 | 208 | 7.11 | 460 |
|  | 36.5°N,  119.0°E | 2007 | YE | | R | | MW | 36.0 | 115.0 | 17.8 | 115.0 | 6.8 | 195 | 60 | 90 | 6.28 | 6.79 | 6.72 | 6.97 | 146 | 6.77 | 356 |
|  |  | 2008 | YE | | R | | MW | 18.0 | 153.0 | 8.6 | 89.0 | 6.8 | 195 | 60 | 90 | 7.04 | 7.62 | 7.57 | 7.70 | 143 | 7.62 | 311 |
|  |  | 2009 | PS | | R | | MW | 35.3 | 117.0 | 16.9 | 78.0 | 7.6 | 195 | 60 | 90 | 5.35 | 6.00 | 6.21 | 6.11 | 159 | 6.18 | 418 |
|  |  | 2010 | PS | | I | | MW | 36.4 | 128.0 | 12.2 | 100.0 | 6.5 | 195 | 60 | 90 | 8.18 | 8.69 | 8.90 | 9.10 | 204 | 8.96 | 358 |
|  |  |  | PS | | R | | MW | 39.1 | 191.0 | 26.6 | 125.0 | 5.9 | 195 | 60 | 90 | 4.78 | 5.46 | 5.81 | 5.90 | 203 | 5.84 | 547 |
| Weihai,  Shandong | 37.3°N,  112.0°E | 2007 | PS | | R | | MW | 13.1 | 116.0 | 15.1 | 63.0 | 5.6 | 195 | 30 | 150 | 6.55 | 7.14 | 7.07 | 7.36 | 169 | 7.17 | 379 |
| Yantai,  Shandong | 37.3°N,  121.2°E | 2008 | PS | | R | | MW | 24.3 | 145.0 | 9.3 | 95.0 | 6.4 | 150 | 45 | 75 | 6.23 | 6.62 | 6.78 | 7.00 | 192 | 6.92 | 438 |
| Zaozhuang,  Shandong | 37.5°N,  117.2°E | 2008 | RE | | R | | MW | 25.0 | 167.0 | 18.7 | 147.0 | 6.4 | 210 | 45 | 90 | 6.91 | 7.47 | 8.01 | 7.92 | 203 | 7.90 | 404 |
| zibo,  Shandong | 36.5°N,  118.0°E | 2008 | YE | | R | | MW | 15.6 | 145.0 | 32.1 | 201.0 | 7.5 | 195 | 60 | 90 | 8.98 | 9.30 | 9.43 | 9.58 | 138 | 9.36 | 247 |
| Jiaozuo,  He'nan | 34.5°N,  112.4°E | 2006 | YE | | R | | MW | 15.6 | 98.0 | 20.1 | 114.0 | 6.8 | 210 | 75 | 75 | 5.75 | 7.30 | 7.71 | 6.57 | 163 | 7.69 | 342 |
|  |  |  | YE | | R | | MW | 17.8 | 96.0 | 11.7 | 125.0 | 6.8 | 210 | 75 | 75 | 7.22 | 8.29 | 9.47 | 8.51 | 187 | 9.11 | 325 |
|  |  |  | YE | | R | | MW | 19.5 | 115.0 | 11.9 | 145.0 | 6.5 | 210 | 75 | 75 | 5.75 | 7.30 | 7.71 | 6.57 | 163 | 7.69 | 342 |
|  |  | 2007 | LS | | R | | MW | 16.4 | 117.0 | 15.4 | 154.0 | 8.5 | 210 | 75 | 75 | 7.20 | 8.29 | 8.90 | 8.43 | 186 | 8.78 | 336 |
|  |  |  | YE | | I | | MW | 15.8 | 154.0 | 15.0 | 124.0 | 7.5 | 180 | 60 | 60 | 5.53 | 6.34 | 6.62 | 6.59 | 170 | 6.63 | 411 |
| Jiaxian,  He'nan | 33.6°N,  113.1°E | 2006 | YE | | R | | MW | 8.2 | 71.0 | 9.3 | 124.0 | 7.0 | 150 | 60 | 60 | 5.51 | 7.26 | 7.02 | 7.52 | 163 | 7.47 | 353 |
| Jingyang,  Shaanxi | 34.3°N,  108.5°E | 2006 | YE | | R | | MW | 13.9 | 170.0 | 35.1 | 89.0 | 7.5 | 195 | 105 | 90 | 6.18 | 7.30 | 8.34 | 8.08 | 213 | 8.21 | 407 |
|  |  |  | RE | | R | | MP | 14.1 | 162.0 | 17.5 | 112.0 | 7.8 | 195 | 105 | 83 | 6.59 | 7.44 | 8.31 | 8.13 | 212 | 8.19 | 406 |
|  |  | 2007 | RE | | I | | MW | 22.1 | 90.3 | 27.4 | 110.0 | 7.6 | 180 | 90 | 75 | 6.33 | 7.32 | 8.22 | 8.06 | 202 | 8.14 | 391 |
|  |  |  | PS | | R | | MW | 17.2 | 84.0 | 26.0 | 112.0 | 6.8 | 180 | 90 | 75 | 5.80 | 7.05 | 7.89 | 7.64 | 189 | 7.81 | 383 |
| Qixian,  He'nan | 34.3°N,  114.5°E | 2007 | PS | | R | | MW | 14.5 | 96.0 | 12.5 | 210.0 | 6.8 | 180 | 60 | 60 | 6.83 | 8.23 | 9.36 | 9.11 | 202 | 9.26 | 343 |
|  |  |  | PS | | R | | MW | 13.5 | 95.0 | 14.6 | 89.0 | 8.4 | 210 | 75 | 75 | 8.24 | 8.86 | 9.83 | 9.04 | 175 | 9.48 | 295 |
| Shangqiu,  He'nan | 34.2°N,  115.4°E | 2007 | YB | | R | | MW | 32.9 | 112.0 | 12.3 | 78.0 | 6.8 | 225 | 90 | 90 | 6.69 | 7.85 | 8.48 | 7.49 | 176 | 8.31 | 338 |
|  |  |  | YE | | R | | MW | 16.9 | 98.5 | 15.1 | 76.0 | 6.8 | 225 | 90 | 90 | 6.54 | 8.22 | 8.91 | 7.08 | 169 | 8.78 | 309 |
| Weinan,  Shaanxi | 34.3°N,  109.3°E | 2007 | YE | | R | | MW | 23.1 | 131.0 | 15.5 | 137.0 | 7.9 | 180 | 60 | 45 | 7.20 | 8.00 | 9.00 | 8.05 | 152 | 8.67 | 287 |
|  |  |  | YE | | R | | MW | 17.8 | 165.0 | 17.4 | 97.0 | 6.1 | 180 | 60 | 45 | 7.88 | 8.57 | 9.55 | 8.65 | 151 | 9.20 | 269 |
|  |  | 2008 | LS | | R | | MW | 42.8 | 253.0 | 18.0 | 46.0 | 6.1 | 180 | 60 | 45 | 8.05 | 8.75 | 9.71 | 8.81 | 150 | 9.37 | 263 |
| Xianyang,  Shaanxi | 34.1°N,  108.0°E | 2006 | YE | | I | | MW | 32.9 | 190.0 | 36.1 | 203.0 | 7.1 | 218 | 120 | 39 | 4.81 | 6.99 | 7.22 | 7.21 | 215 | 7.47 | 452 |
|  |  | 2007 | YE | | R | | MW | 44.4 | 168.0 | 14.9 | 179.0 | 6.9 | 180 | 60 | 45 | 4.88 | 6.41 | 7.30 | 6.99 | 187 | 7.23 | 410 |
|  |  | 2008 | YE | | R | | MW | 32.8 | 124.0 | 31.0 | 120.0 | 6.2 | 180 | 60 | 45 | 4.69 | 6.19 | 7.54 | 6.88 | 185 | 7.28 | 403 |
|  |  | 2009 | RE | | R | | MP | 34.6 | 143.0 | 20.8 | 116.0 | 5.3 | 180 | 60 | 45 | 4.78 | 6.87 | 8.48 | 7.89 | 196 | 8.28 | 373 |
| Xiayi,  He'nan | 34.1°N,  116.1°E | 2007 | RE | | I | | MW | 20.1 | 81.0 | 13.2 | 92.0 | 7.8 | 225 | 90 | 90 | 6.57 | 8.07 | 9.35 | 8.70 | 225 | 9.09 | 388 |
|  |  |  | YE | | R | | MW | 30.9 | 129.0 | 18.1 | 124.0 | 8.1 | 210 | 75 | 75 | 5.94 | 7.19 | 8.22 | 7.04 | 176 | 7.91 | 355 |
| Xinzheng,  He'nan | 34.2°N,  113.4°E | 2007 | RE | | R | | MP | 17.9 | 154.0 | 14.5 | 145.0 | 6.4 | 180 | 120 | 120 | 7.67 | 8.61 | 9.36 | 8.55 | 151 | 9.14 | 271 |
| Xuchang,  He'nan | 34.1°N,  113.5°E | 2008 | RE | | I | | MW | 31.7 | 180.0 | 11.7 | 145.0 | 7.8 | 225 | 90 | 90 | 6.05 | 7.15 | 7.07 | 6.95 | 175 | 7.20 | 388 |
|  |  | 2009 | PS | | R | | MW | 14.9 | 115.0 | 15.0 | 55.0 | 8.0 | 180 | 60 | 60 | 7.12 | 7.44 | 7.72 | 7.60 | 129 | 7.59 | 289 |
| Yuncheng,  Shanxi | 35.0°N,  110.6°E | 2007 | PS | | R | | MW | 11.6 | 145.0 | 15.0 | 154.0 | 6.7 | 180 | 90 | 120 | 7.50 | 9.00 | 9.20 | 9.23 | 179 | 9.37 | 304 |
|  |  |  | PS | | R | | MW | 37.1 | 116.0 | 21.4 | 147.0 | 6.6 | 180 | 90 | 120 | 6.33 | 8.67 | 9.75 | 8.71 | 169 | 9.61 | 282 |
| **NCP2 subregion** |  |  |  | |  | |  |  |  |  |  |  |  |  |  |  |  |  |  |  |  |  |
| Cangzhou,  Hebei | 38.0°N,  116.2°E | 2008 | FAS | | R | | MW | 14.6 | 75.0 | 15.7 | 88.0 | 8.1 | 180 | 60 | 120 | 5.49 | 5.72 | 6.12 | 5.98 | 141 | 5.91 | 396 |
|  |  |  | Solon. | | R | | MW | 13.4 | 75.0 | 16.6 | 98.0 | 6.9 | 225 | 60 | 150 | 7.18 | 7.71 | 7.94 | 7.09 | 124 | 7.88 | 270 |
|  |  | 2009 | FAS | | R | | MW | 12.3 | 110.0 | 15.4 | 92.0 | 8.1 | 225 | 60 | 150 | 5.05 | 5.98 | 3.78 | 7.01 | 121 | 4.69 | 446 |
|  | 37.6°N,  116.4°E | 2006 | FAS | | R | | MW | 18.5 | 81.0 | 17.3 | 123.0 | 7.4 | 135 | 60 | 90 | 9.67 | 9.92 | 11.09 | 9.09 | 132 | 10.63 | 210 |
|  |  | 2007 | FAS | | R | | MW | 16.7 | 56.0 | 8.3 | 57.0 | 7.5 | 225 | 60 | 150 | 7.39 | 7.67 | 8.18 | 7.69 | 148 | 7.92 | 307 |
|  |  | 2007 | FAS | | R | | MW | 7.8 | 121.0 | 10.2 | 79.0 | 6.9 | 225 | 60 | 150 | 5.36 | 6.42 | 6.93 | 6.75 | 137 | 6.91 | 332 |
|  |  | 2008 | PS | | R | | MW | 9.0 | 97.0 | 10.6 | 65.0 | 8.1 | 180 | 60 | 120 | 5.39 | 6.88 | 7.19 | 7.13 | 138 | 7.31 | 315 |
|  | 37.8°N,  116.3°E | 2006 | PS | | R | | MS | 21.3 | 85.7 | 17.8 | 120.0 | 7.6 | 180 | 60 | 120 | 6.59 | 7.12 | 7.34 | 6.95 | 150 | 7.24 | 339 |
|  |  | 2007 | PS | | R | | MW | 16.6 | 76.5 | 11.6 | 90.0 | 7.8 | 180 | 60 | 120 | 7.32 | 8.05 | 8.22 | 7.35 | 147 | 8.20 | 295 |
|  |  | 2010 | LCFS | | R | | MW | 16.9 | 152.0 | 20.1 | 150.0 | 7.4 | 135 | 60 | 90 | 5.46 | 6.13 | 6.81 | 6.12 | 146 | 6.58 | 366 |
| Luohe,  He'nan | 33.3°N,  114.0°E | 2007 | YB | | R | | MW | 20.1 | 148.0 | 21.2 | 146.0 | 7.4 | 210 | 75 | 75 | 5.09 | 6.11 | 6.48 | 5.85 | 217 | 6.38 | 533 |
|  |  |  | LCFS | | R | | MW | 15.8 | 98.0 | 14.5 | 185.0 | 6.8 | 210 | 75 | 75 | 5.89 | 7.41 | 7.35 | 7.69 | 214 | 7.66 | 438 |
|  |  |  | BE | | R | | MW | 18.9 | 106.0 | 15.6 | 169.0 | 6.5 | 240 | 90 | 90 | 4.44 | 5.95 | 6.23 | 5.98 | 213 | 6.30 | 530 |
|  | 32.4°N,  112.1°E | 2008 | DBE | | R | | MW | 13.8 | 75.1 | 25.9 | 80.0 | 7.7 | 210 | 113 | 113 | 7.49 | 8.76 | 7.32 | 7.83 | 89 | 8.01 | 214 |
|  |  |  | BE | | R | | MS | 10.5 | 57.2 | 18.9 | 121.0 | 7.1 | 180 | 75 | 75 | 6.92 | 7.18 | 7.78 | 7.06 | 162 | 7.46 | 350 |
|  |  |  | DBE | | R | | MW | 15.5 | 119.0 | 19.0 | 104.0 | 6.9 | 210 | 75 | 75 | 5.65 | 6.18 | 7.64 | 6.36 | 152 | 7.07 | 351 |
| Zhoukou,  He'nan | 34.0°N,  114.2°E | 2006 | LS | | R | | MW | 23.7 | 113.4 | 17.4 | 56.0 | 6.8 | 210 | 75 | 75 | 6.35 | 7.30 | 7.70 | 7.46 | 164 | 7.67 | 344 |
|  |  |  | YE | | R | | MW | 16.3 | 145.0 | 17.4 | 81.0 | 8.1 | 210 | 75 | 75 | 6.10 | 7.99 | 8.72 | 6.76 | 160 | 8.60 | 301 |
|  |  | 2007 | YE | | R | | MW | 16.4 | 135.0 | 22.3 | 82.0 | 8.0 | 180 | 60 | 60 | 4.73 | 5.71 | 6.39 | 6.43 | 164 | 6.44 | 410 |
|  |  |  | YE | | R | | MW | 15.7 | 122.0 | 16.7 | 66.0 | 7.8 | 180 | 60 | 60 | 4.64 | 5.30 | 6.59 | 5.20 | 166 | 6.09 | 438 |
|  | 34.1°N,  114.2°E | 2006 | YE | | R | | MW | 12.4 | 131.0 | 14.1 | 60.0 | 6.7 | 240 | 90 | 90 | 6.77 | 9.55 | 9.57 | 6.41 | 166 | 9.92 | 269 |
|  |  | 2007 | LCFS | | R | | MW | 21.4 | 124.0 | 19.1 | 115.0 | 6.8 | 240 | 90 | 90 | 5.27 | 6.48 | 7.22 | 6.71 | 169 | 7.08 | 382 |
|  |  |  | PS | | R | | MW | 19.5 | 101.0 | 8.2 | 80.0 | 6.5 | 180 | 60 | 60 | 6.62 | 8.52 | 9.00 | 8.58 | 170 | 9.07 | 300 |
|  |  | 2008 | PS | | R | | MW | 18.1 | 115.0 | 17.7 | 89.0 | 6.4 | 240 | 90 | 90 | 5.93 | 6.14 | 6.84 | 6.62 | 188 | 6.59 | 451 |
|  |  |  | PS | | R | | MW | 14.0 | 132.0 | 16.4 | 136.0 | 8.0 | 240 | 90 | 90 | 5.26 | 6.09 | 6.76 | 6.85 | 174 | 6.84 | 406 |
|  |  |  | PS | | R | | MW | 10.5 | 142.0 | 10.9 | 164.0 | 7.6 | 210 | 75 | 75 | 8.28 | 9.58 | 9.48 | 9.11 | 179 | 9.63 | 295 |
|  | 34.0°N,  114.5°E | 2006 | CS | | R | | MP | 24.4 | 121.0 | 14.9 | 179.0 | 6.9 | 210 | 75 | 75 | 4.50 | 6.03 | 8.15 | 7.59 | 217 | 7.82 | 435 |
|  |  |  | FAS | | R | | MS | 23.3 | 102.0 | 8.1 | 62.0 | 5.5 | 240 | 90 | 90 | 3.93 | 5.44 | 6.48 | 6.51 | 219 | 6.55 | 523 |
|  |  | 2007 | FAS | | R | | MW | 22.8 | 110.0 | 21.0 | 120.0 | 6.2 | 240 | 90 | 90 | 4.23 | 5.53 | 7.51 | 7.00 | 219 | 7.20 | 476 |
|  |  |  | FAS | | R | | MW | 24.6 | 143.0 | 20.8 | 116.0 | 5.3 | 210 | 75 | 75 | 4.74 | 6.09 | 6.45 | 6.33 | 223 | 6.50 | 537 |
|  | 34.1°N,  114.5°E | 2008 | PS | | R | | MW | 32.1 | 142.0 | 18.0 | 179.0 | 6.4 | 180 | 60 | 60 | 6.77 | 7.83 | 8.20 | 8.27 | 226 | 8.26 | 428 |
|  |  |  | PS | | R | | MW | 22.5 | 158.0 | 25.7 | 56.0 | 6.0 | 210 | 75 | 75 | 5.19 | 6.58 | 6.77 | 6.50 | 228 | 6.83 | 523 |
|  |  |  | YE | | R | | MW | 28.7 | 135.0 | 17.2 | 101.0 | 5.7 | 240 | 90 | 90 | 6.44 | 8.40 | 9.60 | 9.71 | 224 | 9.75 | 360 |
|  |  | 2009 | CS | | R | | MW | 27.6 | 102.0 | 21.3 | 136.0 | 6.6 | 180 | 60 | 60 | 3.45 | 5.43 | 6.50 | 6.69 | 226 | 6.71 | 527 |
|  |  |  | PS | | R | | MW | 31.9 | 136.0 | 9.5 | 98.0 | 6.4 | 240 | 90 | 90 | 3.87 | 6.10 | 6.83 | 6.57 | 227 | 6.91 | 514 |
|  |  | 2010 | PS | | R | | MP | 24.7 | 112.0 | 20.0 | 265.0 | 7.2 | 180 | 60 | 60 | 5.34 | 6.26 | 9.30 | 7.79 | 229 | 8.36 | 429 |
|  |  |  | YE | | R | | MW | 20.2 | 148.0 | 20.7 | 90.0 | 6.5 | 240 | 90 | 90 | 5.10 | 6.24 | 8.00 | 6.58 | 233 | 7.40 | 493 |
| Fuyang,  Anhui | 32.5°N,  115.5°E | 2006 | YE | | R | | MW | 14.2 | 97.0 | 15.9 | 108.0 | 7.5 | 240 | 68 | 90 | 4.83 | 5.93 | 6.75 | 5.64 | 178 | 6.50 | 436 |
|  |  | 2007 | YE | | R | | MW | 10.6 | 154.0 | 10.6 | 110.0 | 8.4 | 225 | 60 | 90 | 8.26 | 8.98 | 9.88 | 8.85 | 183 | 9.54 | 304 |
|  |  | 2008 | YE | | R | | MW | 10.2 | 136.0 | 19.6 | 66.0 | 7.6 | 225 | 60 | 90 | 5.23 | 6.37 | 8.00 | 6.40 | 180 | 7.44 | 384 |
|  | 33.1°N,  115.2°E | 2006 | YE | | R | | MW | 11.8 | 96.0 | 18.9 | 110.0 | 7.5 | 240 | 90 | 90 | 5.69 | 5.95 | 6.71 | 6.46 | 195 | 6.45 | 476 |
|  |  | 2007 | LCFS | | R | | MW | 15.1 | 98.0 | 16.4 | 114.0 | 7.8 | 300 | 90 | 150 | 5.07 | 6.07 | 7.52 | 6.22 | 183 | 7.02 | 413 |
|  | 33.1°N,  115.3°E | 2007 | PS | | R | | MW | 14.3 | 123.0 | 16.8 | 110.0 | 7.9 | 276 | 36 | 140 | 6.32 | 8.54 | 8.77 | 7.76 | 184 | 8.91 | 327 |
|  |  | 2008 | PS | | R | | MW | 8.5 | 77.2 | 25.3 | 125.0 | 6.8 | 276 | 36 | 140 | 7.33 | 7.62 | 8.51 | 8.10 | 198 | 8.18 | 381 |
|  |  | 2009 | PS | | R | | MW | 10.4 | 79.3 | 15.9 | 110.0 | 7.1 | 276 | 36 | 140 | 6.40 | 7.45 | 7.82 | 7.59 | 198 | 7.79 | 400 |
|  | 33.0°N,  115.3°E | 2009 | PS | | R | | MW | 16.7 | 90.0 | 20.4 | 130.0 | 6.9 | 180 | 75 | 90 | 4.15 | 7.47 | 8.40 | 8.22 | 193 | 8.64 | 352 |
|  |  |  | CS | | R | | MP | 12.8 | 59.1 | 21.2 | 100.0 | 6.8 | 180 | 75 | 90 | 5.06 | 6.45 | 7.71 | 6.10 | 195 | 7.31 | 420 |
|  | 32.6°N,  115.2°E | 2007 | FAS | | R | | MS | 9.8 | 114.0 | 16.7 | 99.0 | 6.5 | 240 | 100 | 180 | 6.35 | 7.30 | 7.70 | 7.46 | 206 | 7.64 | 423 |
|  |  |  | FAS | | R | | MW | 12.4 | 114.0 | 25.6 | 105.0 | 6.7 | 200 | 100 | 150 | 6.31 | 6.71 | 7.55 | 7.40 | 209 | 7.41 | 442 |
|  |  |  | FAS | | R | | MW | 13.4 | 145.0 | 16.3 | 106.0 | 7.8 | 180 | 60 | 120 | 6.30 | 9.11 | 9.81 | 10.01 | 204 | 10.14 | 316 |
|  |  |  | PS | | R | | MW | 12.2 | 121.0 | 17.2 | 108.0 | 8.1 | 180 | 60 | 120 | 4.83 | 5.93 | 6.75 | 5.64 | 208 | 6.49 | 503 |
| Haozhou,  Anhui | 33.1°N,  116.3°E | 2006 | PS | | R | | MW | 18.6 | 96.0 | 15.9 | 154.0 | 8.1 | 240 | 108 | 113 | 4.95 | 5.37 | 7.88 | 6.21 | 211 | 6.92 | 478 |
|  |  |  | YE | | R | | MW | 19.6 | 89.0 | 21.4 | 154.0 | 6.3 | 225 | 60 | 90 | 5.04 | 6.81 | 7.77 | 7.81 | 212 | 7.88 | 422 |
| Huaibei,  Anhui | 33.5°N,  116.4°E | 2006 | CS | | R | | MW | 27.5 | 75.0 | 22.1 | 138.0 | 6.3 | 225 | 60 | 90 | 5.81 | 7.45 | 7.91 | 7.69 | 215 | 7.97 | 423 |
| Suzhou,  Anhui | 32.2°N,  116.3°E | 2007 | PS | | R | | MW | 13.5 | 100.0 | 11.2 | 121.0 | 7.4 | 300 | 90 | 150 | 5.63 | 6.92 | 8.88 | 7.64 | 206 | 8.29 | 390 |
|  |  | 2008 | PS | | R | | MP | 29.5 | 98.0 | 24.1 | 256.0 | 7.7 | 225 | 60 | 90 | 4.14 | 6.85 | 8.43 | 7.77 | 211 | 8.30 | 398 |
| Haozhou,  Anhui | 33.1°N,  116.3°E | 2006 | YE | | R | | MW | 18.6 | 96.0 | 15.9 | 154.0 | 8.1 | 240 | 108 | 113 | 4.95 | 5.37 | 7.88 | 6.21 | 211 | 6.92 | 478 |
|  |  |  | PS | | R | | MW | 19.6 | 89.0 | 21.4 | 154.0 | 6.3 | 225 | 60 | 90 | 5.04 | 6.81 | 7.77 | 7.81 | 212 | 7.88 | 422 |
| Huaibei,  Anhui | 33.5°N,  116.4°E | 2006 | YE | | R | | MW | 27.5 | 75.0 | 22.1 | 138.0 | 6.3 | 225 | 60 | 90 | 5.81 | 7.45 | 7.91 | 7.69 | 215 | 7.97 | 423 |
| **NW1 subregion** |  |  |  | |  | |  |  |  |  |  |  |  |  |  |  |  |  |  |  |  |  |
| Chengde,  Hebei | 40.5°N,  118.1°E | 2007 | CS | | R | | MM | 16.3 | 80.0 | 13.9 | 74.0 | 8.3 | 225 | 138 | 90 | 5.31 | 6.83 | 7.02 | 6.95 | 209 | 7.15 | 460 |
|  | 41.2°N,  117.4°E | 2008 | FAS | | R | | MM | 17.0 | 105.0 | 17.4 | 193.0 | 7.2 | 240 | 120 | 75 | 6.30 | 7.05 | 7.80 | 7.37 | 210 | 7.58 | 436 |
| Fengning,  Hebei | 41.1°N,  116.4°E | 2006 | CS | | R | | MM | 15.2 | 98.0 | 14.5 | 93.0 | 7.6 | 225 | 60 | 150 | 7.04 | 8.06 | 10.16 | 8.91 | 223 | 9.49 | 369 |
|  |  |  | DC | | R | | MW | 14.0 | 82.8 | 18.7 | 160.0 | 7.7 | 180 | 60 | 120 | 5.58 | 5.97 | 7.46 | 7.04 | 224 | 7.14 | 493 |
|  |  |  | BE | | R | | MM | 28.6 | 121.0 | 17.9 | 150.0 | 7.6 | 180 | 60 | 120 | 5.58 | 5.97 | 7.46 | 6.93 | 208 | 7.07 | 463 |
|  |  |  | CS | | R | | MM | 15.9 | 114.0 | 29.5 | 166.0 | 7.0 | 180 | 60 | 120 | 7.02 | 7.97 | 10.08 | 8.94 | 187 | 9.43 | 315 |
|  |  | 2007 | DC | | R | | MM | 21.6 | 102.0 | 12.3 | 162.0 | 7.9 | 225 | 60 | 150 | 7.04 | 8.06 | 10.16 | 8.91 | 223 | 9.49 | 369 |
|  |  |  | CS | | R | | MM | 17.3 | 99.0 | 35.5 | 151.0 | 7.6 | 180 | 60 | 120 | 5.58 | 5.97 | 7.46 | 7.04 | 224 | 7.14 | 493 |
|  |  |  | CS | | R | | MW | 9.4 | 78.0 | 11.9 | 145.0 | 8.1 | 180 | 60 | 120 | 7.02 | 7.97 | 10.08 | 8.94 | 187 | 9.43 | 315 |
|  | 41.2°N,  116.4°E | 2007 | CS | | R | | MW | 16.6 | 87.0 | 10.3 | 68.0 | 8.2 | 180 | 60 | 120 | 5.58 | 5.97 | 7.46 | 6.93 | 208 | 7.07 | 463 |
|  |  | 2008 | LRE | | R | | MM | 16.1 | 102.0 | 12.5 | 138.0 | 7.5 | 225 | 60 | 150 | 6.03 | 7.01 | 8.51 | 7.80 | 229 | 8.10 | 444 |
|  |  |  | FAS | | R | | MM | 24.6 | 143.3 | 18.3 | 80.0 | 7.4 | 150 | 60 | 120 | 6.68 | 7.04 | 7.20 | 7.10 | 109 | 7.14 | 275 |
|  |  |  | DC | | R | | MM | 16.0 | 114.0 | 17.9 | 78.0 | 7.6 | 225 | 60 | 150 | 5.96 | 7.17 | 7.88 | 7.80 | 237 | 7.86 | 474 |
|  |  |  | DC | | R | | MM | 14.7 | 98.0 | 12.4 | 84.0 | 7.3 | 150 | 60 | 120 | 5.88 | 6.15 | 6.51 | 6.41 | 128 | 6.39 | 343 |
|  |  |  | YE | | R | | MM | 16.5 | 78.0 | 12.3 | 83.0 | 7.8 | 225 | 60 | 120 | 7.46 | 7.65 | 7.76 | 7.01 | 84 | 7.72 | 216 |
|  |  |  | YE | | R | | MM | 17.2 | 106.0 | 10.5 | 76.0 | 7.9 | 150 | 60 | 120 | 4.74 | 6.09 | 6.21 | 6.18 | 144 | 6.36 | 376 |
|  |  | 2009 | YE | | R | | MM | 11.6 | 121.0 | 10.1 | 79.0 | 6.9 | 150 | 60 | 120 | 5.88 | 6.15 | 6.51 | 6.41 | 128 | 6.39 | 343 |
|  |  |  | YE | | R | | MM | 9.6 | 96.0 | 19.6 | 83.0 | 6.8 | 225 | 60 | 120 | 7.46 | 7.65 | 7.76 | 7.01 | 84 | 7.72 | 216 |
|  |  |  | LS | | R | | MM | 13.6 | 100.0 | 13.3 | 111.0 | 8.1 | 150 | 60 | 120 | 4.74 | 6.09 | 6.21 | 6.18 | 144 | 6.36 | 376 |
|  |  |  | YE | | R | | MM | 16.8 | 135.0 | 20.2 | 180.0 | 8.2 | 225 | 60 | 150 | 6.03 | 7.01 | 8.51 | 7.80 | 229 | 8.10 | 444 |
|  |  |  | CS | | R | | MM | 8.6 | 100.0 | 10.7 | 164.0 | 7.2 | 180 | 60 | 120 | 5.58 | 5.97 | 7.46 | 6.93 | 208 | 7.07 | 463 |
|  |  |  | FAS | | R | | MM | 8.7 | 89.0 | 16.1 | 84.0 | 7.4 | 180 | 60 | 120 | 7.04 | 8.06 | 10.16 | 8.91 | 182 | 9.49 | 306 |
|  |  |  | CS | | R | | MW | 13.3 | 78.0 | 13.1 | 81.0 | 7.5 | 225 | 60 | 150 | 7.02 | 7.97 | 10.08 | 8.94 | 229 | 9.42 | 382 |
|  |  |  | CS | | R | | MM | 13.3 | 102.0 | 17.1 | 168.0 | 7.0 | 180 | 60 | 120 | 7.02 | 7.91 | 8.66 | 8.75 | 227 | 8.73 | 409 |
| Handan,  Hebei | 36.3°N,  113.4°E | 2009 | FAS | | R | | MM | 24.6 | 125.0 | 22.6 | 119.0 | 6.8 | 150 | 90 | 60 | 5.63 | 7.84 | 8.69 | 7.73 | 139 | 8.62 | 270 |
|  |  |  | CS | | R | | MM | 27.9 | 110.0 | 23.5 | 187.0 | 7.2 | 180 | 120 | 90 | 4.97 | 7.53 | 8.05 | 7.92 | 185 | 8.27 | 356 |
|  |  |  | CS | | R | | MM | 21.4 | 103.0 | 14.8 | 196.0 | 7.4 | 180 | 120 | 90 | 5.88 | 7.06 | 8.06 | 7.87 | 202 | 7.97 | 400 |
|  |  |  | RE | | R | | MM | 21.3 | 104.0 | 18.6 | 74.0 | 5.9 | 150 | 90 | 60 | 4.68 | 6.88 | 7.65 | 6.86 | 141 | 7.62 | 309 |
|  |  |  | LS | | R | | MM | 24.5 | 189.0 | 23.5 | 189.0 | 7.8 | 150 | 90 | 60 | 4.89 | 7.41 | 8.25 | 7.46 | 144 | 8.25 | 290 |
|  |  |  | FAS | | R | | MM | 19.9 | 97.0 | 17.4 | 96.0 | 6.2 | 180 | 120 | 90 | 5.04 | 7.54 | 8.07 | 7.97 | 186 | 8.29 | 357 |
| Laishui,  Hebei | 39.2°N,  115.4°E | 2008 | YE | | R | | MM | 12.9 | 100.0 | 23.1 | 156.0 | 6.9 | 180 | 60 | 120 | 6.43 | 9.55 | 9.27 | 7.98 | 145 | 9.72 | 247 |
|  |  |  | YE | | R | | MM | 21.5 | 89.0 | 19.0 | 103.0 | 6.7 | 135 | 60 | 90 | 5.39 | 6.48 | 7.52 | 7.11 | 168 | 7.33 | 369 |
|  |  |  | LS | | R | | MM | 11.8 | 96.0 | 13.6 | 61.0 | 7.1 | 180 | 60 | 120 | 6.70 | 8.88 | 9.34 | 8.09 | 150 | 9.38 | 263 |
|  |  | 2009 | FAS | | R | | MM | 25.1 | 98.0 | 28.4 | 311.0 | 7.2 | 180 | 60 | 120 | 7.41 | 8.52 | 9.16 | 9.20 | 205 | 9.21 | 351 |
|  |  |  | FAS | | R | | MW | 15.7 | 97.0 | 15.3 | 112.0 | 7.6 | 135 | 60 | 90 | 5.77 | 6.89 | 7.82 | 7.82 | 187 | 7.85 | 378 |
|  |  |  | FAS | | R | | MW | 14.8 | 104.0 | 28.3 | 155.0 | 7.8 | 135 | 60 | 90 | 5.50 | 6.42 | 7.42 | 7.57 | 222 | 7.61 | 458 |
|  |  |  | FAS | | R | | MM | 22.9 | 115.0 | 22.5 | 86.0 | 8.1 | 180 | 60 | 120 | 7.19 | 8.44 | 9.19 | 8.99 | 188 | 9.14 | 326 |
|  |  |  | YE | | R | | MM | 15.4 | 106.0 | 29.1 | 125.0 | 8.3 | 180 | 60 | 120 | 7.07 | 8.31 | 9.16 | 9.17 | 213 | 9.20 | 364 |
|  |  |  | RE | | R | | MM | 15.7 | 134.0 | 15.3 | 112.0 | 6.8 | 135 | 60 | 90 | 5.56 | 6.90 | 7.56 | 7.73 | 195 | 7.72 | 399 |
|  |  |  | YE | | R | | MM | 8.5 | 89.0 | 20.3 | 81.0 | 6.7 | 180 | 60 | 120 | 7.40 | 8.24 | 8.99 | 8.60 | 171 | 8.82 | 312 |
| Pingshan,  Hebei | 38.2°N,  113.6°E | 2008 | PS | | R | | MM | 20.0 | 114.0 | 23.4 | 152.0 | 6.8 | 210 | 90 | 90 | 4.83 | 6.03 | 7.30 | 7.25 | 264 | 7.29 | 574 |
|  |  |  | YE | | R | | MM | 28.0 | 132.0 | 10.9 | 82.0 | 7.4 | 225 | 120 | 150 | 7.58 | 7.78 | 8.64 | 8.39 | 218 | 8.36 | 410 |
|  |  |  | FAS | | R | | MM | 21.5 | 106.0 | 16.8 | 56.0 | 7.6 | 210 | 135 | 120 | 7.58 | 7.78 | 8.64 | 8.39 | 211 | 8.38 | 396 |
|  |  | 2009 | FAS | | R | | MM | 28.1 | 72.0 | 29.0 | 101.0 | 6.8 | 210 | 90 | 90 | 6.13 | 6.26 | 6.61 | 6.64 | 100 | 6.33 | 293 |
|  |  |  | CLS | | R | | MM | 22.0 | 77.0 | 25.9 | 76.0 | 6.9 | 225 | 113 | 105 | 8.34 | 8.49 | 8.94 | 8.97 | 178 | 8.74 | 325 |
|  |  |  | CLS | | R | | MW | 16.0 | 110.0 | 15.0 | 145.0 | 6.4 | 210 | 135 | 120 | 7.24 | 7.31 | 7.90 | 7.78 | 164 | 7.63 | 348 |
| Wu'an,  Hebei | 36.3°N,  114.4°E | 2007 | CLS | | R | | MM | 28.4 | 88.0 | 18.1 | 131.0 | 6.2 | 135 | 45 | 90 | 6.48 | 7.05 | 7.29 | 7.20 | 138 | 7.26 | 319 |
|  |  |  | CLS | | R | | MM | 25.8 | 92.0 | 31.3 | 144.0 | 6.7 | 180 | 45 | 120 | 6.96 | 7.92 | 8.22 | 7.65 | 141 | 8.18 | 288 |
|  |  |  | BE | | R | | MM | 25.8 | 157.0 | 22.8 | 100.0 | 7.1 | 135 | 45 | 90 | 6.95 | 8.18 | 8.63 | 7.98 | 136 | 8.57 | 267 |
|  |  | 2008 | BE | | R | | MM | 25.2 | 91.0 | 33.6 | 164.0 | 7.5 | 135 | 45 | 90 | 4.40 | 5.09 | 5.95 | 5.63 | 166 | 5.76 | 465 |
|  |  |  | BE | | R | | MM | 21.7 | 117.0 | 18.5 | 158.0 | 6.3 | 180 | 45 | 120 | 7.23 | 7.58 | 7.62 | 7.62 | 102 | 7.56 | 249 |
|  |  | 2009 | BE | | R | | MM | 22.0 | 127.0 | 19.2 | 121.0 | 7.7 | 180 | 105 | 120 | 6.18 | 6.84 | 7.68 | 7.28 | 173 | 7.45 | 373 |
|  |  |  | BE | | R | | MM | 23.0 | 168.0 | 32.6 | 81.0 | 7.8 | 180 | 75 | 90 | 5.42 | 5.75 | 6.12 | 6.23 | 211 | 6.14 | 541 |
| Shijiazhuang,  Hebei | 38.0°N,  114.1°E | 2009 | Castan. | | R | | MM | 24.6 | 95.2 | 19.5 | 70.0 | 7.3 | 210 | 90 | 90 | 7.43 | 8.48 | 9.11 | 9.29 | 255 | 9.23 | 436 |
|  |  |  | Castan. | | R | | MP | 17.8 | 79.2 | 25.7 | 90.0 | 7.8 | 210 | 90 | 90 | 6.26 | 6.46 | 6.73 | 6.79 | 112 | 6.52 | 306 |
|  |  |  | Castan. | | R | | MM | 27.4 | 110.2 | 20.8 | 145.0 | 7.6 | 180 | 75 | 75 | 6.32 | 6.69 | 7.05 | 6.72 | 128 | 6.90 | 318 |
|  |  |  | Castan. | | R | | MM | 15.3 | 75.0 | 28.0 | 90.0 | 6.9 | 210 | 90 | 90 | 6.84 | 8.60 | 9.42 | 9.48 | 238 | 9.54 | 392 |
| Yixian,  Hebei | 39.2°N,  115.3°E | 2008 | Castan. | | R | | MM | 30.0 | 145.7 | 33.7 | 61.0 | 7.0 | 68 | 135 | 203 | 7.33 | 8.03 | 7.97 | 8.23 | 150 | 8.13 | 304 |
|  |  |  | FAS | | R | | MM | 28.5 | 120.0 | 9.9 | 296.0 | 6.4 | 225 | 60 | 150 | 7.83 | 7.80 | 8.17 | 6.40 | 88 | 8.15 | 211 |
| Zhangjiakou,  Hebei | 40.4°N,  114.5°E | 2008 | LCFS | | R | | MM | 18.6 | 66.0 | 18.0 | 69.0 | 6.7 | 263 | 128 | 68 | 4.65 | 5.48 | 5.40 | 5.25 | 171 | 5.45 | 504 |
|  | 40.1°N,  114.1°E | 2009 | FAS | | R | | MM | 26.8 | 209.0 | 9.0 | 157.0 | 6.3 | 200 | 95 | 75 | 6.05 | 7.86 | 8.19 | 8.09 | 196 | 8.34 | 371 |
|  |  |  | FAS | | R | | MM | 10.7 | 102.0 | 11.7 | 142.0 | 6.1 | 200 | 95 | 75 | 6.38 | 7.65 | 8.43 | 8.36 | 219 | 8.43 | 408 |
|  |  |  | FAS | | R | | MW | 20.2 | 122.0 | 10.0 | 151.0 | 6.3 | 200 | 95 | 75 | 6.84 | 8.10 | 8.61 | 8.70 | 219 | 8.71 | 395 |
| Lvliang,  Shanxi | 37.2°N,  111.5°E | 2007 | PS | | R | | MW | 7.2 | 55.0 | 14.7 | 78.0 | 7.1 | 210 | 120 | 120 | 6.09 | 7.52 | 7.74 | 6.49 | 152 | 7.79 | 320 |
|  |  |  | RE | | R | | MM | 18.6 | 44.2 | 28.2 | 125.0 | 8.1 | 210 | 120 | 120 | 8.74 | 9.24 | 9.99 | 8.99 | 148 | 9.69 | 252 |
| Changzhi,  Shanxi | 36.3°N,  113.0°E | 2006 | YE | | R | | MM | 24.4 | 159.0 | 22.4 | 121.0 | 7.3 | 210 | 120 | 120 | 6.45 | 7.05 | 7.49 | 7.55 | 218 | 7.47 | 459 |
|  |  |  | YE | | R | | MM | 26.1 | 144.0 | 16.1 | 69.0 | 7.4 | 210 | 120 | 120 | 7.19 | 8.30 | 8.87 | 9.11 | 258 | 9.04 | 451 |
|  |  | 2007 | YE | | R | | MM | 19.5 | 178.0 | 16.5 | 87.0 | 7.4 | 210 | 120 | 120 | 6.51 | 7.13 | 6.62 | 5.69 | 88 | 6.99 | 246 |
|  |  | 2008 | RE | | R | | MM | 13.3 | 96.0 | 19.3 | 110.0 | 7.3 | 225 | 60 | 150 | 8.39 | 8.97 | 9.17 | 8.73 | 145 | 9.10 | 264 |
|  |  | 2009 | LRE | | R | | MM | 13.2 | 107.0 | 14.0 | 130.0 | 7.5 | 180 | 60 | 120 | 5.52 | 8.35 | 8.77 | 8.66 | 184 | 9.06 | 323 |
|  |  |  | PS | | R | | MM | 8.2 | 71.0 | 9.3 | 120.0 | 7.4 | 225 | 60 | 150 | 5.59 | 8.49 | 8.99 | 8.81 | 226 | 9.25 | 384 |
|  |  |  | FAS | | R | | MM | 9.8 | 110.0 | 17.3 | 120.0 | 7.6 | 225 | 60 | 150 | 5.50 | 8.05 | 8.47 | 8.36 | 225 | 8.71 | 406 |
|  |  | 2010 | CS | | R | | MW | 25.0 | 158.8 | 31.8 | 237.0 | 7.8 | 180 | 60 | 120 | 5.32 | 7.87 | 8.29 | 8.18 | 183 | 8.54 | 341 |
|  |  |  | CS | | R | | MW | 28.8 | 124.0 | 24.6 | 124.0 | 7.1 | 180 | 60 | 120 | 7.41 | 8.04 | 8.54 | 8.36 | 169 | 8.44 | 322 |
|  | 36.3°N,  113.1°E | 2009 | LRE | | R | | MM | 12.1 | 89.0 | 20.0 | 150.0 | 7.9 | 225 | 60 | 150 | 5.75 | 8.47 | 8.82 | 8.80 | 228 | 9.13 | 392 |
|  |  |  | FAS | | R | | MM | 11.3 | 98.0 | 23.1 | 110.0 | 8.1 | 180 | 60 | 120 | 5.43 | 7.91 | 8.62 | 8.96 | 215 | 8.98 | 376 |
|  |  |  | DC | | R | | MM | 18.6 | 115.0 | 30.1 | 146.0 | 8.2 | 180 | 60 | 120 | 5.62 | 7.97 | 8.51 | 8.50 | 191 | 8.73 | 347 |
|  |  |  | DC | | R | | MM | 9.5 | 122.0 | 9.6 | 91.0 | 8.3 | 225 | 60 | 150 | 5.51 | 8.60 | 9.40 | 9.43 | 244 | 9.69 | 396 |
|  |  | 2010 | YE | | R | | MM | 11.1 | 114.0 | 11.4 | 102.0 | 7.6 | 180 | 60 | 120 | 5.75 | 8.11 | 8.79 | 9.06 | 210 | 9.10 | 363 |
|  |  |  | YE | | R | | MM | 12.9 | 102.0 | 12.1 | 116.0 | 7.8 | 225 | 60 | 150 | 6.11 | 9.06 | 9.59 | 9.59 | 235 | 9.89 | 374 |
|  |  |  | YE | | R | | MM | 9.6 | 114.0 | 13.2 | 57.0 | 7.4 | 180 | 60 | 120 | 5.26 | 7.78 | 8.20 | 8.69 | 213 | 8.66 | 387 |
|  |  |  | YE | | R | | MM | 13.3 | 98.0 | 15.7 | 128.0 | 7.3 | 180 | 60 | 120 | 5.61 | 7.71 | 8.80 | 8.78 | 209 | 8.90 | 370 |
|  |  |  | LS | | R | | MM | 8.2 | 89.0 | 11.4 | 132.0 | 6.8 | 180 | 60 | 120 | 5.08 | 7.70 | 8.63 | 8.91 | 216 | 8.95 | 379 |
| Jincheng,  Shanxi | 35.4°N,  113.2°E | 2007 | YE | | R | | MM | 15.1 | 96.0 | 19.5 | 130.0 | 6.4 | 210 | 120 | 120 | 7.95 | 8.40 | 8.70 | 8.15 | 131 | 8.57 | 260 |
|  |  |  | CS | | R | | MM | 16.6 | 92.0 | 28.6 | 130.0 | 6.5 | 210 | 120 | 120 | 5.55 | 6.21 | 6.58 | 5.46 | 133 | 6.48 | 348 |
|  |  |  | FAS | | R | | MM | 16.7 | 89.0 | 10.8 | 138.0 | 6.9 | 210 | 120 | 120 | 7.65 | 7.92 | 8.17 | 8.21 | 135 | 8.02 | 284 |
| Xinzhou,  Shanxi | 38.3°N,  112.6°E | 2007 | CS | | R | | MW | 14.2 | 61.0 | 10.3 | 157.0 | 6.9 | 212 | 120 | 120 | 6.22 | 8.96 | 9.70 | 9.42 | 216 | 9.86 | 344 |
|  |  |  | CS | | R | | MM | 11.2 | 59.0 | 18.3 | 75.0 | 7.2 | 210 | 120 | 120 | 7.13 | 8.60 | 9.27 | 8.78 | 195 | 9.19 | 336 |
|  | 38.2°N,  112.4°E | 2007 | FAS | | R | | MM | 21.0 | 90.0 | 21.1 | 77.0 | 6.8 | 210 | 120 | 120 | 4.16 | 5.87 | 7.46 | 5.79 | 184 | 6.98 | 419 |
|  |  |  | CS | | R | | MM | 25.1 | 115.0 | 15.6 | 203.0 | 6.7 | 210 | 120 | 120 | 5.12 | 8.66 | 8.79 | 8.58 | 204 | 9.24 | 348 |
| Pingliang,  Gansu | 35.1°N,  106.0°E | 2006 | YE | | R | | MM | 26.2 | 124.0 | 10.3 | 110.3 | 7.9 | 150 | 90 | 75 | 3.15 | 4.94 | 5.25 | 5.58 | 175 | 5.56 | 504 |
|  |  |  | LS | | R | | MM | 31.0 | 135.0 | 13.4 | 63.0 | 6.5 | 150 | 90 | 75 | 7.92 | 8.93 | 9.57 | 9.15 | 142 | 9.45 | 250 |
|  |  |  | YE | | R | | MM | 38.0 | 145.0 | 14.6 | 274.0 | 8.1 | 150 | 90 | 75 | 7.92 | 8.51 | 8.83 | 8.84 | 152 | 8.82 | 283 |
|  | 35.4°N,  107.4°E | 2006 | CS | | R | | MM | 16.0 | 30.0 | 15.2 | 90.0 | 7.7 | 83 | 36 | 135 | 5.45 | 5.76 | 5.84 | 5.94 | 114 | 5.82 | 347 |
|  |  | 2007 | FAS | | R | | MM | 26.1 | 144.0 | 16.1 | 69.0 | 7.4 | 240 | 120 | 75 | 7.40 | 7.86 | 8.64 | 8.16 | 195 | 8.34 | 370 |
|  |  |  | CS | | R | | MW | 18.0 | 130.0 | 27.6 | 56.0 | 6.4 | 240 | 120 | 45 | 7.88 | 8.96 | 9.90 | 9.26 | 216 | 9.65 | 352 |
| Tianshui,  Gansu | 34.4°N,  105.3°E | 2007 | CS | | R | | MM | 28.4 | 126.0 | 11.8 | 115.0 | 6.8 | 120 | 90 | 38 | 6.86 | 7.76 | 8.85 | 8.81 | 210 | 8.84 | 374 |
|  |  | 2008 | FAS | | R | | MM | 20.8 | 124.0 | 14.6 | 107.0 | 6.1 | 120 | 90 | 38 | 8.36 | 9.20 | 9.95 | 9.15 | 135 | 9.72 | 234 |
|  |  | 2009 | CS | | R | | MM | 29.0 | 114.0 | 17.3 | 131.0 | 6.2 | 138 | 105 | 50 | 8.90 | 9.45 | 9.45 | 9.11 | 102 | 9.48 | 198 |
| Weinan,  Shannxi | 35.1°N,  109.6°E | 2007 | CS | | R | | MM | 15.4 | 110.0 | 12.6 | 140.0 | 7.8 | 270 | 150 | 60 | 7.77 | 8.78 | 9.77 | 9.33 | 256 | 9.53 | 424 |
| **NW2 subregion** |  |  |  | |  | |  |  |  |  |  |  |  |  |  |  |  |  |  |  |  |  |
| Baotou,  Neimenggu | 40.3°N,  110.3°E | 2005 | Castan. | | | I | MM | 16.7 | 89.0 | 17.2 | 159.0 | 8.0 | 188 | 138 | 75 | 10.10 | 11.57 | 11.93 | 11.76 | 180 | 12.00 | 240 |
|  |  |  | Castan. | | | I | MM | 15.5 | 83.0 | 7.8 | 109.0 | 8.2 | 194 | 111 | 56 | 6.89 | 9.01 | 10.74 | 9.85 | 201 | 10.43 | 305 |
|  |  |  | Castan. | | | I | MM | 16.8 | 121.0 | 8.5 | 126.0 | 8.1 | 194 | 111 | 56 | 8.87 | 9.97 | 9.76 | 10.12 | 186 | 10.03 | 296 |
|  |  | 2006 | Castan. | | | I | MM | 16.3 | 108.0 | 10.7 | 135.0 | 8.4 | 194 | 111 | 56 | 6.44 | 7.30 | 8.73 | 8.18 | 208 | 8.39 | 392 |
|  |  |  | MS | | | I | MM | 13.8 | 144.0 | 7.6 | 137.0 | 8.2 | 194 | 111 | 56 | 10.08 | 10.86 | 11.52 | 11.04 | 169 | 11.34 | 241 |
|  |  | 2007 | Castan. | | | I | MM | 13.0 | 113.0 | 7.1 | 106.0 | 8.5 | 194 | 111 | 56 | 7.76 | 9.42 | 10.22 | 9.75 | 189 | 10.15 | 297 |
|  |  |  | Castan. | | | I | MM | 16.7 | 139.0 | 15.3 | 182.0 | 8.1 | 194 | 111 | 56 | 7.04 | 9.66 | 9.20 | 9.42 | 183 | 9.75 | 300 |
|  | 40.3°N,  110.4°E | 2006 | Chern. | | | I | MM | 19.8 | 140.0 | 8.5 | 157.0 | 8.2 | 194 | 111 | 56 | 8.61 | 10.95 | 12.81 | 11.48 | 190 | 12.40 | 244 |
|  |  |  | Chern. | | | I | MM | 16.2 | 120.0 | 10.2 | 130.0 | 8.3 | 194 | 111 | 56 | 9.03 | 11.34 | 11.46 | 11.85 | 212 | 11.88 | 282 |
|  |  |  | Chern. | | | I | MM | 13.6 | 191.0 | 11.6 | 151.0 | 7.9 | 194 | 111 | 56 | 8.90 | 10.96 | 11.38 | 9.85 | 152 | 11.41 | 220 |
|  |  | 2007 | Chern. | | | I | MM | 29.8 | 78.0 | 7.2 | 132.0 | 8.1 | 188 | 138 | 75 | 6.48 | 9.26 | 9.33 | 9.24 | 183 | 9.70 | 302 |
|  |  |  | Chern. | | | I | MM | 14.6 | 51.0 | 13.7 | 63.0 | 8.3 | 188 | 138 | 75 | 7.71 | 9.50 | 10.49 | 9.65 | 175 | 10.32 | 273 |
|  |  |  | Chern. | | | I | MM | 10.3 | 86.0 | 14.1 | 66.0 | 8.3 | 188 | 138 | 75 | 6.75 | 10.80 | 11.78 | 10.80 | 182 | 11.94 | 244 |
|  |  | 2008 | Castan. | | | I | MM | 16.0 | 131.0 | 12.2 | 78.0 | 8.4 | 188 | 138 | 75 | 7.80 | 10.68 | 11.22 | 10.46 | 174 | 11.37 | 247 |
|  |  |  | MS | | | I | MM | 15.7 | 60.0 | 12.2 | 93.0 | 8.2 | 188 | 138 | 75 | 9.71 | 11.01 | 11.18 | 11.27 | 186 | 11.33 | 262 |
|  |  | 2009 | MS | | | I | MM | 24.1 | 69.0 | 12.9 | 99.0 | 8.2 | 188 | 138 | 75 | 9.78 | 11.84 | 12.77 | 12.15 | 184 | 12.70 | 232 |
|  |  |  | Castan. | | | I | MM | 18.1 | 66.0 | 14.3 | 81.0 | 8.2 | 188 | 138 | 75 | 10.69 | 13.48 | 13.29 | 13.15 | 174 | 13.73 | 204 |
| Bayanzuo'er,  Neimenggu | 40.5°N,  107.1°E | 2006 | MS | | | I | MM | 10.0 | 90.0 | 8.0 | 108.0 | 7.6 | 207 | 156 | 45 | 8.96 | 10.91 | 11.80 | 11.95 | 244 | 11.98 | 322 |
|  |  |  | MS | | | I | MM | 10.0 | 111.0 | 18.0 | 137.0 | 7.5 | 207 | 156 | 45 | 8.82 | 11.21 | 12.18 | 11.77 | 212 | 12.20 | 274 |
|  |  | 2007 | MS | | | I | MM | 10.0 | 104.0 | 12.0 | 127.0 | 7.2 | 207 | 156 | 75 | 10.64 | 12.07 | 13.20 | 11.78 | 173 | 12.87 | 217 |
|  |  | 2008 | MS | | | I | MM | 19.0 | 103.0 | 10.2 | 130.0 | 7.4 | 207 | 156 | 45 | 8.94 | 11.31 | 12.36 | 12.59 | 251 | 12.62 | 315 |
|  |  |  | Chern. | | | I | MM | 15.0 | 91.4 | 9.9 | 129.0 | 7.4 | 207 | 156 | 45 | 10.07 | 10.89 | 11.29 | 11.31 | 207 | 11.30 | 290 |
|  |  |  | Chern. | | | I | MM | 12.0 | 100.3 | 13.2 | 125.0 | 7.3 | 207 | 156 | 45 | 9.54 | 11.74 | 12.68 | 11.86 | 195 | 12.59 | 246 |
|  |  |  | DBE | | | I | MM | 13.0 | 95.6 | 18.6 | 125.0 | 7.6 | 207 | 156 | 45 | 8.39 | 10.18 | 10.99 | 11.07 | 236 | 11.12 | 335 |
|  |  | 2009 | DBE | | | I | MM | 20.0 | 99.6 | 9.8 | 123.0 | 7.5 | 207 | 156 | 75 | 6.41 | 9.45 | 9.43 | 9.53 | 206 | 9.91 | 329 |
|  |  |  | MS | | | I | MM | 20.0 | 110.5 | 10.6 | 134.0 | 7.3 | 207 | 156 | 75 | 7.84 | 12.41 | 12.47 | 12.04 | 199 | 13.06 | 242 |
|  |  |  | Chern. | | | I | MM | 21.0 | 120.3 | 15.6 | 142.0 | 7.2 | 207 | 156 | 75 | 8.15 | 11.67 | 10.99 | 11.36 | 200 | 11.77 | 269 |
|  |  |  | DBE | | | I | MM | 15.0 | 98.2 | 11.0 | 110.0 | 7.5 | 207 | 156 | 75 | 11.48 | 11.75 | 12.55 | 12.33 | 207 | 12.32 | 266 |
|  | 40.5°N,  108.4°E | 2006 | DBE | | | I | MM | 36.6 | 110.0 | 12.1 | 150.0 | 8.1 | 207 | 156 | 75 | 9.11 | 9.68 | 9.62 | 9.14 | 114 | 9.67 | 210 |
|  |  |  | DBE | | | I | MM | 23.6 | 106.0 | 9.6 | 160.0 | 8.1 | 207 | 156 | 75 | 7.73 | 9.72 | 9.77 | 10.17 | 224 | 10.16 | 348 |
|  |  | 2007 | AS | | | I | MM | 20.1 | 165.0 | 9.5 | 110.0 | 8.1 | 207 | 156 | 75 | 8.12 | 10.05 | 10.23 | 10.50 | 220 | 10.54 | 329 |
|  |  |  | CS | | | I | MM | 23.0 | 121.0 | 17.3 | 146.0 | 7.8 | 207 | 156 | 75 | 10.11 | 10.99 | 11.59 | 11.04 | 173 | 11.43 | 244 |
|  |  | 2008 | CS | | | I | MM | 30.6 | 98.0 | 24.9 | 200.0 | 7.4 | 207 | 156 | 75 | 9.76 | 10.64 | 11.45 | 11.03 | 195 | 11.25 | 275 |
|  |  |  | CS | | | I | MM | 23.2 | 75.0 | 19.5 | 132.0 | 7.5 | 207 | 156 | 75 | 10.35 | 11.74 | 12.27 | 12.29 | 219 | 12.35 | 280 |
|  | 40.4°N,  108.4°E | 2009 | CS | | | I | MM | 21.2 | 125.0 | 23.2 | 133.0 | 6.3 | 207 | 156 | 75 | 9.01 | 10.45 | 10.27 | 10.22 | 173 | 10.50 | 266 |
|  |  |  | CS | | | I | MM | 29.3 | 136.0 | 14.3 | 200.0 | 8.0 | 207 | 156 | 75 | 10.31 | 10.88 | 10.91 | 10.93 | 145 | 10.91 | 222 |
|  |  | 2010 | DBE | | | I | MM | 22.3 | 114.0 | 22.4 | 125.0 | 8.0 | 207 | 156 | 75 | 8.78 | 10.20 | 10.29 | 10.56 | 215 | 10.54 | 322 |
|  |  |  | DBE | | | I | MM | 27.4 | 123.0 | 18.1 | 104.0 | 7.4 | 207 | 156 | 75 | 6.13 | 7.56 | 7.41 | 5.42 | 129 | 7.70 | 288 |
|  |  |  | DBE | | | I | MM | 21.6 | 98.0 | 20.4 | 133.0 | 7.9 | 207 | 156 | 75 | 9.71 | 11.15 | 11.44 | 11.65 | 222 | 11.64 | 301 |
|  |  |  | CS | | | I | MM | 22.3 | 78.0 | 14.7 | 133.0 | 7.8 | 207 | 156 | 75 | 7.76 | 9.29 | 9.38 | 9.70 | 220 | 9.66 | 359 |
|  |  |  | CS | | | I | MM | 20.9 | 116.0 | 25.8 | 139.0 | 6.4 | 207 | 156 | 75 | 9.37 | 10.29 | 11.45 | 10.84 | 202 | 11.14 | 287 |
|  |  |  | CS | | | I | MM | 30.2 | 125.0 | 18.0 | 155.0 | 7.4 | 207 | 156 | 75 | 8.95 | 9.51 | 10.93 | 10.40 | 224 | 10.55 | 335 |
|  | 41.0°N,  108.2°E | 2006 | CS | | | I | MM | 20.4 | 163.0 | 21.6 | 193.0 | 6.8 | 240 | 173 | 38 | 6.90 | 7.50 | 8.04 | 7.71 | 226 | 7.81 | 456 |
|  |  |  | ASS | | | I | MM | 31.1 | 154.0 | 13.8 | 93.0 | 7.9 | 240 | 173 | 38 | 7.50 | 7.97 | 8.58 | 8.03 | 202 | 8.28 | 386 |
|  |  |  | ASS | | | I | MM | 33.6 | 150.0 | 16.5 | 112.0 | 7.4 | 240 | 173 | 38 | 6.78 | 9.57 | 10.01 | 8.58 | 252 | 10.11 | 395 |
|  |  |  | MS | | | I | MM | 26.7 | 124.0 | 11.9 | 133.0 | 6.1 | 240 | 173 | 38 | 8.70 | 9.45 | 10.06 | 9.60 | 236 | 9.84 | 378 |
| Chifeng,  Neimenggu | 43.5°N,  120.0°E | 2007 | MS | | | I | MM | 15.6 | 98.0 | 15.0 | 121.0 | 7.2 | 180 | 138 | 75 | 9.23 | 10.49 | 11.01 | 10.68 | 169 | 10.98 | 249 |
|  |  |  | Chern. | | | I | MM | 12.6 | 69.0 | 14.0 | 141.0 | 8.4 | 180 | 138 | 75 | 10.36 | 11.08 | 11.54 | 11.37 | 168 | 11.46 | 237 |
|  |  | 2008 | Chern. | | | I | MM | 13.6 | 95.0 | 21.0 | 132.0 | 7.5 | 180 | 138 | 75 | 12.00 | 12.78 | 13.18 | 12.93 | 158 | 13.11 | 197 |
|  |  |  | Chern. | | | I | MM | 16.3 | 110.0 | 16.0 | 121.0 | 8.0 | 180 | 138 | 75 | 4.78 | 7.88 | 8.05 | 8.08 | 183 | 8.48 | 345 |
|  |  |  | Chern. | | | I | MM | 15.4 | 124.0 | 12.0 | 108.0 | 8.1 | 180 | 138 | 75 | 6.45 | 8.85 | 9.78 | 9.33 | 184 | 9.80 | 300 |
|  |  | 2009 | Chern. | | | I | MM | 13.5 | 136.0 | 21.0 | 106.0 | 6.2 | 180 | 138 | 75 | 7.68 | 8.70 | 10.20 | 9.58 | 193 | 9.85 | 312 |
|  |  |  | Chern. | | | I | MM | 14.8 | 110.0 | 15.0 | 114.0 | 8.1 | 180 | 138 | 75 | 6.88 | 9.93 | 12.61 | 11.51 | 199 | 12.18 | 259 |
|  |  |  | Chern. | | | I | MM | 15.9 | 152.0 | 16.0 | 150.0 | 8.1 | 180 | 138 | 75 | 8.45 | 10.28 | 11.70 | 10.20 | 161 | 11.32 | 232 |
|  |  |  | AS | | | I | MM | 16.5 | 114.0 | 18.0 | 136.0 | 8.1 | 180 | 138 | 75 | 11.82 | 12.67 | 13.62 | 12.85 | 159 | 13.32 | 195 |
|  | 42.1°N,  119.6°E | 2006 | CS | | | I | MM | 14.8 | 124.0 | 21.0 | 132.0 | 7.8 | 180 | 113 | 75 | 8.00 | 9.81 | 11.46 | 11.51 | 241 | 11.56 | 329 |
|  |  |  | CS | | | I | MM | 18.2 | 136.0 | 23.0 | 125.0 | 7.4 | 180 | 113 | 75 | 5.08 | 6.43 | 7.91 | 7.61 | 214 | 7.75 | 436 |
|  |  | 2007 | BE | | | I | MM | 10.9 | 114.0 | 21.0 | 142.0 | 7.5 | 180 | 113 | 75 | 8.22 | 10.48 | 10.21 | 10.35 | 171 | 10.64 | 260 |
|  |  |  | Castan. | | | I | MM | 9.6 | 109.0 | 18.0 | 89.0 | 6.3 | 180 | 113 | 75 | 6.59 | 7.08 | 7.17 | 7.19 | 137 | 7.16 | 324 |
|  |  | 2008 | MS | | | I | MM | 18.9 | 137.0 | 17.0 | 96.0 | 8.3 | 180 | 113 | 75 | 8.34 | 9.01 | 8.79 | 8.70 | 112 | 8.91 | 226 |
|  |  | 2009 | DBE | | | I | MM | 14.4 | 98.0 | 15.0 | 82.0 | 7.1 | 180 | 113 | 75 | 10.41 | 10.97 | 11.10 | 10.95 | 133 | 11.07 | 205 |
|  |  |  | Castan. | | | I | MM | 13.5 | 89.0 | 13.0 | 68.0 | 8.1 | 180 | 113 | 75 | 10.17 | 10.95 | 11.10 | 11.15 | 165 | 11.15 | 240 |
|  | 43.3°N,  118.4°E | 2008 | BS | | | I | MM | 21.2 | 88.0 | 14.0 | 121.0 | 8.5 | 180 | 90 | 75 | 6.95 | 8.62 | 9.86 | 8.47 | 158 | 9.53 | 272 |
|  |  |  | DBE | | | I | MM | 18.9 | 78.0 | 15.0 | 100.0 | 7.0 | 180 | 90 | 75 | 6.57 | 7.36 | 7.85 | 7.88 | 196 | 7.87 | 395 |
|  |  |  | MS | | | I | MM | 16.9 | 110.0 | 8.9 | 106.0 | 6.3 | 180 | 90 | 75 | 7.00 | 8.46 | 8.95 | 9.16 | 208 | 9.14 | 360 |
|  |  | 2009 | BS | | | I | MM | 17.8 | 121.0 | 14.1 | 153.0 | 7.9 | 180 | 90 | 75 | 8.81 | 9.74 | 10.02 | 9.57 | 144 | 9.99 | 241 |
|  |  |  | BS | | | I | MM | 16.5 | 114.0 | 15.0 | 120.0 | 8.3 | 180 | 90 | 75 | 7.26 | 7.93 | 8.58 | 7.73 | 138 | 8.36 | 279 |
|  |  |  | MS | | | I | MM | 14.5 | 145.0 | 13.2 | 87.0 | 8.2 | 180 | 90 | 75 | 8.58 | 9.78 | 10.01 | 10.42 | 223 | 10.32 | 341 |
|  |  | 2010 | DBE | | | I | MM | 16.5 | 136.0 | 12.3 | 89.0 | 8.5 | 180 | 90 | 75 | 8.50 | 9.87 | 10.12 | 10.57 | 225 | 10.47 | 339 |
|  |  |  | BS | | | I | MM | 13.8 | 114.0 | 13.2 | 110.0 | 8.4 | 180 | 90 | 75 | 7.00 | 8.46 | 8.95 | 9.16 | 208 | 9.14 | 360 |
|  | 43.6°N,  119.2°E | 2005 | AS | | | I | MM | 15.6 | 102.0 | 14.5 | 154.0 | 8.3 | 188 | 138 | 75 | 6.83 | 8.45 | 8.64 | 6.30 | 125 | 8.79 | 247 |
|  |  | 2006 | ASS | | | I | MM | 14.8 | 98.0 | 16.5 | 112.0 | 8.3 | 188 | 138 | 75 | 7.85 | 9.44 | 9.24 | 9.11 | 159 | 9.50 | 274 |
|  |  | 2007 | AS | | | I | MM | 16.4 | 98.0 | 15.6 | 420.0 | 8.1 | 188 | 138 | 75 | 8.94 | 10.31 | 11.34 | 11.43 | 237 | 11.44 | 327 |
|  |  | 2008 | AS | | | I | MM | 15.2 | 87.0 | 17.8 | 89.0 | 8.4 | 188 | 138 | 75 | 9.83 | 10.89 | 11.45 | 10.80 | 158 | 11.32 | 229 |
|  |  | 2009 | AS | | | I | MM | 14.7 | 110.0 | 16.2 | 78.0 | 8.4 | 188 | 138 | 75 | 7.17 | 12.26 | 12.56 | 11.76 | 179 | 13.10 | 219 |
|  | 43.6°N,  119.3°E | 2007 | AS | | | I | MM | 18.9 | 136.0 | 13.3 | 110.0 | 7.5 | 188 | 138 | 75 | 8.48 | 11.19 | 11.69 | 10.64 | 166 | 11.79 | 229 |
|  |  | 2008 | AS | | | I | MM | 16.8 | 145.0 | 11.4 | 96.0 | 8.0 | 188 | 138 | 75 | 8.85 | 11.28 | 12.06 | 11.34 | 178 | 12.07 | 237 |
|  |  | 2009 | AS | | | I | MM | 17.0 | 114.0 | 16.5 | 89.0 | 7.4 | 188 | 138 | 75 | 11.51 | 13.17 | 13.46 | 12.17 | 143 | 13.50 | 177 |
|  |  | 2010 | AS | | | I | MM | 14.4 | 95.0 | 14.3 | 76.0 | 7.2 | 188 | 138 | 75 | 11.13 | 12.65 | 13.22 | 12.11 | 152 | 13.13 | 191 |
|  | 41.5°N,  118.4°E | 2007 | MS | | | I | MM | 25.6 | 135.0 | 13.1 | 149.0 | 7.5 | 188 | 108 | 75 | 12.27 | 13.02 | 12.69 | 12.20 | 103 | 12.91 | 148 |
|  |  |  | AS | | | I | MM | 24.9 | 93.0 | 12.3 | 151.0 | 7.6 | 188 | 108 | 38 | 9.88 | 11.19 | 12.76 | 12.13 | 202 | 12.43 | 257 |
|  |  | 2008 | AS | | | I | MM | 7.0 | 100.0 | 7.0 | 120.0 | 8.1 | 188 | 117 | 60 | 8.51 | 10.06 | 12.21 | 11.45 | 214 | 11.79 | 287 |
|  |  |  | BE | | | I | MM | 12.0 | 98.0 | 6.8 | 130.0 | 8.2 | 188 | 108 | 75 | 4.44 | 7.63 | 8.57 | 4.88 | 142 | 8.53 | 279 |
|  |  |  | Castan. | | | I | MM | 13.0 | 126.0 | 9.2 | 120.0 | 7.6 | 188 | 108 | 75 | 8.53 | 9.06 | 9.57 | 9.55 | 196 | 9.51 | 327 |
|  |  | 2009 | MS | | | I | MM | 15.0 | 108.4 | 9.5 | 200.0 | 6.9 | 188 | 108 | 75 | 8.51 | 9.56 | 10.39 | 9.39 | 155 | 10.15 | 251 |
|  |  |  | Castan. | | | I | MM | 13.0 | 108.4 | 9.5 | 120.0 | 6.9 | 188 | 108 | 75 | 10.38 | 11.63 | 12.00 | 11.39 | 155 | 11.97 | 213 |
|  |  |  | Chern. | | | I | MM | 16.0 | 100.4 | 7.9 | 110.0 | 6.8 | 188 | 108 | 75 | 7.80 | 10.19 | 12.61 | 12.10 | 227 | 12.38 | 289 |
|  | 41.6°N,  118.4°E | 2009 | Castan. | | | I | MM | 15.0 | 100.0 | 7.0 | 118.0 | 7.3 | 188 | 108 | 75 | 10.81 | 11.59 | 13.11 | 12.69 | 222 | 12.83 | 273 |
|  |  |  | BS | | | I | MM | 11.0 | 112.4 | 8.7 | 110.0 | 7.5 | 188 | 108 | 75 | 11.63 | 11.88 | 12.13 | 12.13 | 128 | 11.99 | 184 |
|  |  |  | MS | | | I | MM | 12.0 | 120.0 | 8.1 | 100.0 | 7.6 | 188 | 108 | 75 | 11.88 | 12.82 | 13.76 | 13.60 | 212 | 13.66 | 245 |
|  |  | 2010 | Castan. | | | I | MM | 11.5 | 116.0 | 7.1 | 110.0 | 8.1 | 188 | 117 | 60 | 9.76 | 12.62 | 12.50 | 12.01 | 167 | 12.89 | 210 |
|  |  |  | DBE | | | I | MM | 13.4 | 102.0 | 9.8 | 130.0 | 8.2 | 188 | 117 | 60 | 11.10 | 11.50 | 11.60 | 10.95 | 100 | 11.58 | 161 |
|  |  |  | Castan. | | | I | MM | 14.7 | 91.0 | 6.5 | 70.0 | 7.5 | 188 | 117 | 60 | 11.02 | 13.10 | 12.96 | 12.48 | 158 | 13.24 | 195 |
|  | 41.6°N,  118.5°E | 2005 | CS | | | I | MM | 10.4 | 95.0 | 15.4 | 170.0 | 7.6 | 188 | 117 | 60 | 12.73 | 13.08 | 13.92 | 13.49 | 169 | 13.63 | 201 |
|  |  | 2006 | CS | | | I | MM | 22.4 | 130.0 | 6.4 | 150.0 | 7.8 | 188 | 138 | 75 | 10.55 | 14.31 | 12.64 | 11.48 | 138 | 13.78 | 169 |
|  |  | 2007 | Castan. | | | I | MM | 18.6 | 154.0 | 27.1 | 162.0 | 6.8 | 188 | 138 | 75 | 9.22 | 10.48 | 10.66 | 9.77 | 140 | 10.70 | 220 |
|  |  | 2008 | CS | | | I | MM | 15.2 | 168.0 | 7.1 | 60.0 | 6.9 | 188 | 138 | 75 | 10.02 | 13.11 | 12.34 | 10.29 | 134 | 13.04 | 175 |
|  |  | 2009 | Castan. | | | I | MM | 15.0 | 143.3 | 20.3 | 115.0 | 6.5 | 188 | 138 | 75 | 8.13 | 9.73 | 11.27 | 10.12 | 178 | 10.87 | 263 |
|  |  | 2010 | CS | | | I | MM | 14.0 | 168.2 | 25.6 | 128.0 | 7.8 | 188 | 138 | 75 | 9.58 | 10.07 | 10.84 | 10.72 | 210 | 10.72 | 310 |
|  | 43.2°N,  117.3°E | 2008 | CS | | | I | MM | 14.6 | 98.0 | 8.9 | 116.0 | 8.3 | 165 | 90 | 75 | 5.10 | 6.40 | 7.87 | 7.16 | 173 | 7.54 | 370 |
|  |  |  | MS | | | I | MM | 19.6 | 122.0 | 21.3 | 153.0 | 7.1 | 165 | 90 | 75 | 7.67 | 9.93 | 11.47 | 9.64 | 147 | 11.08 | 221 |
|  |  |  | MS | | | I | MM | 13.7 | 79.0 | 17.3 | 123.0 | 8.2 | 165 | 90 | 75 | 4.85 | 6.02 | 7.70 | 7.08 | 185 | 7.35 | 402 |
|  |  |  | CS | | | I | MM | 13.4 | 68.0 | 28.3 | 153.0 | 8.5 | 165 | 90 | 75 | 4.65 | 6.27 | 7.72 | 7.31 | 187 | 7.55 | 395 |
|  |  | 2009 | MS | | | I | MM | 17.6 | 102.0 | 17.3 | 133.0 | 7.0 | 165 | 90 | 75 | 5.51 | 6.81 | 8.10 | 7.11 | 155 | 7.75 | 329 |
|  |  |  | ISS | | | I | MM | 17.6 | 122.0 | 24.9 | 125.0 | 6.3 | 165 | 90 | 75 | 5.20 | 6.60 | 7.85 | 7.38 | 177 | 7.65 | 372 |
|  |  |  | ISS | | | I | MM | 28.9 | 58.0 | 13.4 | 178.0 | 7.9 | 165 | 90 | 75 | 5.26 | 7.11 | 8.53 | 8.63 | 217 | 8.67 | 395 |
|  |  | 2010 | ISS | | | I | MM | 25.3 | 74.0 | 18.5 | 157.0 | 8.0 | 165 | 90 | 75 | 6.02 | 6.98 | 7.80 | 7.36 | 161 | 7.63 | 344 |
|  |  |  | ISS | | | I | MM | 10.6 | 96.0 | 11.9 | 129.0 | 8.1 | 165 | 90 | 75 | 7.23 | 7.82 | 9.85 | 9.16 | 202 | 9.37 | 341 |
|  |  |  | BS | | | I | MM | 12.5 | 98.0 | 23.5 | 119.0 | 8.0 | 165 | 90 | 75 | 6.96 | 9.32 | 11.25 | 11.12 | 208 | 11.26 | 292 |
|  | 43.4°N,  118.1°E | 2005 | Chern. | | | I | MM | 23.1 | 85.0 | 9.2 | 146.0 | 8.2 | 180 | 90 | 75 | 4.78 | 6.79 | 9.11 | 7.93 | 191 | 8.57 | 355 |
|  |  |  | BS | | | I | MM | 30.6 | 107.0 | 9.6 | 145.0 | 8.1 | 180 | 90 | 75 | 5.48 | 7.09 | 8.93 | 7.81 | 181 | 8.45 | 343 |
|  |  | 2006 | MS | | | I | MM | 31.5 | 104.0 | 8.6 | 109.0 | 8.2 | 180 | 90 | 75 | 4.61 | 6.38 | 8.64 | 7.01 | 175 | 7.97 | 353 |
|  |  |  | DBE | | | I | MM | 28.6 | 119.0 | 9.9 | 117.0 | 8.4 | 180 | 90 | 75 | 6.09 | 8.82 | 11.64 | 10.50 | 201 | 11.13 | 286 |
|  |  | 2007  2007 | AS | | | I | MM | 24.2 | 85.0 | 10.2 | 176.0 | 8.1 | 180 | 90 | 75 | 4.76 | 5.78 | 8.21 | 6.97 | 192 | 7.48 | 408 |
|  |  |  | AS | | | I | MM | 23.1 | 91.0 | 10.4 | 162.0 | 8.3 | 180 | 90 | 75 | 4.56 | 5.79 | 7.45 | 6.79 | 197 | 7.09 | 441 |
|  |  | 2008  2008 | BS | | | I | MM | 26.0 | 99.0 | 14.0 | 104.0 | 8.1 | 180 | 90 | 75 | 4.97 | 6.04 | 8.06 | 7.48 | 218 | 7.68 | 448 |
|  |  |  | ISS | | | I | MM | 24.3 | 103.0 | 10.7 | 197.0 | 8.4 | 180 | 90 | 75 | 5.73 | 6.78 | 8.98 | 7.65 | 181 | 8.28 | 350 |
|  | 43.4°N,  118.2°E | 2008 | ISS | | | I | MM | 20.8 | 195.0 | 23.8 | 146.0 | 8.3 | 180 | 90 | 75 | 5.53 | 7.70 | 9.44 | 6.74 | 148 | 8.92 | 276 |
|  |  |  | ISS | | | I | MM | 21.6 | 157.0 | 11.8 | 183.0 | 7.4 | 180 | 90 | 75 | 5.17 | 7.15 | 9.00 | 7.66 | 175 | 8.53 | 330 |
|  |  |  | ISS | | | I | MM | 26.3 | 150.0 | 16.5 | 112.0 | 7.4 | 180 | 90 | 75 | 5.55 | 6.45 | 9.06 | 7.81 | 198 | 8.28 | 379 |
|  |  |  | ISS | | | I | MM | 37.6 | 193.0 | 11.9 | 133.0 | 6.1 | 180 | 90 | 75 | 5.72 | 7.25 | 9.03 | 8.26 | 194 | 8.65 | 356 |
|  |  | 2009 | ISS | | | I | MM | 25.3 | 182.0 | 11.6 | 133.0 | 7.7 | 180 | 90 | 75 | 4.73 | 7.74 | 8.49 | 7.86 | 176 | 8.62 | 328 |
|  |  |  | ISS | | | I | MM | 28.4 | 187.9 | 15.9 | 160.0 | 6.1 | 180 | 90 | 75 | 4.58 | 5.87 | 8.06 | 5.97 | 159 | 7.28 | 357 |
|  |  |  | ISS | | | I | MM | 20.7 | 135.4 | 13.4 | 133.0 | 6.5 | 180 | 90 | 75 | 4.88 | 8.06 | 10.02 | 9.63 | 207 | 9.99 | 328 |
|  |  |  | Castan. | | | I | MM | 20.3 | 114.0 | 24.3 | 110.0 | 6.9 | 180 | 90 | 75 | 6.11 | 7.92 | 9.49 | 9.06 | 204 | 9.32 | 346 |
|  |  | 2010 | ASS | | | I | MM | 29.9 | 151.0 | 22.0 | 105.0 | 6.6 | 180 | 90 | 75 | 5.78 | 8.43 | 9.79 | 8.97 | 182 | 9.66 | 302 |
|  |  |  | Castan. | | | I | MM | 28.5 | 121.0 | 18.0 | 118.0 | 6.0 | 180 | 90 | 75 | 5.86 | 8.15 | 10.50 | 9.97 | 217 | 10.25 | 334 |
|  |  |  | Castan. | | | I | MM | 23.1 | 96.0 | 19.0 | 191.0 | 6.7 | 180 | 90 | 75 | 6.39 | 8.67 | 11.10 | 7.50 | 147 | 10.30 | 237 |
|  |  |  | MS | | | I | MM | 31.6 | 89.0 | 23.6 | 195.0 | 6.1 | 180 | 90 | 75 | 7.19 | 8.55 | 10.01 | 7.28 | 134 | 9.53 | 239 |
|  | 41.3°N,  119.2°E | 2008 | CS | | | I | MM | 5.0 | 114.0 | 13.9 | 64.0 | 8.3 | 188 | 113 | 75 | 9.22 | 10.85 | 10.96 | 10.48 | 158 | 11.09 | 233 |
|  |  |  | CS | | | I | MM | 8.4 | 73.0 | 16.3 | 175.0 | 8.3 | 188 | 113 | 75 | 4.58 | 6.57 | 6.91 | 6.46 | 172 | 7.02 | 395 |
|  | 42.5°N,  119.2°E | 2008 | CS | | | I | MM | 22.3 | 89.0 | 21.9 | 157.0 | 6.0 | 188 | 113 | 75 | 9.45 | 11.11 | 11.73 | 11.51 | 189 | 11.76 | 256 |
|  |  |  | CS | | | I | MM | 23.6 | 98.0 | 24.0 | 189.0 | 6.9 | 188 | 113 | 75 | 7.88 | 9.67 | 11.71 | 11.25 | 226 | 11.47 | 311 |
|  |  | 2009 | Castan. | | | I | MM | 20.3 | 115.0 | 18.3 | 161.0 | 6.6 | 188 | 113 | 75 | 9.82 | 12.08 | 12.28 | 12.52 | 200 | 12.62 | 251 |
|  |  |  | MS | | | I | MM | 22.8 | 125.0 | 26.2 | 131.0 | 6.9 | 188 | 113 | 75 | 10.52 | 11.51 | 11.78 | 11.51 | 160 | 11.77 | 222 |
|  |  |  | Castan. | | | I | MM | 21.4 | 131.0 | 27.2 | 121.0 | 6.5 | 188 | 113 | 75 | 10.14 | 10.85 | 11.36 | 11.12 | 171 | 11.25 | 246 |
|  |  |  | CS | | | I | MM | 20.2 | 89.0 | 24.4 | 132.0 | 6.5 | 188 | 113 | 75 | 9.21 | 12.64 | 13.25 | 11.36 | 159 | 13.36 | 195 |
|  |  | 2010 | Castan. | | | I | MM | 20.3 | 96.0 | 7.7 | 198.0 | 5.7 | 188 | 113 | 75 | 11.20 | 11.89 | 12.20 | 11.01 | 119 | 12.14 | 173 |
|  |  |  | Castan. | | | I | MM | 27.5 | 94.0 | 17.7 | 200.0 | 6.3 | 188 | 113 | 75 | 9.53 | 10.61 | 12.03 | 11.42 | 198 | 11.70 | 268 |
|  |  |  | Castan. | | | I | MM | 22.7 | 120.0 | 19.1 | 111.0 | 5.9 | 188 | 113 | 75 | 10.45 | 12.10 | 12.72 | 12.41 | 184 | 12.73 | 231 |
| E'erduosi,  Neimenggu | 40.2°N,  110.1°E | 2006 | MS | | | I | MM | 14.0 | 60.0 | 17.4 | 80.0 | 7.8 | 207 | 156 | 75 | 7.43 | 9.62 | 9.58 | 9.24 | 182 | 9.85 | 296 |
|  |  |  | MS | | | I | MM | 25.6 | 147.0 | 38.1 | 104.0 | 7.9 | 207 | 156 | 75 | 10.61 | 12.07 | 12.56 | 10.61 | 145 | 12.52 | 193 |
|  |  |  | CLS | | | I | MM | 22.2 | 156.9 | 8.8 | 125.0 | 7.6 | 207 | 156 | 75 | 6.63 | 8.94 | 10.96 | 10.07 | 221 | 10.61 | 329 |
|  |  | 2007 | MS | | | I | MM | 20.0 | 209.0 | 31.0 | 120.0 | 7.4 | 240 | 156 | 75 | 11.93 | 12.13 | 12.42 | 10.64 | 115 | 12.42 | 165 |
|  |  | 2008 | MS | | | I | MM | 22.0 | 132.0 | 29.0 | 95.0 | 8.1 | 240 | 156 | 75 | 9.00 | 9.85 | 10.77 | 10.37 | 252 | 10.53 | 379 |
|  |  | 2009 | AS | | | I | MM | 22.0 | 165.0 | 12.0 | 109.0 | 7.6 | 240 | 156 | 75 | 10.61 | 11.71 | 11.61 | 11.26 | 185 | 11.72 | 252 |
|  |  | 2010 | PS | | | I | MM | 22.0 | 170.0 | 19.0 | 111.0 | 7.7 | 240 | 156 | 75 | 9.29 | 11.61 | 11.67 | 10.92 | 222 | 11.89 | 295 |
|  | 38.2°N,  107.3°E | 2008 | AS | | | I | MM | 25.0 | 127.0 | 21.0 | 136.0 | 8.2 | 188 | 138 | 75 | 4.93 | 7.98 | 10.74 | 8.38 | 179 | 10.01 | 287 |
|  |  |  | AS | | | I | MM | 23.0 | 269.0 | 16.2 | 115.0 | 8.4 | 188 | 138 | 75 | 9.33 | 10.07 | 10.84 | 10.72 | 207 | 10.75 | 305 |
|  |  | 2009 | AS | | | I | MM | 21.0 | 178.0 | 17.0 | 107.0 | 7.5 | 188 | 138 | 75 | 6.38 | 6.78 | 6.87 | 6.20 | 99 | 6.86 | 271 |
|  |  |  | DBE | | | I | MM | 20.0 | 140.0 | 15.0 | 114.0 | 7.6 | 188 | 138 | 75 | 6.12 | 9.08 | 8.78 | 9.62 | 214 | 9.53 | 355 |
|  |  |  | AS | | | I | MM | 29.0 | 80.0 | 27.0 | 105.0 | 7.3 | 188 | 138 | 75 | 6.98 | 7.31 | 7.71 | 7.49 | 141 | 7.54 | 314 |
|  |  |  | BE | | | I | MM | 14.0 | 106.6 | 26.1 | 129.0 | 7.1 | 188 | 138 | 75 | 6.74 | 8.55 | 8.60 | 8.55 | 177 | 8.83 | 322 |
|  |  | 2010 | Chern. | | | I | MP | 15.0 | 110.3 | 25.2 | 119.0 | 7.0 | 188 | 138 | 75 | 6.87 | 7.71 | 8.64 | 7.85 | 163 | 8.35 | 318 |
|  |  |  | Chern. | | | I | MM | 13.0 | 117.6 | 25.9 | 108.0 | 7.3 | 188 | 138 | 75 | 8.91 | 9.98 | 10.59 | 8.42 | 124 | 10.48 | 206 |
|  |  |  | BS | | | I | MM | 29.0 | 167.0 | 20.1 | 121.0 | 7.4 | 188 | 138 | 75 | 9.44 | 10.28 | 10.14 | 9.35 | 114 | 10.29 | 198 |
|  | 39.5°N,  108.4°E | 2007 | BE | | | I | MM | 20.0 | 141.0 | 16.1 | 67.0 | 7.4 | 240 | 144 | 75 | 8.54 | 9.17 | 9.56 | 9.63 | 247 | 9.50 | 411 |
|  |  |  | BS | | | I | MM | 15.0 | 178.0 | 15.8 | 130.0 | 6.8 | 240 | 144 | 75 | 10.67 | 11.33 | 11.50 | 11.38 | 189 | 11.43 | 264 |
|  |  | 2008 | BS | | | I | MM | 13.0 | 116.0 | 16.0 | 150.0 | 6.9 | 240 | 138 | 75 | 11.90 | 13.19 | 13.78 | 13.22 | 233 | 13.67 | 269 |
|  | 38.3°N,  108.5°E | 2007 | AS | | | I | MP | 26.1 | 117.0 | 10.5 | 146.0 | 6.8 | 207 | 156 | 75 | 6.25 | 7.72 | 8.80 | 8.61 | 231 | 8.74 | 417 |
|  |  |  | AS | | | I | MP | 32.8 | 97.0 | 7.6 | 124.0 | 6.6 | 207 | 156 | 75 | 10.75 | 11.66 | 12.83 | 12.47 | 224 | 12.60 | 280 |
|  |  | 2008 | ISS | | | I | MM | 25.1 | 89.0 | 12.4 | 105.0 | 7.9 | 207 | 156 | 75 | 5.38 | 6.23 | 7.48 | 7.28 | 251 | 7.34 | 541 |
|  |  |  | Chern. | | | I | MM | 26.1 | 76.0 | 12.4 | 108.0 | 7.4 | 207 | 156 | 75 | 6.55 | 7.82 | 8.30 | 8.33 | 217 | 8.37 | 409 |
|  |  | 2009 | Chern. | | | I | MM | 31.0 | 110.0 | 11.1 | 124.0 | 6.0 | 207 | 156 | 75 | 5.38 | 6.23 | 7.48 | 7.28 | 251 | 7.34 | 541 |
|  |  |  | BE | | | I | MM | 33.1 | 121.0 | 11.5 | 168.0 | 6.7 | 207 | 156 | 75 | 8.08 | 8.58 | 9.15 | 8.90 | 180 | 8.97 | 322 |
|  |  |  | CS | | | I | MM | 31.7 | 114.0 | 13.5 | 101.0 | 7.1 | 207 | 156 | 75 | 12.57 | 13.02 | 13.30 | 12.92 | 135 | 13.18 | 174 |
|  |  | 2010 | Castan. | | | I | MM | 24.4 | 150.0 | 20.1 | 140.0 | 6.3 | 207 | 156 | 75 | 5.75 | 7.93 | 8.50 | 8.64 | 228 | 8.74 | 411 |
|  |  |  | CLS | | | I | MM | 24.0 | 153.0 | 23.9 | 195.0 | 7.1 | 207 | 156 | 75 | 6.25 | 8.12 | 8.80 | 8.61 | 212 | 8.86 | 378 |
|  | 38.3°N,  108.6°E | 2008 | RE | | | I | MM | 27.4 | 123.0 | 23.6 | 145.0 | 6.8 | 207 | 156 | 75 | 8.08 | 8.58 | 9.15 | 8.90 | 180 | 8.97 | 322 |
|  |  |  | RE | | | I | MM | 20.7 | 132.0 | 15.4 | 155.0 | 7.2 | 207 | 156 | 75 | 8.42 | 9.09 | 9.90 | 9.72 | 218 | 9.76 | 352 |
|  |  |  | DBE | | | I | MM | 28.8 | 87.0 | 15.4 | 179.0 | 6.4 | 207 | 156 | 75 | 12.57 | 13.02 | 13.30 | 12.92 | 135 | 13.18 | 174 |
|  |  |  | DBE | | | I | MM | 29.2 | 146.0 | 7.9 | 170.0 | 6.1 | 207 | 156 | 75 | 10.75 | 11.66 | 12.83 | 12.47 | 224 | 12.60 | 280 |
|  |  | 2009 | DBE | | | I | MM | 31.1 | 100.0 | 16.6 | 149.0 | 6.6 | 207 | 156 | 75 | 6.38 | 6.78 | 6.87 | 6.83 | 122 | 6.79 | 314 |
|  |  |  | CS | | | I | MM | 20.0 | 180.0 | 21.9 | 135.0 | 7.4 | 207 | 156 | 75 | 5.75 | 7.93 | 8.50 | 8.64 | 228 | 8.74 | 411 |
|  |  |  | CS | | | I | MM | 28.8 | 157.0 | 17.9 | 164.0 | 7.2 | 207 | 156 | 75 | 8.88 | 9.48 | 9.90 | 9.87 | 197 | 9.84 | 318 |
|  |  |  | CS | | | I | MM | 24.8 | 173.0 | 21.8 | 177.0 | 6.2 | 207 | 156 | 75 | 10.75 | 11.66 | 12.83 | 12.47 | 224 | 12.60 | 280 |
|  | 39.3°N,  109.5°E | 2007 | CS | | | I | MM | 7.5 | 110.0 | 6.8 | 142.0 | 8.2 | 150 | 113 | 75 | 6.62 | 8.66 | 9.76 | 9.88 | 186 | 9.94 | 299 |
|  |  |  | ASS | | | I | MM | 7.3 | 137.0 | 10.0 | 99.0 | 8.4 | 150 | 113 | 75 | 6.76 | 8.95 | 10.14 | 10.29 | 187 | 10.34 | 289 |
|  |  |  | ASS | | | I | MM | 5.8 | 80.5 | 19.6 | 100.0 | 8.0 | 150 | 113 | 75 | 8.20 | 10.08 | 11.09 | 11.18 | 183 | 11.24 | 260 |
|  |  |  | MS | | | I | MM | 7.7 | 147.0 | 13.2 | 77.0 | 8.4 | 150 | 113 | 75 | 8.32 | 9.72 | 10.47 | 10.49 | 174 | 10.54 | 266 |
|  |  |  | MS | | | I | MM | 8.1 | 135.0 | 12.6 | 143.0 | 8.1 | 150 | 113 | 75 | 9.45 | 10.19 | 11.40 | 11.12 | 180 | 11.21 | 257 |
|  |  |  | Chern. | | | I | MM | 5.1 | 87.7 | 14.6 | 71.0 | 8.3 | 150 | 113 | 75 | 7.12 | 9.01 | 11.47 | 10.71 | 179 | 11.06 | 260 |
|  |  | 2008 | Chern. | | | I | MM | 8.5 | 110.0 | 15.6 | 86.0 | 8.1 | 180 | 128 | 75 | 6.84 | 8.36 | 9.68 | 8.73 | 173 | 9.35 | 298 |
|  |  |  | Chern. | | | I | MM | 9.4 | 116.0 | 7.2 | 84.0 | 8.5 | 180 | 128 | 75 | 9.30 | 9.81 | 10.14 | 9.24 | 113 | 10.04 | 202 |
|  | 39.4°N,  109.5°E | 2008 | Chern. | | | I | MM | 6.9 | 156.0 | 17.0 | 106.0 | 8.1 | 180 | 128 | 75 | 7.18 | 8.31 | 9.12 | 7.44 | 133 | 8.89 | 255 |
|  |  |  | Chern. | | | I | MM | 5.6 | 88.6 | 12.2 | 77.0 | 8.4 | 180 | 128 | 75 | 10.05 | 11.25 | 13.20 | 12.30 | 194 | 12.70 | 243 |
|  |  |  | Chern. | | | I | MM | 6.5 | 138.0 | 25.4 | 64.0 | 8.5 | 180 | 128 | 75 | 9.00 | 10.20 | 12.00 | 10.95 | 181 | 11.49 | 252 |
|  |  |  | Chern. | | | I | MM | 8.9 | 131.0 | 7.3 | 143.0 | 8.2 | 180 | 128 | 75 | 11.19 | 11.97 | 12.60 | 12.00 | 151 | 12.41 | 201 |
|  |  | 2009 | MS | | | I | MM | 19.1 | 76.0 | 17.0 | 108.0 | 8.3 | 180 | 128 | 75 | 4.73 | 9.90 | 10.25 | 8.75 | 163 | 10.72 | 248 |
|  |  |  | AS | | | I | MM | 20.7 | 87.0 | 23.4 | 142.0 | 8.2 | 180 | 128 | 75 | 7.35 | 9.19 | 10.88 | 9.34 | 166 | 10.41 | 259 |
|  |  |  | PS | | | I | MM | 18.4 | 93.0 | 25.1 | 180.0 | 8.1 | 180 | 128 | 75 | 8.31 | 9.42 | 9.47 | 8.78 | 131 | 9.55 | 235 |
|  | 39.5°N,  111.2°E | 2008 | AS | | | I | MM | 14.9 | 90.4 | 17.0 | 106.7 | 8.1 | 207 | 156 | 75 | 9.50 | 11.69 | 13.26 | 11.97 | 196 | 12.91 | 241 |
|  |  |  | AS | | | I | MM | 24.4 | 129.3 | 15.0 | 81.8 | 8.4 | 207 | 156 | 75 | 6.18 | 7.82 | 8.80 | 7.72 | 182 | 8.58 | 340 |
|  |  |  | AS | | | I | MM | 36.4 | 123.4 | 26.3 | 176.0 | 8.4 | 207 | 156 | 75 | 7.95 | 9.44 | 12.50 | 11.03 | 226 | 11.65 | 306 |
|  |  | 2009 | DBE | | | I | MM | 6.1 | 85.2 | 16.0 | 114.4 | 8.0 | 207 | 156 | 75 | 7.66 | 8.92 | 9.91 | 9.32 | 198 | 9.70 | 324 |
|  |  |  | AS | | | I | MM | 12.7 | 69.2 | 18.9 | 139.5 | 7.6 | 207 | 156 | 75 | 7.15 | 8.43 | 10.09 | 9.71 | 245 | 9.86 | 393 |
|  |  |  | BE | | | I | MM | 33.5 | 117.3 | 22.6 | 177.1 | 8.3 | 207 | 156 | 75 | 7.77 | 9.24 | 10.88 | 9.42 | 187 | 10.37 | 288 |
|  |  |  | Chern. | | | I | MP | 22.8 | 107.7 | 14.0 | 139.4 | 8.2 | 207 | 156 | 75 | 7.42 | 9.17 | 10.82 | 10.34 | 232 | 10.60 | 345 |
|  |  |  | Chern. | | | I | MM | 8.8 | 89.9 | 21.0 | 116.5 | 6.8 | 207 | 156 | 75 | 9.48 | 11.64 | 13.32 | 11.76 | 191 | 12.90 | 236 |
| Huhehaote,  Neimenggu | 40.3°N,  111.5°E | 2005 | BS | | | I | MM | 21.0 | 169.0 | 10.3 | 104.0 | 6.7 | 188 | 138 | 75 | 8.34 | 10.01 | 11.22 | 9.89 | 165 | 10.90 | 246 |
|  |  |  | BE | | | I | MM | 18.0 | 116.0 | 23.1 | 111.0 | 7.5 | 188 | 138 | 75 | 7.50 | 8.38 | 9.40 | 8.75 | 176 | 9.11 | 311 |
|  |  | 2006 | BS | | | I | MM | 25.0 | 120.0 | 15.0 | 132.0 | 7.4 | 188 | 138 | 75 | 8.85 | 9.90 | 10.44 | 9.84 | 159 | 10.33 | 252 |
|  |  |  | BS | | | I | MM | 21.0 | 138.0 | 11.7 | 144.0 | 8.1 | 188 | 138 | 75 | 7.02 | 8.49 | 8.73 | 8.40 | 166 | 8.81 | 306 |
|  |  |  | AS | | | I | MP | 20.0 | 159.0 | 21.5 | 198.0 | 8.2 | 188 | 138 | 75 | 9.41 | 10.19 | 10.98 | 10.08 | 151 | 10.71 | 233 |
|  |  | 2007 | AS | | | I | MP | 21.0 | 170.0 | 24.0 | 160.0 | 6.8 | 188 | 138 | 75 | 9.00 | 9.91 | 10.63 | 9.22 | 136 | 10.41 | 221 |
|  |  |  | ISS | | | I | MM | 20.0 | 98.0 | 21.7 | 129.0 | 6.9 | 188 | 138 | 75 | 8.55 | 9.06 | 10.17 | 9.54 | 176 | 9.80 | 289 |
|  | 40.2°N,  112.0°E | 2008 | Chern. | | | I | MM | 23.7 | 121.4 | 7.9 | 130.0 | 6.9 | 188 | 138 | 75 | 8.93 | 11.25 | 12.65 | 9.70 | 147 | 12.30 | 199 |
|  |  |  | Chern. | | | I | MM | 19.8 | 87.8 | 23.2 | 102.0 | 7.5 | 188 | 138 | 75 | 6.78 | 7.47 | 8.25 | 8.10 | 203 | 8.14 | 395 |
|  |  | 2009 | BE | | | I | MM | 19.8 | 142.3 | 21.2 | 90.0 | 7.3 | 188 | 138 | 75 | 6.19 | 6.78 | 7.66 | 7.44 | 203 | 7.49 | 429 |
|  |  |  | CS | | | I | MM | 28.0 | 134.5 | 21.0 | 119.0 | 7.4 | 188 | 138 | 75 | 6.78 | 9.87 | 10.11 | 8.94 | 162 | 10.35 | 255 |
|  |  | 2010 | Castan. | | | I | MM | 21.0 | 115.6 | 29.0 | 157.0 | 7.5 | 188 | 138 | 75 | 6.75 | 9.96 | 10.29 | 8.84 | 159 | 10.50 | 248 |
|  | 40.2°N,  111.7°E | 2008 | CLS | | | I | MM | 26.4 | 188.1 | 10.5 | 140.0 | 6.8 | 188 | 138 | 75 | 7.60 | 9.28 | 10.34 | 8.94 | 160 | 10.08 | 259 |
|  |  |  | RE | | | I | MM | 26.3 | 166.0 | 18.0 | 130.0 | 8.1 | 188 | 138 | 75 | 8.57 | 9.47 | 11.45 | 10.35 | 191 | 10.84 | 280 |
|  |  |  | RE | | | I | MM | 27.2 | 151.3 | 16.0 | 115.0 | 8.2 | 188 | 138 | 75 | 9.41 | 11.73 | 11.29 | 10.92 | 156 | 11.72 | 219 |
|  |  | 2009 | DBE | | | I | MM | 20.8 | 98.0 | 16.5 | 140.0 | 8.0 | 188 | 138 | 75 | 6.66 | 8.10 | 10.69 | 9.19 | 193 | 9.92 | 309 |
|  |  |  | DBE | | | I | MM | 20.3 | 89.0 | 19.6 | 130.0 | 7.6 | 188 | 138 | 75 | 6.83 | 8.72 | 10.10 | 8.75 | 171 | 9.76 | 283 |
|  | 39.5°N,  111.4°E | 2007 | DBE | | | I | MM | 15.0 | 115.0 | 16.0 | 110.0 | 7.5 | 165 | 69 | 45 | 6.41 | 7.29 | 7.59 | 7.13 | 133 | 7.55 | 300 |
|  |  |  | CS | | | I | MM | 16.0 | 125.0 | 15.0 | 121.0 | 7.9 | 165 | 69 | 45 | 6.20 | 7.25 | 8.00 | 6.14 | 117 | 7.80 | 266 |
|  |  |  | CS | | | I | MM | 14.5 | 103.0 | 23.0 | 98.0 | 6.8 | 165 | 69 | 45 | 7.19 | 9.26 | 9.06 | 8.81 | 145 | 9.39 | 258 |
|  |  | 2008 | CS | | | I | MM | 13.6 | 98.0 | 14.0 | 89.0 | 6.9 | 150 | 69 | 75 | 7.67 | 9.00 | 10.86 | 9.27 | 139 | 10.26 | 228 |
|  |  |  | CS | | | I | MM | 17.0 | 96.0 | 21.3 | 96.0 | 6.4 | 165 | 69 | 45 | 4.54 | 6.34 | 7.25 | 7.09 | 181 | 7.28 | 398 |
|  |  |  | ASS | | | I | MM | 18.0 | 91.0 | 25.0 | 87.0 | 6.4 | 165 | 69 | 45 | 4.85 | 8.16 | 9.26 | 9.95 | 216 | 9.89 | 345 |
|  |  |  | ASS | | | I | MM | 11.6 | 105.0 | 14.0 | 69.0 | 6.8 | 165 | 69 | 45 | 5.66 | 6.50 | 8.14 | 6.90 | 153 | 7.58 | 333 |
|  | 39.6°N,  111.4°E | 2009 | MS | | | I | MM | 15.4 | 107.0 | 21.0 | 151.0 | 6.9 | 165 | 69 | 45 | 5.75 | 6.60 | 7.02 | 6.80 | 153 | 6.97 | 362 |
|  |  |  | MS | | | I | MM | 16.3 | 115.0 | 13.0 | 121.0 | 8.1 | 165 | 69 | 45 | 4.94 | 5.95 | 7.88 | 6.31 | 151 | 7.21 | 346 |
|  |  |  | Chern. | | | I | MM | 14.5 | 136.0 | 14.0 | 140.0 | 7.2 | 165 | 69 | 45 | 5.77 | 6.48 | 7.19 | 5.90 | 119 | 6.96 | 301 |
|  |  | 2010 | Chern. | | | I | MM | 15.6 | 124.0 | 14.0 | 124.0 | 7.6 | 150 | 69 | 75 | 4.68 | 7.32 | 8.90 | 6.15 | 125 | 8.51 | 255 |
|  |  |  | Chern. | | | I | MM | 11.9 | 90.0 | 14.5 | 89.0 | 8.4 | 150 | 69 | 75 | 6.06 | 7.92 | 9.50 | 8.67 | 156 | 9.20 | 278 |
|  |  |  | Chern. | | | I | MM | 11.6 | 91.0 | 13.0 | 150.0 | 8.0 | 150 | 69 | 75 | 4.67 | 5.84 | 7.88 | 6.12 | 137 | 7.17 | 323 |
|  | 40.4°N,  111.3°E | 2005 | Chern. | | | I | MM | 11.6 | 86.0 | 5.6 | 136.0 | 8.1 | 195 | 138 | 75 | 7.65 | 9.03 | 8.59 | 8.42 | 145 | 8.90 | 272 |
|  |  |  | Chern. | | | I | MM | 9.8 | 72.0 | 9.1 | 117.0 | 8.4 | 195 | 69 | 75 | 7.23 | 11.03 | 12.94 | 10.12 | 172 | 12.56 | 221 |
|  |  | 2006 | MS | | | I | MM | 12.1 | 68.0 | 15.8 | 112.0 | 8.5 | 195 | 138 | 75 | 7.74 | 8.91 | 9.14 | 8.33 | 146 | 9.15 | 266 |
|  |  |  | AS | | | I | MM | 10.1 | 65.0 | 13.2 | 86.0 | 8.3 | 195 | 69 | 75 | 7.23 | 11.03 | 12.94 | 10.12 | 172 | 12.56 | 221 |
|  |  | 2007 | PS | | | I | MM | 23.2 | 102.0 | 15.7 | 160.0 | 8.3 | 195 | 69 | 75 | 6.97 | 10.51 | 10.76 | 9.57 | 171 | 11.06 | 250 |
|  |  |  | AS | | | I | MM | 26.1 | 128.0 | 14.1 | 147.0 | 8.3 | 195 | 138 | 75 | 9.33 | 12.89 | 12.18 | 11.37 | 161 | 12.86 | 204 |
|  |  |  | AS | | | I | MM | 21.8 | 119.0 | 12.6 | 158.0 | 8.2 | 195 | 138 | 75 | 9.43 | 10.45 | 10.33 | 9.88 | 136 | 10.46 | 220 |
|  | 40.4°N,  111.2°E | 2007 | AS | | | I | MM | 12.5 | 108.0 | 14.7 | 136.0 | 8.5 | 195 | 69 | 75 | 11.12 | 12.44 | 12.53 | 11.53 | 140 | 12.62 | 187 |
|  |  | 2008 | DBE | | | I | MM | 12.5 | 102.0 | 11.6 | 128.0 | 8.1 | 195 | 138 | 75 | 8.56 | 11.07 | 11.90 | 9.95 | 159 | 11.81 | 220 |
|  |  |  | AS | | | I | MM | 16.1 | 180.0 | 13.7 | 152.0 | 8.3 | 195 | 138 | 75 | 8.27 | 9.14 | 9.66 | 9.54 | 192 | 9.61 | 318 |
|  |  | 2009 | BE | | | I | MM | 26.5 | 138.0 | 6.4 | 176.0 | 8.3 | 195 | 138 | 75 | 8.17 | 10.69 | 12.28 | 11.03 | 189 | 11.98 | 251 |
|  |  |  | Chern. | | | I | MP | 22.8 | 142.0 | 15.9 | 192.0 | 8.0 | 195 | 138 | 75 | 10.69 | 11.50 | 11.77 | 10.12 | 119 | 11.78 | 178 |
|  |  | 2010 | Chern. | | | I | MM | 22.7 | 109.0 | 15.9 | 101.0 | 8.4 | 195 | 138 | 75 | 7.32 | 11.45 | 13.89 | 13.22 | 220 | 13.81 | 251 |
|  |  |  | BS | | | I | MM | 15.2 | 66.0 | 6.2 | 172.0 | 8.4 | 195 | 138 | 75 | 10.08 | 11.85 | 11.46 | 11.53 | 169 | 11.83 | 231 |
|  | 40.2°N,  111.1°E | 2006 | BE | | | I | MM | 13.6 | 64.0 | 13.5 | 123.0 | 7.5 | 188 | 138 | 75 | 9.30 | 12.00 | 12.60 | 10.94 | 156 | 12.63 | 203 |
|  |  |  | BS | | | I | MM | 15.1 | 93.0 | 7.8 | 148.0 | 8.0 | 188 | 138 | 75 | 5.10 | 9.01 | 9.77 | 6.81 | 151 | 9.86 | 253 |
|  |  |  | BS | | | I | MM | 11.4 | 80.0 | 11.8 | 165.0 | 8.1 | 188 | 138 | 75 | 10.00 | 10.86 | 11.63 | 11.25 | 180 | 11.46 | 252 |
|  |  | 2007 | AS | | | I | MP | 13.9 | 73.0 | 6.1 | 99.0 | 6.2 | 188 | 138 | 75 | 8.16 | 9.60 | 11.04 | 11.04 | 247 | 11.09 | 352 |
| Tongliao,  Neimenggu | 44.3°N,  121.0°E | 2007 | AS | | | I | MP | 13.9 | 134.0 | 14.2 | 115.0 | 8.1 | 188 | 138 | 75 | 6.62 | 7.43 | 8.30 | 7.94 | 187 | 8.10 | 368 |
|  |  |  | ISS | | | I | MM | 17.1 | 121.0 | 17.0 | 102.0 | 8.1 | 188 | 138 | 75 | 7.02 | 7.67 | 8.25 | 8.03 | 177 | 8.12 | 350 |
|  |  | 2008 | Chern. | | | I | MM | 11.5 | 141.0 | 14.8 | 68.0 | 8.1 | 188 | 138 | 75 | 7.80 | 9.38 | 9.51 | 9.33 | 171 | 9.66 | 286 |
|  |  |  | Chern. | | | I | MM | 13.5 | 98.0 | 21.5 | 112.0 | 8.1 | 188 | 138 | 75 | 6.87 | 8.33 | 9.27 | 8.09 | 160 | 9.03 | 290 |
|  |  |  | BE | | | I | MM | 16.0 | 96.0 | 9.8 | 85.0 | 8.1 | 188 | 138 | 75 | 7.05 | 8.73 | 10.50 | 9.45 | 189 | 10.07 | 299 |
|  |  | 2009 | CS | | | I | MM | 13.3 | 115.0 | 8.2 | 108.0 | 8.2 | 188 | 138 | 75 | 8.79 | 9.72 | 10.08 | 10.02 | 181 | 10.09 | 287 |
|  |  |  | Castan. | | | I | MM | 10.8 | 124.0 | 15.4 | 99.0 | 8.0 | 188 | 138 | 75 | 6.24 | 6.84 | 7.68 | 7.28 | 178 | 7.44 | 384 |
|  | 44.2°N,  121.0°E | 2006 | CLS | | | I | MM | 7.5 | 131.0 | 8.0 | 80.0 | 8.1 | 188 | 138 | 75 | 5.34 | 5.99 | 8.04 | 7.13 | 207 | 7.44 | 440 |
|  |  |  | RE | | | I | MM | 12.2 | 143.0 | 15.4 | 160.0 | 8.1 | 188 | 138 | 75 | 6.02 | 6.39 | 7.20 | 6.78 | 168 | 6.92 | 393 |
|  |  |  | RE | | | I | MM | 7.4 | 105.0 | 9.4 | 150.0 | 8.3 | 188 | 138 | 75 | 7.65 | 8.97 | 10.04 | 7.17 | 130 | 9.76 | 229 |
|  |  | 2007 | DBE | | | I | MM | 6.5 | 121.0 | 12.9 | 80.0 | 8.2 | 188 | 138 | 75 | 7.28 | 7.65 | 9.03 | 8.52 | 208 | 8.65 | 380 |
|  |  |  | DBE | | | I | MM | 9.0 | 116.0 | 23.4 | 120.0 | 8.2 | 188 | 138 | 75 | 6.62 | 7.08 | 8.09 | 7.82 | 209 | 7.87 | 420 |
|  |  | 2008 | DBE | | | I | MM | 5.0 | 135.0 | 20.0 | 155.0 | 8.3 | 188 | 138 | 75 | 7.55 | 8.51 | 9.65 | 9.15 | 193 | 9.38 | 327 |
|  |  |  | CS | | | I | MM | 8.8 | 98.0 | 29.1 | 110.0 | 8.2 | 188 | 138 | 75 | 7.88 | 9.81 | 10.70 | 9.56 | 165 | 10.55 | 254 |
|  |  |  | CS | | | I | MM | 12.8 | 78.0 | 14.4 | 145.0 | 8.3 | 188 | 138 | 75 | 8.60 | 9.26 | 10.98 | 10.13 | 195 | 10.45 | 296 |
|  | 44.1°N,  121.0°E | 2008 | CS | | | I | MM | 7.4 | 92.0 | 20.9 | 68.0 | 8.2 | 188 | 138 | 75 | 6.33 | 8.03 | 10.35 | 9.33 | 206 | 9.82 | 332 |
|  |  |  | CS | | | I | MM | 11.3 | 89.0 | 15.1 | 120.0 | 8.2 | 188 | 138 | 75 | 6.92 | 8.13 | 10.23 | 9.11 | 195 | 9.63 | 322 |
|  |  | 2009 | ASS | | | I | MM | 11.9 | 104.0 | 13.1 | 112.0 | 8.5 | 188 | 138 | 75 | 7.55 | 10.59 | 11.58 | 9.60 | 160 | 11.49 | 228 |
|  |  |  | ASS | | | I | MM | 7.8 | 112.0 | 5.6 | 90.0 | 8.5 | 188 | 138 | 75 | 7.55 | 9.60 | 11.13 | 10.52 | 200 | 10.93 | 290 |
|  |  | 2010 | MS | | | I | MM | 12.2 | 140.0 | 6.4 | 80.0 | 8.2 | 188 | 138 | 75 | 8.72 | 10.25 | 10.95 | 9.90 | 158 | 10.81 | 239 |
|  |  |  | MS | | | I | MM | 7.8 | 116.0 | 8.7 | 95.0 | 8.4 | 188 | 138 | 75 | 6.42 | 7.71 | 10.20 | 8.85 | 196 | 9.47 | 329 |
|  | 42.5°N,  120.4°E | 2006 | Chern. | | | I | MM | 5.2 | 89.0 | 12.1 | 78.0 | 8.4 | 188 | 120 | 75 | 6.60 | 7.70 | 9.56 | 8.45 | 187 | 9.00 | 332 |
|  |  |  | Chern. | | | I | MM | 12.2 | 97.0 | 6.4 | 80.0 | 8.4 | 188 | 120 | 75 | 7.58 | 10.64 | 12.14 | 11.76 | 207 | 12.16 | 269 |
|  |  | 2007 | Chern. | | | I | MM | 11.8 | 67.0 | 24.2 | 142.0 | 8.4 | 188 | 120 | 75 | 9.63 | 10.97 | 11.63 | 11.48 | 194 | 11.62 | 265 |
|  |  |  | Chern. | | | I | MM | 10.7 | 60.0 | 20.1 | 167.0 | 8.5 | 188 | 120 | 75 | 4.80 | 7.28 | 10.86 | 9.93 | 235 | 10.33 | 359 |
|  |  | 2008 | Chern. | | | I | MM | 6.0 | 121.0 | 17.6 | 168.0 | 7.6 | 188 | 120 | 75 | 6.10 | 8.38 | 7.23 | 6.40 | 127 | 7.99 | 275 |
|  |  |  | Chern. | | | I | MM | 5.7 | 114.0 | 8.1 | 130.0 | 8.4 | 188 | 120 | 75 | 8.93 | 10.88 | 11.70 | 11.63 | 206 | 11.79 | 276 |
|  | 43.3°N,  121.2°E | 2006 | MS | | | I | MM | 21.8 | 175.9 | 18.9 | 92.0 | 7.7 | 188 | 138 | 75 | 6.73 | 9.62 | 11.51 | 9.80 | 178 | 11.11 | 257 |
|  |  |  | AS | | | I | MM | 27.6 | 101.9 | 20.7 | 83.0 | 7.4 | 188 | 138 | 75 | 10.50 | 11.57 | 13.41 | 13.10 | 245 | 13.22 | 293 |
|  |  |  | PS | | | I | MM | 23.7 | 105.6 | 28.1 | 117.0 | 7.3 | 188 | 138 | 75 | 7.19 | 9.06 | 12.45 | 11.04 | 215 | 11.63 | 292 |
|  |  |  | AS | | | I | MM | 25.9 | 98.3 | 16.3 | 82.0 | 7.6 | 188 | 138 | 75 | 8.90 | 12.44 | 12.77 | 12.75 | 193 | 13.22 | 232 |
|  |  |  | AS | | | I | MM | 30.0 | 154.0 | 22.7 | 112.0 | 6.9 | 188 | 138 | 75 | 8.18 | 10.17 | 10.98 | 10.46 | 183 | 10.95 | 267 |
|  | 43.3°N,  121.1°E | 2006 | AS | | | I | MM | 15.0 | 138.6 | 19.5 | 100.0 | 6.8 | 188 | 138 | 75 | 8.61 | 9.44 | 9.74 | 8.22 | 118 | 9.72 | 214 |
|  |  |  | DBE | | | I | MM | 21.3 | 115.5 | 19.1 | 108.0 | 6.7 | 242 | 90 | 75 | 9.20 | 10.16 | 10.56 | 10.52 | 227 | 10.55 | 339 |
|  |  |  | AS | | | I | MM | 26.2 | 138.6 | 6.0 | 150.0 | 8.2 | 242 | 90 | 75 | 10.79 | 12.77 | 12.90 | 12.90 | 230 | 13.14 | 276 |
|  |  |  | BE | | | I | MM | 24.8 | 109.0 | 21.4 | 118.0 | 8.1 | 188 | 138 | 75 | 12.45 | 13.21 | 13.55 | 13.44 | 170 | 13.52 | 203 |
|  |  | 2007 | Chern. | | | I | MP | 33.4 | 157.5 | 21.3 | 151.0 | 7.6 | 222 | 138 | 75 | 9.12 | 12.17 | 12.63 | 12.22 | 214 | 12.88 | 262 |
|  |  |  | Chern. | | | I | MM | 24.7 | 145.0 | 23.5 | 73.0 | 7.7 | 222 | 138 | 75 | 11.43 | 12.49 | 13.23 | 12.33 | 180 | 13.02 | 222 |
|  |  |  | BS | | | I | MM | 14.0 | 83.0 | 17.4 | 116.0 | 7.4 | 188 | 138 | 75 | 8.46 | 11.33 | 11.51 | 12.45 | 240 | 12.28 | 309 |
|  |  |  | BE | | | I | MM | 14.0 | 121.0 | 16.0 | 192.0 | 7.3 | 188 | 138 | 75 | 8.45 | 13.25 | 12.20 | 13.47 | 205 | 13.49 | 240 |
|  |  | 2008 | BS | | | I | MM | 19.6 | 185.0 | 26.8 | 159.0 | 6.8 | 188 | 138 | 75 | 7.28 | 8.94 | 9.89 | 9.63 | 200 | 9.85 | 322 |
|  |  |  | BS | | | I | MM | 21.3 | 82.0 | 20.6 | 63.0 | 6.9 | 188 | 138 | 75 | 7.76 | 8.40 | 8.58 | 8.19 | 133 | 8.54 | 266 |
|  |  |  | AS | | | I | MP | 12.4 | 107.0 | 18.9 | 136.0 | 7.2 | 188 | 138 | 75 | 5.78 | 10.04 | 10.44 | 9.87 | 182 | 10.87 | 268 |
|  |  |  | AS | | | I | MP | 28.6 | 103.0 | 11.2 | 103.0 | 8.1 | 188 | 138 | 75 | 9.58 | 10.02 | 11.61 | 11.03 | 214 | 11.19 | 302 |
|  | 43.4°N,  122.2°E | 2008 | ISS | | | I | MM | 25.6 | 188.0 | 32.5 | 181.0 | 7.8 | 188 | 138 | 75 | 7.22 | 9.98 | 11.18 | 10.53 | 191 | 11.14 | 273 |
|  |  |  | Chern. | | | I | MM | 31.0 | 107.0 | 18.9 | 136.0 | 7.9 | 188 | 138 | 75 | 10.22 | 11.31 | 13.70 | 12.48 | 200 | 13.00 | 244 |
|  |  |  | Chern. | | | I | MM | 21.3 | 103.0 | 21.4 | 74.0 | 8.2 | 188 | 138 | 75 | 8.19 | 13.41 | 13.35 | 13.33 | 189 | 14.15 | 213 |
|  |  |  | BE | | | I | MM | 18.5 | 128.0 | 27.2 | 82.0 | 7.6 | 188 | 138 | 75 | 9.79 | 12.92 | 12.97 | 11.86 | 160 | 13.29 | 197 |
|  |  |  | CS | | | I | MM | 19.6 | 128.0 | 19.3 | 114.0 | 8.2 | 188 | 138 | 75 | 10.73 | 12.38 | 13.07 | 13.36 | 233 | 13.32 | 276 |
|  | 43.3°N,  122.1°E | 2008 | Castan. | | | I | MM | 28.9 | 114.0 | 32.5 | 117.0 | 7.6 | 188 | 138 | 75 | 12.40 | 12.95 | 13.61 | 13.27 | 170 | 13.41 | 205 |
|  |  | 2009 | CLS | | | I | MM | 35.7 | 156.0 | 32.1 | 79.0 | 7.7 | 167 | 138 | 75 | 10.40 | 11.61 | 11.61 | 11.04 | 127 | 11.73 | 187 |
|  |  |  | RE | | | I | MM | 29.5 | 163.0 | 16.6 | 198.0 | 6.9 | 168 | 138 | 75 | 10.67 | 11.29 | 11.11 | 11.03 | 106 | 11.21 | 173 |
|  |  |  | RE | | | I | MM | 20.8 | 98.0 | 9.4 | 191.0 | 6.8 | 167 | 138 | 75 | 10.98 | 11.90 | 12.75 | 12.62 | 189 | 12.68 | 238 |
|  |  |  | DBE | | | I | MM | 13.6 | 114.0 | 23.6 | 171.0 | 6.9 | 167 | 138 | 75 | 11.07 | 12.68 | 13.17 | 13.26 | 184 | 13.33 | 221 |
|  | 42.6°N,  122.3°E | 2006 | DBE | | | I | MM | 10.9 | 121.0 | 13.6 | 90.0 | 7.3 | 150 | 98 | 75 | 6.24 | 7.14 | 7.88 | 7.98 | 195 | 7.98 | 388 |
|  |  |  | DBE | | | I | MM | 15.9 | 114.0 | 26.0 | 113.0 | 7.9 | 150 | 98 | 75 | 8.27 | 9.50 | 9.92 | 9.57 | 139 | 9.91 | 236 |
|  |  |  | CS | | | I | MM | 11.9 | 78.0 | 16.9 | 124.0 | 8.2 | 150 | 98 | 75 | 8.09 | 9.24 | 10.53 | 9.36 | 136 | 10.14 | 227 |
| Baiyin,  Gansu | 35.4°N,  105.0°E | 2007 | CS | | | I | MM | 22.8 | 86.0 | 18.0 | 152.0 | 7.2 | 240 | 135 | 105 | 7.91 | 9.96 | 11.46 | 10.28 | 251 | 11.12 | 357 |
|  |  | 2008 | CS | | | I | MM | 21.6 | 137.1 | 25.3 | 116.0 | 6.4 | 240 | 135 | 105 | 4.56 | 7.70 | 8.33 | 7.02 | 234 | 8.42 | 438 |
|  | 37.1°N,  104.0°E | 2007 | CS | | | I | MM | 23.2 | 103.9 | 19.8 | 200.0 | 6.1 | 240 | 180 | 120 | 12.33 | 13.91 | 13.58 | 13.92 | 239 | 13.92 | 271 |
|  |  | 2008 | ASS | | | I | MM | 12.2 | 78.0 | 25.6 | 128.0 | 6.6 | 240 | 180 | 120 | 10.36 | 11.45 | 12.36 | 12.00 | 259 | 12.18 | 337 |
|  | 35.5°N,  104.1°E | 2007 | ASS | | | I | MM | 11.6 | 78.0 | 18.0 | 131.0 | 7.4 | 240 | 120 | 120 | 5.95 | 6.54 | 7.45 | 7.25 | 251 | 7.26 | 547 |
| **NW3 subregion** |  |  |  | | |  |  |  |  |  |  |  |  |  |  |  |  |  |  |  |  |  |
| Akesu,  Xinjiang | 41.4°N,  82.5°E | 2006 | FAS | | | I | MM | 14.6 | 90.6 | 19.6 | 145.0 | 8.3 | 205 | 150 | 30 | 6.45 | 7.64 | 8.12 | 8.06 | 206 | 8.15 | 395 |
|  |  | 2007 | BP | | | I | MM | 8.5 | 96.5 | 15.4 | 128.0 | 8.4 | 200 | 132 | 30 | 6.44 | 7.66 | 8.13 | 8.04 | 199 | 8.16 | 382 |
| Aletai,  Xinjiang | 46.6°N,  89.3°E | 2008 | BP | | | I | MM | 22.3 | 100.0 | 15.0 | 218.0 | 7.2 | 225 | 96 | 30 | 5.99 | 7.79 | 8.70 | 8.40 | 222 | 8.67 | 399 |
|  | 48.0°N,  86.3°E | 2007 | BP | | | I | MM | 12.9 | 120.0 | 11.9 | 86.0 | 7.8 | 225 | 138 | 30 | 8.12 | 10.50 | 11.66 | 11.13 | 220 | 11.60 | 296 |
|  | 47.1°N,  87.5°E | 2009 | FAS | | | I | MM | 12.4 | 110.0 | 16.9 | 146.0 | 7.9 | 225 | 96 | 30 | 4.82 | 7.31 | 10.27 | 9.20 | 245 | 9.73 | 394 |
| Hejing,  Xinjiang | 42.3°N,  86.2°E | 2009 | FAS | | | I | MM | 19.1 | 68.0 | 11.5 | 176.0 | 8.3 | 225 | 75 | 30 | 8.96 | 10.43 | 11.78 | 10.88 | 206 | 11.44 | 281 |
|  |  |  | FAS | | | I | MM | 21.0 | 71.0 | 22.5 | 173.0 | 8.3 | 225 | 75 | 30 | 9.08 | 10.41 | 11.48 | 10.79 | 204 | 11.23 | 283 |
| **SW1 sub-region** |  |  |  | | |  |  |  |  |  |  |  |  |  |  |  |  |  |  |  |  |  |
| Deyang,  Sichuan | 31.0°N,  104.4°E | 2006 | YE | R | | | MM | 32.6 | 123.0 | 12.5 | 168.0 | 6.7 | 240 | 90 | 90 | 5.54 | 6.39 | 7.20 | 6.29 | 188 | 6.93 | 429 |
|  |  | 2007 | PS | I | | | MSP | 25.2 | 154.0 | 7.4 | 148.0 | 6.2 | 240 | 90 | 90 | 6.17 | 7.02 | 7.67 | 6.86 | 184 | 7.46 | 391 |
|  |  | 2008 | PS | R | | | MM | 14.0 | 125.0 | 6.3 | 125.0 | 5.9 | 240 | 90 | 90 | 7.89 | 9.24 | 8.94 | 9.05 | 194 | 9.20 | 333 |
|  | 31.0°N,  104.5°E | 2008 | PS | R | | | MM | 15.6 | 96.0 | 19.2 | 135.0 | 6.8 | 195 | 60 | 90 | 8.54 | 8.88 | 9.15 | 8.86 | 123 | 9.02 | 235 |
|  |  | 2009 | PS | I | | | MSP | 13.8 | 75.8 | 16.8 | 110.0 | 7.0 | 195 | 60 | 75 | 7.19 | 7.58 | 7.95 | 7.74 | 147 | 7.80 | 310 |
|  |  | 2010 | FAS | I | | | MW | 21.9 | 86.8 | 22.5 | 140.0 | 6.1 | 195 | 60 | 75 | 6.42 | 6.96 | 7.37 | 6.98 | 148 | 7.22 | 337 |
| Bazhong,  Sichuan | 31.3°N,  107.1°E | 2008 | PS | I | | | MM | 26.0 | 110.6 | 7.9 | 66.0 | 8.3 | 218 | 53 | 30 | 6.53 | 7.61 | 8.08 | 8.06 | 219 | 8.10 | 424 |
| Dazhou,  Sichuan | 31.1°N,  107.3°E | 2007 | PS | R | | | MM | 21.3 | 108.0 | 11.5 | 95.0 | 7.2 | 225 | 90 | 113 | 6.39 | 7.15 | 7.61 | 7.43 | 199 | 7.52 | 416 |
|  | 30.4°N,  107.1°E | 2007 | PS | R | | | MM | 13.7 | 134.2 | 14.1 | 104.0 | 8.1 | 240 | 105 | 75 | 4.30 | 6.35 | 7.40 | 5.83 | 201 | 7.18 | 440 |
|  |  | 2009 | PS | R | | | MM | 15.5 | 103.2 | 21.0 | 76.0 | 7.8 | 240 | 105 | 75 | 4.14 | 7.71 | 8.15 | 6.70 | 207 | 8.36 | 389 |
| Guangyuan, Sichuan | 32.2°N,  105.3°E | 2005 | FAS | R | | | MM | 16.5 | 73.0 | 15.7 | 64.0 | 8.2 | 152 | 56 | 84 | 5.37 | 6.39 | 7.16 | 6.63 | 143 | 6.99 | 339 |
|  |  | 2008 | FAS | R | | | MM | 13.4 | 89.0 | 14.1 | 91.0 | 8.1 | 155 | 46 | 74 | 6.20 | 9.59 | 8.67 | 7.71 | 123 | 9.42 | 225 |
|  |  | 2009 | CS | I | | | MOR | 17.3 | 145.6 | 16.0 | 131.0 | 8.0 | 210 | 40 | 45 | 3.20 | 5.75 | 5.72 | 4.25 | 163 | 5.99 | 439 |
|  | 32.4°N,  105.5°E | 2007 | YE | I | | | MM | 13.2 | 116.5 | 6.4 | 74.7 | 5.9 | 225 | 75 | 90 | 7.00 | 7.29 | 7.55 | 7.37 | 124 | 7.38 | 289 |
|  |  | 2008 | YE | I | | | MM | 14.5 | 156.3 | 20.8 | 146.0 | 6.0 | 225 | 90 | 90 | 6.06 | 6.73 | 7.52 | 7.31 | 226 | 7.35 | 482 |
|  |  | 2009 | YE | R | | | MOR | 16.0 | 145.7 | 19.5 | 103.0 | 5.6 | 225 | 75 | 75 | 4.74 | 6.35 | 7.82 | 6.71 | 210 | 7.44 | 443 |
|  |  |  | YE | R | | | MM | 19.5 | 142.0 | 14.6 | 119.0 | 5.6 | 225 | 90 | 75 | 6.27 | 7.76 | 9.54 | 8.21 | 211 | 9.01 | 367 |
|  |  |  | YE | R | | | MM | 16.9 | 135.2 | 10.5 | 86.0 | 5.7 | 225 | 75 | 90 | 6.19 | 7.50 | 8.98 | 8.00 | 214 | 8.57 | 391 |
|  | 32.4°N,  105.6°E | 2009 | YE | R | | | MM | 19.5 | 124.3 | 22.8 | 130.0 | 6.9 | 240 | 60 | 90 | 6.83 | 8.54 | 9.26 | 9.09 | 245 | 9.28 | 415 |
|  |  |  | YE | R | | | MM | 14.6 | 152.0 | 19.4 | 120.0 | 6.8 | 240 | 60 | 90 | 7.05 | 8.82 | 9.54 | 9.41 | 247 | 9.59 | 405 |
|  |  |  | PS | R | | | MM | 16.9 | 76.8 | 18.4 | 130.0 | 6.5 | 240 | 60 | 90 | 6.03 | 7.79 | 8.25 | 8.24 | 243 | 8.38 | 455 |
|  |  | 2009 | PS | R | | | MSP | 17.9 | 120.1 | 30.9 | 170.0 | 6.2 | 240 | 60 | 90 | 6.35 | 8.10 | 8.57 | 8.57 | 244 | 8.70 | 440 |
|  |  |  | FAS | R | | | MM | 18.7 | 74.3 | 16.7 | 140.0 | 6.1 | 240 | 60 | 90 | 6.02 | 7.92 | 8.39 | 8.24 | 234 | 8.50 | 432 |
|  |  |  | FAS | I | | | MW | 26.4 | 88.0 | 19.8 | 83.0 | 6.3 | 240 | 60 | 90 | 5.07 | 6.71 | 7.17 | 7.01 | 231 | 7.23 | 501 |
|  |  | 2010 | PS | I | | | MM | 13.4 | 69.0 | 8.1 | 100.0 | 6.3 | 240 | 60 | 90 | 5.63 | 6.62 | 6.89 | 6.80 | 209 | 6.89 | 476 |
|  |  |  | PS | I | | | MOR | 20.5 | 98.7 | 17.4 | 82.0 | 5.8 | 240 | 60 | 90 | 5.61 | 6.60 | 6.89 | 6.78 | 209 | 6.89 | 476 |
|  | 32.5°N,  105.6°E | 2006 | RE | I | | | MW | 15.8 | 78.0 | 21.9 | 158.0 | 7.0 | 240 | 60 | 90 | 5.03 | 6.12 | 6.35 | 6.32 | 215 | 6.40 | 527 |
|  |  | 2007 | PS | R | | | MSP | 16.3 | 126.0 | 6.7 | 110.0 | 6.0 | 240 | 60 | 90 | 4.89 | 6.09 | 6.36 | 6.33 | 221 | 6.42 | 539 |
|  |  |  | YB | R | | | MM | 19.4 | 110.0 | 28.6 | 120.0 | 6.1 | 240 | 60 | 90 | 5.00 | 6.08 | 6.30 | 6.30 | 217 | 6.36 | 535 |
|  |  | 2008 | RE | R | | | MM | 16.3 | 85.6 | 16.1 | 111.0 | 6.9 | 240 | 60 | 90 | 4.85 | 5.67 | 6.23 | 6.20 | 240 | 6.18 | 609 |
|  |  |  | YE | R | | | MM | 17.1 | 96.3 | 22.1 | 88.0 | 6.4 | 240 | 60 | 90 | 4.35 | 5.25 | 5.37 | 5.36 | 198 | 5.41 | 576 |
|  |  | 2009 | YB | R | | | MOR | 13.9 | 84.0 | 11.3 | 110.0 | 6.6 | 240 | 60 | 90 | 4.40 | 5.33 | 5.61 | 5.60 | 216 | 5.62 | 602 |
|  |  |  | YB | I | | | MM | 16.1 | 87.0 | 7.5 | 100.0 | 6.4 | 240 | 60 | 90 | 4.47 | 5.52 | 5.67 | 5.61 | 204 | 5.72 | 560 |
| Guang'an,  Sichuan | 30.2°N,  106.6°E | 2006 | LS | R | | | MM | 17.8 | 105.6 | 15.4 | 89.0 | 7.8 | 210 | 90 | 75 | 4.08 | 6.23 | 7.91 | 6.27 | 191 | 7.47 | 404 |
|  |  | 2009 | RE | R | | | MW | 19.7 | 108.0 | 18.4 | 89.0 | 7.6 | 210 | 90 | 75 | 4.40 | 6.41 | 9.55 | 8.03 | 228 | 8.74 | 409 |
| Mianyang,  Sichuan | 31.5°N,  104.4°E | 2006 | YE | I | | | MM | 15.6 | 89.7 | 12.7 | 135.0 | 8.1 | 180 | 90 | 120 | 3.89 | 4.98 | 5.24 | 4.98 | 158 | 5.26 | 487 |
|  |  | 2009 | RE | R | | | MM | 16.5 | 90.0 | 6.3 | 95.0 | 7.9 | 180 | 105 | 105 | 5.37 | 7.00 | 6.84 | 7.22 | 186 | 7.20 | 409 |
|  | 31.4°N,  105.1°E | 2006 | RE | R | | | MSP | 18.7 | 79.6 | 10.9 | 136.5 | 6.0 | 180 | 60 | 75 | 7.61 | 8.82 | 9.57 | 8.78 | 157 | 9.40 | 271 |
| Nanchong,  Sichuan | 31.2°N,  106.0°E | 2006 | YB | R | | | MW | 18.2 | 154.3 | 7.9 | 79.8 | 7.5 | 188 | 75 | 90 | 4.34 | 5.63 | 5.58 | 5.52 | 162 | 5.75 | 455 |
|  |  | 2008 | LS | R | | | MM | 28.5 | 135.6 | 9.9 | 76.0 | 7.9 | 225 | 90 | 81 | 6.57 | 6.86 | 6.87 | 6.30 | 87 | 6.86 | 247 |
|  | 31.0°N,  106.3°E | 2008 | PS | R | | | MM | 21.8 | 110.4 | 34.8 | 65.0 | 5.8 | 240 | 105 | 105 | 4.91 | 7.31 | 5.75 | 4.35 | 144 | 6.78 | 351 |
|  |  | 2009 | YE | I | | | MSP | 26.3 | 131.2 | 41.3 | 107.5 | 5.8 | 240 | 90 | 60 | 5.30 | 6.29 | 7.31 | 6.73 | 224 | 7.04 | 498 |
|  | 31.0°N,  106.2°E | 2008 | FAS | R | | | MM | 26.3 | 150.0 | 13.4 | 143.0 | 7.7 | 180 | 60 | 45 | 3.35 | 6.20 | 5.87 | 5.42 | 157 | 6.33 | 403 |
|  |  |  | PS | R | | | MOR | 22.6 | 141.0 | 14.4 | 141.0 | 7.9 | 192 | 90 | 45 | 4.55 | 7.52 | 7.99 | 5.96 | 154 | 8.11 | 310 |
| Neijiang,  Sichuan | 29.3°N,  104.4°E | 2006 | CS | R | | | MM | 25.7 | 143.5 | 7.1 | 98.0 | 5.8 | 180 | 81 | 72 | 3.52 | 4.27 | 5.40 | 4.46 | 157 | 5.01 | 509 |
|  |  | 2007 | PS | R | | | MM | 16.8 | 98.0 | 13.5 | 105.6 | 6.3 | 210 | 90 | 90 | 6.34 | 7.13 | 7.54 | 7.71 | 239 | 7.63 | 491 |
|  |  |  | PS | R | | | MSP | 22.7 | 116.5 | 16.7 | 88.0 | 5.6 | 206 | 72 | 72 | 3.45 | 4.52 | 5.58 | 4.79 | 187 | 5.28 | 561 |
|  | 31.2°N,  106.2°E | 2006 | PS | R | | | MW | 22.6 | 120.3 | 26.9 | 181.0 | 5.9 | 204 | 113 | 90 | 4.15 | 5.08 | 5.68 | 5.28 | 182 | 5.55 | 521 |
|  |  |  | YE | R | | | MM | 31.9 | 125.7 | 17.2 | 134.0 | 5.6 | 204 | 113 | 90 | 4.18 | 5.04 | 6.08 | 5.84 | 226 | 5.92 | 598 |
| Suining,  Sichuan | 30.5°N,  105.4°E | 2008 | YE | R | | | MM | 22.0 | 80.5 | 15.2 | 143.0 | 8.1 | 240 | 60 | 60 | 4.89 | 6.51 | 8.89 | 6.78 | 216 | 8.09 | 418 |
| Tongren,  Sichuan | 27.3°N,  108.1°E | 2008 | YE | R | | | MM | 28.0 | 146.8 | 11.5 | 160.0 | 6.4 | 225 | 150 | 240 | 5.42 | 6.71 | 8.08 | 7.69 | 247 | 7.87 | 493 |
|  |  |  | YE | R | | | MM | 25.4 | 145.3 | 22.9 | 156.0 | 5.8 | 225 | 150 | 240 | 6.30 | 7.03 | 7.37 | 6.45 | 148 | 7.27 | 335 |
|  |  |  | YE | R | | | MM | 35.3 | 155.4 | 32.4 | 130.0 | 5.8 | 225 | 150 | 240 | 5.62 | 7.43 | 8.21 | 7.49 | 205 | 8.12 | 397 |
| Yibin,  Sichuan | 28.3°N,  104.6°E | 2007 | PS | R | | | MM | 19.5 | 151.0 | 15.6 | 102.0 | 7.0 | 210 | 75 | 90 | 3.19 | 5.35 | 6.23 | 4.53 | 173 | 6.08 | 455 |
|  | 28.4°N,  105.0°E | 2008 | YE | R | | | MM | 21.6 | 69.1 | 12.5 | 98.0 | 7.9 | 180 | 81 | 119 | 5.88 | 7.19 | 8.30 | 6.68 | 145 | 7.95 | 301 |
|  | 28.5°N,  104.4°E | 2005 | PS | R | | | MM | 20.2 | 142.3 | 15.2 | 90.0 | 5.7 | 180 | 75 | 105 | 7.00 | 7.93 | 8.44 | 7.47 | 136 | 8.31 | 275 |
|  |  | 2006 | PS | I | | | MW | 16.0 | 92.0 | 19.1 | 94.0 | 6.7 | 225 | 60 | 150 | 7.48 | 7.66 | 7.75 | 7.00 | 81 | 7.73 | 210 |
|  |  |  | PS | R | | | MSP | 15.2 | 74.0 | 26.9 | 89.0 | 5.9 | 225 | 60 | 150 | 7.42 | 8.56 | 9.04 | 9.02 | 226 | 9.07 | 390 |
|  |  |  | PS | R | | | MM | 16.2 | 75.0 | 25.4 | 78.0 | 6.1 | 225 | 60 | 150 | 5.81 | 7.62 | 7.63 | 7.86 | 223 | 7.93 | 441 |
|  |  |  | PS | R | | | MOR | 15.4 | 64.0 | 24.8 | 79.0 | 5.9 | 225 | 60 | 150 | 7.44 | 8.28 | 8.58 | 7.57 | 149 | 8.52 | 287 |
|  | 28.5°N,  104.5°E | 2006 | YE | R | | | MSP | 15.0 | 123.2 | 20.8 | 101.0 | 5.9 | 225 | 60 | 150 | 7.90 | 9.00 | 9.15 | 9.39 | 229 | 9.32 | 385 |
|  |  | 2007 | YE | I | | | MSP | 17.5 | 143.2 | 13.4 | 75.0 | 6.0 | 225 | 60 | 150 | 6.12 | 7.09 | 7.50 | 6.94 | 177 | 7.41 | 381 |
|  |  |  | FAS | R | | | MM | 16.8 | 98.6 | 21.2 | 120.0 | 5.9 | 225 | 60 | 150 | 6.04 | 7.02 | 8.22 | 7.80 | 236 | 7.97 | 464 |
|  |  |  | PS | R | | | MOR | 16.1 | 89.5 | 20.4 | 111.0 | 6.5 | 225 | 60 | 150 | 4.75 | 6.10 | 6.15 | 6.32 | 215 | 6.35 | 531 |
|  |  |  | CS | R | | | MM | 19.6 | 96.3 | 8.7 | 88.0 | 7.3 | 225 | 60 | 150 | 7.28 | 8.40 | 9.36 | 7.39 | 161 | 9.06 | 287 |
|  |  | 2008 | PS | R | | | MM | 16.3 | 79.6 | 16.7 | 84.0 | 7.0 | 225 | 60 | 150 | 7.44 | 8.01 | 8.86 | 8.19 | 183 | 8.55 | 340 |
|  | 28.4°N,  104.5°E | 2008 | PS | R | | | MSP | 19.1 | 87.5 | 18.4 | 117.0 | 6.5 | 225 | 60 | 150 | 8.15 | 8.63 | 9.12 | 8.95 | 188 | 8.97 | 332 |
|  |  |  | PS | R | | | MW | 15.8 | 102.6 | 16.0 | 90.0 | 6.3 | 225 | 60 | 150 | 7.66 | 8.28 | 8.30 | 8.36 | 161 | 8.31 | 313 |
|  |  |  | YE | R | | | MM | 17.8 | 153.2 | 22.8 | 68.0 | 6.3 | 225 | 60 | 150 | 6.50 | 7.38 | 8.20 | 7.33 | 181 | 7.93 | 363 |
|  |  |  | YE | R | | | MM | 16.7 | 132.4 | 16.4 | 89.0 | 6.8 | 225 | 60 | 150 | 7.36 | 7.77 | 7.92 | 7.50 | 122 | 7.84 | 269 |
|  |  | 2009 | YE | R | | | MM | 21.2 | 142.3 | 19.7 | 144.0 | 7.1 | 225 | 60 | 150 | 6.65 | 8.76 | 9.36 | 9.48 | 246 | 9.57 | 404 |
|  |  |  | YE | R | | | MM | 21.1 | 125.3 | 12.7 | 65.0 | 7.1 | 225 | 60 | 150 | 6.70 | 8.61 | 8.83 | 8.45 | 200 | 8.97 | 351 |
|  |  |  | YE | R | | | MM | 16.5 | 136.1 | 10.1 | 80.0 | 7.0 | 225 | 60 | 150 | 6.56 | 8.09 | 8.51 | 8.65 | 239 | 8.67 | 432 |
| Zigong,  Sichuan | 29.1°N,  104.6°E | 2009 | PS | R | | | MM | 22.8 | 86.0 | 13.9 | 152.0 | 7.7 | 210 | 75 | 75 | 3.81 | 6.94 | 7.22 | 7.52 | 226 | 7.70 | 460 |
|  | 29.3°N,  104.3°E | 2009 | YE | R | | | MM | 20.2 | 134.5 | 10.6 | 95.0 | 8.3 | 225 | 90 | 90 | 4.19 | 6.45 | 6.43 | 5.92 | 192 | 6.69 | 453 |
| Ziyang,  Sichuan | 30.2°N,  104.3°E | 2006 | PS | R | | | MM | 15.9 | 98.0 | 18.7 | 137.0 | 8.0 | 240 | 90 | 45 | 5.05 | 5.70 | 6.54 | 5.53 | 178 | 6.22 | 456 |
|  |  | 2008 | PS | I | | | MW | 19.4 | 131.0 | 22.7 | 65.0 | 8.1 | 240 | 90 | 45 | 7.64 | 8.61 | 9.03 | 8.38 | 182 | 8.93 | 324 |
|  |  |  | PS | R | | | MSP | 15.6 | 110.0 | 21.0 | 90.0 | 8.1 | 240 | 90 | 45 | 5.98 | 6.93 | 7.41 | 6.83 | 189 | 7.29 | 410 |
| **SW2 subregion** |  |  |  |  | | |  |  |  |  |  |  |  |  |  |  |  |  |  |  |  |  |
| Anshun,  Guizhou | 26.2°N,  106.2°E | 2008 | YB | I | | | MW | 27.4 | 108.9 | 18.9 | 66.0 | 6.9 | 210 | 135 | 210 | 5.36 | 8.31 | 8.53 | 8.60 | 214 | 8.93 | 380 |
|  |  |  | YE | R | | | MW | 25.0 | 112.0 | 13.0 | 151.0 | 7.0 | 180 | 120 | 180 | 5.85 | 7.19 | 10.46 | 8.80 | 196 | 9.49 | 330 |
|  | 26.2°N,  105.4°E | 2008 | YE | R | | | MW | 21.3 | 78.5 | 10.1 | 154.0 | 6.9 | 240 | 150 | 240 | 7.71 | 8.43 | 8.90 | 8.54 | 191 | 8.76 | 349 |
|  | 26.2°N,  106.2°E | 2008 | YE | R | | | MP | 29.3 | 88.0 | 13.4 | 130.0 | 7.1 | 210 | 135 | 210 | 5.42 | 6.72 | 6.62 | 6.94 | 206 | 6.89 | 475 |
|  |  |  | Sier. | I | | | MW | 12.4 | 122.0 | 40.6 | 127.0 | 7.1 | 225 | 150 | 240 | 5.67 | 7.88 | 10.35 | 9.41 | 250 | 9.89 | 402 |
|  |  |  | YB | R | | | MSP | 13.1 | 157.0 | 12.4 | 114.0 | 7.3 | 225 | 150 | 240 | 5.31 | 7.53 | 9.07 | 7.38 | 200 | 8.68 | 367 |
|  | 25.5°N,  106.1°E | 2008 | RE | R | | | MP | 22.4 | 114.5 | 17.4 | 125.0 | 5.9 | 180 | 120 | 180 | 5.97 | 7.40 | 8.14 | 8.06 | 196 | 8.17 | 383 |
|  |  |  | RE | R | | | MP | 25.6 | 112.4 | 15.6 | 108.0 | 5.7 | 210 | 135 | 210 | 8.21 | 9.64 | 10.63 | 9.94 | 197 | 10.43 | 301 |
|  |  |  | RE | R | | | MW | 18.9 | 98.6 | 16.5 | 109.0 | 5.9 | 210 | 135 | 210 | 7.77 | 9.22 | 9.42 | 9.48 | 205 | 9.58 | 340 |
|  |  |  | RE | R | | | MW | 19.8 | 153.2 | 18.9 | 98.0 | 5.5 | 225 | 150 | 240 | 5.35 | 9.42 | 9.92 | 10.26 | 247 | 10.51 | 373 |
|  | 26.1°N,  105.6°E | 2006 | RE | R | | | MW | 16.6 | 130.0 | 16.5 | 98.0 | 6.4 | 210 | 135 | 210 | 6.05 | 6.85 | 7.11 | 6.43 | 147 | 7.05 | 349 |
|  |  |  | YB | R | | | MW | 23.0 | 138.6 | 13.3 | 96.0 | 6.5 | 225 | 150 | 240 | 7.65 | 7.84 | 8.48 | 8.30 | 185 | 8.22 | 361 |
|  |  | 2007 | LRE | I | | | MW | 27.2 | 124.8 | 32.6 | 85.0 | 6.8 | 210 | 135 | 210 | 6.15 | 7.20 | 7.70 | 6.68 | 157 | 7.58 | 342 |
|  |  |  | RE | R | | | MSP | 19.6 | 117.0 | 8.4 | 74.0 | 7.1 | 210 | 135 | 210 | 5.90 | 7.73 | 7.46 | 6.58 | 155 | 7.76 | 330 |
|  |  |  | RE | R | | | MP | 12.6 | 156.0 | 15.9 | 149.0 | 7.1 | 210 | 135 | 210 | 6.00 | 6.92 | 7.58 | 7.60 | 234 | 7.59 | 488 |
|  |  |  | PS | R | | | MW | 12.6 | 76.0 | 13.8 | 136.0 | 7.3 | 180 | 120 | 180 | 4.84 | 5.42 | 5.62 | 5.88 | 221 | 5.78 | 606 |
|  |  |  | YE | R | | | MW | 26.7 | 107.0 | 21.4 | 105.0 | 7.2 | 225 | 150 | 240 | 7.54 | 9.41 | 9.30 | 9.16 | 196 | 9.57 | 327 |
|  |  |  | PS | R | | | MW | 22.9 | 108.0 | 22.5 | 135.0 | 5.9 | 180 | 120 | 180 | 5.47 | 5.98 | 6.21 | 6.34 | 182 | 6.24 | 469 |
|  |  |  | PS | R | | | MW | 25.1 | 115.0 | 23.6 | 104.0 | 5.9 | 210 | 135 | 210 | 4.67 | 6.35 | 7.54 | 6.72 | 201 | 7.30 | 438 |
|  |  | 2009 | YE | R | | | MW | 16.8 | 125.0 | 25.4 | 122.0 | 6.0 | 210 | 135 | 210 | 8.63 | 9.21 | 9.23 | 8.84 | 125 | 9.24 | 236 |
|  | 29.5°N,  114.2°E | 2008 | LRE | R | | | MW | 12.0 | 98.5 | 9.2 | 132.0 | 5.8 | 188 | 68 | 90 | 6.22 | 8.13 | 8.49 | 8.12 | 174 | 8.59 | 328 |
|  |  |  | LRE | R | | | MW | 17.1 | 111.0 | 8.8 | 152.0 | 5.8 | 188 | 81 | 68 | 4.56 | 6.49 | 6.86 | 7.16 | 213 | 7.16 | 472 |
|  |  | 2009 | LRE | I | | | MSP | 16.3 | 118.0 | 21.5 | 135.0 | 6.0 | 218 | 54 | 90 | 4.23 | 8.55 | 7.95 | 8.70 | 228 | 8.92 | 405 |
|  |  | 2010 | LRE | R | | | MW | 25.9 | 178.0 | 8.4 | 108.0 | 5.5 | 218 | 81 | 90 | 5.95 | 8.97 | 9.12 | 9.12 | 216 | 9.52 | 360 |
| Zunyi,  Guizhou | 28.5°N,  107.4°E | 2007 | RE | R | | | MW | 21.3 | 157.0 | 16.9 | 109.0 | 7.5 | 210 | 135 | 210 | 4.23 | 5.76 | 6.72 | 6.24 | 208 | 6.59 | 501 |
|  |  |  | RE | R | | | MW | 19.7 | 88.0 | 15.4 | 155.0 | 6.5 | 210 | 135 | 210 | 4.25 | 5.85 | 6.40 | 6.40 | 221 | 6.50 | 539 |
|  |  | 2008 | YE | R | | | MW | 21.0 | 87.0 | 21.9 | 58.0 | 6.4 | 225 | 150 | 225 | 4.91 | 6.39 | 5.93 | 5.71 | 164 | 6.25 | 429 |
|  |  |  | YB | R | | | MW | 24.3 | 90.0 | 11.0 | 89.0 | 6.5 | 210 | 135 | 210 | 6.54 | 7.86 | 7.92 | 6.84 | 147 | 8.02 | 307 |
|  |  | 2009 | PS | R | | | MW | 19.7 | 92.9 | 23.3 | 85.0 | 6.8 | 180 | 105 | 180 | 4.78 | 5.23 | 5.79 | 4.88 | 122 | 5.58 | 385 |
|  | 28.5°N,  107.5°E | 2007 | PS | R | | | MW | 19.8 | 67.8 | 21.2 | 192.0 | 5.8 | 225 | 150 | 225 | 7.36 | 8.37 | 8.60 | 8.25 | 178 | 8.60 | 334 |
|  |  | 2008 | PS | R | | | MP | 17.1 | 70.8 | 36.7 | 114.0 | 5.9 | 210 | 135 | 210 | 6.55 | 7.57 | 8.02 | 7.52 | 174 | 7.93 | 355 |
|  |  | 2009 | PS | R | | | MW | 18.3 | 128.0 | 9.8 | 111.0 | 6.4 | 195 | 135 | 210 | 6.83 | 7.72 | 8.58 | 8.53 | 230 | 8.54 | 427 |
|  |  | 2010 | PS | R | | | MW | 11.5 | 152.0 | 24.3 | 127.0 | 6.8 | 150 | 105 | 150 | 5.72 | 6.36 | 6.94 | 6.76 | 153 | 6.84 | 371 |
|  | 28.1°N,  106.5°E | 2007 | PS | R | | | MW | 22.9 | 141.8 | 14.4 | 114.0 | 6.5 | 210 | 135 | 210 | 4.91 | 6.23 | 6.30 | 6.39 | 198 | 6.47 | 488 |
|  |  |  | PS | R | | | MW | 22.3 | 182.0 | 14.0 | 116.0 | 6.4 | 210 | 135 | 210 | 7.00 | 9.84 | 9.67 | 9.46 | 192 | 10.10 | 304 |
|  |  | 2008 | RE | R | | | MW | 18.9 | 152.6 | 25.9 | 118.0 | 6.2 | 180 | 120 | 180 | 4.72 | 5.96 | 6.15 | 6.53 | 213 | 6.43 | 525 |
|  |  |  | RE | R | | | MW | 23.8 | 151.0 | 14.2 | 93.0 | 6.6 | 210 | 135 | 210 | 6.02 | 7.13 | 8.72 | 7.77 | 205 | 8.26 | 395 |
|  | 28.3°N,  107.5°E | 2005 | RE | I | | | MP | 9.6 | 83.0 | 16.9 | 125.0 | 8.1 | 180 | 120 | 180 | 5.03 | 7.12 | 7.03 | 7.03 | 170 | 7.35 | 376 |
|  |  |  | RE | R | | | MW | 12.3 | 85.0 | 13.8 | 134.0 | 5.9 | 210 | 135 | 210 | 6.19 | 8.05 | 8.94 | 7.61 | 177 | 8.76 | 326 |
|  |  | 2006 | RE | R | | | MSP | 14.9 | 120.0 | 22.5 | 124.0 | 7.9 | 210 | 135 | 210 | 4.65 | 7.19 | 8.07 | 7.30 | 200 | 8.06 | 395 |
|  |  |  | LRE | R | | | MW | 14.7 | 105.9 | 38.5 | 135.0 | 6.4 | 210 | 135 | 210 | 6.57 | 7.25 | 8.13 | 7.69 | 197 | 7.88 | 399 |
|  |  | 2007 | LRE | I | | | MW | 19.1 | 140.0 | 21.3 | 114.0 | 5.8 | 225 | 150 | 240 | 7.23 | 8.99 | 8.72 | 8.73 | 193 | 9.04 | 341 |
|  |  |  | LRE | R | | | MW | 17.5 | 168.0 | 26.5 | 124.0 | 5.8 | 210 | 135 | 210 | 6.38 | 7.78 | 8.55 | 7.85 | 188 | 8.40 | 359 |
|  |  | 2008 | LRE | R | | | MS | 25.6 | 135.6 | 38.4 | 126.0 | 6.5 | 225 | 150 | 240 | 4.98 | 7.06 | 8.46 | 6.70 | 194 | 8.10 | 383 |
|  | 28.3°N,  107.6°E | 2008 | YB | R | | | MW | 26.8 | 163.0 | 12.6 | 128.0 | 5.4 | 210 | 135 | 210 | 5.98 | 7.11 | 7.93 | 7.19 | 183 | 7.72 | 381 |
|  |  |  | YB | R | | | MSP | 15.6 | 122.0 | 25.6 | 127.0 | 5.9 | 225 | 150 | 240 | 3.31 | 5.20 | 6.31 | 5.94 | 235 | 6.23 | 598 |
|  |  |  | YB | R | | | MW | 26.0 | 137.0 | 15.5 | 115.0 | 5.6 | 180 | 120 | 180 | 6.32 | 7.65 | 7.90 | 7.66 | 162 | 7.97 | 333 |
|  |  | 2009 | YB | R | | | MW | 24.4 | 159.0 | 22.4 | 113.0 | 6.9 | 180 | 120 | 180 | 5.60 | 7.78 | 7.63 | 7.43 | 161 | 7.96 | 332 |
|  | 28.2°N,  106.1°E | 2006 |  | R | | | MW | 18.3 | 98.0 | 13.8 | 126.0 | 5.8 | 180 | 120 | 180 | 4.50 | 5.43 | 6.45 | 5.80 | 170 | 6.17 | 448 |
|  |  |  | YE | R | | | MW | 9.3 | 80.0 | 13.9 | 145.0 | 6.7 | 210 | 135 | 210 | 7.40 | 8.65 | 9.20 | 8.73 | 186 | 9.13 | 327 |
|  |  | 2007 | YE | R | | | MW | 20.3 | 72.0 | 17.4 | 171.0 | 6.2 | 225 | 150 | 240 | 6.35 | 7.83 | 8.70 | 8.73 | 257 | 8.76 | 467 |
|  |  |  | YE | R | | | MW | 21.6 | 68.0 | 15.7 | 111.0 | 6.8 | 180 | 150 | 240 | 6.93 | 7.80 | 9.45 | 9.18 | 238 | 9.29 | 406 |
|  |  | 2008 | CS | R | | | MP | 13.0 | 79.0 | 16.2 | 117.0 | 5.8 | 225 | 150 | 240 | 7.63 | 8.61 | 9.93 | 8.90 | 201 | 9.50 | 337 |
|  |  |  | CS | R | | | MW | 13.9 | 80.0 | 8.7 | 79.0 | 6.7 | 210 | 135 | 210 | 6.20 | 9.11 | 10.18 | 10.28 | 241 | 10.43 | 366 |
|  |  | 2009 | YE | R | | | MW | 13.8 | 83.0 | 11.2 | 198.0 | 6.8 | 210 | 135 | 210 | 7.03 | 7.83 | 8.03 | 8.25 | 220 | 8.15 | 428 |
|  |  |  | PS | R | | | MW | 18.9 | 87.0 | 8.3 | 191.0 | 6.8 | 210 | 135 | 210 | 5.70 | 8.85 | 8.93 | 6.83 | 163 | 9.23 | 289 |
|  | 27.1°N,  107.5°E | 2005 | PS | R | | | MW | 19.6 | 112.9 | 16.9 | 124.0 | 6.5 | 225 | 150 | 240 | 6.30 | 7.70 | 9.10 | 8.00 | 206 | 8.70 | 376 |
|  |  |  | PS | R | | | MW | 20.5 | 105.0 | 25.3 | 154.0 | 6.8 | 225 | 150 | 240 | 4.80 | 7.15 | 8.67 | 7.75 | 224 | 8.43 | 421 |
|  |  | 2006 | PS | R | | | MW | 23.9 | 109.0 | 27.4 | 145.0 | 6.3 | 180 | 120 | 180 | 4.37 | 5.20 | 7.03 | 6.65 | 237 | 6.78 | 554 |
|  |  |  | PS | R | | | MW | 26.4 | 147.0 | 31.4 | 135.0 | 6.4 | 210 | 135 | 210 | 3.88 | 6.55 | 7.47 | 6.80 | 205 | 7.49 | 435 |
|  |  | 2007 | PS | R | | | MW | 23.1 | 116.0 | 33.5 | 136.0 | 6.5 | 225 | 150 | 240 | 6.00 | 8.25 | 9.47 | 8.95 | 232 | 9.38 | 392 |
|  |  |  | PS | R | | | MW | 32.2 | 131.5 | 26.9 | 125.0 | 6.5 | 180 | 120 | 180 | 4.20 | 6.45 | 6.47 | 6.23 | 166 | 6.75 | 401 |
|  |  | 2008 | PS | I | | | MW | 27.3 | 91.8 | 26.7 | 124.0 | 7.0 | 210 | 135 | 210 | 5.15 | 6.55 | 7.35 | 7.15 | 218 | 7.31 | 473 |
|  |  |  | PS | R | | | MP | 26.3 | 145.6 | 17.0 | 136.0 | 6.4 | 180 | 120 | 180 | 4.55 | 5.43 | 6.35 | 6.05 | 190 | 6.18 | 492 |
|  |  | 2009 | PS | R | | | MW | 18.6 | 94.0 | 32.1 | 145.0 | 6.5 | 210 | 135 | 210 | 5.45 | 6.25 | 8.15 | 7.18 | 216 | 7.57 | 452 |
|  |  |  | YE | R | | | MW | 30.8 | 90.0 | 28.3 | 162.0 | 6.3 | 225 | 150 | 240 | 6.15 | 7.13 | 8.93 | 8.03 | 230 | 8.41 | 433 |
| Qian'nan,  Guizhou | 26.1°N,  107.3°E | 2007 | YE | R | | | MW | 18.7 | 79.6 | 27.9 | 150.0 | 5.9 | 210 | 135 | 210 | 5.30 | 7.60 | 7.77 | 7.48 | 195 | 8.00 | 389 |
|  |  |  | YE | R | | | MW | 13.2 | 89.5 | 21.6 | 98.0 | 7.4 | 225 | 150 | 240 | 5.52 | 8.39 | 8.85 | 7.75 | 196 | 8.98 | 348 |
|  |  |  | YB | R | | | MW | 20.9 | 98.4 | 44.9 | 142.0 | 5.9 | 210 | 135 | 210 | 6.40 | 7.60 | 9.99 | 8.55 | 211 | 9.24 | 362 |
|  |  |  | YE | R | | | MW | 21.5 | 87.6 | 25.8 | 94.0 | 6.4 | 210 | 135 | 210 | 5.21 | 7.77 | 8.25 | 7.82 | 201 | 8.42 | 380 |
|  |  |  | YE | R | | | MW | 14.9 | 88.8 | 27.2 | 123.0 | 6.5 | 225 | 150 | 240 | 5.08 | 7.55 | 7.87 | 7.71 | 219 | 8.11 | 428 |
|  |  |  | YE | R | | | MW | 11.5 | 141.0 | 34.9 | 79.0 | 5.6 | 180 | 120 | 180 | 5.80 | 8.39 | 9.07 | 8.58 | 177 | 9.18 | 311 |
|  |  | 2009 | YE | R | | | MW | 15.5 | 96.0 | 26.9 | 90.0 | 7.8 | 225 | 150 | 240 | 4.56 | 6.38 | 7.88 | 6.58 | 207 | 7.49 | 439 |
|  | 26.5°N,  107.5°E | 2006 | LS | R | | | MW | 18.3 | 125.3 | 24.5 | 106.0 | 7.5 | 210 | 135 | 210 | 6.29 | 8.99 | 8.52 | 8.71 | 196 | 9.08 | 344 |
|  |  |  | YE | R | | | MW | 17.2 | 98.6 | 25.3 | 83.0 | 7.4 | 225 | 150 | 240 | 5.96 | 7.08 | 8.22 | 7.85 | 236 | 8.02 | 466 |
|  |  | 2007 | YE | R | | | MW | 20.6 | 96.2 | 17.6 | 117.0 | 6.4 | 210 | 135 | 210 | 8.70 | 9.14 | 9.15 | 9.12 | 118 | 9.11 | 230 |
|  |  |  | Lato. | R | | | MW | 12.2 | 159.2 | 16.8 | 79.0 | 6.6 | 180 | 120 | 180 | 3.66 | 6.36 | 8.42 | 7.89 | 207 | 8.25 | 399 |
|  |  | 2008 | Lato. | R | | | MW | 11.5 | 68.7 | 27.8 | 94.0 | 7.0 | 210 | 135 | 210 | 4.70 | 7.82 | 7.57 | 7.73 | 205 | 8.12 | 401 |
|  |  |  | YB | R | | | MW | 10.6 | 122.7 | 8.4 | 90.0 | 7.1 | 210 | 135 | 210 | 3.52 | 5.59 | 6.87 | 6.25 | 215 | 6.71 | 508 |
|  | 25.5°N,  107.2°E | 2008 | YE | R | | | MW | 17.6 | 154.3 | 17.8 | 132.0 | 7.6 | 180 | 120 | 180 | 6.12 | 6.72 | 7.29 | 6.73 | 144 | 7.10 | 341 |
|  |  |  | YE | I | | | MW | 14.300 | 145.3 | 5.5 | 100.0 | 7.5 | 180 | 120 | 180 | 3.23 | 5.15 | 6.71 | 6.21 | 199 | 6.52 | 486 |
|  |  |  | YE | R | | | MW | 14.800 | 154.3 | 15.5 | 120.0 | 8.0 | 225 | 150 | 240 | 6.56 | 10.07 | 9.23 | 9.42 | 206 | 10.02 | 327 |
|  |  | 2009 | YE | R | | | MW | 12.4 | 89.7 | 18.4 | 190.0 | 6.5 | 210 | 135 | 210 | 3.60 | 7.38 | 7.47 | 7.71 | 218 | 8.07 | 428 |
|  | 27.0°N,  107.3°E | 2006 | YE | R | | | MS | 25.3 | 123.9 | 22.0 | 55.0 | 7.8 | 180 | 120 | 180 | 5.02 | 6.69 | 7.98 | 7.60 | 198 | 7.85 | 402 |
|  |  | 2007 | YE | I | | | MW | 26.9 | 152.4 | 13.3 | 122.0 | 7.6 | 225 | 150 | 240 | 4.03 | 6.97 | 8.13 | 7.85 | 241 | 8.23 | 464 |
|  |  |  | YE | R | | | MW | 20.9 | 127.6 | 22.0 | 125.0 | 7.5 | 180 | 120 | 180 | 6.52 | 8.41 | 9.48 | 8.45 | 166 | 9.27 | 292 |
|  |  |  | YE | R | | | MW | 23.6 | 125.0 | 16.9 | 126.0 | 7.6 | 180 | 120 | 180 | 7.00 | 8.88 | 9.27 | 8.48 | 155 | 9.32 | 275 |
|  |  | 2008 | YB | R | | | MW | 21.8 | 88.0 | 15.0 | 196.0 | 7.7 | 210 | 135 | 210 | 6.26 | 8.90 | 10.35 | 9.14 | 201 | 10.12 | 316 |
|  |  |  | YB | R | | | MW | 18.0 | 105.0 | 17.8 | 134.0 | 7.9 | 225 | 150 | 240 | 6.14 | 8.64 | 9.84 | 8.98 | 218 | 9.71 | 356 |
| Guiyang,  Guizhou | 27.0°N,  106.6°E | 2006 | YB | R | | | MW | 21.4 | 132.6 | 28.1 | 84.0 | 6.5 | 158 | 101 | 129 | 7.21 | 8.34 | 8.30 | 8.01 | 126 | 8.43 | 261 |
|  |  |  | YB | R | | | MS | 17.0 | 114.3 | 30.5 | 152.0 | 6.5 | 152 | 129 | 126 | 5.11 | 9.40 | 8.25 | 8.48 | 141 | 9.27 | 257 |
| Qianxi,  Guizhou | 25.1°N,  104.5°E | 2006 | YB | R | | | MW | 10.9 | 72.0 | 9.7 | 98.0 | 6.9 | 210 | 135 | 210 | 7.45 | 7.95 | 9.03 | 8.72 | 228 | 8.78 | 411 |
|  |  |  | YB | R | | | MW | 14.4 | 75.0 | 9.0 | 78.0 | 5.4 | 180 | 120 | 180 | 7.05 | 8.08 | 10.20 | 8.94 | 182 | 9.54 | 307 |
|  |  | 2007 | YB | R | | | MW | 17.2 | 138.0 | 44.0 | 85.0 | 5.1 | 225 | 150 | 240 | 3.93 | 5.82 | 6.35 | 6.32 | 232 | 6.49 | 566 |
|  |  |  | YB | R | | | MW | 13.8 | 88.0 | 8.2 | 86.0 | 5.6 | 210 | 135 | 210 | 3.04 | 5.39 | 5.56 | 5.58 | 208 | 5.86 | 564 |
|  |  | 2008 | YB | R | | | MW | 29.0 | 153.0 | 26.1 | 108.0 | 6.5 | 180 | 120 | 180 | 3.43 | 4.88 | 5.54 | 5.28 | 181 | 5.52 | 528 |
|  |  |  | YB | R | | | MW | 15.4 | 121.0 | 16.8 | 105.0 | 6.1 | 210 | 135 | 210 | 4.33 | 5.33 | 7.43 | 6.12 | 206 | 6.75 | 485 |
|  |  | 2009 | YE | I | | | MW | 12.8 | 88.7 | 11.6 | 118.0 | 6.6 | 180 | 120 | 180 | 4.68 | 5.84 | 7.89 | 6.29 | 168 | 7.20 | 380 |
|  |  |  | PS | R | | | MW | 10.2 | 76.1 | 21.9 | 125.0 | 7.0 | 210 | 135 | 210 | 5.49 | 6.62 | 6.82 | 6.29 | 163 | 6.84 | 390 |
|  | 25.1°N,  104.6°E | 2007 | YE | R | | | MW | 18.1 | 70.0 | 13.9 | 165.0 | 7.1 | 210 | 135 | 210 | 3.67 | 4.96 | 6.95 | 5.31 | 191 | 6.28 | 487 |
|  |  |  | YE | R | | | MW | 23.7 | 147.0 | 9.8 | 156.0 | 6.6 | 225 | 150 | 240 | 4.79 | 7.54 | 7.78 | 6.89 | 195 | 7.99 | 390 |
|  |  | 2008 | FAS | R | | | MW | 14.9 | 105.0 | 13.9 | 189.0 | 6.5 | 225 | 150 | 240 | 4.14 | 7.02 | 8.43 | 7.22 | 215 | 8.24 | 414 |
|  |  |  | FAS | R | | | MW | 25.7 | 133.0 | 14.8 | 178.0 | 6.8 | 225 | 150 | 240 | 4.27 | 6.01 | 6.63 | 6.49 | 228 | 6.69 | 540 |
|  |  | 2009 | RE | R | | | MW | 13.4 | 98.5 | 8.9 | 154.0 | 6.9 | 225 | 150 | 240 | 4.90 | 5.50 | 6.88 | 6.22 | 223 | 6.45 | 548 |
|  |  |  | FAS | R | | | MW | 28.5 | 145.6 | 17.6 | 87.0 | 6.4 | 225 | 150 | 240 | 4.95 | 5.88 | 7.29 | 5.92 | 188 | 6.79 | 444 |
|  |  | 2010 | YE | R | | | MW | 12.2 | 76.0 | 13.6 | 78.0 | 7.2 | 180 | 120 | 180 | 5.74 | 6.43 | 7.77 | 6.92 | 170 | 7.33 | 377 |
|  | 25.0°N,  104.6°E | 2008 | YE | R | | | MW | 20.4 | 171.0 | 26.5 | 106.0 | 7.1 | 180 | 120 | 180 | 5.45 | 6.16 | 6.95 | 6.64 | 180 | 6.77 | 428 |
|  |  |  | YB | R | | | MW | 11.1 | 155.0 | 19.8 | 124.0 | 7.3 | 210 | 135 | 210 | 5.99 | 6.94 | 7.88 | 6.63 | 164 | 7.56 | 355 |
|  |  | 2009 | YE | R | | | MW | 17.4 | 73.0 | 12.0 | 152.0 | 7.4 | 210 | 135 | 210 | 6.30 | 6.84 | 7.93 | 7.48 | 209 | 7.61 | 436 |
|  |  |  | CS | R | | | MP | 11.5 | 104.0 | 16.2 | 145.0 | 7.2 | 225 | 120 | 150 | 6.63 | 7.89 | 9.62 | 7.79 | 190 | 9.02 | 337 |
|  |  |  | CS | R | | | MW | 14.1 | 124.0 | 16.1 | 135.0 | 6.5 | 210 | 105 | 120 | 5.08 | 6.16 | 8.13 | 7.00 | 210 | 7.53 | 443 |
|  |  | 2010 | YE | R | | | MW | 26.1 | 129.0 | 10.3 | 154.0 | 6.4 | 210 | 105 | 120 | 6.45 | 8.67 | 9.64 | 9.05 | 207 | 9.59 | 343 |
|  |  |  | PS | R | | | MW | 24.3 | 132.0 | 14.8 | 136.0 | 6.3 | 180 | 90 | 90 | 5.96 | 7.31 | 10.60 | 9.26 | 211 | 9.76 | 343 |
|  |  |  | PS | R | | | MW | 20.6 | 165.0 | 27.5 | 155.0 | 6.9 | 225 | 120 | 150 | 7.10 | 8.05 | 9.66 | 8.23 | 194 | 9.08 | 341 |
| Tongren,  Guizhou | 27.4°N,  109.1°E | 2008 | PS | R | | | MW | 29.1 | 114.0 | 17.3 | 97.0 | 7.9 | 210 | 135 | 210 | 8.42 | 9.47 | 9.84 | 9.52 | 181 | 9.81 | 297 |
|  |  |  | PS | R | | | MW | 23.2 | 128.7 | 9.9 | 106.0 | 8.1 | 210 | 135 | 210 | 4.90 | 6.74 | 7.71 | 6.92 | 196 | 7.56 | 414 |
|  |  |  | PS | R | | | MW | 20.9 | 112.0 | 14.8 | 120.0 | 7.9 | 210 | 135 | 210 | 7.73 | 8.68 | 8.63 | 8.10 | 142 | 8.72 | 275 |
|  | 27.6°N,  108.2°E | 2006 | PS | R | | | MW | 18.9 | 148.9 | 18.4 | 115.0 | 7.2 | 210 | 135 | 210 | 7.24 | 7.97 | 8.87 | 8.38 | 195 | 8.61 | 362 |
|  |  |  | PS | R | | | MW | 17.8 | 154.9 | 13.3 | 85.0 | 7.3 | 180 | 120 | 180 | 7.52 | 9.63 | 8.87 | 8.06 | 129 | 9.42 | 237 |
|  |  | 2007 | PS | I | | | MW | 23.6 | 125.0 | 16.9 | 197.4 | 7.0 | 210 | 135 | 210 | 6.77 | 8.77 | 9.33 | 9.01 | 204 | 9.40 | 345 |
|  |  |  | PS | R | | | MP | 18.0 | 105.0 | 16.9 | 132.0 | 7.5 | 210 | 135 | 210 | 3.73 | 7.78 | 9.65 | 7.42 | 194 | 9.37 | 331 |
|  |  | 2008 | PS | R | | | MW | 25.9 | 109.0 | 27.6 | 130.0 | 5.8 | 180 | 120 | 180 | 7.79 | 8.04 | 8.33 | 8.08 | 110 | 8.18 | 245 |
|  | 27.6°N,  108.3°E | 2008 | YE | R | | | MW | 20.9 | 113.0 | 11.0 | 140.0 | 7.5 | 210 | 135 | 210 | 5.94 | 7.54 | 8.08 | 6.96 | 169 | 8.01 | 343 |
|  |  |  | YE | R | | | MW | 22.0 | 148.0 | 11.4 | 90.0 | 7.7 | 210 | 135 | 210 | 6.69 | 7.21 | 7.54 | 7.00 | 140 | 7.41 | 320 |
|  |  | 2009 | YE | R | | | MW | 16.9 | 129.0 | 9.2 | 63.0 | 6.0 | 225 | 150 | 240 | 6.77 | 7.60 | 8.13 | 6.83 | 152 | 7.97 | 317 |
|  |  | 2010 | YB | R | | | MW | 20.5 | 107.0 | 16.5 | 150.0 | 6.6 | 225 | 150 | 240 | 6.33 | 7.83 | 8.17 | 7.10 | 172 | 8.17 | 342 |
|  |  |  | YE | R | | | MW | 26.1 | 104.6 | 9.3 | 103.0 | 7.7 | 225 | 150 | 240 | 7.13 | 8.65 | 9.52 | 8.82 | 205 | 9.36 | 348 |
|  | 28.1°N,  109.1°E | 2005 | YE | R | | | MW | 20.9 | 116.0 | 10.3 | 161.0 | 7.6 | 180 | 120 | 180 | 4.89 | 8.42 | 8.00 | 7.38 | 158 | 8.58 | 303 |
|  |  | 2006 | YE | R | | | MW | 21.6 | 124.0 | 23.1 | 90.0 | 7.2 | 180 | 120 | 180 | 5.79 | 8.14 | 8.87 | 8.87 | 197 | 9.06 | 347 |
|  |  |  | YE | R | | | MW | 25.4 | 169.0 | 14.3 | 200.0 | 7.6 | 150 | 120 | 180 | 5.04 | 7.20 | 8.47 | 8.54 | 186 | 8.62 | 346 |
|  |  |  | LS | R | | | MW | 27.5 | 116.8 | 18.7 | 130.0 | 6.6 | 150 | 120 | 150 | 4.67 | 7.25 | 8.44 | 8.12 | 164 | 8.48 | 316 |
|  |  | 2007 | YE | R | | | MW | 16.9 | 88.0 | 17.1 | 160.0 | 7.5 | 210 | 120 | 120 | 5.97 | 8.37 | 9.01 | 8.95 | 221 | 9.20 | 381 |
|  |  |  | YE | R | | | MW | 18.9 | 88.0 | 8.1 | 102.1 | 7.1 | 180 | 120 | 180 | 5.82 | 7.45 | 8.54 | 8.59 | 221 | 8.63 | 406 |
|  | 28.1°N,  109.2°E | 2008 | Lato. | R | | | MW | 22.4 | 92.0 | 31.3 | 62.0 | 7.1 | 240 | 90 | 210 | 4.54 | 7.74 | 8.42 | 7.71 | 228 | 8.57 | 421 |
|  |  |  | Lato. | R | | | MW | 29.6 | 161.0 | 43.9 | 85.0 | 6.5 | 150 | 90 | 180 | 6.34 | 8.10 | 8.44 | 8.47 | 157 | 8.63 | 300 |
|  |  |  | YB | R | | | MW | 25.8 | 157.0 | 22.8 | 105.0 | 6.6 | 180 | 120 | 180 | 4.80 | 7.74 | 8.47 | 8.50 | 197 | 8.76 | 359 |
|  |  | 2009 | YE | R | | | MW | 25.2 | 91.0 | 33.6 | 117.0 | 6.8 | 180 | 120 | 150 | 5.93 | 7.44 | 8.75 | 7.53 | 163 | 8.39 | 318 |
|  |  |  | YE | I | | | MW | 21.7 | 117.0 | 8.5 | 96.0 | 6.9 | 180 | 120 | 150 | 5.65 | 7.08 | 7.99 | 7.94 | 208 | 8.02 | 412 |
|  |  | 2010 | YE | R | | | MW | 22.2 | 164.0 | 16.2 | 118.0 | 6.2 | 210 | 120 | 180 | 4.70 | 7.92 | 8.67 | 8.49 | 219 | 8.93 | 389 |
|  | 27.6°N,  108.2°E | 2008 | YE | R | | | MW | 22.0 | 97.0 | 38.8 | 136.0 | 6.3 | 210 | 150 | 420 | 7.51 | 8.59 | 9.00 | 8.04 | 156 | 8.92 | 289 |
|  |  | 2009 | YE | R | | | MS | 21.0 | 149.9 | 24.0 | 156.0 | 6.2 | 225 | 150 | 240 | 6.83 | 7.60 | 9.33 | 8.77 | 257 | 8.94 | 458 |
|  |  |  | PS | R | | | MW | 20.9 | 146.9 | 32.1 | 108.0 | 6.5 | 225 | 150 | 240 | 3.68 | 6.46 | 6.05 | 5.93 | 200 | 6.56 | 486 |
| Zunyi,  Guizhou | 28.3°N,  107.3°E | 2006 | YE | R | | | MW | 23.4 | 89.2 | 15.9 | 98.0 | 6.7 | 210 | 135 | 210 | 4.14 | 4.86 | 5.71 | 5.42 | 211 | 5.52 | 607 |
|  |  | 2007 | LS | R | | | MW | 21.3 | 126.0 | 21.0 | 109.0 | 6.6 | 210 | 135 | 210 | 4.18 | 6.40 | 6.91 | 6.86 | 217 | 7.10 | 484 |
|  |  |  | LS | R | | | MW | 24.0 | 156.0 | 10.0 | 89.0 | 6.9 | 210 | 135 | 210 | 7.34 | 7.85 | 7.63 | 7.26 | 95 | 7.74 | 236 |
|  |  | 2008 | YE | R | | | MW | 30.1 | 150.0 | 20.1 | 165.0 | 6.7 | 180 | 120 | 180 | 4.85 | 5.57 | 5.75 | 5.57 | 145 | 5.73 | 425 |
|  |  |  | YE | R | | | MW | 22.7 | 104.9 | 20.1 | 98.0 | 6.4 | 158 | 150 | 240 | 5.12 | 6.91 | 6.32 | 5.95 | 120 | 6.75 | 314 |
|  |  | 2009 | YE | R | | | MW | 20.3 | 187.4 | 13.7 | 96.0 | 6.5 | 180 | 120 | 165 | 5.03 | 8.14 | 7.63 | 8.19 | 185 | 8.34 | 356 |
|  |  |  | YE | R | | | MW | 20.9 | 132.0 | 8.9 | 87.0 | 7.1 | 210 | 120 | 150 | 6.19 | 9.96 | 8.94 | 8.44 | 175 | 9.79 | 289 |
|  | 27.4°N,  106.6°E | 2010 | YE | R | | | MW | 17.9 | 145.3 | 19.8 | 98.0 | 6.5 | 225 | 150 | 240 | 7.73 | 8.72 | 9.58 | 8.94 | 199 | 9.34 | 339 |
|  |  |  | FAS | R | | | MW | 30.7 | 169.8 | 16.0 | 97.0 | 6.5 | 180 | 105 | 180 | 6.08 | 7.33 | 8.56 | 8.27 | 204 | 8.41 | 386 |
|  |  |  | FAS | R | | | MW | 28.1 | 116.5 | 13.0 | 69.0 | 6.9 | 180 | 105 | 180 | 5.27 | 6.20 | 7.14 | 7.20 | 234 | 7.20 | 515 |
|  |  |  | FAS | R | | | MW | 27.3 | 152.9 | 14.9 | 95.0 | 7.0 | 210 | 135 | 210 | 6.32 | 7.87 | 9.11 | 9.10 | 257 | 9.14 | 448 |
|  |  |  | FAS | R | | | MW | 25.6 | 106.8 | 42.0 | 85.0 | 7.0 | 180 | 105 | 180 | 6.50 | 8.40 | 9.26 | 9.34 | 210 | 9.41 | 354 |
|  |  |  | FAS | R | | | MW | 16.9 | 114.3 | 35.9 | 106.0 | 6.8 | 210 | 135 | 210 | 6.27 | 7.00 | 8.00 | 7.90 | 252 | 7.91 | 506 |
| Baise,  Guangxi | 23.1°N,  106.3°E | 2006 | FAS | R | | | MW | 11.2 | 101.0 | 22.1 | 75.0 | 5.8 | 240 | 75 | 180 | 2.76 | 4.34 | 4.56 | 4.09 | 200 | 4.63 | 688 |
|  |  |  | FAS | R | | | MW | 15.8 | 130.0 | 34.5 | 145.0 | 5.8 | 240 | 75 | 180 | 7.49 | 10.42 | 10.60 | 9.25 | 197 | 10.83 | 290 |
|  |  | 2007 | FAS | R | | | MW | 18.2 | 101.8 | 10.5 | 187.0 | 5.4 | 195 | 60 | 150 | 3.97 | 4.57 | 5.81 | 5.28 | 201 | 5.46 | 586 |
|  |  |  | FAS | R | | | MW | 14.0 | 60.2 | 24.3 | 118.0 | 5.9 | 240 | 75 | 180 | 5.06 | 6.73 | 9.33 | 6.85 | 212 | 8.43 | 399 |
|  |  | 2008 | CS | I | | | MW | 12.8 | 65.0 | 25.0 | 114.0 | 5.6 | 240 | 75 | 180 | 3.37 | 4.40 | 4.67 | 4.52 | 205 | 4.68 | 696 |
|  |  |  | FAS | R | | | MW | 10.8 | 150.0 | 11.4 | 112.0 | 5.3 | 150 | 45 | 120 | 5.51 | 5.89 | 7.16 | 5.70 | 115 | 6.64 | 311 |
|  |  | 2009 | PS | R | | | MW | 16.7 | 97.0 | 27.7 | 93.0 | 5.8 | 150 | 45 | 120 | 7.23 | 8.76 | 10.86 | 10.14 | 175 | 10.46 | 271 |
|  |  |  | YE | R | | | MW | 13.5 | 74.1 | 42.6 | 98.0 | 6.7 | 195 | 60 | 150 | 7.34 | 7.65 | 8.50 | 7.29 | 129 | 8.15 | 274 |
|  |  | 2010 | LS | R | | | MW | 18.9 | 86.2 | 12.5 | 166.0 | 6.2 | 240 | 75 | 180 | 7.27 | 7.54 | 7.78 | 7.43 | 112 | 7.61 | 266 |
|  |  |  | FAS | R | | | MW | 17.5 | 82.6 | 25.0 | 105.0 | 5.9 | 240 | 75 | 180 | 6.77 | 7.77 | 8.08 | 8.28 | 252 | 8.19 | 489 |
|  | 23.2°N,  107.4°E | 2007 | FAS | R | | | MW | 28.0 | 148.8 | 16.3 | 108.0 | 6.4 | 240 | 75 | 180 | 7.50 | 8.28 | 8.26 | 8.09 | 162 | 8.30 | 320 |
|  |  |  | YE | R | | | MW | 21.8 | 108.3 | 37.5 | 150.0 | 6.3 | 240 | 75 | 180 | 4.34 | 5.21 | 5.73 | 5.34 | 200 | 5.60 | 569 |
|  |  |  | YE | R | | | MW | 21.0 | 117.0 | 17.2 | 118.0 | 6.7 | 195 | 60 | 150 | 4.83 | 5.11 | 5.72 | 5.73 | 237 | 5.68 | 661 |
| Changde,  Hu'nan | 29.4°N,  111.2°E | 2008 | YE | R | | | MW | 24.0 | 102.3 | 37.4 | 96.0 | 5.1 | 200 | 108 | 108 | 5.74 | 6.53 | 7.50 | 6.81 | 178 | 7.20 | 399 |
|  |  | 2009 | FAS | R | | | MW | 36.9 | 114.5 | 13.1 | 64.0 | 5.6 | 180 | 75 | 90 | 4.71 | 6.75 | 7.64 | 7.88 | 221 | 7.89 | 444 |
|  |  | 2010 | Sier. | R | | | MW | 34.2 | 153.6 | 8.0 | 146.0 | 6.1 | 180 | 75 | 90 | 6.35 | 7.82 | 7.56 | 7.56 | 154 | 7.83 | 326 |
|  |  |  | BE | I | | | MW | 28.2 | 156.0 | 17.6 | 78.0 | 5.3 | 180 | 75 | 90 | 5.64 | 5.71 | 6.22 | 6.18 | 184 | 6.08 | 486 |
| Chenzhou,  Hu'nan | 25.2°N,  112.3°E | 2007 | BE | R | | | MW | 16.4 | 78.5 | 26.6 | 108.0 | 7.4 | 180 | 75 | 90 | 3.38 | 4.56 | 5.93 | 4.85 | 166 | 5.52 | 491 |
|  |  |  | BE | R | | | MW | 17.7 | 105.6 | 25.4 | 108.0 | 7.0 | 180 | 75 | 90 | 4.98 | 6.60 | 7.83 | 7.19 | 183 | 7.62 | 386 |
|  |  | 2008 | LRE | R | | | MW | 11.2 | 89.4 | 20.5 | 110.0 | 7.0 | 180 | 75 | 75 | 4.93 | 6.06 | 7.25 | 6.63 | 181 | 6.97 | 418 |
|  |  | 2009 | YE | R | | | MW | 15.7 | 87.6 | 15.3 | 112.0 | 6.1 | 180 | 75 | 90 | 3.38 | 4.56 | 5.93 | 4.85 | 166 | 5.52 | 491 |
|  |  |  | YE | R | | | MW | 10.7 | 91.6 | 19.9 | 113.0 | 6.2 | 180 | 75 | 90 | 4.38 | 6.06 | 7.45 | 6.71 | 184 | 7.18 | 412 |
|  |  | 2010 | YE | R | | | MW | 12.6 | 93.5 | 34.6 | 113.0 | 6.3 | 180 | 75 | 90 | 4.29 | 5.13 | 7.01 | 6.03 | 187 | 6.44 | 466 |
|  | 25.2°N,  112.6°E | 2008 | FAS | R | | | MW | 21.2 | 125.9 | 15.9 | 114.0 | 7.4 | 180 | 75 | 90 | 5.67 | 6.45 | 7.43 | 6.81 | 168 | 7.14 | 383 |
| Chongzuo,  Guangxi | 23.0°N,  107.1°E | 2007 | FAS | R | | | MW | 29.2 | 151.0 | 17.0 | 151.0 | 5.9 | 150 | 45 | 120 | 4.91 | 4.97 | 5.19 | 4.35 | 63 | 5.13 | 286 |
|  |  |  | YE | R | | | MW | 32.1 | 150.0 | 10.9 | 73.0 | 5.8 | 240 | 75 | 150 | 9.87 | 10.72 | 10.57 | 10.12 | 145 | 10.68 | 228 |
|  |  |  | LRE | R | | | MW | 20.1 | 152.4 | 14.5 | 116.0 | 5.5 | 195 | 60 | 150 | 3.41 | 4.15 | 4.37 | 4.32 | 170 | 4.37 | 632 |
|  |  |  | LRE | R | | | MW | 26.0 | 113.5 | 15.6 | 192.0 | 5.5 | 150 | 45 | 120 | 2.83 | 4.11 | 4.69 | 4.40 | 148 | 4.66 | 531 |
| Nan'ning,  Guangxi | 23.1°N,  107.4°E | 2007 | PS | R | | | MW | 18.3 | 110.3 | 35.3 | 97.0 | 5.8 | 195 | 60 | 150 | 5.73 | 7.02 | 6.53 | 6.54 | 145 | 6.85 | 355 |
|  |  |  | PS | R | | | MW | 19.3 | 98.7 | 12.6 | 185.0 | 6.7 | 240 | 75 | 180 | 6.15 | 7.58 | 7.43 | 7.47 | 204 | 7.65 | 424 |
|  | 23.4°N,  108.1°E | 2007 | YB | R | | | MW | 16.5 | 89.6 | 13.6 | 82.0 | 6.8 | 195 | 60 | 150 | 3.69 | 4.17 | 4.29 | 4.43 | 164 | 4.29 | 625 |
|  |  |  | YB | R | | | MW | 17.8 | 78.9 | 14.8 | 112.0 | 6.8 | 240 | 75 | 180 | 5.11 | 5.30 | 5.51 | 5.04 | 89 | 5.35 | 328 |
|  |  |  | PS | R | | | MW | 18.9 | 88.4 | 15.3 | 100.0 | 6.6 | 150 | 45 | 120 | 3.79 | 4.76 | 5.03 | 4.96 | 145 | 5.08 | 479 |
| Chenzhou,  Hu'nan | 25.5°N,  112.4°E | 2005 | PS | R | | | MW | 13.2 | 107.0 | 14.0 | 116.0 | 6.9 | 180 | 90 | 105 | 6.54 | 8.13 | 9.36 | 9.18 | 209 | 9.31 | 356 |
|  |  | 2006 | PS | R | | | MW | 18.2 | 71.0 | 9.3 | 136.0 | 7.0 | 180 | 90 | 90 | 6.38 | 8.02 | 9.38 | 9.20 | 214 | 9.33 | 364 |
|  |  | 2007 | CS | R | | | MW | 9.8 | 156.0 | 17.3 | 125.0 | 6.8 | 180 | 90 | 90 | 6.39 | 8.25 | 9.48 | 9.18 | 200 | 9.41 | 339 |
|  |  | 2008 | CS | R | | | MW | 15.1 | 89.0 | 21.0 | 136.0 | 6.5 | 180 | 90 | 90 | 6.27 | 8.08 | 9.29 | 9.00 | 200 | 9.22 | 345 |
|  |  | 2009 | CS | R | | | MW | 13.9 | 79.0 | 16.9 | 89.0 | 6.3 | 180 | 90 | 90 | 6.25 | 7.86 | 9.20 | 9.02 | 213 | 9.14 | 370 |
| Hengyang,  Hu'nan | 26.4°N,  112.4°E | 2009 | PS | R | | | MW | 18.1 | 97.0 | 24.2 | 125.0 | 7.0 | 150 | 36 | 75 | 4.68 | 5.32 | 5.77 | 5.68 | 152 | 5.72 | 441 |
| Huaihua,  Hu'nan | 27.2°N,  109.1°E | 2009 | CS | R | | | MW | 23.0 | 118.0 | 31.9 | 98.0 | 5.6 | 180 | 75 | 75 | 5.25 | 7.16 | 7.96 | 8.27 | 226 | 8.25 | 434 |
|  |  |  | YE | R | | | MW | 15.8 | 180.7 | 24.1 | 78.0 | 5.5 | 180 | 75 | 75 | 5.28 | 7.16 | 7.96 | 8.28 | 227 | 8.25 | 436 |
|  | 27.5°N,  110.4°E | 2008 | YE | R | | | MW | 15.9 | 67.0 | 9.4 | 89.0 | 6.9 | 180 | 75 | 90 | 3.63 | 4.40 | 5.58 | 5.00 | 181 | 5.25 | 555 |
|  |  | 2009 | YE | R | | | MW | 9.0 | 60.0 | 13.5 | 68.0 | 6.8 | 180 | 75 | 90 | 4.35 | 5.33 | 6.45 | 5.84 | 177 | 6.16 | 464 |
|  |  | 2010 | CS | R | | | MP | 11.4 | 125.0 | 20.2 | 89.0 | 6.9 | 180 | 75 | 90 | 4.58 | 5.10 | 6.15 | 5.67 | 180 | 5.84 | 497 |
|  |  |  | CS | R | | | MW | 12.0 | 117.0 | 8.0 | 78.0 | 6.0 | 180 | 75 | 90 | 4.80 | 5.33 | 6.45 | 6.15 | 208 | 6.22 | 531 |
|  | 28.3°N,  110.2°E | 2010 | YE | R | | | MW | 13.9 | 152.0 | 19.5 | 136.0 | 6.5 | 180 | 75 | 90 | 6.57 | 7.57 | 8.04 | 7.43 | 148 | 7.94 | 311 |
|  |  |  | PS | R | | | MW | 27.1 | 174.0 | 9.2 | 106.0 | 6.5 | 180 | 75 | 90 | 5.53 | 6.52 | 8.37 | 7.91 | 223 | 8.07 | 438 |
|  |  |  | PS | R | | | MW | 24.4 | 142.0 | 10.4 | 108.0 | 6.6 | 180 | 75 | 90 | 6.07 | 6.95 | 7.54 | 7.09 | 159 | 7.40 | 354 |
|  | 27.3°N,  109.4°E | 2008 | PS | R | | | MW | 26.5 | 88.0 | 10.2 | 105.0 | 7.1 | 180 | 75 | 90 | 5.78 | 6.80 | 7.34 | 7.28 | 188 | 7.34 | 410 |
|  |  | 2010 | PS | R | | | MW | 22.6 | 123.0 | 12.5 | 106.0 | 7.4 | 180 | 75 | 90 | 4.95 | 6.47 | 7.10 | 7.28 | 212 | 7.28 | 462 |
|  |  |  | PS | R | | | MW | 29.6 | 134.3 | 29.5 | 145.0 | 7.7 | 180 | 75 | 90 | 3.92 | 5.48 | 6.20 | 6.37 | 215 | 6.37 | 535 |
|  | 26.1°N,  109.5°E | 2010 | PS | R | | | MW | 22.7 | 111.3 | 23.7 | 89.0 | 6.5 | 150 | 75 | 90 | 4.37 | 5.52 | 5.89 | 5.00 | 118 | 5.84 | 359 |
| Loudi,  Hu'nan | 27.4°N,  111.4°E | 2006 | PS | R | | | MW | 13.6 | 153.2 | 31.8 | 150.0 | 6.5 | 225 | 75 | 105 | 4.32 | 5.95 | 6.61 | 6.65 | 245 | 6.71 | 579 |
|  |  |  | PS | I | | | MW | 11.9 | 78.6 | 32.7 | 55.0 | 7.0 | 240 | 72 | 135 | 5.28 | 7.01 | 7.56 | 7.25 | 227 | 7.59 | 474 |
|  |  |  | PS | R | | | MP | 17.7 | 79.6 | 16.0 | 56.0 | 7.3 | 225 | 75 | 105 | 3.79 | 5.74 | 6.41 | 6.53 | 250 | 6.59 | 603 |
|  | 27.4°N,  111.2°E | 2007 | PS | R | | | MW | 20.9 | 129.0 | 8.1 | 124.0 | 8.1 | 150 | 75 | 75 | 6.46 | 6.75 | 7.17 | 6.33 | 92 | 7.01 | 256 |
|  |  | 2008 | YE | R | | | MW | 14.4 | 60.0 | 15.3 | 136.0 | 7.7 | 210 | 90 | 105 | 4.40 | 5.67 | 5.88 | 6.06 | 216 | 6.05 | 566 |
|  |  |  | YE | R | | | MW | 12.0 | 98.5 | 9.2 | 158.0 | 8.0 | 210 | 90 | 105 | 4.98 | 6.41 | 7.11 | 7.05 | 225 | 7.14 | 499 |
|  |  |  | YE | R | | | MW | 17.1 | 111.0 | 8.8 | 112.0 | 6.2 | 210 | 90 | 105 | 5.96 | 7.14 | 8.15 | 7.22 | 184 | 7.86 | 376 |
|  | 27.4°N,  111.3°E | 2008 | YB | R | | | MW | 25.8 | 145.6 | 8.4 | 106.0 | 6.1 | 240 | 90 | 105 | 5.13 | 5.60 | 6.06 | 5.60 | 164 | 5.86 | 458 |
|  |  |  | YE | R | | | MW | 20.6 | 136.0 | 19.8 | 90.0 | 7.2 | 210 | 90 | 105 | 6.99 | 7.70 | 8.10 | 7.85 | 175 | 8.00 | 354 |
|  |  |  | YE | R | | | MW | 21.9 | 130.0 | 8.9 | 127.0 | 7.8 | 210 | 90 | 105 | 6.86 | 7.95 | 8.25 | 7.95 | 179 | 8.25 | 350 |
|  |  | 2009 | YE | R | | | MW | 28.2 | 117.0 | 16.5 | 67.0 | 7.4 | 210 | 90 | 105 | 6.23 | 7.44 | 7.91 | 7.95 | 220 | 7.98 | 437 |
|  |  |  | YE | R | | | MW | 14.1 | 62.0 | 10.3 | 105.0 | 6.4 | 240 | 90 | 105 | 4.73 | 4.95 | 5.36 | 5.28 | 148 | 5.11 | 484 |
| Shaoyang,  Hu'nan | 27.2°N,  111.3°E | 2007 | LS | R | | | MW | 22.4 | 114.3 | 20.9 | 86.0 | 6.9 | 180 | 75 | 90 | 5.08 | 5.78 | 6.63 | 6.58 | 218 | 6.59 | 524 |
|  |  | 2008 | YE | R | | | MW | 21.2 | 106.9 | 18.9 | 119.0 | 6.9 | 225 | 75 | 105 | 5.78 | 6.70 | 7.17 | 7.19 | 228 | 7.18 | 503 |
|  | 26.6°N,  111.2°E | 2007 | YE | R | | | MW | 19.3 | 114.0 | 33.5 | 68.0 | 6.4 | 180 | 75 | 90 | 5.55 | 6.22 | 6.99 | 5.47 | 124 | 6.73 | 323 |
|  |  | 2008 | Lato. | R | | | MW | 17.5 | 78.0 | 25.0 | 144.0 | 6.5 | 180 | 75 | 90 | 6.95 | 7.89 | 8.57 | 8.26 | 175 | 8.45 | 335 |
|  |  |  | Lato. | R | | | MW | 27.3 | 126.0 | 36.9 | 63.0 | 7.8 | 180 | 75 | 90 | 5.44 | 7.24 | 7.70 | 7.70 | 189 | 7.86 | 385 |
|  | 26.4°N,  110.4°E | 2007 | YB | R | | | MW | 30.4 | 157.0 | 28.4 | 170.0 | 6.8 | 180 | 75 | 90 | 5.75 | 6.30 | 7.02 | 6.97 | 211 | 6.96 | 481 |
|  |  | 2008 | YE | R | | | MW | 31.5 | 150.0 | 13.1 | 80.0 | 5.7 | 180 | 75 | 90 | 4.21 | 5.56 | 7.47 | 7.30 | 246 | 7.41 | 527 |
|  |  | 2009 | YE | I | | | MW | 25.9 | 89.7 | 29.8 | 172.4 | 7.9 | 180 | 75 | 90 | 5.32 | 7.26 | 8.71 | 7.97 | 186 | 8.47 | 352 |
|  |  | 2010 | YE | R | | | MW | 30.1 | 145.0 | 15.0 | 132.0 | 7.8 | 180 | 75 | 90 | 6.09 | 7.75 | 8.04 | 7.87 | 172 | 8.16 | 342 |
|  |  |  | YE | R | | | MW | 29.9 | 72.0 | 14.6 | 114.5 | 7.6 | 180 | 75 | 90 | 5.86 | 7.52 | 7.96 | 7.63 | 170 | 8.01 | 345 |
|  | 26.4°N,  110.5°E | 2009 | CS | R | | | MP | 26.8 | 95.0 | 44.0 | 102.0 | 7.8 | 180 | 75 | 90 | 4.64 | 7.25 | 7.98 | 7.77 | 188 | 8.15 | 370 |
|  |  |  | CS | R | | | MW | 29.5 | 137.0 | 34.2 | 147.0 | 7.7 | 180 | 75 | 90 | 6.09 | 7.60 | 7.76 | 6.93 | 141 | 7.84 | 304 |
|  |  |  | YE | R | | | MW | 27.5 | 75.0 | 38.2 | 138.0 | 6.3 | 180 | 75 | 90 | 6.09 | 7.60 | 7.76 | 6.93 | 141 | 7.84 | 304 |
|  |  |  | PS | R | | | MW | 23.3 | 148.0 | 9.3 | 103.0 | 6.4 | 180 | 75 | 90 | 5.94 | 7.60 | 8.04 | 7.72 | 171 | 8.09 | 343 |
|  |  |  | PS | R | | | MW | 22.9 | 144.3 | 36.1 | 154.6 | 7.1 | 180 | 75 | 90 | 6.02 | 7.54 | 8.12 | 7.78 | 174 | 8.11 | 347 |
|  | 26.6°N,  111.2°E | 2010 | PS | R | | | MW | 23.5 | 167.0 | 32.5 | 134.0 | 5.3 | 150 | 75 | 75 | 7.70 | 8.05 | 8.44 | 7.77 | 98 | 8.29 | 224 |
| Yueyang,  Hu'nan | 28.5°N,  113.0°E | 2007 | PS | R | | | MW | 16.9 | 98.4 | 16.0 | 118.0 | 6.0 | 180 | 75 | 90 | 5.43 | 7.53 | 8.87 | 8.38 | 193 | 8.75 | 352 |
| Zhangjiajie,  Hu'nan | 29.3°N,  111.1°E | 2006 | PS | R | | | MW | 22.4 | 114.5 | 16.9 | 63.0 | 5.8 | 180 | 75 | 90 | 6.61 | 7.45 | 8.23 | 7.02 | 137 | 7.97 | 293 |
|  |  |  | PS | R | | | MW | 10.6 | 115.4 | 13.5 | 136.0 | 5.6 | 180 | 90 | 120 | 6.41 | 6.97 | 7.66 | 7.11 | 150 | 7.42 | 337 |
|  |  |  | PS | R | | | MW | 19.8 | 113.6 | 21.0 | 103.0 | 5.5 | 200 | 72 | 108 | 6.43 | 6.95 | 7.72 | 7.09 | 162 | 7.43 | 357 |
|  |  | 2007 | PS | I | | | MW | 18.9 | 89.7 | 8.9 | 181.0 | 5.8 | 180 | 75 | 90 | 6.17 | 7.81 | 8.57 | 8.50 | 197 | 8.63 | 364 |
|  |  |  | PS | R | | | MP | 10.8 | 79.5 | 11.4 | 136.0 | 7.8 | 200 | 72 | 108 | 8.01 | 8.72 | 9.53 | 7.06 | 124 | 9.32 | 233 |
|  |  |  | PS | R | | | MW | 10.5 | 69.9 | 17.5 | 74.0 | 8.0 | 180 | 75 | 90 | 7.37 | 8.11 | 8.72 | 7.96 | 141 | 8.52 | 280 |
|  | 29.3°N,  111.0°E | 2008 | YE | R | | | MW | 25.3 | 79.5 | 18.9 | 82.0 | 7.9 | 195 | 75 | 105 | 4.88 | 6.45 | 6.98 | 6.23 | 168 | 6.94 | 394 |
|  |  |  | YE | R | | | MW | 15.3 | 116.3 | 19.8 | 114.0 | 8.0 | 165 | 45 | 75 | 6.29 | 7.33 | 8.28 | 7.29 | 142 | 8.00 | 300 |
|  |  | 2009 | YE | R | | | MW | 20.5 | 114.5 | 17.0 | 131.0 | 7.7 | 225 | 105 | 120 | 5.03 | 6.86 | 7.63 | 7.59 | 243 | 7.72 | 499 |
|  |  |  | YB | R | | | MW | 16.9 | 123.6 | 14.9 | 114.0 | 8.0 | 225 | 105 | 120 | 6.45 | 7.34 | 8.58 | 6.90 | 171 | 8.13 | 342 |
|  |  | 2010 | YE | R | | | MW | 13.0 | 132.4 | 17.0 | 64.0 | 7.9 | 180 | 75 | 105 | 5.81 | 7.19 | 8.92 | 8.26 | 199 | 8.57 | 370 |
|  |  |  | YE | R | | | MW | 19.8 | 145.8 | 17.6 | 111.0 | 7.8 | 200 | 72 | 108 | 5.64 | 6.68 | 7.74 | 6.29 | 157 | 7.38 | 351 |
|  | 29.2°N,  110.1°E | 2007 | YE | R | | | MW | 25.6 | 96.8 | 16.2 | 132.0 | 6.8 | 180 | 75 | 90 | 3.38 | 6.05 | 6.85 | 6.67 | 191 | 7.02 | 435 |
|  |  |  | YE | R | | | MW | 26.1 | 97.4 | 15.4 | 83.0 | 7.0 | 206 | 74 | 108 | 4.29 | 5.39 | 5.71 | 5.56 | 187 | 5.73 | 523 |
|  |  |  | LS | R | | | MW | 24.5 | 79.5 | 12.5 | 146.0 | 5.9 | 206 | 74 | 108 | 3.20 | 4.60 | 5.00 | 5.15 | 222 | 5.16 | 682 |
|  |  |  | YE | R | | | MW | 25.2 | 89.3 | 20.5 | 78.0 | 5.9 | 180 | 75 | 90 | 3.23 | 5.48 | 6.52 | 5.34 | 164 | 6.37 | 421 |
|  |  | 2008 | YE | R | | | MW | 16.3 | 91.3 | 18.3 | 84.0 | 5.7 | 180 | 75 | 90 | 4.28 | 6.08 | 6.58 | 6.30 | 176 | 6.65 | 428 |
|  |  | 2009 | Lato. | R | | | MW | 22.1 | 125.4 | 20.7 | 158.0 | 6.9 | 180 | 75 | 90 | 4.82 | 5.92 | 6.80 | 6.43 | 182 | 6.65 | 440 |
|  | 29.1°N,  110.1°E | 2008 | Lato. | R | | | MW | 21.6 | 126.2 | 21.2 | 176.0 | 6.8 | 180 | 75 | 90 | 3.75 | 6.18 | 6.86 | 6.91 | 198 | 7.09 | 445 |
|  |  |  | YB | R | | | MW | 27.3 | 108.8 | 23.0 | 125.0 | 6.9 | 180 | 75 | 90 | 4.83 | 6.69 | 7.71 | 7.46 | 195 | 7.69 | 405 |
|  |  |  | YE | R | | | MW | 31.2 | 148.1 | 20.5 | 104.0 | 6.5 | 180 | 75 | 90 | 6.02 | 8.15 | 8.44 | 8.50 | 186 | 8.70 | 343 |
|  |  |  | YE | I | | | MW | 20.3 | 89.2 | 32.9 | 113.0 | 6.5 | 180 | 75 | 90 | 3.56 | 5.36 | 6.44 | 5.17 | 159 | 6.20 | 422 |
|  |  | 2009 | YE | R | | | MW | 23.9 | 143.6 | 16.0 | 114.0 | 6.5 | 180 | 75 | 90 | 5.00 | 5.76 | 6.48 | 6.37 | 196 | 6.40 | 489 |
|  |  |  | YE | R | | | MW | 20.6 | 148.1 | 15.1 | 123.0 | 6.2 | 180 | 75 | 90 | 3.72 | 5.34 | 6.80 | 6.29 | 197 | 6.58 | 477 |
|  | 30.1°N,  115.1°E | 2005 | CS | R | | | MP | 14.5 | 85.4 | 44.0 | 143.0 | 6.6 | 180 | 75 | 90 | 6.61 | 7.45 | 8.23 | 7.02 | 137 | 7.97 | 293 |
|  |  | 2006 | CS | R | | | MW | 15.9 | 96.5 | 28.0 | 125.0 | 6.7 | 200 | 72 | 108 | 5.64 | 6.68 | 7.74 | 6.29 | 157 | 7.38 | 351 |
|  | 29.1°N,  110.1°E | 2009 | YE | R | | | MW | 21.1 | 145.1 | 10.8 | 132.0 | 6.5 | 180 | 75 | 90 | 3.79 | 5.59 | 5.63 | 5.84 | 184 | 5.92 | 499 |
| Xiangxi,  Hu'nan | 28.4°N,  109.4°E | 2008 | PS | R | | | MW | 16.8 | 79.0 | 22.8 | 159.0 | 5.5 | 180 | 75 | 90 | 4.90 | 5.13 | 5.59 | 5.27 | 127 | 5.38 | 410 |
|  | 28.3°N,  109.3°E | 2006 | PS | R | | | MW | 20.0 | 152.6 | 26.7 | 167.0 | 6.5 | 180 | 75 | 90 | 4.56 | 6.30 | 7.65 | 6.89 | 182 | 7.40 | 396 |
|  |  |  | PS | R | | | MW | 10.1 | 96.8 | 21.1 | 118.0 | 5.8 | 180 | 75 | 90 | 7.73 | 8.43 | 8.43 | 8.34 | 135 | 8.47 | 272 |
|  |  |  | PS | R | | | MW | 13.2 | 114.5 | 35.7 | 155.0 | 5.9 | 165 | 83 | 90 | 3.81 | 5.66 | 5.30 | 3.52 | 111 | 5.70 | 353 |
|  |  | 2007 | PS | R | | | MW | 18.5 | 113.2 | 28.7 | 116.0 | 6.4 | 165 | 90 | 120 | 5.85 | 6.84 | 6.84 | 5.67 | 107 | 6.97 | 282 |
|  |  | 2008 | PS | R | | | MW | 22.1 | 112.3 | 17.1 | 123.0 | 6.3 | 165 | 90 | 120 | 5.96 | 6.40 | 6.47 | 5.56 | 89 | 6.51 | 270 |
|  |  | 2009 | PS | R | | | MW | 17.6 | 98.0 | 14.6 | 120.0 | 6.6 | 165 | 90 | 120 | 5.24 | 6.25 | 7.15 | 6.18 | 141 | 6.88 | 346 |
|  |  | 2010 | PS | I | | | MW | 10.3 | 69.0 | 15.5 | 62.0 | 6.6 | 180 | 75 | 90 | 4.56 | 6.30 | 7.65 | 6.89 | 182 | 7.40 | 396 |
|  | 28.2°N,  109.4°E | 2009 | PS | R | | | MP | 18.8 | 87.7 | 36.2 | 135.0 | 6.8 | 180 | 75 | 90 | 7.30 | 8.18 | 9.40 | 8.84 | 185 | 9.09 | 327 |
|  |  | 2010 | PS | R | | | MW | 16.9 | 66.9 | 13.8 | 110.0 | 6.6 | 180 | 75 | 90 | 7.30 | 8.15 | 8.71 | 8.56 | 180 | 8.65 | 335 |
|  |  |  | YE | R | | | MW | 23.7 | 91.4 | 18.7 | 170.0 | 6.1 | 180 | 75 | 90 | 7.36 | 8.30 | 8.85 | 8.61 | 172 | 8.78 | 318 |
|  | 29.3°N,  109.3°E | 2007 | YE | R | | | MW | 18.9 | 89.6 | 39.9 | 170.0 | 6.5 | 180 | 75 | 90 | 6.74 | 6.85 | 6.90 | 6.39 | 53 | 6.88 | 197 |
|  |  |  | YE | R | | | MW | 17.9 | 97.8 | 24.2 | 172.0 | 6.8 | 180 | 75 | 90 | 5.98 | 6.69 | 7.41 | 7.29 | 196 | 7.32 | 427 |
|  | 29.0°N,  109.5°E | 2007 | YB | R | | | MW | 28.8 | 118.5 | 10.8 | 96.0 | 7.5 | 180 | 75 | 90 | 6.83 | 8.02 | 8.23 | 7.58 | 141 | 8.26 | 289 |
|  |  | 2008 | YE | R | | | MW | 23.4 | 184.3 | 10.8 | 89.0 | 7.7 | 180 | 75 | 90 | 7.35 | 8.55 | 7.74 | 6.86 | 99 | 8.28 | 226 |
|  |  | 2009 | YE | R | | | MW | 26.4 | 143.6 | 10.8 | 98.0 | 7.7 | 180 | 75 | 90 | 7.48 | 8.43 | 9.24 | 8.30 | 149 | 8.99 | 277 |
|  |  | 2010 | YE | R | | | MW | 21.7 | 184.3 | 9.9 | 103.0 | 6.9 | 180 | 75 | 90 | 5.56 | 5.63 | 5.78 | 5.24 | 56 | 5.70 | 244 |
| Yueyang,  Hu'nan | 28.5°N,  113.0°E | 2007 | YE | R | | | MW | 18.9 | 78.6 | 17.9 | 180.0 | 5.9 | 180 | 75 | 90 | 5.23 | 6.77 | 7.74 | 7.42 | 190 | 7.66 | 397 |
|  |  |  | LS | R | | | MW | 17.9 | 89.6 | 20.6 | 154.0 | 6.9 | 180 | 75 | 90 | 5.27 | 7.12 | 8.01 | 7.57 | 182 | 7.96 | 368 |
|  |  | 2008 | YE | R | | | MW | 16.9 | 78.5 | 20.4 | 134.0 | 5.8 | 180 | 75 | 90 | 5.58 | 7.46 | 8.40 | 8.03 | 186 | 8.36 | 357 |
|  |  | 2009 | YE | R | | | MW | 22.1 | 96.4 | 20.5 | 89.0 | 5.9 | 180 | 75 | 90 | 5.19 | 7.05 | 8.13 | 7.69 | 188 | 8.04 | 375 |
|  | 28.4°N,  113.3°E | 2009 | Lato. | R | | | MW | 19.0 | 120.0 | 39.4 | 79.0 | 6.0 | 180 | 75 | 90 | 4.85 | 5.64 | 5.98 | 5.02 | 125 | 5.91 | 370 |
|  |  | 2010 | Lato. | R | | | MW | 26.1 | 135.2 | 14.4 | 152.0 | 5.8 | 180 | 75 | 90 | 4.10 | 5.40 | 7.60 | 6.86 | 211 | 7.15 | 468 |
| Yiyang,  Hu'nan | 28.2°N,  111.1°E | 2006 | YB | R | | | MW | 15.1 | 85.7 | 22.0 | 115.0 | 6.9 | 150 | 75 | 120 | 5.56 | 7.21 | 7.66 | 7.81 | 169 | 7.86 | 350 |
|  |  |  | YE | R | | | MW | 16.0 | 107.1 | 36.5 | 113.5 | 6.7 | 180 | 60 | 120 | 3.98 | 5.29 | 5.50 | 5.56 | 180 | 5.64 | 514 |
|  |  |  | YE | I | | | MW | 18.8 | 99.9 | 15.0 | 121.6 | 6.5 | 180 | 75 | 150 | 4.60 | 5.87 | 6.23 | 6.37 | 196 | 6.38 | 490 |
|  |  |  | YE | R | | | MW | 20.0 | 142.8 | 32.4 | 111.0 | 6.5 | 150 | 75 | 120 | 5.11 | 6.58 | 7.12 | 7.30 | 177 | 7.30 | 392 |
|  |  | 2007 | YE | R | | | MW | 15.0 | 113.0 | 28.7 | 102.0 | 6.3 | 180 | 60 | 90 | 6.76 | 7.33 | 8.10 | 8.06 | 216 | 8.05 | 425 |
|  |  |  | YE | R | | | MS | 11.7 | 86.0 | 42.8 | 150.0 | 6.8 | 150 | 60 | 90 | 6.07 | 7.36 | 8.68 | 8.56 | 190 | 8.64 | 352 |
|  |  | 2008 | CS | R | | | MP | 10.4 | 136.0 | 14.3 | 160.0 | 7.4 | 150 | 75 | 120 | 6.07 | 7.45 | 7.60 | 7.15 | 128 | 7.69 | 289 |
|  |  |  | CS | R | | | MW | 22.4 | 78.5 | 18.9 | 136.0 | 7.4 | 150 | 45 | 75 | 5.05 | 6.31 | 6.40 | 6.40 | 143 | 6.55 | 368 |
| Yongzhou,  Hu'nan | 26.1°N,  111.4°E | 2009 | YE | R | | | MW | 16.2 | 98.6 | 22.9 | 132.0 | 6.5 | 180 | 75 | 90 | 4.48 | 6.00 | 7.36 | 7.20 | 217 | 7.31 | 471 |
|  |  |  | PS | R | | | MW | 16.2 | 85.6 | 21.8 | 134.0 | 6.5 | 180 | 75 | 90 | 5.50 | 6.60 | 7.92 | 7.26 | 184 | 7.60 | 389 |
|  |  | 2010 | PS | R | | | MW | 12.6 | 78.3 | 12.5 | 105.0 | 6.8 | 180 | 75 | 90 | 5.76 | 6.56 | 7.64 | 6.68 | 155 | 7.28 | 352 |
|  |  |  | PS | R | | | MW | 19.0 | 76.3 | 38.6 | 164.0 | 6.8 | 180 | 75 | 90 | 3.30 | 5.52 | 7.65 | 7.56 | 237 | 7.68 | 489 |
|  | 27.1°N,  111.0°E | 2007 | PS | R | | | MW | 15.6 | 78.5 | 26.9 | 139.0 | 6.4 | 180 | 75 | 90 | 5.64 | 5.71 | 6.22 | 6.18 | 184 | 6.08 | 486 |
|  | 26.2°N,  111.2°E | 2005 | PS | R | | | MW | 19.7 | 114.5 | 22.0 | 140.0 | 6.5 | 180 | 72 | 105 | 5.22 | 6.75 | 8.27 | 8.07 | 221 | 8.19 | 428 |
|  |  | 2006 | PS | R | | | MW | 18.4 | 116.5 | 18.6 | 100.0 | 6.4 | 180 | 72 | 105 | 4.50 | 7.54 | 8.04 | 7.86 | 184 | 8.33 | 355 |
|  |  | 2007 | PS | R | | | MW | 19.8 | 126.5 | 28.3 | 58.0 | 6.9 | 180 | 72 | 105 | 5.26 | 6.88 | 8.30 | 8.11 | 215 | 8.23 | 414 |
|  |  | 2008 | PS | I | | | MW | 13.0 | 134.3 | 10.5 | 91.0 | 6.4 | 180 | 72 | 105 | 4.50 | 7.54 | 8.04 | 7.86 | 184 | 8.33 | 355 |
|  |  | 2009 | PS | R | | | MP | 12.1 | 148.5 | 14.3 | 59.0 | 6.4 | 180 | 72 | 105 | 5.22 | 6.75 | 7.94 | 8.07 | 237 | 8.08 | 465 |
| Enshi,  Hubei | 30.1°N,  109.3°E | 2006 | PS | R | | | MW | 18.7 | 78.9 | 8.5 | 110.0 | 7.4 | 240 | 90 | 135 | 7.86 | 9.12 | 8.91 | 8.43 | 167 | 9.11 | 299 |
|  |  | 2007 | YE | R | | | MW | 15.2 | 89.4 | 23.0 | 143.0 | 5.6 | 240 | 90 | 135 | 4.86 | 7.36 | 7.43 | 6.42 | 195 | 7.66 | 406 |
|  |  |  | YE | R | | | MW | 12.6 | 85.6 | 37.3 | 114.0 | 5.9 | 188 | 68 | 45 | 6.47 | 6.84 | 6.69 | 6.53 | 73 | 6.73 | 234 |
|  |  | 2008 | YE | R | | | MW | 12.4 | 114.8 | 28.0 | 167.0 | 5.8 | 210 | 72 | 120 | 5.69 | 6.59 | 6.84 | 7.09 | 232 | 6.99 | 526 |
|  |  |  | YB | R | | | MW | 11.3 | 156.3 | 11.4 | 169.0 | 5.9 | 210 | 72 | 120 | 4.30 | 5.34 | 5.94 | 5.99 | 232 | 5.98 | 615 |
|  |  | 2009 | YE | R | | | MW | 14.1 | 136.2 | 15.2 | 57.0 | 6.8 | 195 | 60 | 180 | 5.70 | 6.36 | 6.78 | 6.85 | 208 | 6.79 | 487 |
|  |  |  | YE | R | | | MW | 20.2 | 112.5 | 18.0 | 98.0 | 6.4 | 225 | 72 | 150 | 5.99 | 6.64 | 7.14 | 7.04 | 209 | 7.05 | 471 |
|  |  | 2010 | YE | R | | | MW | 15.8 | 123.8 | 19.0 | 73.0 | 7.8 | 165 | 26 | 180 | 6.54 | 7.30 | 8.06 | 7.49 | 147 | 7.84 | 314 |
|  |  |  | YE | R | | | MW | 16.2 | 159.0 | 8.9 | 60.0 | 6.9 | 218 | 68 | 90 | 4.03 | 5.13 | 5.07 | 5.32 | 203 | 5.27 | 613 |
|  | 30.2°N,  109.3°E | 2006 | LS | R | | | MW | 10.8 | 78.7 | 31.0 | 70.0 | 6.8 | 240 | 45 | 54 | 5.63 | 8.90 | 9.17 | 6.73 | 185 | 9.40 | 316 |
|  |  |  | YE | R | | | MW | 9.1 | 103.5 | 21.8 | 65.0 | 6.7 | 240 | 63 | 54 | 4.37 | 8.10 | 9.57 | 7.73 | 219 | 9.41 | 369 |
|  |  | 2007 | YE | R | | | MW | 12.3 | 89.6 | 16.0 | 110.0 | 5.8 | 210 | 45 | 54 | 3.22 | 7.33 | 8.59 | 8.70 | 239 | 8.98 | 422 |
|  |  |  | Lato. | R | | | MW | 9.2 | 63.0 | 16.3 | 93.0 | 5.7 | 210 | 45 | 54 | 3.26 | 6.74 | 9.19 | 8.26 | 230 | 8.92 | 408 |
|  |  | 2008 | Lato. | R | | | MW | 24.1 | 64.0 | 21.3 | 150.0 | 5.4 | 210 | 45 | 54 | 4.03 | 7.30 | 7.93 | 6.90 | 192 | 8.07 | 380 |
|  |  |  | YB | R | | | MW | 13.1 | 75.9 | 26.6 | 153.0 | 5.6 | 210 | 75 | 113 | 7.90 | 8.31 | 8.31 | 7.57 | 100 | 8.35 | 226 |
|  |  |  | YE | R | | | MW | 12.1 | 92.3 | 10.0 | 144.0 | 8.0 | 180 | 60 | 135 | 5.62 | 7.64 | 7.76 | 8.17 | 201 | 8.16 | 392 |
|  | 30.2°N,  109.2°E | 2008 | YE | I | | | MW | 11.2 | 158.0 | 8.4 | 75.0 | 7.6 | 180 | 60 | 150 | 5.71 | 8.56 | 8.03 | 8.10 | 166 | 8.62 | 314 |
|  |  |  | YE | R | | | MW | 13.8 | 100.1 | 26.2 | 141.0 | 7.5 | 210 | 75 | 150 | 5.24 | 7.32 | 8.24 | 7.68 | 206 | 8.18 | 400 |
|  |  |  | YE | R | | | MW | 11.6 | 72.0 | 22.1 | 109.0 | 7.2 | 195 | 60 | 180 | 8.98 | 10.39 | 10.43 | 9.99 | 158 | 10.56 | 247 |
|  |  | 2009 | CS | R | | | MP | 13.8 | 75.3 | 26.2 | 100.0 | 6.5 | 180 | 90 | 120 | 4.23 | 6.19 | 6.74 | 6.33 | 173 | 6.80 | 412 |
|  |  |  | CS | R | | | MW | 16.5 | 123.4 | 21.8 | 120.0 | 6.5 | 180 | 60 | 120 | 2.75 | 4.65 | 8.11 | 6.58 | 204 | 7.24 | 448 |
|  |  |  | YE | R | | | MW | 14.5 | 152.0 | 29.2 | 120.0 | 6.6 | 210 | 63 | 90 | 5.17 | 7.91 | 8.62 | 8.20 | 208 | 8.75 | 378 |
|  |  | 2010 | PS | R | | | MW | 21.7 | 86.3 | 21.5 | 130.0 | 6.4 | 210 | 63 | 72 | 5.21 | 8.89 | 9.68 | 9.22 | 212 | 9.93 | 339 |
|  |  |  | PS | R | | | MW | 16.7 | 76.5 | 17.8 | 100.0 | 6.6 | 233 | 45 | 72 | 6.19 | 7.78 | 8.94 | 8.19 | 222 | 8.70 | 404 |
|  |  |  | PS | R | | | MW | 17.2 | 157.0 | 15.9 | 160.0 | 5.7 | 210 | 63 | 72 | 5.28 | 7.82 | 8.79 | 8.83 | 237 | 8.99 | 418 |
| Shiyan,  Hubei | 32.4°N,  110.5°E | 2005 | PS | R | | | MW | 23.1 | 131.0 | 15.5 | 124.0 | 6.9 | 240 | 53 | 45 | 4.19 | 7.73 | 7.22 | 6.71 | 207 | 7.83 | 420 |
|  |  |  | PS | R | | | MW | 12.2 | 110.3 | 12.0 | 141.0 | 7.4 | 188 | 54 | 81 | 7.31 | 7.58 | 7.74 | 6.89 | 89 | 7.70 | 228 |
|  |  | 2006 | PS | R | | | MW | 22.0 | 110.0 | 17.6 | 132.0 | 7.5 | 188 | 54 | 81 | 7.30 | 7.55 | 7.66 | 6.92 | 85 | 7.64 | 224 |
|  |  |  | PS | R | | | MW | 26.5 | 175.0 | 42.6 | 154.0 | 7.3 | 188 | 54 | 81 | 7.31 | 7.56 | 7.71 | 6.92 | 87 | 7.67 | 226 |
|  |  | 2007 | PS | I | | | MW | 17.8 | 103.9 | 17.4 | 110.5 | 6.9 | 158 | 45 | 45 | 8.55 | 9.69 | 8.46 | 6.57 | 72 | 9.43 | 166 |
|  | 32.4°N,  110.4°E | 2007 | PS | R | | | MP | 10.3 | 150.3 | 21.6 | 142.0 | 7.1 | 150 | 60 | 68 | 6.03 | 6.81 | 7.28 | 7.10 | 146 | 7.22 | 339 |
|  |  | 2008 | PS | R | | | MW | 24.3 | 87.9 | 44.3 | 87.0 | 7.2 | 150 | 60 | 90 | 7.77 | 8.10 | 6.98 | 5.65 | 31 | 7.97 | 141 |
|  |  |  | YE | R | | | MW | 27.0 | 98.6 | 8.9 | 101.0 | 7.2 | 203 | 59 | 90 | 7.35 | 8.75 | 10.01 | 7.75 | 154 | 9.61 | 265 |
|  |  | 2009 | YE | R | | | MW | 21.3 | 85.6 | 14.2 | 93.0 | 7.3 | 225 | 41 | 54 | 4.90 | 8.15 | 8.96 | 8.20 | 216 | 9.08 | 377 |
|  |  |  | YE | R | | | MW | 19.8 | 97.6 | 17.5 | 95.0 | 7.2 | 225 | 59 | 68 | 4.90 | 7.95 | 8.81 | 8.30 | 224 | 8.93 | 397 |
| Xiangfan,  Hubei | 32.0°N,  112.1°E | 2005 | YB | R | | | MW | 12.5 | 88.0 | 15.1 | 171.0 | 5.9 | 225 | 75 | 68 | 4.08 | 5.31 | 7.76 | 6.33 | 227 | 7.00 | 514 |
|  |  |  | YE | R | | | MW | 17.2 | 107.0 | 10.9 | 90.0 | 6.4 | 195 | 75 | 75 | 4.50 | 5.75 | 7.66 | 6.77 | 207 | 7.17 | 459 |
|  |  | 2006 | YE | R | | | MW | 12.5 | 120.3 | 15.6 | 113.0 | 6.3 | 188 | 54 | 45 | 4.88 | 6.57 | 7.61 | 7.65 | 225 | 7.70 | 463 |
|  |  |  | YE | R | | | MW | 20.1 | 104.6 | 13.1 | 124.0 | 6.7 | 188 | 81 | 45 | 5.18 | 6.69 | 7.34 | 7.10 | 189 | 7.33 | 413 |
|  |  | 2007 | YE | R | | | MW | 22.8 | 134.0 | 16.0 | 152.0 | 5.9 | 195 | 81 | 59 | 5.70 | 8.00 | 8.31 | 8.60 | 214 | 8.66 | 392 |
|  |  |  | LS | R | | | MW | 17.8 | 93.0 | 9.9 | 116.0 | 5.8 | 225 | 81 | 59 | 5.40 | 7.20 | 8.93 | 6.75 | 188 | 8.38 | 360 |
|  |  | 2008 | YE | R | | | MW | 17.1 | 66.3 | 14.5 | 200.0 | 6.5 | 225 | 36 | 41 | 5.81 | 7.41 | 8.94 | 8.00 | 220 | 8.57 | 407 |
|  |  |  | YE | R | | | MW | 14.5 | 126.0 | 13.6 | 128.0 | 5.5 | 188 | 81 | 90 | 4.75 | 6.27 | 7.57 | 6.80 | 186 | 7.29 | 409 |
|  |  | 2009 | Lato. | R | | | MW | 25.9 | 135.9 | 38.4 | 131.0 | 5.5 | 188 | 54 | 90 | 4.55 | 6.35 | 7.66 | 6.90 | 188 | 7.42 | 406 |
|  |  |  | Lato. | R | | | MW | 26.8 | 163.0 | 12.6 | 114.0 | 5.8 | 195 | 75 | 75 | 4.50 | 5.75 | 7.66 | 6.77 | 207 | 7.17 | 459 |
|  |  | 2010 | YB | R | | | MW | 15.6 | 122.0 | 25.9 | 152.0 | 5.8 | 188 | 54 | 45 | 4.88 | 6.57 | 7.61 | 7.65 | 225 | 7.70 | 463 |
|  |  |  | YE | R | | | MW | 26.0 | 137.0 | 15.5 | 89.0 | 5.5 | 188 | 81 | 45 | 5.18 | 6.69 | 7.34 | 7.10 | 189 | 7.33 | 413 |
| Yichang,  Hubei | 30.4°N,  111.2°E | 2005 | YE | I | | | MW | 25.4 | 121.5 | 28.6 | 87.0 | 5.8 | 240 | 120 | 180 | 4.85 | 5.45 | 5.45 | 5.69 | 184 | 5.48 | 539 |
|  |  |  | YE | R | | | MW | 19.5 | 119.9 | 28.6 | 86.0 | 5.9 | 225 | 77 | 72 | 9.00 | 9.75 | 10.01 | 8.25 | 130 | 10.03 | 224 |
|  |  | 2006 | YE | R | | | MW | 20.0 | 119.5 | 20.4 | 89.0 | 5.7 | 203 | 41 | 72 | 5.18 | 7.05 | 8.25 | 7.20 | 188 | 8.00 | 377 |
|  |  |  | YE | R | | | MS | 15.2 | 63.5 | 14.9 | 95.0 | 5.7 | 240 | 72 | 27 | 5.39 | 6.84 | 6.65 | 6.12 | 174 | 6.86 | 411 |
|  |  | 2007 | YE | R | | | MW | 28.4 | 151.0 | 24.8 | 91.0 | 6.9 | 240 | 18 | 27 | 4.43 | 5.70 | 6.59 | 5.93 | 216 | 6.38 | 537 |
|  |  |  | YE | R | | | MW | 25.1 | 136.9 | 37.9 | 90.0 | 6.5 | 225 | 30 | 60 | 5.34 | 7.24 | 7.02 | 6.24 | 170 | 7.30 | 379 |
|  |  | 2008 | YE | R | | | MW | 12.9 | 123.0 | 29.8 | 93.0 | 6.2 | 195 | 68 | 105 | 6.30 | 8.00 | 8.75 | 8.19 | 185 | 8.68 | 342 |
|  |  |  | YE | R | | | MW | 22.7 | 162.0 | 25.4 | 105.0 | 6.1 | 210 | 81 | 72 | 6.64 | 8.05 | 8.58 | 8.14 | 190 | 8.54 | 356 |
|  |  | 2009 | LS | R | | | MW | 16.4 | 115.0 | 25.0 | 116.0 | 6.1 | 210 | 81 | 72 | 6.98 | 7.44 | 7.84 | 7.74 | 178 | 7.72 | 372 |
|  |  |  | YE | R | | | MW | 21.6 | 134.0 | 21.1 | 146.0 | 6.2 | 210 | 54 | 72 | 5.82 | 6.99 | 7.19 | 7.19 | 196 | 7.29 | 429 |
| Ankang,  Shaanxi | 33.0°N,  108.2°E | 2006 | YE | R | | | MW | 25.6 | 84.3 | 23.2 | 97.0 | 6.8 | 158 | 45 | 75 | 7.67 | 8.09 | 8.22 | 8.05 | 109 | 8.17 | 244 |
|  |  |  | Lato. | R | | | MW | 34.1 | 116.8 | 19.8 | 143.0 | 6.3 | 158 | 45 | 75 | 6.30 | 8.70 | 9.23 | 9.72 | 194 | 9.68 | 320 |
|  |  | 2007 | Lato. | R | | | MW | 21.3 | 96.5 | 11.0 | 159.0 | 7.2 | 158 | 45 | 75 | 6.07 | 7.13 | 8.82 | 8.77 | 234 | 8.88 | 417 |
|  |  |  | YB | R | | | MW | 14.1 | 138.3 | 12.3 | 66.7 | 7.3 | 210 | 75 | 75 | 3.66 | 5.64 | 5.32 | 5.09 | 175 | 5.67 | 499 |
|  |  | 2008 | YE | R | | | MW | 11.2 | 136.3 | 10.9 | 77.2 | 7.3 | 240 | 90 | 90 | 6.31 | 7.56 | 8.01 | 7.66 | 211 | 7.98 | 420 |
|  |  |  | YE | I | | | MW | 16.2 | 142.0 | 41.4 | 86.0 | 7.4 | 210 | 75 | 75 | 6.18 | 8.90 | 9.19 | 8.40 | 186 | 9.39 | 318 |
|  | 32.0°N,  108.0°E | 2008 | YE | R | | | MW | 26.0 | 96.0 | 20.9 | 120.0 | 7.4 | 240 | 90 | 90 | 4.38 | 5.15 | 5.63 | 5.42 | 207 | 5.52 | 596 |
| **SW3 subregion** |  |  |  |  | | |  |  |  |  |  |  |  |  |  |  |  |  |  |  |  |  |
| Bijie,  Guizhou | 27.2°N,  105.2°E | 2009 | Sier. | R | | | MW | 10.0 | 133.0 | 26.8 | 145.0 | 8.0 | 180 | 120 | 180 | 4.87 | 5.46 | 5.59 | 5.74 | 172 | 5.65 | 497 |
|  |  |  | DLS | R | | | MM | 18.5 | 133.0 | 39.0 | 160.0 | 7.8 | 225 | 150 | 240 | 7.07 | 9.27 | 9.10 | 8.73 | 191 | 9.42 | 326 |
|  |  |  | CLS | I | | | MW | 14.4 | 144.0 | 12.6 | 168.0 | 6.9 | 210 | 135 | 210 | 6.36 | 7.22 | 8.06 | 7.97 | 235 | 7.99 | 468 |
|  | 27.1°N,  105.4°E | 2008 | FAS | R | | | MP | 11.8 | 105.6 | 18.9 | 135.6 | 6.8 | 180 | 120 | 180 | 5.69 | 6.77 | 7.40 | 7.30 | 190 | 7.38 | 415 |
|  |  |  | CS | R | | | MW | 19.8 | 106.2 | 19.8 | 157.0 | 7.9 | 225 | 150 | 240 | 6.88 | 8.61 | 10.12 | 9.67 | 247 | 9.92 | 397 |
|  |  |  | FAS | R | | | MW | 16.9 | 112.3 | 30.6 | 53.2 | 8.0 | 210 | 135 | 210 | 5.87 | 7.14 | 7.67 | 7.46 | 202 | 7.65 | 423 |
|  |  |  | FAS | R | | | MOR | 18.9 | 89.6 | 22.4 | 126.0 | 5.9 | 225 | 150 | 240 | 6.84 | 7.60 | 8.66 | 8.37 | 240 | 8.45 | 453 |
|  |  | 2009 | Lato. | R | | | MW | 18.6 | 82.0 | 19.7 | 89.0 | 6.2 | 225 | 150 | 240 | 6.33 | 9.62 | 10.01 | 9.16 | 205 | 10.25 | 320 |
|  |  |  | LRE | I | | | MOR | 20.0 | 91.0 | 39.0 | 115.0 | 6.7 | 180 | 120 | 180 | 6.26 | 7.56 | 8.11 | 8.35 | 220 | 8.30 | 422 |
|  | 28.6°N,  103.5°E | 2009 | LRE | R | | | MW | 15.9 | 142.3 | 23.6 | 130.0 | 6.9 | 225 | 150 | 240 | 4.70 | 6.24 | 7.74 | 7.39 | 255 | 7.56 | 539 |
|  | 26.5°N,  105.2°E | 2009 | LRE | R | | | MW | 16.9 | 96.3 | 23.9 | 123.0 | 6.5 | 210 | 135 | 210 | 4.30 | 5.67 | 6.44 | 6.22 | 215 | 6.39 | 537 |
|  |  |  | LRE | R | | | MW | 18.9 | 85.6 | 24.5 | 135.0 | 6.3 | 225 | 150 | 240 | 6.55 | 6.80 | 7.87 | 7.50 | 227 | 7.54 | 479 |
|  |  |  | YE | R | | | MW | 17.8 | 78.6 | 25.6 | 142.0 | 6.5 | 210 | 135 | 210 | 3.95 | 6.27 | 7.44 | 6.87 | 214 | 7.36 | 464 |
|  |  |  | YE | R | | | MW | 16.8 | 69.9 | 28.9 | 115.0 | 6.2 | 180 | 120 | 180 | 3.25 | 5.80 | 6.24 | 5.94 | 177 | 6.44 | 447 |
|  | 27.0°N,  106.0°E | 2007 | YE | R | | | MW | 22.1 | 78.9 | 24.5 | 136.0 | 7.6 | 210 | 135 | 210 | 7.26 | 7.68 | 8.36 | 8.08 | 186 | 8.14 | 369 |
|  |  |  | YE | R | | | MW | 22.5 | 96.5 | 17.9 | 125.0 | 6.6 | 210 | 135 | 210 | 7.81 | 9.89 | 10.67 | 9.98 | 196 | 10.63 | 296 |
|  |  | 2008 | PS | I | | | MW | 16.8 | 92.0 | 24.6 | 81.0 | 6.4 | 210 | 135 | 210 | 4.78 | 8.50 | 9.23 | 9.03 | 218 | 9.56 | 364 |
|  |  |  | YE | R | | | MW | 26.2 | 140.0 | 21.0 | 103.0 | 6.2 | 210 | 135 | 210 | 6.77 | 9.48 | 9.75 | 9.60 | 206 | 10.05 | 328 |
|  |  |  | YE | R | | | MM | 27.8 | 142.0 | 21.3 | 115.0 | 6.4 | 225 | 150 | 240 | 7.25 | 9.83 | 10.47 | 9.72 | 207 | 10.54 | 314 |
|  | 26.5°N,  104.2°E | 2008 | BE | R | | | MOR | 27.1 | 155.3 | 11.0 | 82.0 | 6.8 | 210 | 135 | 180 | 4.46 | 6.12 | 8.23 | 6.85 | 207 | 7.63 | 434 |
|  |  |  | BE | R | | | MW | 28.4 | 138.0 | 12.0 | 121.0 | 5.6 | 180 | 120 | 150 | 4.09 | 5.23 | 6.84 | 6.07 | 188 | 6.44 | 471 |
|  |  |  | YE | I | | | MW | 24.9 | 101.0 | 14.7 | 96.0 | 6.5 | 225 | 150 | 225 | 6.43 | 8.05 | 9.08 | 8.35 | 212 | 8.89 | 381 |
|  | 27.3°N,  106.1°E | 2009 | YE | R | | | MW | 19.6 | 84.2 | 37.2 | 180.0 | 6.2 | 210 | 135 | 210 | 7.55 | 9.28 | 9.63 | 9.75 | 220 | 9.82 | 357 |
|  | 26.4°N,  115.5°E | 2006 | PS | R | | | MP | 23.5 | 137.0 | 18.2 | 199.8 | 6.5 | 225 | 150 | 240 | 6.00 | 7.56 | 7.62 | 7.80 | 220 | 7.85 | 447 |
|  |  |  | YE | R | | | MW | 28.0 | 77.0 | 10.2 | 158.9 | 7.1 | 225 | 150 | 240 | 5.94 | 7.49 | 7.55 | 7.71 | 219 | 7.77 | 449 |
|  |  |  | YB | R | | | MW | 25.6 | 142.0 | 10.2 | 138.1 | 6.2 | 225 | 150 | 240 | 7.42 | 9.50 | 9.46 | 8.72 | 181 | 9.69 | 303 |
|  |  | 2008 | YB | R | | | MM | 29.5 | 103.9 | 16.3 | 175.1 | 6.0 | 210 | 135 | 210 | 7.02 | 9.66 | 10.07 | 9.66 | 200 | 10.27 | 312 |
|  |  |  | YB | R | | | MW | 28.4 | 68.0 | 8.2 | 125.3 | 6.5 | 210 | 135 | 210 | 5.51 | 7.25 | 7.54 | 7.03 | 182 | 7.62 | 387 |
|  |  |  | YB | I | | | MW | 23.4 | 79.0 | 14.2 | 106.0 | 7.1 | 180 | 120 | 180 | 7.02 | 9.66 | 10.06 | 9.66 | 173 | 10.27 | 275 |
| Liupanshui,  Guizhou | 25.4°N,  104.3°E | 2007 | YB | R | | | MW | 14.0 | 105.0 | 26.8 | 160.0 | 6.9 | 210 | 135 | 210 | 6.78 | 7.75 | 7.95 | 7.82 | 180 | 7.98 | 366 |
|  |  | 2008 | YB | R | | | MW | 13.3 | 105.3 | 26.4 | 114.0 | 6.9 | 210 | 135 | 210 | 4.65 | 6.43 | 8.37 | 7.47 | 222 | 7.95 | 445 |
|  |  |  | YB | R | | | MW | 12.6 | 106.4 | 10.5 | 123.0 | 7.1 | 180 | 120 | 180 | 5.05 | 6.83 | 7.37 | 7.25 | 185 | 7.47 | 400 |
|  |  | 2009 | PS | R | | | MW | 22.7 | 108.7 | 17.4 | 139.0 | 7.8 | 210 | 102 | 105 | 5.18 | 7.82 | 8.87 | 7.88 | 198 | 8.79 | 361 |
|  |  |  | YB | R | | | MW | 20.0 | 125.0 | 9.1 | 141.0 | 6.5 | 225 | 105 | 120 | 6.24 | 9.18 | 9.75 | 9.29 | 218 | 9.94 | 350 |
| Qianxi,  Guizhou | 25.3°N,  105.1°E | 2008 | YE | R | | | MW | 21.8 | 121.0 | 8.8 | 100.0 | 5.4 | 210 | 75 | 90 | 8.20 | 9.40 | 10.24 | 9.81 | 203 | 10.09 | 322 |
| Leshan,  Sichuan | 29.4°N,  103.3°E | 2006 | YE | R | | | MW | 18.3 | 157.0 | 34.4 | 152.0 | 6.4 | 210 | 90 | 105 | 6.45 | 6.74 | 7.25 | 6.96 | 153 | 7.03 | 364 |
|  |  | 2008 | PS | R | | | MW | 14.5 | 66.7 | 25.2 | 110.0 | 6.2 | 210 | 90 | 120 | 6.75 | 7.44 | 8.02 | 7.59 | 175 | 7.84 | 364 |
|  | 28.6°N,  103.5°E | 2008 | PS | R | | | MW | 27.8 | 114.3 | 29.8 | 89.0 | 6.5 | 225 | 105 | 45 | 4.84 | 5.95 | 6.61 | 6.38 | 219 | 6.53 | 535 |
|  |  |  | PS | R | | | MW | 22.4 | 89.7 | 25.6 | 96.0 | 6.6 | 180 | 90 | 90 | 5.17 | 8.51 | 8.87 | 9.04 | 192 | 9.33 | 331 |
|  |  |  | PS | I | | | MM | 18.9 | 78.9 | 26.5 | 135.0 | 7.0 | 180 | 90 | 90 | 4.60 | 6.87 | 7.74 | 7.94 | 214 | 7.99 | 427 |
|  |  | 2009 | PS | R | | | MP | 19.8 | 116.5 | 25.7 | 105.0 | 7.0 | 180 | 90 | 90 | 7.01 | 7.17 | 7.29 | 6.74 | 72 | 7.23 | 219 |
|  |  |  | LRE | R | | | MW | 16.5 | 126.5 | 26.5 | 106.0 | 6.9 | 180 | 90 | 90 | 6.30 | 8.84 | 8.34 | 7.64 | 144 | 8.82 | 277 |
|  |  | 2010 | LRE | R | | | MW | 18.9 | 139.8 | 26.3 | 124.0 | 6.8 | 180 | 90 | 90 | 3.40 | 7.80 | 7.60 | 6.00 | 152 | 8.17 | 311 |
| Liangshan,  Sichuan | 26.4°N,  102.3°E | 2007 | LRE | R | | | MW | 11.7 | 62.1 | 25.7 | 104.0 | 5.8 | 255 | 105 | 60 | 7.36 | 9.25 | 9.58 | 8.26 | 194 | 9.62 | 324 |
|  |  |  | LRE | R | | | MW | 14.0 | 133.0 | 40.3 | 110.0 | 6.6 | 255 | 105 | 60 | 6.85 | 8.74 | 9.08 | 8.61 | 224 | 9.16 | 390 |
|  | 27.5°N,  102.2°E | 2006 | LRE | R | | | MW | 16.0 | 130.0 | 15.2 | 90.0 | 7.7 | 150 | 36 | 135 | 5.45 | 5.76 | 5.84 | 5.94 | 90 | 5.89 | 304 |
|  | 26.4°N,  102.3°E | 2008 | CLS | R | | | MW | 12.5 | 115.2 | 32.5 | 150.0 | 7.1 | 240 | 90 | 60 | 6.47 | 7.99 | 8.49 | 7.18 | 201 | 8.41 | 383 |
|  |  |  | FAS | R | | | MOR | 20.1 | 89.0 | 37.2 | 114.0 | 7.1 | 240 | 90 | 60 | 6.63 | 8.18 | 8.77 | 7.37 | 203 | 8.66 | 375 |
| Meishan,  Sichuan | 29.5°N,  103.2°E | 2008 | LS | I | | | MM | 14.4 | 84.0 | 19.6 | 87.0 | 5.9 | 240 | 117 | 90 | 5.23 | 6.17 | 5.16 | 4.45 | 108 | 5.78 | 346 |
| Panzhihua,  Sichuan | 26.5°N,  102.1°E | 2007 | PS | R | | | MM | 22.4 | 78.9 | 26.5 | 119.0 | 6.8 | 188 | 84 | 150 | 6.53 | 7.66 | 9.04 | 8.19 | 183 | 8.66 | 342 |
|  |  |  | RE | R | | | MS | 25.6 | 98.6 | 25.6 | 121.0 | 6.9 | 273 | 78 | 218 | 6.90 | 9.09 | 8.99 | 5.27 | 169 | 9.44 | 293 |
|  |  | 2008 | YE | R | | | MW | 26.5 | 115.6 | 14.9 | 145.0 | 6.0 | 234 | 90 | 162 | 7.65 | 8.81 | 10.69 | 9.65 | 234 | 10.14 | 368 |
|  |  |  | YE | I | | | MW | 24.3 | 116.5 | 28.9 | 98.0 | 6.3 | 211 | 103 | 254 | 6.55 | 9.04 | 9.39 | 8.43 | 182 | 9.52 | 310 |
| Luzhou,  Sichuan | 28.4°N,  106.0°E | 2008 | CLS | R | | | MW | 18.5 | 98.7 | 15.0 | 139.0 | 5.7 | 193 | 65 | 72 | 5.67 | 7.74 | 9.85 | 9.08 | 216 | 9.49 | 363 |
|  |  | 2009 | FAS | R | | | MOR | 12.9 | 56.0 | 18.2 | 130.0 | 7.2 | 193 | 65 | 72 | 6.16 | 7.35 | 8.10 | 5.23 | 126 | 7.98 | 278 |
|  | 28.5°N,  105.5°E | 2010 | LS | I | | | MM | 12.4 | 96.4 | 33.8 | 153.0 | 6.8 | 207 | 72 | 90 | 4.17 | 5.69 | 5.80 | 6.10 | 220 | 6.07 | 578 |
| Ya'an,  Sichuan | 29.2°N,  102.4°E | 2007 | PS | R | | | MM | 13.7 | 65.0 | 14.1 | 104.0 | 7.1 | 159 | 63 | 80 | 7.06 | 8.83 | 9.60 | 8.67 | 143 | 9.49 | 256 |
|  |  |  | RE | R | | | MS | 16.7 | 78.9 | 16.8 | 70.0 | 6.9 | 159 | 63 | 80 | 5.53 | 6.61 | 6.93 | 6.68 | 145 | 6.94 | 354 |
|  |  | 2008 | YE | R | | | MW | 12.2 | 100.0 | 33.8 | 107.0 | 6.4 | 162 | 65 | 81 | 6.03 | 6.75 | 7.56 | 6.78 | 137 | 7.29 | 323 |
|  |  |  | YE | I | | | MW | 18.8 | 110.0 | 20.5 | 148.0 | 6.5 | 159 | 63 | 80 | 7.59 | 8.82 | 9.58 | 8.69 | 138 | 9.40 | 252 |

^a^ Soil type: Lato.: Latosols; LRE: Lateritic red earths; RE: Red earths; YE: Yellow earths; YB: Yellow-brownearths; BE: Brown earths; DBE: Dark brown earths; CS: Cinnamon soils; CCS: Cumulic cinnamon soils; GS: Graycinnamon soils; DLS: Dark loessial soils; BS: Black soils; AS: Albic soils; Chern.: Chernozems; DC: Dark castanozems; Castan.: Castanozems; BP: Brown pedocals; Sier.: Sierozems; PS: Purplish soils; LS: Limestone soils; CLS: Cultivated-loessial soils; ASS: Aeolian sandy soils; MS: Meadow soils; FAS: Fluvo-aquic soils; LCFS: Lime concretion fluvo-aquic soils; ISS: Irrigating silting soil; BS: Bog soils ; PS: Paddy soils; Solon.: Solonchaks.

^b^ Irrigation: R: Rainfed; I: Irrigation.

^c^ Crop rotations: MM: Continuous maize; MW: Maize-wheat rotation; MS: Maize-soybean rotation; MP: Maize-potato rotation; MOR: Maize-oilseed rape rotation; MSP: Maize-sweet potato rotation.
