# Supplementary material for: Establishing a Regional Nitrogen Management Approach to Mitigate Greenhouse Gas Emission Intensity from Intensive Smallholder Maize Production
Source: PLoS One. 2014 May 29;9(5):e98481. doi: 10.1371/journal.pone.0098481 (PMC4038602; doi:10.1371/journal.pone.0098481)
Supplement: Table S3 — GHG emission factors of agricultural inputs. (DOCX) [file pone.0098481.s004.docx]

**Table S3** GHG emission factors of agricultural inputs.

| Input | unit | GHG emissions  (kg CO_2_ eq per unit input) | | | | References |
| --- | --- | --- | --- | --- | --- | --- |
|  |  | CO_2_ | CH_4_ | N_2_O | Total |  |
| N fertilizer production | kg N | 7.61 | 0.56 | 0.03 | 8.21 | Zhang *et al*., 2012 |
| P fertilizer production | kg P_2_O_5_ | 0.71 | 0.02 | 0.00 | 0.73 | Brentrup & Pallière, 2008 |
| K fertilizer production | kg K_2_O | 0.48 | 0.02 | 0.00 | 0.50 | Brentrup & Pallière, 2008 |
| N fertilizer transportation | kg N | 0.08 | 0.00 | 0.00 | 0.09 | Di *et al.*, 2005; IPCC, 2006; Yuan *et al.*, 2006;  National Bureau of Statistics of China, 2011a, b |
| P fertilizer transportation | kg P_2_O_5_ | 0.05 | 0.00 | 0.00 | 0.06 | Di *et al.*, 2005; IPCC, 2006; Yuan *et al.*, 2006;  National Bureau of Statistics of China, 2011a, b |
| K fertilizer transportation | kg K_2_O | 0.04 | 0.00 | 0.00 | 0.05 | Di *et al.*, 2005; IPCC, 2006; Yuan *et al.*, 2006;  National Bureau of Statistics of China, 2011a, b |
| Pesticides production and  transportation | kg | 18.28 | 0.80 | 0.05 | 19.12 | Williams *et al.*, 2006 |
| Diesel fuel | kg | 3.38 | 0.01 | 0.36 | 3.75 | IPCC, 2006; Yuan *et al.*, 2006;  National Bureau of Statistics of China, 2011a |

References

Brentrup F, Pallière C (2008) GHG emission and energy efficiency in European nitrogen fertilizer production and use. In: *IFA Proceedings No. 639*. International Fertiliser Society, York, United Kingdom

Di X, Nie Z, Zuo T (2005) Life cycle emission inventories for the fuels consumed by thermal power in China. *China Environmental Science*, **25**(5), 632-635 (in Chinese with English abstract).

IPCC (2006) Energy. In: *2006 IPCC Guidelines for National Greenhouse Gas Inventories, Prepared by the National Greenhouse Gas Inventories Programme* (eds Eggelston S, Buendia L, Miwa K, Ngara T, Tanabe K), IGES, Japan

National Bureau of Statistics of China (2011a) *China Energy Statistical Yearbook*. China Statistics Press, Beijing, China.

National Bureau of Statistics of China (2011b) *China Statistical Yearbook*. China Statistics Press, Beijing, China.

Williams AG, Audsley E, Sandars DL (2006) Determining the environmental burdens and resource use in the production of agricultural and horticultural commodities. Final report to Defra on project IS0205. Available on www.agrilca.org and www.defra.gov.uk.

Yuan B, Nie Z, Di X, Zuo T (2006) Life cycle inventories of fossil fuels in China(Ⅱ):Final life cycle inventories. *Modern Chemical Industry*, **26**(3), 59-61 (in Chinese with English abstract).

Zhang W, Dou Z, He P, Ju X, Powlson D, Chadwick D, Norse D, Lu Y *et al.* (2012) Improvements in manufacture and agricultural use of nitrogen fertilizer in China offer scope for significant reductions in greenhouse gas emissions. *Proceedings of the National Academy of Sciences*. (in press)
