# Supplementary material for: Establishing a Regional Nitrogen Management Approach to Mitigate Greenhouse Gas Emission Intensity from Intensive Smallholder Maize Production
Source: PLoS One. 2014 May 29;9(5):e98481. doi: 10.1371/journal.pone.0098481 (PMC4038602; doi:10.1371/journal.pone.0098481)
Supplement: Table S4 — The site, year, annual mean precipitation, temperature, soil organic matter (SOM), total N content, pH, N rate, grain yield, and direct N2O emissions at different experimental sites. (DOCX) [file pone.0098481.s005.docx]

**Table S4** The site, year, annual mean precipitation, temperature, soil organic matter (SOM), total N content, pH, N rate, grain yield, and direct N_2_O emissions at different experimental sites.

| Site | | Coordinate | Year | Preci. | Temp. | SOM | Total N | pH | N rate | Fertilizer type | Direct N_2_O emission | Experimental  treatments | Note ^a, b^ | Reference |
| --- | --- | --- | --- | --- | --- | --- | --- | --- | --- | --- | --- | --- | --- | --- |
|  |  |  |  | mm | ^o^C | --g kg^-1^-- | |  | kg ha^-1^ |  | kg N ha^-1^ |  |  |  |
| **Spring maize** | |  |  |  |  |  | |  |  |  |  |  |  |  |
| Shenyang, Liaoning | | 41.5°N, 122.4°E | 1994 | 700 | 7.5 | 16.2 | 0.8 | 6.4 | 0 |  | 0.43 |  |  | Gu et al., 2007; Huang et al., 1998 |
| Harbin, Heilongjiang | | 45.7°N, 126.6°E | 2008 | 533 | 3.5 | 16.2 | 1.3 | 7.1 | 0 |  | 0.21 | N rate |  | Ni et al., 2012 |
|  |  |  |  |  |  |  |  |  | 225 | Urea | 0.88 |  |  |  |
| Hailun, Heilongjiang | | 47.4°N, 126.9°E | 2000 | 500 | 4 | 48.2 | 2.6 | 6.3 | 0 |  | 0.41 | N rate |  | Ding & Wang, 2004 |
|  |  |  |  |  |  |  |  |  | 140 | Urea | 1.37 |  |  |  |
| Shenyang, Liaoning | | 41.3°N, 123.2°E | 1992 | 806 | 5.3 | 16.2 | 0.8 | 6.3 | 350 | Urea | 4.52 | N rate |  | Huang et al., 1995 |
| Jizhong, Shanxi | | 37.6°N, 112.8°E | 2009 | 430 | 9.3 | ND | ND | 8.4 | 120 | Urea | 0.78 |  |  | Liu. et al., 2011c |
| Jizhong, Shanxi | | 37.6°N, 112.8°E | 2009 | 430 | 9.3 | 16.9 | 1.8 | 7.5 | 0 |  | 0.21 | Fertilizer management | No fertilizer | Liu et al., 2011b |
|  |  |  |  |  |  |  |  |  | 180 | Urea | 0.93 |  | N |  |
|  |  |  |  |  |  |  |  |  | 180 | Urea | 1.19 |  | N+P |  |
| Yongji,  Shanxi | | 34.9N, 110.7°E | 2009 | 562 | 14.8 | 11.3 | 1.1 | 8.7 | 210 | Urea | 3.00 | Straw management | With straw | Liu et al., 2011a |
|  |  |  |  |  |  |  |  |  | 210 | Urea | 1.90 |  | No straw |  |
| Yongji,  Shanxi | | 34.9°N, 110.7°E | 2008 | 624 | 13.1 | 9.6 | 1.1 | 8.5 | 60 | Urea | 0.52 | Straw management | with straw | Liang, 2009 |
|  |  |  |  |  |  |  |  |  | 60 | Urea | 0.39 |  | with 1/2straw |  |
|  |  |  |  |  |  |  |  |  | 60 | Urea | 0.35 |  | without straw |  |
| Yanting, Sichuan | | 31.3°N, 105.4°E | 2005 | 826 | 17.3 | 12.0 | 0.9 | 8.3 | 0 |  | 0.88 | N rate |  | Xiang et al., 2007 |
|  |  |  |  |  |  |  |  |  | 150 | Urea | 2.19 |  |  |  |
|  |  |  |  |  |  |  |  |  | 250 | Urea | 2.52 |  |  |  |
|  |  |  |  |  |  | 14.3 | 0.8 | 7.7 | 0 |  | 0.90 | N fertilizer type |  |  |
|  |  |  |  |  |  |  |  |  | 150 | Urea | 2.09 |  |  |  |
|  |  |  |  |  |  |  |  |  | 150 | NH_4_(SO_4_)_2_ | 1.80 |  |  |  |
|  |  |  |  |  |  |  |  |  | 150 | KNO_3_ | 1.27 |  |  |  |
| Yanting, Sichuan | | 31.0°N, 105.4°E | 2005 | 826 | 17.3 | 14.6 | 1.0 | 8.3 | 0 |  | 0.93 | N rate |  | Zhou et al., 2012 |
|  |  |  |  |  |  |  |  |  | 150 | Urea | 1.94 |  |  |  |
|  |  |  |  |  |  |  |  |  | 250 | Urea | 2.74 |  |  |  |
|  |  |  | 2006 | 826 | 17.3 | 14.6 | 1.0 | 8.3 | 0 |  | 0.17 |  |  |  |
|  |  |  |  |  |  |  |  |  | 150 | Urea | 0.45 |  |  |  |
|  |  |  |  |  |  |  |  |  | 250 | Urea | 0.47 |  |  |  |
|  |  |  | 2007 | 826 | 17.3 | 14.6 | 1.0 | 8.3 | 0 |  | 0.13 |  |  |  |
|  |  |  |  |  |  |  |  |  | 150 | Urea | 1.15 |  |  |  |
|  |  |  |  |  |  |  |  |  | 250 | Urea | 1.31 |  |  |  |
| **Summer maize** | | | | | | | | | | | | | | |
| Fengqiu, Henan | | 35.0°N, 114.4°E | 1998 | 615 | 13.9 | ND | ND | ND | 0 |  | 0.10 | N application method* |  | Cai et al., 2002a, b |
|  |  |  |  |  |  |  |  |  | 75 | Urea | 1.10 |  | BI |  |
|  |  |  |  |  |  |  |  |  | 75 | Urea | 0.80 |  | SB |  |
|  |  |  |  |  |  |  |  |  | 200 | Urea | 2.30 |  | DP |  |
|  |  |  | 1999 | 615 | 13.9 | ND | ND | ND | 0 |  | 0.30 |  |  |  |
|  |  |  |  |  |  |  |  |  | 150 | Urea | 2.55 |  | DP |  |
|  |  |  |  |  |  |  |  |  | 150 | Urea | 2.85 |  | SB |  |
| Fengqiu, Henan | | 35.0°N, 114.4°E | 2009 | 615 | 13.9 | 12.0 | 1.5 | 8.6 | 200 | Urea | 0.77 |  |  | Ding et al., 2010 |
| Fengqiu, Henan | | 35.0°N, 114.4°E | 2002 | 615 | 13.9 | 11.7 | 0.4 | 8.5 | 150 | Urea | 0.50 | Fertilizer management | N+P+K | Meng et al., 2005 |
|  |  |  |  |  |  |  |  |  | 0 |  | 0.08 |  | No fertilizer |  |
|  |  |  |  |  |  |  |  |  | 150 | Urea | 0.42 |  | N+P |  |
|  |  |  |  |  |  |  |  |  | 150 | Urea | 0.37 |  | N+K |  |
|  |  |  |  |  |  |  |  |  | 0 |  | 0.06 |  | P+K |  |
| Haidian, Beijing | 40.1°N, 116.5°E | | 2001 | 404 | 11.5 | 21.3 | 1.2 | 7.9 | 0 |  | 0.15 | N rate | CK | Ju et al., 2011 |
|  |  |  |  |  |  |  |  |  | 75 | Urea | 0.88 |  | Opt. N |  |
|  |  |  |  |  |  |  |  |  | 300 | Urea | 2.40 |  | Conv. N |  |
|  |  |  | 2002 |  | 11.5 | 21.3 | 1.2 | 7.9 | 0 |  | 0.15 | N rate | CK |  |
|  |  |  |  |  |  |  |  |  | 62 | Urea | 0.44 |  | Opt. N |  |
|  |  |  |  |  |  |  |  |  | 300 | Urea | 1.71 |  | Conv. N |  |
|  |  |  | 2003 |  | 11.5 | 21.3 | 1.2 | 7.9 | 0 |  | 0.15 | N rate | CK |  |
|  |  |  |  |  |  |  |  |  | 50 | Urea | 0.10 |  | Opt. N |  |
|  |  |  |  |  |  |  |  |  | 300 | Urea | 0.84 |  | Conv. N |  |
|  |  |  | 2004 |  | 11.5 | 21.3 | 1.2 | 7.9 | 0 |  | 0.16 | N rate | CK |  |
|  |  |  |  |  |  |  |  |  | 122 | Urea | 0.52 |  | Opt. N |  |
|  |  |  |  |  |  |  |  |  | 300 | Urea | 0.91 |  | Conv. N |  |
|  |  |  | 2005 |  |  |  |  |  | 0 |  | 0.53 | N fertilizer type |  |  |
|  |  |  |  |  |  |  |  |  | 250 | NH_4_(SO_4_)_2_ | 3.52 |  |  |  |
|  |  |  |  |  |  |  |  |  | 250 | Ca(NO_3_)_2_ | 0.87 |  |  |  |
|  |  |  | 2006 |  |  |  |  |  | 0 |  | 0.43 | N fertilizer type |  |  |
|  |  |  |  |  |  |  |  |  | 250 | NH_4_(SO_4_)_2_ | 4.52 |  |  |  |
|  |  |  |  |  |  |  |  |  | 250 | Ca(NO_3_)_2_ | 0.51 |  |  |  |
|  |  |  | 2007 |  |  |  |  |  | 0 |  | 0.27 | N fertilizer type |  |  |
|  |  |  |  |  |  |  |  |  | 250 | NH_4_(SO_4_)_2_ | 1.31 |  |  |  |
|  |  |  |  |  |  |  |  |  | 250 | Ca(NO_3_)_2_ | 0.38 |  |  |  |
| Fengqiu, Henan | | 35.0°N, 114.4°E | 2007 | 615 | 13.9 | 9.7 | 0.7 | 8.3 | 0 | Urea | 0.08 | Fertilizer management (interrow + row soil) | No fertilizer | Cai et al., 2012 |
|  |  |  |  |  |  |  |  |  | 150 | Urea | 1.22 |  | N+K |  |
|  |  |  |  |  |  |  |  |  | 150 | Urea | 0.92 |  | N+P+K |  |
|  |  |  |  |  |  |  |  |  | 0 |  | 0.06 | Fertilizer management (interrow soil) | No fertilizer |  |
|  |  |  |  |  |  |  |  |  | 150 | Urea | 0.49 |  | N+K |  |
|  |  |  |  |  |  |  |  |  | 150 | Urea | 0.47 |  | N+P+K |  |
| Wangdu, Hebei | | 38.7°N, 115.2°E | 2009 | 413 | 10.5 | ND | ND | ND | 0 |  | 0.94 | N rate/straw | CK without straw | Zhang et al., 2011 |
|  |  |  |  |  |  |  |  |  | 168.2 | Urea | 2.75 |  | N without straw |  |
|  |  |  |  |  |  |  |  |  | 188.8 | Urea | 3.21 |  | N with straw |  |
| Luancheng, Hebei | | 37.8°N, 114.7°E | 2000 | 537 | 12.2 | 15.1 | 1.0 | 8.2 | 172.5 | Urea | 2.46 |  |  | Zhang et al., 2004; 2005 |
| Fengqiu, Henan | | 35.00N，114.24E | 2009 | 615 | 13.9 | 12.0 | 0.61 | ND | 0 |  | 0.32 | Tillage treatment | No-tillage | Cai et al.2011 |
|  |  |  |  |  |  |  |  |  | 0 |  | 0.27 |  | tillage |  |
| Fengqiu, Henan | | 35.0°N, 114.4°E | 1999 | 615 | 13.8 | 10.1 | 0.6 | 8.7 | 0 |  | 0.75 | N rate |  | Ding et al. 2001a |
|  |  |  |  |  |  |  |  |  | 150 | Urea | 3.12 |  |  |  |
| Fengqiu, Henan | | 35.0°N, 114.4°E | 1999 | 615 | 13.9 | 9.9 | 0.6 | 8.6 | 0 | Urea | 0.33 | N application method |  | Ding et al., 2001b; Ding et al., 2003 |
|  |  |  |  |  |  |  |  |  | 150 | Urea | 3.24 |  | SB |  |
|  |  |  |  |  |  |  |  |  | 150 | Urea | 1.46 |  | BI |  |
|  |  |  |  |  |  |  |  |  | 150 | Urea | 2.83 |  | DP |  |
| Fengqiu, Henan | | 35.0°N, 114.4°E | 2000 | 615 | 13.9 | 9.9 | 0.6 | 8.6 | 0 |  | 0.18 | N fertilizer type |  | Ding et al., 2004 |
|  |  |  |  |  |  |  |  |  | 150 | Urea | 0.56 |  |  |  |
|  |  |  |  |  |  |  |  |  | 150 | NH_4_HCO_3_ | 0.85 |  |  |  |
|  |  |  |  |  |  |  |  |  | 150 | NH_4_NO_3_ | 1.04 |  |  |  |
|  |  |  |  |  |  |  |  |  | 150 | Ca(NO_3_)_2_ | 0.24 |  |  |  |
|  |  |  |  |  |  |  |  |  | 0 |  | 0.46 | N application time |  |  |
|  |  |  |  |  |  |  |  |  | 150 | Urea | 0.67 |  | one time |  |
|  |  |  |  |  |  |  |  |  | 150 | Urea | 2.52 |  | two time |  |
| Haidian, Beijing | | 39.5°N, 116.3°E | 2002 | 436 | 11.5 | 21.4 | 1.2 | 8.0 | 80 | Urea | 0.62 | N rate |  | Gao, 2004 |
|  |  |  |  |  |  |  |  |  | 300 | Urea | 1.85 |  |  |  |
|  |  |  | 2003 |  |  |  |  |  | 80 | Urea | 0.10 |  |  |  |
|  |  |  |  |  |  |  |  |  | 300 | Urea | 0.84 |  |  |  |
| Quzhou, Hebei | | 36.5°N, 115.1°E | 2008 | 556 | 13.1 | 14.7 | 0.9 | 8.2 | 300 | Urea | 3.46 | N rate |  | Hu et al., 2011 |
|  |  |  |  |  |  |  |  |  | 250 | Urea | 2.34 |  |  |  |
|  |  |  |  |  |  |  |  |  | 185 | Urea | 1.68 |  |  |  |
| Quzhou, Hebei | | 36.5°N, 115.1°E | 2009 | 556 | 13.1 | 14.7 | 0.9 | 8.2 | 0 |  | 0.55 | Straw management |  | Hu, 2011 |
|  |  |  |  |  |  |  |  |  | 180 | Urea | 1.70 |  | without straw |  |
|  |  |  |  |  |  |  |  |  | 180 | Urea | 1.76 |  | with straw |  |
|  |  |  | 2010 |  |  |  |  |  | 0 |  | 0.34 | Straw management |  |  |
|  |  |  |  |  |  |  |  |  | 180 | Urea | 1.09 |  | without straw |  |
|  |  |  |  |  |  |  |  |  | 180 | Urea | 1.31 |  | with straw |  |
| Baoding, Hebei | | 38.1°N, 115.1°E | 2005 | 396 | 13.8 | 19.3 | 1.0 | 7.8 | 0 |  | 0.16 | N rate |  | Li, 2006 |
|  |  |  |  |  |  |  |  |  | 60 | Urea | 0.19 |  |  |  |
|  |  |  |  |  |  |  |  |  | 120 | Urea | 0.47 |  |  |  |
|  |  |  |  |  |  |  |  |  | 180 | Urea | 0.44 |  |  |  |
|  |  |  |  |  |  |  |  |  | 240 | Urea | 0.65 |  |  |  |
| Baoding, Hebei | | 38.0°N, 115.0°E | 2006 | 575 | 12.3 | 19.3 | 1.0 | 8.6 | 0 |  | 0.16 | N rate |  | Ma et al., 2012 |
|  |  |  |  |  |  |  |  |  | 60 | Urea | 0.19 |  |  |  |
|  |  |  |  |  |  |  |  |  | 120 | Urea | 0.74 |  |  |  |
|  |  |  |  |  |  |  |  |  | 180 | Urea | 0.69 |  |  |  |
|  |  |  |  |  |  |  |  |  | 240 | Urea | 1.10 |  |  |  |
| Luancheng, Hebei | | 37.8°N, 114.6°E | 1992 | 550 | 13.3 | 7.4 | 0.8 | 8.5 | 0 |  | 0.32 | N rate |  | Song et al., 1997 |
|  |  |  |  |  |  |  |  |  | 138 | Urea | 0.63 |  |  |  |
| Luancheng, Hebei | | 37.8°N, 114.6°E | 1993 | 549 | 13.3 | ND | ND | ND | 0 |  | 0.36 | N rate |  | Wang et al., 1994 |
|  |  |  |  |  |  |  |  |  | 150 | Urea | 0.54 |  |  |  |
| Hengshui, Hebei | | 38.0°N, 115.3°E | 2008 | 460 | 12.6 | 11.7 | ND | 8.0 | 0 |  | 0.86 | N rate/application time |  | Wang, 2009 |
|  |  |  |  |  |  |  |  |  | 240 | Urea | 3.16 |  | two times |  |
|  |  |  |  |  |  |  |  |  | 168 | Urea | 2.61 |  | two times |  |
|  |  |  |  |  |  |  |  |  | 168 | Urea | 2.01 |  | three times |  |
| Luancheng, Hebei | | 37.8°N, 114.6°E | 1998 | 550 | 13.3 | 12.5 | 0.9 | 8.5 | 0 |  | 0.75 | N rate |  | Zhang et al., 2001 |
|  |  |  |  |  |  |  |  |  | 100 | Urea | 1.10 |  |  |  |
|  |  |  |  |  |  |  |  |  | 200 | Urea | 0.88 |  |  |  |
|  |  |  |  |  |  |  |  |  | 300 | Urea | 1.66 |  |  |  |
| Luancheng, Hebei | | 37.8°N, 114.6°E | 1999 | 537 | 12.2 | 15.1 | 1.0 | 8.2 | 0 |  | 0.58 | N rate |  | Zhang, 2005 |
|  |  |  |  |  |  |  |  |  | 100 | Urea | 1.00 |  |  |  |
|  |  |  |  |  |  |  |  |  | 200 | Urea | 0.75 |  |  |  |
|  |  |  |  |  |  |  |  |  | 300 | Urea | 1.59 |  |  |  |
|  |  |  | 2000 | 537 | 12.2 | 15.1 | 1.0 | 8.2 | 0 |  | 0.69 | N rate |  |  |
|  |  |  |  |  |  |  |  |  | 100 | Urea | 1.97 |  |  |  |
|  |  |  |  |  |  |  |  |  | 200 | Urea | 2.46 |  |  |  |
|  |  |  |  |  |  |  |  |  | 300 | Urea | 4.51 |  |  |  |
| Quzhou, Hebei | | 36.5°N, 115.1°E | 2009 | 556.2 | 13.2 | 12.6 | 0.7 | 7.7 | 250 | Urea | 2.03 | Cropping system^#^ | Conv.W/M | Gao, 2012 |
|  |  |  |  |  |  |  |  |  | 185 | Urea | 2.57 |  | Opt.W/M |  |
|  |  |  |  |  |  |  |  |  | 135 | Urea | 1.35 |  | W/M-M |  |
|  |  |  |  |  |  |  |  |  | 210 | Urea | 1.56 |  | W/S-M |  |
|  |  |  |  |  |  |  |  |  | 95 | Urea | 1.12 |  | M |  |
|  |  |  | 2010 | 556.2 | 13.2 | 12.6 | 0.7 | 7.7 | 250 | Urea | 1.67 | Cropping system | Conv.W/M |  |
|  |  |  |  |  |  |  |  |  | 185 | Urea | 2.01 |  | Opt.W/M |  |
|  |  |  |  |  |  |  |  |  | 185 | Urea | 1.72 |  | W/M-M |  |
|  |  |  |  |  |  |  |  |  | 150 | Urea | 1.13 |  | M |  |
|  |  |  | 2011 | 556.2 | 13.2 | 12.6 | 0.7 | 7.7 | 250 | Urea | 1.76 | Cropping system | Conv.W/M |  |
|  |  |  |  |  |  |  |  |  | 185 | Urea | 1.28 |  | Opt.W/M |  |
|  |  |  |  |  |  |  |  |  | 162 | Urea | 1.29 |  | W/M-M |  |
|  |  |  |  |  |  |  |  |  | 178 | Urea | 1.01 |  | W/S-M |  |
|  |  |  |  |  |  |  |  |  | 150 | Urea | 1.11 |  | M |  |

^a^ N application method: SB: surface broadcast; BI: broadcast followed by irrigation; DP: deep-point placement

^b^ Cropping system: Conv.W/M: convention management of winter wheat/summer maize rotation system in one year; Opt.W/M: optimum management of winter wheat/summer maize rotation system in one year; W/M-M: winter wheat/summer maize in one year and spring maize in next year; W/S-M: winter wheat/soybean in one year and spring maize in next year; M: continuous spring maize in one year.

**References:**

Cai G, White RE, Chen D, Fan X, Pacholski A, Zhu Z, Ding H (2002a) Gaseous nitrogen losses from urea applied to maize on a calcareous fluvo-aquic soil in the North China Plain. *Australian Journal of Soil Research*, **40**, 737-748.

Cai G, Chen D, Ding H, Pacholski A, Fan X, Zhu Z (2002b) Nitrogen losses from fertilizers applied to maize, wheat and rice in the North China Plain. *Nutrient Cycling in Agroecosystems*, **63**, 187-195.

Cai Y, Ding W, Luo J (2012) Spatial variation of nitrous oxide emission between interrow soil and interrow plus row soil in a long-term maize cultivated sandy loam soil. *Geoderma*, **181–182**, 2-10.

Cai Y, Ding W, Zhu A, Zhang J (2011) Effects of no-tillage on N_2_O and CO_2_ emissions from sandy loam soil in the North China Plain. *Journal of Ecology and Rural Environment*, **27**(5), 1-6 (in Chinese with English abstract).

Ding H, Cai G, Wang Y, Chen D (2001a) N_2_O emission from different crop-fluvo-aquic soil systems in the North China Plain. *Agro-environmental Protection*, **20**(1), 7-9 (in Chinese with English abstract).

Ding H, Cai G, Wang Y, Chen D (2001b) Nitrification-denitrification losses of nitrogen fertilizer and N_2_O emission from maize-chao soil system in North China Plain. *Scientia Agricultura Sinica*, **34**(4), 416-421 (in Chinese with English abstract).

Ding H, Cai G, Wang Y, Chen D (2003) Nitrification-denitrification loss and N_2_O emission from maize-wheat rotation system in North China. *Journal of Agro-environmental Science*, **22**(5), 557-560 (in Chinese with English abstract).

Ding H, Wang Y (2004) Denitrification losses of nitrogen fertilizer and N_2_O emission from different crop-black soil systems in North-east China. *Journal of Agro-environmental Science*, **23**(2), 323-326 (in Chinese with English abstract).

Ding W, Yu H, Cai Z (2010) Impact of urease and nitrification inhibitors on nitrous oxide emissions from fluvo-aquic soil in the North China Plain. *Biology and Fertility of Soils*, **47**, 91-99.

Gao B (2012) *Greenhouse gases emissions and net global warming potential of different cropping systems and management practices on the North China Plain*. PhD thesis, China Agricultural University. (in Chinese with English abstract).

Gao Z (2004) *N_2_O Flux and CH_4_ Uptake of Soil in Winter Wheat and Summer Maize Rotation System*. PhD thesis, China Agricultural University. (in Chinese with English abstract).

Gu JX, Zheng XH, Wang YH, Ding WX, Zhu B, Chen X, Wang YY, Zhao ZC *et al.* (2007) Regulatory effects of soil properties on background N_2_O emissions from agricultural soils in China. *Plant and Soil*, **295**, 53-65.

Hu X (2011) *Greenhouse gases fluxes of winter wheat-summer maize rotation and mitigation strategies on the North China Plain*. PhD thesis, China Agricultural University. (in Chinese with English abstract).

Huang G, Chen G, Wu J, Huang B, Yu K (1995) N_2_O and CH_4_ fluxes from typical upland fields in northeast China. *Chinese Journal of Applied Ecology*, **6**(4), 383-386 (in Chinese with English abstract).

Ju X, Lu X, Gao Z, Chen X, Su F, Kogge M, Römheld V, Christie P *et al.* (2011) Processes and factors controlling N_2_O production in an intensively managed low carbon calcareous soil under sub-humid monsoon conditions. *Environmental Pollution*, **159**, 1007-1016.

Li X, Hu C, Zhang Y, Dong W, Ouyang Z (2006) Losses of nitrate-nitrogen from a wheat-corn rotation in north China. *Agricultural Research in the Arid Areas*, **24**(6), 7-10 (in Chinese with English abstract).

Liang W (2009) *Study of impacts of amendant plant residues on the emissions of greenhouse gases and NO from typical saline soil*. Master thesis, Southwestern University. (in Chinese with English abstract).

Liu C, Wang K, Meng S, Zheng X, Zhou Z, Han S, Chen D, Yang Z (2011a) Effects of irrigation, fertilization and crop straw management on nitrous oxide and nitric oxide emissions from a wheat–maize rotation field in northern China. *Agriculture, Ecosystems and Environment*, **140**, 226-233.

Liu Y, Li Y, Wan Y, Gao Q, Qin X, Chen D (2011b) Nitrous oxide emissions from spring-maize field under the application of different nitrogen and phosphorus fertilizers. *Journal of Agro-Environment Science*, **30**(7), 1468-1475 (in Chinese with English abstract).

Liu YT, Li YE, Wan YF, Chen DL, Gao QZ, Li Y, Qin XB (2011c) Nitrous oxide emissions from irrigated and fertilized spring maize in semi-arid northern China. *Agriculture, Ecosystems & Environment*, **141**, 287-295.

Ma Y, Ji R, Li X, Zhang L, Ju X, Zhang L (2012) Effects of N fertilization rates on the NH_3_ volatilization and N_2_O emissions from the wheat-maize rotation system in North China Plain. Ecology and Environmental Sciences, 21(2), 225-230 (in Chinese with English abstract).

Meng L, Ding W, Cai Z (2005) Long-term application of organic manure and nitrogen fertilizer on N_2_O emissions, soil quality and crop production in a sandy loam soil. *Soil Biology and Biochemistry*, **37**, 2037-2045.

Ni K, Ding W, Zaman M, Cai Z, Wang Y, Zhang X, Zhou B (2012) Nitrous oxide emissions from a rainfed-cultivated black soil in Northeast China: effect of fertilization and maize crop. *Biology and Fertility of Soils*, **48**, 973-979.

Song W, Wang S, Zeng J, Wang Z, Zhang Y (1997) Emission of nitrous oxide from dryland in Northern China. *Advances in Environmental Science*, **5**(4), 50-56 (in Chinese with English abstract).

Wang S, Song W, Su W, Zeng J, Wang Z, Zhang Y (1994) Release of N_2_O from N-fertilized corn fields. *Rural Eco-Environment*, **10**(4), 12-14 (in Chinese with English abstract).

Wang X (2009) *Nitrogen cycling and balance in winter wheat-summer corn rotation system under optimized nitrogen management*. PhD thesis, Chinese Academy of Agricultural Sciences. (in Chinese with English abstract).

Xiang H, Zhu B, Kuang F, Wang Y, Zheng X (2007) Effects of nitrogen fertilizer application on N_2_O emission in a purple soil and maize root system. *Acta Scientiae Circumstantiae*, **27**(3), 413-420 (in Chinese with English abstract).

Zhang Y (2005) *Nitrogen cycling and balance in wheat-maize rotation field in Piedmont Plain of MT Taihang in the North China Plain*. PhD thesis, China Agricultural University. (in Chinese with English abstract).

Zhang Y, Chen D, Zhang J, Edis R, Hu C, Zhu A (2004) Ammonia volatilization and denitrification losses from an irrigated maize-wheat rotation field in the North China Plain. *Pedosphere*, **14**, 533-540.

Zhang Y, Liu J, Mu Y, Pei S, Lun X, Chai F (2011) Emissions of nitrous oxide, nitrogen oxides and ammonia from a maize field in the North China Plain. *Atmospheric Environment*, **45**, 2956-2961.

Zhou M, Zhu B, Butterbach-Bahl K, Zheng X, Wang T, Wang Y (2012) Nitrous oxide emissions and nitrate leaching from a rain-fed wheat-maize rotation in the Sichuan Basin, China. *Plant and Soil*, DOI: 10.1007/s11104-012-1269-5.
