# Supplementary material for: Establishing a Regional Nitrogen Management Approach to Mitigate Greenhouse Gas Emission Intensity from Intensive Smallholder Maize Production
Source: PLoS One. 2014 May 29;9(5):e98481. doi: 10.1371/journal.pone.0098481 (PMC4038602; doi:10.1371/journal.pone.0098481)
Supplement: Table S5 — Descriptive statistics of the surveyed farms N fertilizer application rate, maize grain yield, PFPN, N balance and GHG emission intensity for 5,406 farmed fields between 2007 and 2009 in China. (DOCX) [file pone.0098481.s006.docx]

**Table S5** Descriptive statistics of the surveyed farms N fertilizer application rate, maize grain yield, PFP_N_, N balance and GHG emission intensity for 5,406 farmed fields between 2007 and 2009 in China.

|  | Mean±SD ^a^ | Minimum | 25%quartile | Median | 75%quartile | Maximum |
| --- | --- | --- | --- | --- | --- | --- |
| N rate (kg N ha^-1^) | 220±88 | 46 | 156 | 205 | 268 | 615 |
| Grain yield (Mg ha^-1^) | 7.56±1.94 | 3.39 | 6.06 | 7.95 | 8.99 | 12.00 |
| PFP_N_ (kg kg^-1^) | 36.6±19.7 | 12.0 | 22.6 | 32.3 | 55.7 | 110.0 |
| N balance (kg N ha^-1^) | 69±87 | -100 | 5 | 59 | 124 | 466 |
| GHG intensity  (kg CO_2_ eq Mg^-1^ grain) | 482±206 | 364 | 404 | 421 | 462 | 1399 |

^a^ SD: standard deviation.
