# Supplementary material for: Establishing a Regional Nitrogen Management Approach to Mitigate Greenhouse Gas Emission Intensity from Intensive Smallholder Maize Production
Source: PLoS One. 2014 May 29;9(5):e98481. doi: 10.1371/journal.pone.0098481 (PMC4038602; doi:10.1371/journal.pone.0098481)
Supplement: Text S1 — Detailed information for each of these regions. (DOCX) [file pone.0098481.s007.docx]

**Text S1** Detailed information for each of these regions.

The NE China region is located between 40–55°N and 110–135°E, and has a warm-temperate, frigid-humid, or sub-humid continental monsoon climate with warm, wet summers and long, cold winters. In NE1, maize grows in rain-fed conditions, with <2900 growing degree days (GDD, baseline 10°C), and the average maize cycle is 130 days. In NE2, maize grows in black soils, chernozems, and meadow soils. In NE3, maize is grown with irrigation, because the annual average precipitation is less than 450 mm. In NE4, maize grows in rain-fed conditions, where the average annual precipitation is 500–800 mm and the average maize cycle is 150 days.

The NCP is located between 32–41°N and 113–120°E and has a warm, semi-humid continental monsoon climate, where maize grows in rain-fed conditions. There are 1300–1900 GDD for maize in NCP1 and 1900–2100 GDD in NCP2.

The NW region is located between 34–40°N and 95–115°E. It has an arid and semi-arid continental monsoon climate with a cold winter and dry, warm summer. Maize is grown in rain-fed conditions in NW1, in a mid-temperate zone under irrigation in NW2, and in a warm-temperate zone under irrigation in NW3.

The SW region is located between 21–34°N and 97–112°E. It has a humid or sub-humid, subtropical climate, with moist, hot summers and cold winters. The most notable type of terrain consists of plains in SW1, hills and mountains in SW2, and highlands in SW3.
